# Global variation of risk thresholds for initiating statins for primary prevention of cardiovascular disease: a benefit-harm balance modelling study

**DOI:** 10.1186/s12872-020-01697-6

**Published:** 2020-09-17

**Authors:** Henock G. Yebyo, Sofia Zappacosta, Hélène E. Aschmann, Sarah R. Haile, Milo A. Puhan

**Affiliations:** 1 Department of Epidemiology, Epidemiology, Biostatistics and Prevention Institute, University of Zurich, Hirschengraben 84, CH-8001 Zurich, Switzerland; 2 School of Public Health, Mekelle University, Ayder, Mekelle, Ethiopia; 3 Institute of Medical Information Processing, Biometry and Epidemiology (IBE), Ludwig Maximilians Universität, Marchioninistrasse 15, 81377 Munich, Germany

**Keywords:** Statins, Primary prevention, Cardiovascular disease, Risk thresholds: benefit-harm analysis

## Abstract

**Background:**

We previously showed that the 10-year cardiovascular disease (CVD) risk threshold to initiate statins for primary prevention depends on the baseline CVD risk, age, sex, and the incidence of statin-related harm outcome and competing risk for non-CVD death. As these factors appear to vary across countries, we aimed in this study to determine country-specific thresholds and provide guidelines a quantitative benefit-harm assessment method for local adaptation.

**Methods:**

For each of the 186 countries included, we replicated the benefit-harm balance analysis using an exponential model to determine the thresholds to initiate statin use for populations aged 40 to 75 years, with no history of CVD. The analyses took data inputs from a priori studies, including statin effect estimates (network meta-analysis), patient preferences (survey), and baseline incidence of harm outcomes and competing risk for non-CVD (global burden of disease study). We estimated the risk thresholds above which the benefits of statins were more likely to outweigh the harms using a stochastic approach to account for statistical uncertainty of the input parameters.

**Results:**

The 5^th^ and 95^th^ percentiles of the 10-year risk thresholds above which the benefits of statins outweigh the harms across 186 countries ranged between 14 and 20% in men and 19–24% in women, depending on age (i.e., 90% of the country-specific thresholds were in the ranges stated). The median risk thresholds varied from 14 to 18.5% in men and 19 to 22% in women. The between-country variability of the thresholds was slightly attenuated when further adjusted for age resulting, for example, in a 5^th^ and 95^th^ percentiles of 14–16% for ages 40–44 years and 17–21% for ages 70–74 years in men. Some countries, especially the islands of the Western Pacific Region, had higher thresholds to achieve net benefit of statins at 25–36% 10-year CVD risks.

**Conclusions:**

This extensive benefit-harm analysis modeling shows that a single CVD risk threshold, irrespective of age, sex and country, is not appropriate to initiate statin use globally. Instead, countries need to carefully determine thresholds, considering the national or subnational contexts, to optimize benefits of statins while minimizing related harms and economic burden.

## Introduction

Most current guidelines recommend a single threshold for initiating statins for primary prevention of cardiovascular disease (CVD) [1–3]. Irrespective of age, sex, or other factors that influence the benefit-harm balance of statins, common thresholds are 7.5% or 10% 10-year risks for CVD, with or without consideration of additional risk factors [1–3]. Many regions, especially the low- and middle-income countries (LMICs), usually simply adopt recommendations from the authoritative guidelines [4], such as from the US or Europe, without a formal adaptation process [5]. The World Health Organization (WHO) recommends 10-year risk thresholds of 20% in high-income, 30% in medium-income, and 40% in low-income settings [6]; but this seems to rely entirely on resource availability and not on the clinical benefit-harm balance, which would suggest different thresholds. Recently, the introduction of generic statins would reduce the cost of statins [7]; yet even if costs are too high, subsidized access to medication programs in resource-limited settings for patients with high risk populations– as in the example of antiretroviral therapy for HIV–, can be devised if statins have relevant net benefit in reducing CVD burden.

The use of a single threshold globally to initiate statins is very likely to lead to an over- and underuse of statins in different target groups. We found in our recent quantitative benefit-harm balance modelling study for a few specific countries that age and sex had a large influence on appropriate risk thresholds since these factors are associated with differences in baseline incidences of the health outcomes, both of which impact have significant impact on the benefit-harm balance of statins [8]. Thus, even within a population or country, recommendations for statin use should take into account age- and sex of target subgroups or patients. In addition, the analyses in the same study performed for the populations in Switzerland, the UK, and the US showed somewhat different thresholds, as the outcome baseline risks differ between the countries [8]. Without considering such variations between different environments and countries, there is a great risk that a single threshold leads to an over- or underuse of statins. It would be important to take local population factors into account and inform and guide national policy makers to develop recommendations contextualized to national or even local settings. Against this expected significance, this study aimed to determine age- and sex-specific CVD risk thresholds for 186 countries, particularly focusing on baseline risk variation such as type 2 diabetes, hemorrhagic stroke, and competing risk for non-CVD death.

## Methods

### Study design and population

We modelled the quantitative benefit-harm balance using a model developed by Gail et. al. [9], which allows to convert multiple parameters simultaneously into a common scale that enable to judge whether the benefits outweigh the harms or vice versa (see Additional file: Figure S1 for analytical flow chart). The method is also further described in detail elsewhere [8, 10]. The study addressed statins for primary prevention of CVD for persons in the general population older than 40 and younger than 75 years with no history of CVD events (but with a certain predisposing risk) across 186 countries. People aged 75 years or older were excluded from this study due to limited data and treated in a separate study [11]. We focused on low- or medium-dose statins, which are commonly used for primary prevention [12]. While benefit-harm profiles of specific statins differs–as previously shown –[8] we focused in this study on statins as a class because we mainly intended to assess the extent of threshold variation between populations. That is, the aim here was to trigger an initiative to consider contextualized recommendations for or against statins as the function of differences in outcome risks, and not due to differences between statins.

### Selection of evidence: health outcomes, statin treatment effects, baseline risks, and preferences

Several parameters were considered in the benefit-harm balance modeling. The most important were statin treatment effect estimates, baseline incidence of health outcomes, patient preferences for outcomes, competing risk for non-CVD deaths. The benefit health outcome of statins considered was CVD (fatal and non-fatal events combined), which is commonly used as endpoint in most guidelines and risk scores [1–3]. The adverse effects (hereinafter referred to as harms) of statins were myopathy, hepatic and renal dysfunctions, cataracts, hemorrhagic stroke, type 2 diabetes, all cancers, and nausea or headache, and treatment discontinuation [12]. While stroke is generally considered a CVD event, in this study specifically hemorrhagic stroke was considered a harm effect as some evidence suggests [12], but ischemic stroke was constitutive for the benefit outcome of CVD. We considered these predefined outcomes based on a prior selection of outcomes and preference-eliciting study [13]. We considered the outcomes despite the statistical significance of the statin treatment effects, since the absolute effect should be determined by the baseline incidence of the outcomes in the general population. In the analysis, we also considered non-CVD deaths as a competing risk–i.e., when people die of non-CVD death they should be deduced from the population at risk for having CVD events or the harm outcomes. We used a systematic approach to identify and select the most applicable, precise, and valid estimates for treatment effects, baseline risks, and preferences of the benefit and harm outcomes, as described below [14, 15]. Of note, this study focused on the clinical benefit-harm balance of statins and did not account for other factors such as the cost of statins and associated expenses. However, countries could further adjust thresholds depending on the resource availability as well as the proportion of populations at high risk for CVD, health system and health infrastructure, affordability, etc.

We took treatment effects from our previous network meta-analysis of statins from randomized controlled trials (RCTs) in primary prevention population [12]. However, comparative observational studies are also potential data sources for providing relevant information on long-term effects, particularly harm risks despite some inherited limitations [16, 17]. We consolidated effects of statins specifically for some harm outcomes from both RCTs and observational studies using inverse variance weighted averaging [18–20]. We used estimates that were likely to be valid and applicable and that did not represent extremes [21]. Nonetheless, we also performed sensitivity analyses taking estimates from RCTs only.

We previously elicited outcome preferences from potential statin users using a best-worst scaling survey in Switzerland and Ethiopia to inform this study [13]. Because people may have different values and preferences for treatment outcomes and goals, it is important to consider patient preferences rather than clinician perspectives when issuing recommendations. For example, to some persons the risks associated with statins are perceived acceptable, whereas to other risk-averse persons statin therapy is harmful. Hence, we considered preferences from the survey that were estimated on a scale over 0 to 1 (a higher value means that people have a stronger preference to avoid the disease) [22]. The preferences were very similar between both countries, and thus we took the average estimates to apply across countries [13]. Indeed, the Global Burden of Disease (GBD) study on different health outcomes also found similar disability weights in different countries that corroborated our assumption [23].

Age- and sex-specific baseline risks for diabetes, cancer, hemorrhagic stroke, and non-CVD death were extracted from the 2016 GBD estimates for the different countries (http://ghdx.healthdata.org/gbd-results-tool) and rates of cataracts, myopathy, and hepatic and renal dysfunctions from other observational data (age- or country-specific rates were not available for these harms) [18, 24]. The GBD estimates may have limitations but these are the most replicable and comparable data available for baseline risks. Nevertheless, our method is open to updating when further national or sub-national data are available to estimate more accurate thresholds that apply to the specific populations. The input estimates for treatment effect, preference, and outcome risks are presented in Additional file, Tables S1 and S2.

We considered a wide, hypothetical CVD risk spectrum (1 to 40% 10-year risk score) to determine the threshold at which statins provide net benefit. For this analysis it was not necessary to know which CVD risk score to use a priori. Of course, to apply the CVD risk thresholds from this study in practice, physicians need to use an appropriate CVD prediction model for their country and setting in order to determine the risk in an individual and to recommend statins if this risk falls above the risk thresholds determined in this study. However, it should be noted that for the application of the thresholds, risk score for the combined CVD endpoint (fatal and non-fatal events) must be used, not for fatal CVD only, such as the SCORE [25].

### Time horizon

We considered a 10-year horizon over which we predicted the benefit-harm balance, which is a commonly used time horizon in most guidelines for primary prevention. Other assumptions about time horizons and temporal effects (e.g., a yearly decline in the effect of statins on harm outcomes over time) were reported in our earlier study [8]; and, we did not duplicate such analyses here.

### Model structure

We made some adjustment to the Gail et. al. [9] model to meet our aim for determining risk thresholds (Additional file: Figure S2) [8]. The model was designed to predict benefit and harm events in cohorts of apparently healthy people (i.e., with no history of CVD events) aged 40 years to 75 years taking statins or not for 10 years. The cohorts were assumed to have certain predisposing risks for CVD; hence, we stratified a hypothetical 10-year CVD risk spectrum, from 1 to 40% until statins showed net benefit. We stratified the analyses by age and sex, and country.

### Analysis

Using the described model structure, we calculated the expected prevented CVD risks and excess risks of each harm outcome in 10,000 people over 10 years treated with statins vs. not treated to obtain absolute expected benefit and harm risk differences. We calculated this for sex, age (7 age groups), and 186 countries for a range of 10-year CVD risks from 1 to 40%, using an exponential model. The detailed statistical model was published previously and further details included in the Additional file, method S1 [8]. For each subgroup, we then weighted the absolute events of the outcomes by their respective preference values. We summed the weighted risk differences across all outcomes to yield the benefit-harm balance index in a common scale. We simulated the analysis 100,000 times taking into account the statistical uncertainty of each input parameter to generate a distribution of the index.

From the distribution of the benefit-harm balance index, we calculated the probability that the index was positive (more benefit than harms) across the 1% rise of the 10-year CVD risks from 1 to 40%. We defined net benefit at the probability of 0.6. This means that in 60% of the 100,000 repetitions, taking statins showed net benefit compared to not taking statins. The CVD risk level (across the 1 to 40% risk spectrum) above which the probability of net benefit exceeded 0.6 was the risk threshold for initiating statin use for primary prevention of CVD. The reason for defining the probability of net benefit at 0.6, and not at 0.5 as is often done, was because we aimed to ensure some clinical relevance at the determined thresholds. With a 0.5 probability for the index being positive, the index is zero, on average; or, benefits of statins equal harms. However, the 0.6 probability ensures certain absolute net benefit; yet, the clinical relevance of the net benefit can further be evaluated. Conversely, we defined net harm, or harms outweighed benefits, if the probability for the index being positive was less than 0.4. Probabilities between 0.4 and 0.6 represented neither net benefit nor net harm (see Additional file: Figure S3 for additional illustration).

Moreover, while it is important to estimate the clinical relevance of the thresholds, the benefit-harm balance index is difficult to interpret because it is a composite metric resulting from multiple interaction of parameters. As it stands, the index can be interpreted as number of prevented fatal CVD or a poor prognosis outcome with a maximum preference value of 1. To estimate how many absolute events could be prevented at the calculated risk thresholds, we preferred to interpret the index in terms of myocardial infarction (MI) events – a more common, single, non-fatal outcome that would be more important for clinical decision making– by dividing the index by the preference value of the MI.

### Sensitivity analysis

We conducted a sensitivity analysis to assess the impact of including observational data. We performed the analysis, relying solely on treatment effects on harm outcomes based on RCTs. Results of other sensitivity analyses were published previously, including a possible decrease in treatment effects of statins over time, possible correlation between outcomes (e.g., between CVD and diabetes, or between diabetes and renal dysfunction), as well as different preference weights [8].

## Results

### 10-year CVD risk thresholds for net benefit of statins

**Fig. 1:**
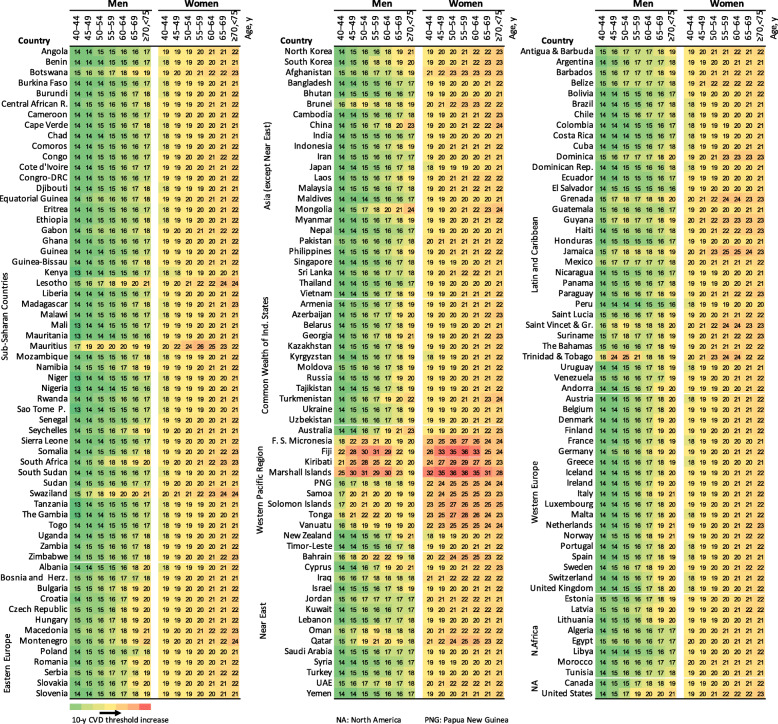
Country-specific 10-year CVD risk thresholds in men and women and different age groups (countries grouped by region)

**Fig. 2:**
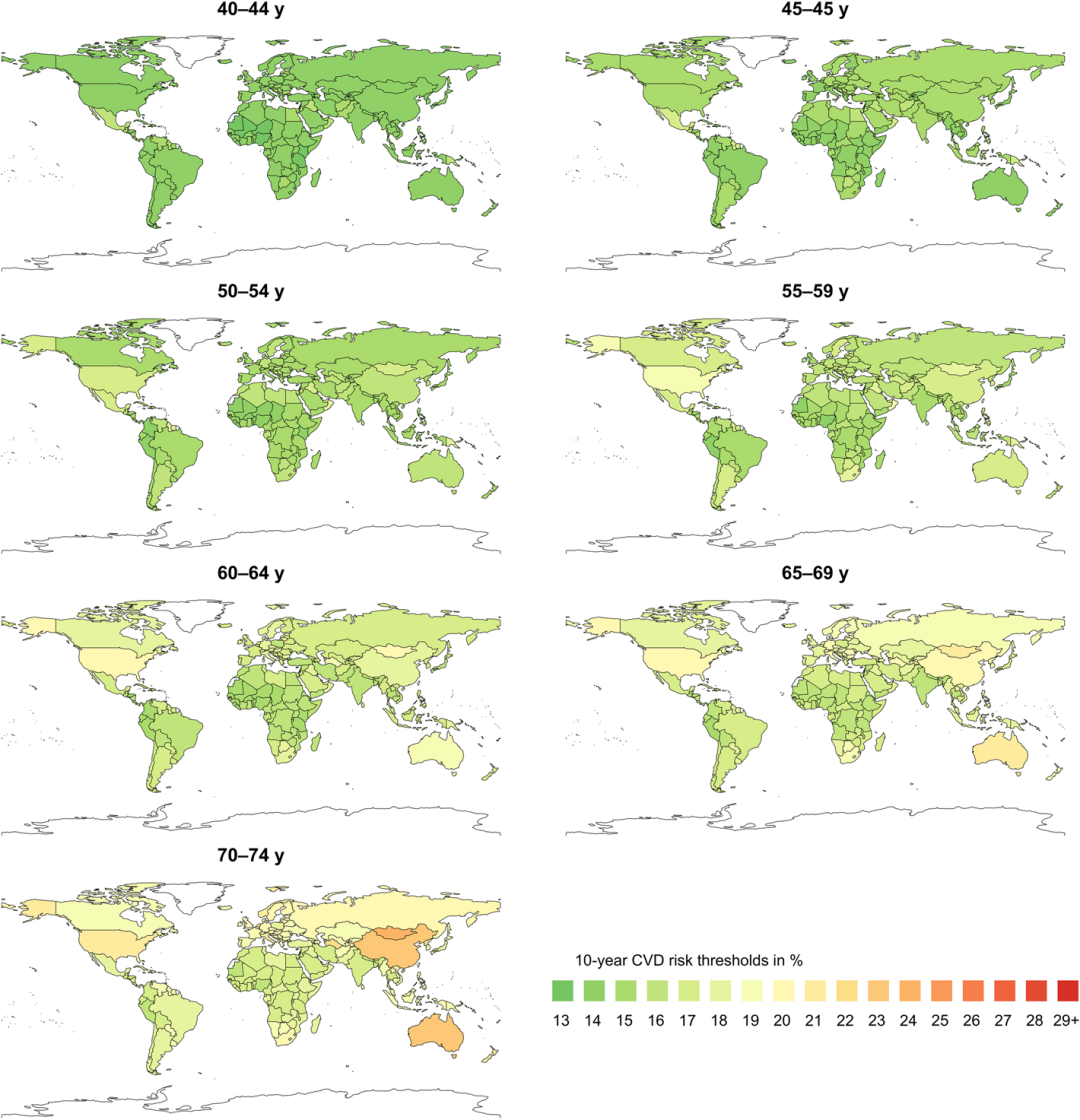
Map of 10-year CVD risk thresholds in men of different age groups across countries

**Fig. 3:**
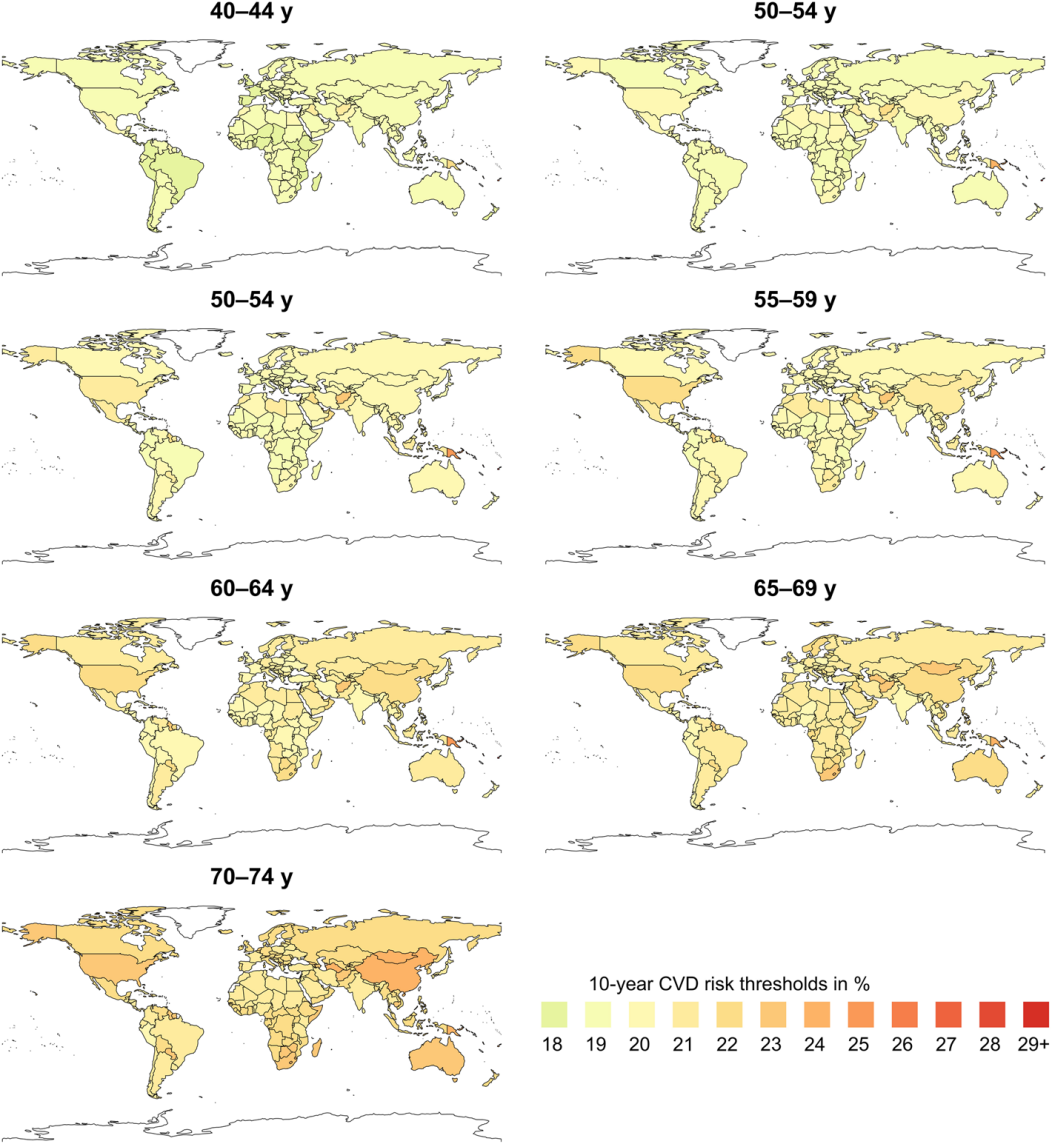
Map of 10-year CVD risk thresholds in women of different age groups across countries

Figure 1 shows the specific thresholds for each country where statins were more likely to provide a net benefit with a probability of 0.6 (see Additional file: Figures S4 and S5 for further results). The 10-year CVD risk thresholds ranged between 13 and 31% in men and 19–36% in women of all ages and countries. The 5^th^ and 95^th^ percentiles of the 10-year CVD risk-thresholds of the countries ranged from 14 to 20% in men and from 19 to 24% in women, with median varying from 14 to 18.5% in men and 19–22% in women, depending on age from younger (40–44 years) to older people (70–74 years). After adjustment for age, the between-country variation of the 10-year CVD risks was slightly reduced– but still considerable– with 5^th^ and 95^th^ percentiles of 14–16% for ages 40–44 years and 17–21% for ages 70–74 years in men as well as 18–21% and 20–24% in women for the respective age groups (see detailed subgroup estimates in the Additional file: Table S3). The thresholds are visually illustrated in Figs. 2 and 3 across countries globally.

While the majority of thresholds were below 21%, some countries, such as Bahrain, Jamaica, Qatar, Trinidad and Tobago, Brunei, and Mongolia among others, had thresholds higher than 21% particularly for older people. Most of the island countries of the Western Pacific region, including Marshall Islands, Kiribati, Fiji, Federated States of Micronesia, Tonga, and Solomon Islands had even higher thresholds of 25–36%. On the other hand, although not materially different from many of the other countries, the smallest threshold was 13%, which occurred in most African countries. Generally, countries in Sub-Saharan Africa (except Lesotho, Mauritius, Swaziland, and Botswana), countries in South America (except Trinidad and Tobago, Jamaica, Grenada, Dominica, and Saint Vincent and the Grenadines), and Asia (except Mongolia, Brunei, China, and Turkmenistan for the older people) were more likely to benefit from statins at relatively lower risk thresholds related to lower baseline risks of most harm outcomes.

### Clinical relevance of the thresholds

The thresholds were determined ensuring that the benefits exceeded the harms at least to some extent (further illustrations, Additional file: Figure S6). The extent of net prevented MI events were similar across countries and between age groups at the established risk thresholds, with only marginal differences between men and women. The average prevented MI events at the determined thresholds ranged from 22 to 41 in 10,000 people treated with statins compared to untreated over 10 years. In other words, taking low- or moderate-dose statins daily for 10 years could prevent 22–41 MI events in 10,000 adult people prescribed at the determined age- and sex-specific baseline CVD thresholds.

### Sensitivity analysis

The exclusion of observational data (i.e., taking only RCT data) resulted in lower CVD risk thresholds for initiating statins than the base-case analysis where combined effects were considered. The 5^th^ and 95^th^ percentiles of the 10-year thresholds ranged from 11 to 16% (median 11–15%, depending on age) in men and 15–19.5% (median, 15–18%, depending on age) in women (Additional file: Figure S7 and Table S4).

## Discussion

We performed the first worldwide benefit-harm balance modelling study and found that 5^th^ and 95^th^ percentiles of the 10-year CVD risk-thresholds in 186 countries varied from 14 to 20% in men and from 19 to 24% in women and with median varying from 14 to 18.5% and 19–22% respectively, depending on age. While most countries had 10-year risk thresholds below 21%, some countries had higher thresholds. The differences in thresholds across populations should be interpreted with respect to baseline risks of harm outcomes and the competing risk varying across countries while we kept, according to current evidence, treatment effects and preference values similar. The small differences in thresholds between men and women were due to variation in baseline risks of the outcomes and effect estimates.

Our study showed variation in risk thresholds across countries and, as in our earlier study [8], differences according to age and sex. As reported previously, the higher thresholds in the older age groups were due to the fact that the excess harms increased with age, offsetting the benefits of statins. The small islands countries in the western pacific region had higher risk thresholds in our study. This was due to higher risk rates for diabetes, which would be further increased with the use of statins that counterbalance the possible benefit of statins in reducing CVD events. This imply that use of a one-size-fits-all threshold or recommendation for all populations would lead to gross over- or underuse of statins in different populations, depending on the distribution of the benefit and harm risks. The threshold variation could have even been higher if country-specific rates for all outcomes were available as well as other factors (e.g., absolute proportion of people above the calculated risk threshold) were considered. The findings suggest the need of contextualized thresholds that reflect national or subnational distributions of the harm risks and CVD risks, rather than importing recommendations from authoritative guidelines without proper adaptation to the local settings.

There are no well-documented approaches of how countries determine thresholds to initiate statins. Some countries adapted guidelines to their settings or developed their own, including Japan and Fiji (threshold ≥30% 10-year risk), but it is not clear what evidence was considered in the customization of the guidelines. Nonetheless, many countries globally, especially LMICs, do not have clear guidelines. Those that have some sort of guidelines, for example, Kenya, India, Fiji, South Africa, and Brazil, are not clear about how they established their thresholds, which seem to be adopted from guidelines developed for other populations or settings [26–29]. For example, the South African guideline was adapted from the European one (probably with some changes) and thus recommends statins for people with at least a 10-year CVD risk of 30% (3–15% risk with additional consideration of lipid target) [29]. However, our thresholds for this country were 14–23%, depending on age and sex. Similarly in Kenya where statins are recommended starting at a 20% 10-year risk, our findings showed lower thresholds, especially for men [26]. This implies that there could be missed opportunities of preventing CVD events with statins in these populations. On the contrary, an overuse of statins in some settings is also possible such as in China, Brazil (for women), the UK, and the US where a 10% or 7.5% risk is used to start therapy (e.g., NICE or USPSTF use 10% whereas ACC/AHA uses 7.5%), which is lower than ours [1, 2, 6, 30]. This generally suggest the need of systematic and quantitative approaches to establish recommendations based on the distribution of risks, health system, infrastructure and resources.

The idea of contextualizing recommendations to different populations may not be new. There are initiatives by WHO and World Heart Federation (WHF) to develop roadmaps to promote development of national policy and health systems, especially to support LMICs in detection, treatment, and management of CVD [6]. For example, the WHO/International Society of Hypertension (ISH) as well as the INTERHEART developed risk scores for different countries and sub-regions to detect people at high-risk [31, 32]. While absolute risk scores assist in risk stratification or detection of people at high-risk, they do not provide direct information on who should take statins, and how possible harm risks may influence the treatment outcome. Harms of statins need to be weighed against the benefit to determine even if a group of people have higher risk for CVD. Thereby, our country-specific thresholds could serve as a base-case–or insight– for guideline developers to extend the thresholds to more precise and tailor to country-specific circumstances in order to harness the benefits of statins for primary prevention and minimize related risks and costs. It shoulld therefore be noted that the thresholds might not be ready-to-use for treatment decisions, because it was difficult to find all detailed information in each country that may have significant impact on the precision.

Our thresholds show the lowest possible CVD risk levels across countries at which statins would be more likely to provide more benefits than harms. For example, the absolute prevented MI events in 10 years were 22–41 among hypothetical 10,000 people with a baseline CVD risk equivalent to the calculated thresholds. However, these events do not show the total prevented CVD events in the general population of the different countries, which national guidelines need to take into account. That is, the thresholds could be increased or decreased, thus can be updated to specific settings, depending on the distribution of CVD risk factors in populations, country’s target to reduce morbidity or deaths due to CVD with statin use, and resource availability. For example, lower (or similar) threshold in the Sub-Saharan Africa does not mean that statin therapy would lead to higher proportion of prevented CVD events in the region than high-income countries with higher thresholds. Such impact estimation is beyond this paper, as data on the distribution of CVD risk in the respective populations would be necessary. Our study was directed to assisting decision-making for clinical guideline developers. The findings were not intended to dictate clinicians when to prescribe statins by using only the calculated thresholds. In fact, the findings also convey important message for individual patient decision-making that clinicians need to evaluate the patient risks for the potential harm outcomes (besides risk for CVD) and involve patients to discuss the tradeoff between the benefits and harms of statins.

Our findings target the general primary prevention populations and their applicability to the diabetes population should be considered with caution. People may have different levels of glycemic impairment, although it may not reach the threshold for diabetes diagnosis. Thus, the use of statin could still raise glycaemia in diabetic patients, but the extent of the excessive effect is not clear in this population. In addition, it would be difficult to understand whether the excessive risk of glycaemia due to statins would be negligible if patients are under well-controlled antidiabetic treatment. On the other hand, it is clear that diabetic patients would be more likely to be eligible for statin treatment anyway (with a higher 10-year baseline risk) than non-diabetic patients, as diabetes is a strong predictor of CVD. In general, the application of the thresholds to diabetic patients might lead to under-treatment with statins, especially in patients with well-controlled blood glucose treatment. This can be left to the discretion of doctors and patients, to decide whether to initiate statins, taking into account the patients' glycemic control.

Our study is the first global benefit-harm balance modeling study that quantitatively considers important parameters required to determine thresholds that have practical relevance for patients and clinical decision-making. However, it comes with limitations such as that we obtained country-specific baseline risks only for part of the harm outcomes. We believe that this limitation of not having baseline incidences for some harm outcomes, including myopathy, renal and hepatic dysfunctions, and cataract, for each country may have led to an underestimation of the threshold variation between countries. Specific countries could update the thresholds according to harm outcome risks available for their specific context. The debate on what evidence to consider for harms of statins is still ongoing. The effect estimates on harms range from almost none to large effects, depending the sources but all data sources have their own strengths and limitations [12, 21, 33–36]. Hence, we considered convergent estimates, specifically for the harms, from both RCTs and observational data that were likely to be valid, have less bias, applicable and do not represent the extremes. We excluded observational data and post-market surveillance sources that reported extreme and contentious estimates [21]. Indeed, the RCTs contributed substantially more to the convergent estimates because of their higher precision; thus, the effect estimates used for harms were rather small. The estimates from observational studies were less precise likely due to adjustment for several covariates, which may have increased the standard error. In addition, the different outcomes considered in the analysis were defined a priori and thus considered regardless of their effect size or baseline rate. For example, hemorrhagic stroke and cancer were included in the model, but their influence on the results was not considerable due to the low rates or effect size of the outcomes.

In terms of patient preferences, we took average estimates from Switzerland and Ethiopia. While we elicited preferences of the different outcomes related to statins in socio-demographically and economically disparate countries, Ethiopia and Switzerland [13], there may be other factors that could have influenced the estimates that remain unaccounted for (e.g., cost). In fact, the GBD study also reported consistent disability weights across countries [23], which supports our assumption of taking the average estimates. However, it is worthwhile to test how the preferences are affected if economic factors are taken into account. Of note, our risk thresholds cannot be directly applied within the European Society of Cardiology and European Atherosclerosis Society guideline, because this clinical guideline addresses fatal CVD, while our risk thresholds considered fatal and non-fatal CVD events combined [25].

In summary, our worldwide map of 10-year CVD risk thresholds above which statins for primary prevention of CVD is likely to provide net benefit show high variation across populations due to differences in the baseline incidence of some harm outcomes and the competing risk for non-CVD death. The thresholds were also higher in the older people than in younger adults. The findings provide an insight for policy makers and guideline developers to consider contextual data to revise their guidelines on the use of statins, and adapt eligibility criteria for their specific country to avoid an over- and underuse of statins for primary prevention.

## Supplementary information

**Additional file 1: Appendix Figure S1.** Analytical flow chart for the estimation of CVD risk thresholds for initiating statins for primary prevention of CVD. **Appendix Figure S2.** Gail/NCI model structure. **Appendix methods S1.** Benefit-Harm Balance Modeling. **Appendix Figure S3.** Probability of net benefit across CVD risk spectrum for men aged 40–44 years, Switzerland. **Appendix Figure S4.** Mean probabilities (across countries) across 10-y CVD baseline risk among men and women and different age groups. **Appendix Figure S5.** Probability of net benefit across 10-year CVD baseline risk among men and women and different age groups for 186 countries. **Appendix Figure S6.** Net prevented non-fatal MI-equivalent events per 10,000 people in 10 years of taking statins. **Appendix Figure S7.** Country-specific 10-year CVD risk thresholds for net benefit in men and women and different age groups taking treatment effects from randomized controlled trials only (ie., excluding observational studies). **Appendix Table S1.** Summary of model input parameters. **Appendix Table S2.** Global burden of disease risk rates per 100,000 people yearly for selected outcomes across countries by age and sex, 2016. **Appendix Table S3.** Summary of the 10-y CVD risk thresholds by age and sex across countries: median (5th–95th percentiles) (base-case analysis). **Appendix Table S4.** Summary of the 10-y CVD risk thresholds (of Appendix Figure S5) by age and sex across countries from the sensitivity analysis taking treatment effects from randomized controlled trials only.

## Data Availability

The datasets supporting the conclusions of this article are included within the article and its additional file.

## Abbreviations

ACC/AHA: American College of Cardiology/American Heart Association
CVD: Cardiovascular disease
GBD: Global burden disease
HIV: Human immunodeficiency virus
ISH: International Society of Hypertension
LMIC: Low- and middle-income country
MI: Myocardial infarction
NICE: National Institute for health and care excellence
RCT: Randomized controlled trial
SCORE: Systematic coronary risk evaluation
UK: United Kingdom
US: United States
USPSTF: United States preventive service task force
WHO: World Health Organization
