# Supplementary material for: Global variation of risk thresholds for initiating statins for primary prevention of cardiovascular disease: a benefit-harm balance modelling study
Source: BMC Cardiovasc Disord. 2020 Sep 17;20:418. doi: 10.1186/s12872-020-01697-6 (PMC7495829; doi:10.1186/s12872-020-01697-6)
Supplement: Supplementary file 1 — Additional file 1: Appendix Figure S1. Analytical flow chart for the estimation of CVD risk thresholds for initiating statins for primary prevention of CVD. Appendix Figure S2. Gail/NCI model structure. Appendix methods S1. Benefit-Harm Balance Modeling. Appendix Figure S3. Probability of net benefit across CVD risk spectrum for men aged 40–44 years, Switzerland. Appendix Figure S4. Mean probabilities (across countries) across 10-y CVD baseline risk among men and women and different age groups. Appendix Figure S5. Probability of net benefit across 10-year CVD baseline risk among men and women and different age groups for 186 countries. Appendix Figure S6. Net prevented non-fatal MI-equivalent events per 10,000 people in 10 years of taking statins. Appendix Figure S7. Country-specific 10-year CVD risk thresholds for net benefit in men and women and different age groups taking treatment effects from randomized controlled trials only (ie., excluding observational studies). Appendix Table S1. Summary of model input parameters. Appendix Table S2. Global burden of disease risk rates per 100,000 people yearly for selected outcomes across countries by age and sex, 2016. Appendix Table S3. Summary of the 10-y CVD risk thresholds by age and sex across countries: median (5th–95th percentiles) (base-case analysis). Appendix Table S4. Summary of the 10-y CVD risk thresholds (of Appendix Figure S5) by age and sex across countries from the sensitivity analysis taking treatment effects from randomized controlled trials only. [file 12872_2020_1697_MOESM1_ESM.pdf]

Additional file

Global variation of risk thresholds for initiating statins for primary prevention of cardiovascular disease: a benefit-harm balance modelling study

Henock G. Yebyo, Sofia Zappacosta, Hélène E. Aschmann, Sarah R. Haile, Milo A. Puhan

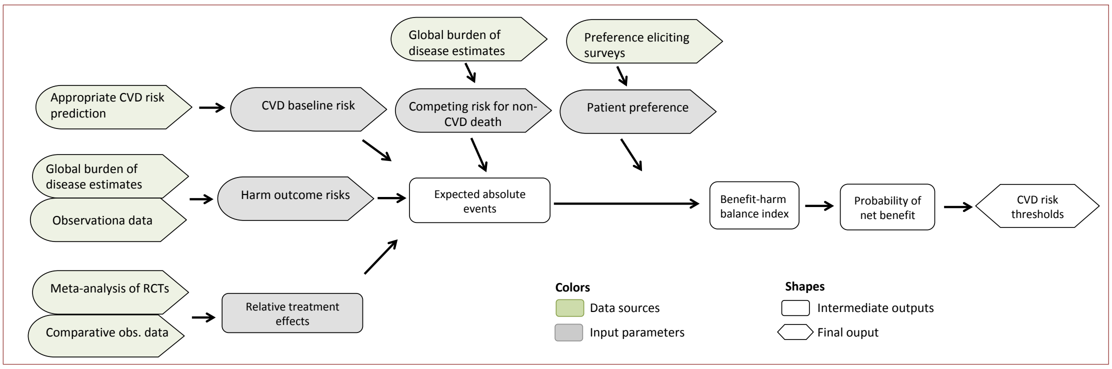

Appendix Figure S1. Analytical flow chart for the estimation of CVD risk thresholds for initiating statins for primary prevention of CVD

This outlines the general process of outcome estimation from data inputs to finalization of the benefit-harm balance analysis

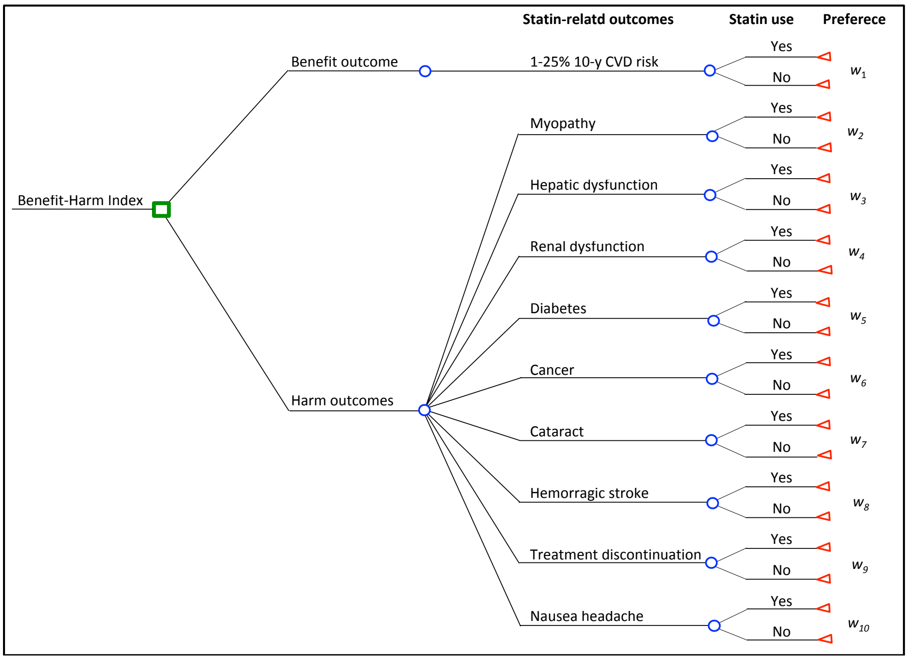

Appendix Figure S2: Gail/NCI model structure

Appendix methods S1. Benefit-Harm Balance Modeling

We used exponential model to estimate the expected risk of benefit and harm outcomes and further estimated the benefit-harm balance of statin use for primary prevention of CVD. While further details can be found in our previous study,[1] we briefly present the model and calculations of CVD risk thresholds as follows.

1. Expected risk of events ($N_{, no\ statin}$) over 10 years per 10,000 people having no history of CVD and without statin use was calculated using the following equation taking into consideration the baseline risk of benefit and harm outcome (I_i) and competing risk for non-CVD death (M_i).

$$N_{i,\ no\ statin} = 10000\{I_i/(I_i + M_i)\}[1 - exp\{-10\,yrs(I_i + M_i)\}]$$

2. The corresponding expected risk of events ($N_{, statin}$) per 10,000 people using statins for 10 years was calculated as below taking into consideration the treatment effect of statin use on each outcome (RR) in addition to the parameters in equation 1.

$$N_{i,\ statin} = 10000\{RR_i \times I_i/(RR_i \times I_i + M_i)\}[1 - exp\{-10\,yrs(RR_i \times I_i + M_i)\}]$$

3. The expected risk difference between people taking statins ($N_{, no\ statin}$) and not taking statins ($N_{, statin}$) would be the attributable net effect of statin use. However, in order to assess the metric for benefit-harm balance, the events should be transformed into similar scale of importance because the outcomes had different preferences or relative importance to patients. For example, say if statin use prevents 10 CVDs but has 40 excess myopathy risks, it would not be true to conclude that statins have net harm events (10-40= 30 excess myopathy events) because the outcomes are not similar in importance to patients. As a result, we weighted the events by preference values of each outcome to yield a commonly scaled index for the benefit-harm balance using:

$$Benefit - harm\ index = \sum_{i}^{10\ outcomes} \left(N_{i,\ no\ statins} - N_{i,\ statins}\right) \times W_i$$

4. We considered the statistical uncertainty of the input parameters taking their distributions and resampled them 100,000 times to estimate the benefit-harm balance index. We repeated this analysis for wide CVD risk spectrum (1% to 40%, depending on the country) and further estimated the probability at which of this CVD risk spectrum that statin use would more likely to provide net benefit (i.e., net benefit if the probability equaled to 0.6 or higher). This CVD risk level was defined as the "threshold" to recommend statins (see an example probability curve in appendix Figure 3 for Switzerland).

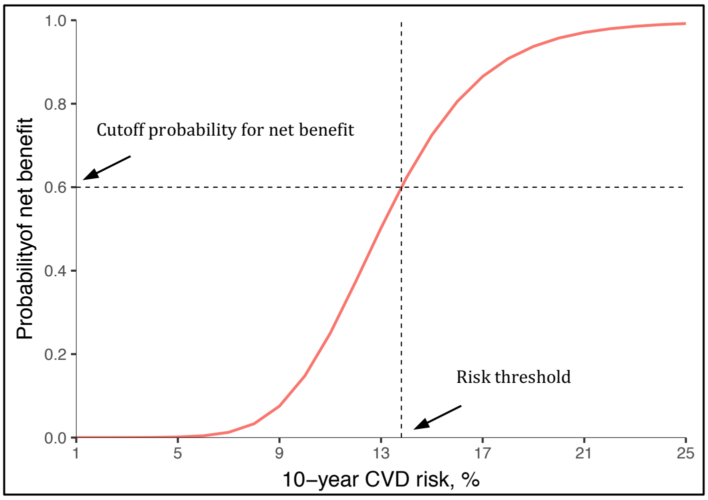

Appendix Figure S3. Probability of net benefit across CVD risk spectrum for men aged 40–44 years, Switzerland
The Probability of net benefit was estimated from the distribution of benefit-harm balance index across CVD risk spectrum, 1% to 25% 10-year risk. The threshold was defined at CVD risk level where the probability of net benefit reached 0.6.

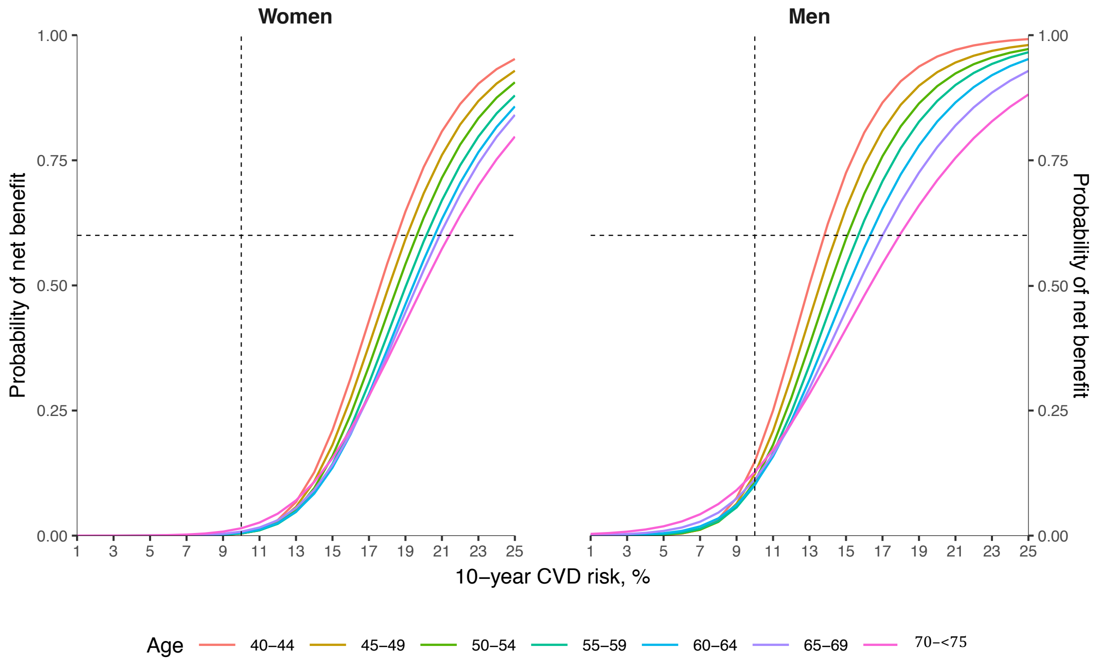

Appendix Figure S4. Mean probabilities (across countries) across 10-y CVD baseline risk among men and women and different age groups

The horizontal line is a reference value for the 0.6 probability of net benefit. The vertical line shows the 10% 10-year CVD risk that is commonly used by most clinical guidelines. The thresholds are the values in the x-axis corresponding to the points where the horizontal line at probability of 0.6 meets the age-specific curves.

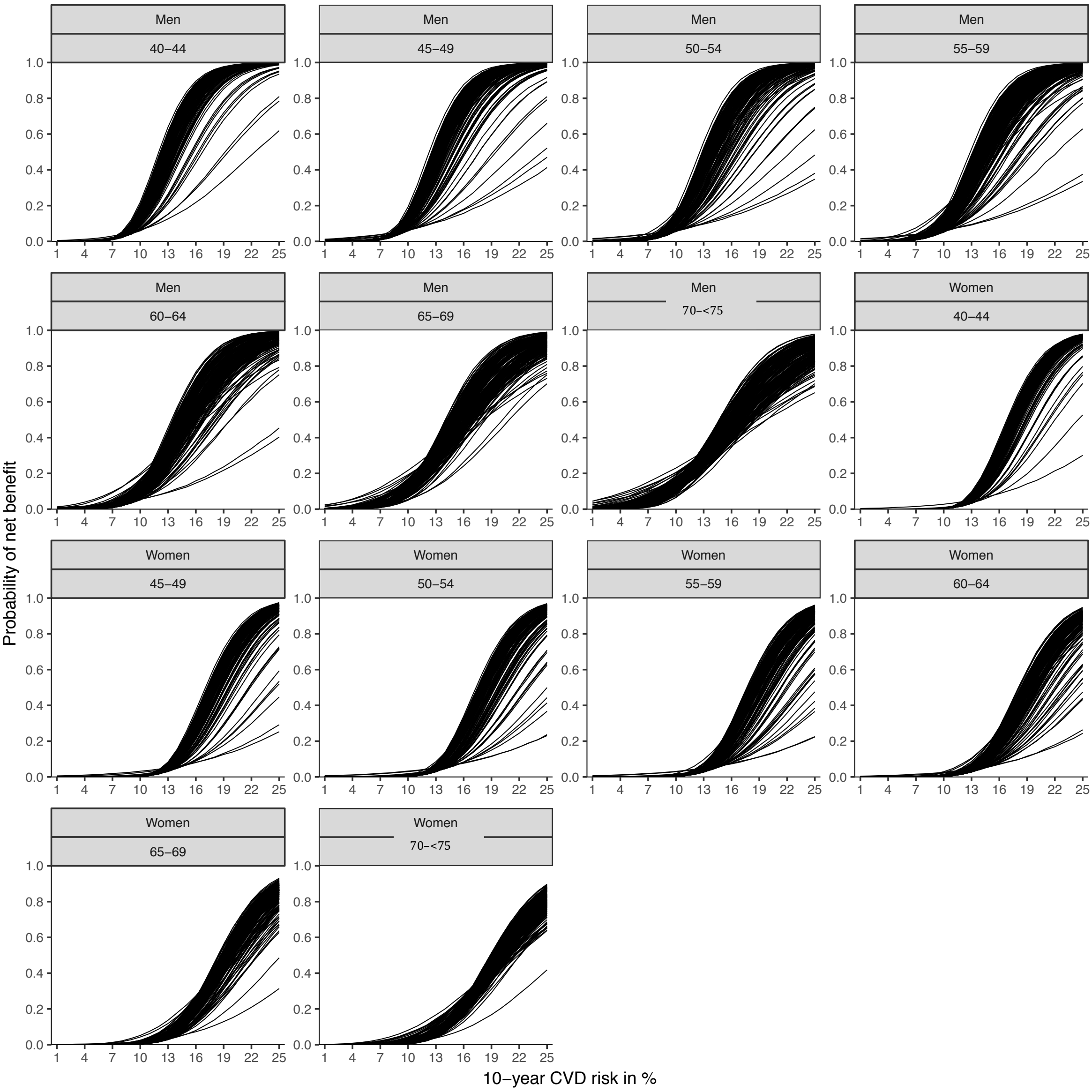

Appendix Figure S5: Probability of net benefit across 10-year CVD baseline risk among men and women and different age groups for 186 countries
The line represents countries

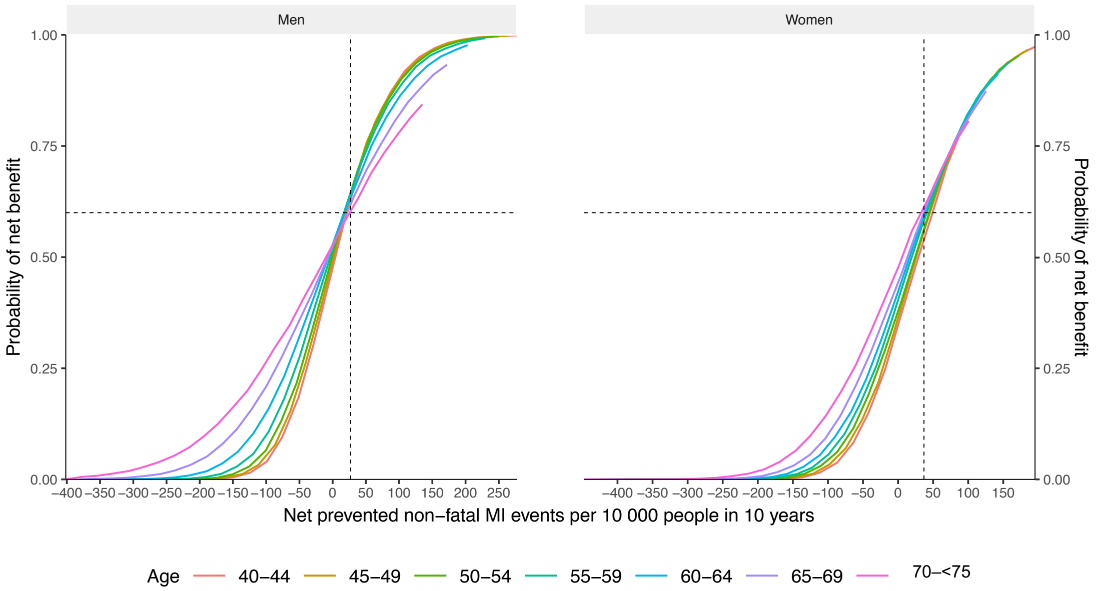

Appendix Figure S6: Net prevented non-fatal MI-equivalent events per 10 000 people in 10 years of taking statins

The distribution, by age and sex, of mean net prevented MI-equivalent events per 10 000 people treated with statins (versus 10 000 untreated people) at different level for probabilities of net benefit. The horizontal line is a reference value for the 0.6 probability of net benefit. The vertical line indicates the average net prevented MI-equivalent events at which the thresholds was defined.

	Men							Women						
Country	40-44	45-49	50-54	55-59	60-64	65-69	70-75	40-44	45-49	50-54	55-59	60-64	65-69	70-75
Afghanistan	11	12	13	13	14	14	15	16	17	18	18	18	18	19
Albania	11	11	12	12	13	15	17	15	15	15	16	16	17	19
Algeria	11	11	12	12	13	13	13	15	16	16	16	17	17	17
Andorra	11	11	11	12	13	14	16	15	15	16	16	16	17	17
Angola	11	11	11	12	12	13	14	15	15	16	16	17	17	18
ANT	11	12	13	13	13	14	15	15	16	16	17	17	17	18
Argentina	11	11	12	13	13	13	14	15	15	16	16	16	17	17
Armenia	11	12	12	13	13	14	15	15	16	16	16	17	17	18
Australia	11	11	12	13	14	15	17	15	15	16	16	17	17	18
Austria	11	11	12	12	13	14	15	15	15	16	16	16	17	18
Azerbaijan	11	12	12	13	14	15	16	15	16	16	17	17	18	19
Bahrain	12	13	14	16	16	14	14	16	17	18	19	19	18	17
Bangladesh	11	11	11	12	13	13	14	15	16	16	16	17	17	18
Barbados	11	12	13	13	14	14	15	16	16	17	17	18	18	18
Belarus	11	12	12	13	14	15	15	15	15	16	16	16	17	18
Belgium	11	11	11	12	13	14	15	15	15	16	16	16	17	17
Belize	11	12	13	13	13	13	14	15	16	17	17	18	17	18
Benin	11	11	11	12	12	13	14	15	15	16	16	16	17	18
Bhutan	11	11	11	12	12	13	13	15	16	16	16	16	16	17
Bolivia	11	11	12	12	12	13	13	15	16	16	16	17	17	18
BIH	11	12	12	12	13	14	15	15	15	16	16	17	17	17
Botswana	11	12	12	13	14	14	15	15	16	16	16	17	18	18
Brazil	11	11	11	12	12	13	14	15	15	16	16	16	17	17
Brunei	12	13	14	14	14	14	15	16	16	17	18	18	18	18
Bulgaria	11	12	12	13	14	15	16	15	16	16	16	17	17	18
Burkina Faso	11	11	11	12	12	13	14	15	15	16	16	16	17	17
Burundi	11	11	11	12	12	13	14	15	15	16	16	16	17	18
Cambodia	11	11	12	12	13	14	15	15	16	16	17	17	18	19
Cameroon	11	11	12	12	12	13	14	15	15	16	16	16	17	18
Canada	11	11	12	13	13	14	15	15	15	16	16	16	17	17
Cape Verde	11	11	12	12	12	13	15	15	15	16	16	16	17	17
CAR	11	11	12	12	13	14	15	15	15	16	16	17	18	19
Chad	11	11	11	12	12	13	14	15	15	16	16	16	17	18
Chile	11	11	12	12	13	13	14	15	15	16	16	16	17	17
China	11	12	13	14	15	17	19	15	16	17	17	18	19	20
Colombia	11	11	11	12	12	13	14	15	15	15	16	16	16	17
Comoros	11	11	11	12	12	13	14	15	15	15	16	16	17	18
Congo	11	11	12	12	12	13	14	15	16	16	16	17	18	18
Costa Rica	11	11	11	11	12	12	13	15	15	15	16	16	16	17
Cote d'Ivoire	11	11	12	12	12	13	14	15	16	16	16	17	17	18
Croatia	11	11	12	13	13	14	16	15	15	16	16	16	17	18
Cuba	11	11	12	12	12	13	14	15	15	16	16	16	17	17
Cyprus	11	11	12	13	14	15	16	15	15	16	17	17	17	18
Czech Republic	11	12	12	13	13	14	16	15	16	16	16	17	17	18
DRC	11	11	12	12	12	13	14	15	15	16	16	17	17	18
Denmark	11	11	12	12	13	14	16	15	15	16	16	16	17	18
Djibouti	11	11	12	12	12	13	14	15	15	15	16	16	17	17
Dominica	11	12	13	13	13	14	16	15	16	17	17	18	18	18
Dominican Rep.	11	11	12	12	12	13	14	15	15	16	16	16	17	17
Ecuador	11	11	11	12	12	12	13	15	15	16	16	16	16	17
Egypt	11	12	12	12	13	13	14	16	16	16	16	16	17	17
El Salvador	11	11	12	12	12	12	13	15	15	16	16	16	16	17
Equatorial Guinea	11	12	12	12	12	13	14	15	15	16	16	17	17	18
Eritrea	11	11	11	12	12	13	14	15	15	16	16	16	17	18
Estonia	11	11	12	12	13	14	15	15	15	16	16	16	17	17
Ethiopia	11	11	11	12	12	13	14	15	15	15	16	16	17	18
F. S. Micronesia	13	15	16	16	15	15	16	17	19	20	21	20	19	20
Fiji	15	20	20	21	21	17	15	20	21	22	23	21	21	19
Finland	11	11	12	12	13	14	16	15	15	16	16	17	17	18
France	11	11	11	12	13	14	15	15	15	15	16	16	16	17
Gabon	11	12	12	12	13	13	14	15	16	16	16	17	17	18
Georgia	11	12	13	14	14	15	17	15	15	16	17	17	18	19
Germany	11	11	12	12	13	14	15	15	15	16	16	17	17	17
Ghana	11	11	12	12	12	13	13	15	16	16	16	17	17	18
Greece	11	11	11	12	13	14	15	15	15	15	16	16	17	18
Grenada	12	13	13	13	14	14	16	16	16	17	18	19	18	19
Guatemala	11	12	12	12	12	12	13	15	16	16	16	16	17	17
Guinea	11	11	11	12	12	13	14	15	15	16	16	16	17	18
Guinea-Bissau	11	11	12	12	13	13	15	15	16	16	16	17	17	18
Guyana	12	13	13	13	14	14	16	16	17	17	18	18	18	19
Haiti	11	12	12	12	13	13	14	16	16	17	17	18	18	19
Honduras	11	11	12	12	12	13	13	15	15	16	16	16	16	17
Hungary	11	12	12	13	14	14	15	15	16	16	16	17	17	17
Iceland	11	11	11	12	13	14	15	15	15	15	16	16	17	18
India	11	11	12	12	12	13	13	15	15	16	16	16	16	17
Indonesia	11	11	12	13	13	14	15	15	16	16	17	17	17	18
Iran	11	11	12	12	12	13	13	15	16	16	16	16	16	17
Iraq	12	12	13	13	14	14	15	16	17	17	17	18	18	18
Ireland	11	11	12	12	13	14	15	15	15	16	16	16	17	18
Israel	11	11	12	12	13	14	15	15	15	16	16	17	17	17
Italy	11	11	12	12	13	14	16	15	15	16	16	16	17	18
Jamaica	12	13	13	14	14	14	15	16	16	18	19	20	19	19
Japan	11	11	11	12	13	13	14	15	15	15	16	16	16	17
Jordan	11	12	12	13	13	13	13	16	16	17	17	17	17	17
Kazakhstan	11	12	12	13	14	14	16	15	15	16	16	17	17	18
Kenya	11	11	11	12	12	13	13	15	15	15	15	16	16	17
Kiribati	15	19	20	18	17	16	17	18	21	22	21	20	20	19
Kuwait	11	11	12	12	13	13	13	15	16	16	17	17	17	17
Kyrgyzstan	11	11	12	13	14	15	16	15	15	16	16	17	18	18
Laos	11	11	12	13	13	14	15	16	16	17	17	17	18	19
Latvia	11	12	12	13	14	15	16	15	16	16	16	17	17	18
Lebanon	11	11	12	12	12	13	13	15	16	16	16	16	17	17
Lesotho	11	12	13	14	14	15	16	15	16	16	17	18	19	20
Liberia	11	11	11	12	12	13	14	15	15	16	16	17	17	18
Libya	11	12	12	12	13	13	14	15	16	16	16	17	17	17
Lithuania	11	12	12	13	14	15	16	15	15	16	16	16	17	17
Luxembourg	11	11	12	12	13	14	15	15	15	16	16	17	17	18
Macedonia	11	12	13	13	14	15	17	15	16	16	17	17	18	19
Madagascar	11	11	12	12	13	14	15	15	15	16	16	17	18	19
Malawi	11	11	11	12	12	13	14	15	15	15	16	16	17	18
Malaysia	11	11	12	12	13	14	14	15	16	16	17	17	17	18
Maldives	11	11	11	12	12	13	14	15	15	16	16	16	17	17
Mali	11	11	11	11	12	13	13	15	15	16	16	16	17	18
Malta	11	11	12	12	13	14	16	15	15	16	16	16	17	18
Marshall Islands	17	21	22	22	21	17	16	26	26	28	29	25	25	25
Mauritania	11	11	11	11	12	12	13	15	15	16	16	16	17	17
Mauritius	13	14	14	15	15	14	15	16	17	18	20	20	18	18
Mexico	12	13	13	13	13	13	14	16	16	16	17	17	17	17
Moldova	11	12	12	13	14	15	16	15	15	16	16	17	18	18
Mongolia	11	12	14	15	16	18	20	15	16	17	17	18	19	21
Montenegro	11	12	12	13	14	16	18	15	16	16	17	17	18	20
Morocco	11	11	12	12	13	13	14	16	16	16	17	17	17	17
Mozambique	11	11	12	12	13	13	14	15	15	15	16	16	17	18
Myanmar	11	11	12	13	14	14	16	15	16	16	16	17	17	18
Namibia	11	11	12	12	13	14	15	15	15	16	16	16	17	18
Nepal	11	11	11	12	12	13	13	15	16	16	16	16	17	17
Netherlands	11	11	12	12	13	14	16	15	15	16	16	17	17	18
New Zealand	11	11	11	12	13	14	16	15	15	16	16	16	17	18
Nicaragua	11	11	12	12	12	13	13	15	15	16	16	16	16	17
Niger	11	11	11	12	12	13	13	15	15	16	16	16	17	18
Nigeria	10	11	11	11	12	12	13	15	15	15	15	16	16	17
North Korea	11	12	12	13	14	15	18	15	16	16	17	18	18	20
Norway	11	11	12	12	13	15	16	15	15	16	16	17	17	18
Oman	12	13	14	14	14	14	15	16	17	17	17	17	17	18
Pakistan	11	12	12	12	13	14	14	16	16	17	17	17	17	18
Panama	11	11	12	12	12	13	14	15	15	16	16	16	17	17
Papua New Guinea	12	13	13	14	14	14	16	17	18	19	20	19	19	20
Paraguay	11	12	12	13	13	14	15	15	16	17	17	17	18	19
Peru	11	11	11	11	12	12	13	15	15	15	16	16	16	17
Philippines	11	12	12	13	14	14	15	15	16	16	17	17	18	18
Poland	11	11	12	12	13	13	14	15	15	15	16	16	16	17
Portugal	11	11	12	12	13	14	15	15	15	15	16	16	17	17
Qatar	12	13	14	15	15	14	14	16	17	18	19	19	18	17
Romania	11	12	12	13	14	15	17	15	15	16	16	17	17	18
Russia	11	12	12	13	14	15	16	15	16	16	16	17	17	18
Rwanda	11	11	11	12	12	13	14	15	15	15	16	16	17	17
Saint Lucia	11	12	12	13	13	14	15	15	16	16	17	17	17	18
SVG	12	13	14	13	14	14	15	16	16	17	18	18	18	18
Samoa	13	14	15	15	15	15	15	17	18	19	20	19	19	19
STP	11	11	11	12	12	13	14	15	15	15	16	16	17	18
Saudi Arabia	11	11	12	12	13	13	14	15	16	16	16	16	17	17
Senegal	11	11	11	12	12	13	14	15	16	16	16	17	17	18
Serbia	11	12	12	13	13	14	15	15	16	16	16	17	17	18
Seychelles	11	11	12	12	13	14	15	15	16	16	17	17	17	17
Sierra Leone	11	11	11	12	12	13	14	15	15	16	16	17	17	18
Singapore	11	11	11	12	12	13	14	15	15	16	16	16	17	17
Slovakia	11	12	12	13	14	15	16	15	15	16	16	16	17	18
Slovenia	11	11	12	12	13	14	15	15	15	16	16	16	16	17
Solomon Islands	13	14	15	15	15	15	16	18	20	21	25	21	20	20
Somalia	11	11	11	12	12	13	14	15	15	16	16	17	18	19
South Africa	11	12	12	13	14	15	15	15	16	16	17	17	18	19
South Korea	11	12	12	13	13	14	15	15	15	16	16	16	17	18
South Sudan	11	11	11	12	12	13	14	15	15	16	16	17	17	18
Spain	11	11	12	12	13	14	15	15	15	15	16	16	16	17
Sri Lanka	11	11	12	13	13	13	14	15	16	16	17	17	17	17
Sudan	11	11	12	12	13	13	14	15	16	16	16	17	17	18
Suriname	12	13	13	13	14	14	16	16	16	17	17	17	18	19
Swaziland	12	13	13	14	15	15	16	16	16	17	17	18	19	19
Sweden	11	11	12	13	13	14	16	15	16	16	16	17	17	18
Switzerland	11	11	12	12	13	14	16	15	15	16	16	16	17	18
Syria	11	11	12	12	12	13	13	15	15	16	16	16	16	17
Tajikistan	11	11	12	13	13	14	16	15	15	16	16	17	18	19
Tanzania	11	11	11	12	12	13	14	15	15	15	16	16	17	18
Thailand	11	11	12	12	12	13	14	15	15	16	16	16	17	17
The Bahamas	11	12	12	12	13	14	15	15	16	16	17	17	17	18
The Gambia	11	11	11	12	12	13	13	15	15	16	16	16	17	17
Timor-Leste	11	11	12	12	13	13	14	15	16	16	16	17	17	18
Togo	11	11	11	12	12	13	14	15	15	16	16	16	17	18
Tonga	13	15	16	16	15	15	15	18	20	25	26	21	19	19
Trinidad and Tobago	13	17	17	15	14	14	15	16	17	17	18	18	18	18
Tunisia	11	11	12	12	13	13	14	15	16	16	16	16	17	17
Turkey	11	11	12	12	13	13	14	15	16	16	16	16	17	17
Turkmenistan	11	12	13	14	15	17	19	15	16	16	17	18	19	21
Uganda	11	11	12	12	13	13	14	15	15	15	16	16	17	18
Ukraine	11	12	12	13	13	14	15	15	15	16	16	16	17	18
UAE	12	12	13	13	13	13	14	16	16	17	17	17	17	17
United Kingdom	11	11	11	12	13	14	15	15	15	16	16	16	17	18
United States	11	12	12	14	15	15	16	15	16	16	17	17	17	18
Uruguay	11	11	12	12	13	14	15	15	15	16	16	16	17	17
Uzbekistan	11	12	12	13	14	14	15	15	16	16	16	17	17	18
Vanuatu	12	13	14	14	15	15	16	17	18	19	20	20	19	20
Venezuela	11	12	12	12	13	13	14	15	16	16	16	17	17	17
Vietnam	11	11	12	13	13	14	16	15	16	16	17	17	17	18
Yemen	11	11	12	12	13	13	14	15	16	16	16	17	17	18
Zambia	11	11	12	12	13	13	14	15	15	16	16	16	17	18
Zimbabwe	11	11	12	12	13	13	14	15	16	16	16	17	18	18

Appendix Figure S7: Country-specific 10-year CVD risk thresholds for net benefit in men and women and different age groups taking treatment effects from randomized controlled trials only (ie., excluding observational studies).

Appendix Table S1. Summary of model input parameters

	Treat effects consolidated from RCTs and observational data, RR (95% CI)		Baseline rates per 10,000 per year		Patient preferences
**Outcomes**	**Men**	**Women**	**Men**	**Women**	**SUCRA (95% CI)**
Myopathy	1.33 (1.25-1.41)	1.16 (1.09-1.23)	3.6 (3.4–3.9)	3.2 (2.9–3.5)	0.230 (0.228–0.238)
Renal dysfunction	1.31 (1.19-1.44)	1.26 (1.14-1.40)	7.4 (6.9–7.9)	6.0 (5.6–6.4)	0.236 (0.215–0.252)
Hemorrhagic stroke	1.17 (0.58-2.37)‡		See appendix Table 2 for country-specific rates		0.735 (0.671–0.802)
Hepatic dysfunction	1.41 (1.32-1.50)	1.40 (1.31-1.50)	0.00244	0.00246	0.431 (0.345–0.475)
Type 2 diabetes	1.09 (0.96-1.22)	1.09 (0.96-1.22)	See appendix Table 2 for country-specific rates		0.470 (0.452–0.501)
Any cancer	1.02 (0.95-1.10)	1.02 (0.95-1.10)	See appendix Table 2 for country-specific rates		0.846 (0.829–0.855)
Cataracts †	1.32 (1.27-1.37)	1.30 (1.25-1.34)	99 (98–100)	176 (174–178)	0.431 (0.345–0.475)
Nausea/headache §	1.12 (0.96-1.32)	1.12 (0.96-1.32)	409 (363–460)	409 (363–460)	0.060 (0.034–0.094)
Trt. discontinuation §	0.99 (0.83-1.21)	0.99 (0.83-1.21)	352 (327–379)	352 (327–379)	0.00
CVD§	0.74 (0.68-0.81)	0.74 (0.68-0.81)	NA	NA	0.664 (0.611–0.715)

RR= Risk ratio; Trt. discontinuation= Treatment discontinuation due to side effects; SUCRA= Surface Under the Cumulative Ranking Curve (the preference estimates were published previously);[2] Not Applicable; † The treatment effect was taken from the Heart Outcomes Prevention Evaluation (HOPE-3) trial only for cataract surgery;[3]‡ The relative treatment effect was taken from Management of Elevated Cholesterol in the Primary Prevention Group of Adult Japanese (MEGA) trial only and assumed to be similar for all subgroups due to unavailability of group-specific estimates;[4] § Treatment effect for CVD was considered only from the meta-analysis of randomized trials.

The relative effect estimates in Appendix Table 1 for harm outcomes were combined effects from RCTs and observational data sources published previously.[5,6] We consolidated the treatment effects from both sources using inverse-variance-weighted averaging equation (see equations below).[7] This method combines treatment estimates from two distributions (defined by mean treatment, *log RR* and variance, *var (log RR)*). The information of treatment effects are weighted by their inverse variance, 1/*var()*. We assumed that estimates from both sources were adequately approximated by normal distributions defined by the respective *log RR* and *var (log RR)*). Variance of the combined effect is the total variance of both sources.

$$\log RR_{mixed} = \frac{\frac{1}{var(\log RR_{RCTs})}\log RR_{RCTs} + \frac{1}{var(\log RR_{obs})}\log RR_{obs}}{\frac{1}{var(\log RR_{RCTs})} + \frac{1}{var(\log RR_{obs})}}$$

$$var(log\ RR_{mixed}) = \frac{1}{\frac{1}{var(\log RR_{RCTs})} + \frac{1}{var(\log RR_{obs})}}$$

log RR_{mixed}	*Log transformed combined effect estimate from RCTs, (log RR_{RCTs}) and observational data, (log RR_{obs})*
Var (log RR_{mixed})	*Variance of the combined effect was defined by the inverse of total variance of statin excess effect from RCTs, var (log RR_{RCTs}) and from observational data, var, (log RR_{obs}). Thus, confidence intervals were calculated using the log RR_{mixed} and Var (log RR_{mixed}).*

Appendix Table S2. Global burden of disease risk rates per 100,000 people yearly for selected outcomes across countries by age and sex, 2016

				Diabetes type 2	Cancers	Hemorrhagic stroke	All-cause mortality	CVD mortality
ID	Country	Sex	Age	Rate (95% CI)	Rate (95% CI)	Rate (95% CI)	Rate (95% CI)	Rate (95% CI)
1	Afghanistan	Female	40 to 44	117 (86-154)	17 (7-26)	4 (3-5)	91 (78-105)	29 (21-39)
2	Afghanistan	Female	45 to 49	157 (105-222)	23 (10-35)	7 (5-9)	124 (108-142)	49 (38-63)
3	Afghanistan	Female	50 to 54	179 (131-241)	28 (14-39)	10 (8-12)	179 (158-202)	83 (67-100)
4	Afghanistan	Female	55 to 59	184 (135-246)	34 (18-47)	12 (9-16)	254 (226-285)	125 (104-150)
5	Afghanistan	Female	60 to 64	161 (107-235)	37 (27-47)	17 (13-21)	358 (323-398)	174 (149-204)
6	Afghanistan	Female	65 to 69	110 (78-158)	46 (36-57)	24 (17-30)	540 (490-597)	274 (235-320)
7	Afghanistan	Female	70 to <75	76 (47-121)	58 (46-69)	33 (27-40)	833 (755-919)	436 (373-506)
8	Afghanistan	Male	40 to 44	79 (60-100)	7 (4-9)	4 (3-5)	104 (89-120)	32 (25-40)
9	Afghanistan	Male	45 to 49	98 (70-139)	11 (7-16)	7 (5-9)	142 (122-163)	56 (45-69)
10	Afghanistan	Male	50 to 54	108 (80-147)	18 (12-24)	10 (8-12)	198 (169-227)	91 (74-109)
11	Afghanistan	Male	55 to 59	109 (79-144)	25 (18-34)	14 (11-18)	273 (235-309)	132 (109-156)
12	Afghanistan	Male	60 to 64	101 (71-137)	34 (28-43)	19 (15-24)	392 (344-435)	201 (172-229)
13	Afghanistan	Male	65 to 69	85 (61-116)	47 (39-57)	25 (19-32)	552 (493-607)	288 (250-324)
14	Afghanistan	Male	70 to <75	68 (44-100)	68 (58-83)	35 (28-42)	808 (735-876)	405 (356-454)
15	Albania	Female	40 to 44	22 (17-28)	14 (11-17)	3 (2-4)	11 (9-12)	3 (2-3)
16	Albania	Female	45 to 49	21 (15-27)	20 (16-25)	5 (4-6)	16 (14-19)	5 (4-6)
17	Albania	Female	50 to 54	21 (16-29)	25 (21-30)	7 (6-9)	25 (21-30)	8 (7-10)
18	Albania	Female	55 to 59	24 (18-32)	29 (25-35)	10 (7-13)	38 (33-45)	15 (13-19)
19	Albania	Female	60 to 64	26 (19-34)	36 (31-42)	16 (12-19)	64 (55-76)	31 (26-38)
20	Albania	Female	65 to 69	26 (19-35)	43 (38-51)	24 (18-30)	109 (93-127)	62 (52-73)
21	Albania	Female	70 to <75	23 (16-32)	51 (43-61)	38 (32-44)	197 (166-233)	127 (106-150)
22	Albania	Male	40 to 44	29 (23-37)	9 (8-10)	5 (4-6)	22 (19-26)	8 (6-10)
23	Albania	Male	45 to 49	26 (19-35)	15 (13-17)	8 (6-10)	32 (27-38)	13 (10-16)
24	Albania	Male	50 to 54	26 (19-35)	25 (22-28)	11 (9-14)	50 (42-59)	21 (17-25)
25	Albania	Male	55 to 59	28 (21-37)	41 (35-48)	15 (12-21)	78 (65-93)	35 (28-42)
26	Albania	Male	60 to 64	28 (20-38)	66 (57-78)	23 (19-28)	133 (112-156)	63 (52-75)
27	Albania	Male	65 to 69	25 (18-35)	98 (84-117)	34 (25-43)	216 (181-257)	110 (90-133)
28	Albania	Male	70 to <75	21 (15-32)	130 (110-158)	53 (44-64)	360 (300-433)	202 (165-245)
29	Algeria	Female	40 to 44	75 (56-98)	12 (9-15)	2 (2-3)	15 (13-18)	4 (3-5)
30	Algeria	Female	45 to 49	95 (68-127)	16 (13-20)	3 (2-5)	24 (20-27)	8 (6-10)
31	Algeria	Female	50 to 54	106 (81-137)	19 (15-22)	5 (4-6)	36 (31-42)	13 (11-17)
32	Algeria	Female	55 to 59	108 (79-145)	21 (18-25)	6 (4-8)	56 (48-65)	23 (19-28)
33	Algeria	Female	60 to 64	97 (69-134)	25 (22-28)	9 (7-12)	94 (81-105)	43 (36-50)
34	Algeria	Female	65 to 69	74 (53-103)	27 (25-30)	13 (10-18)	145 (129-161)	70 (59-82)
35	Algeria	Female	70 to <75	57 (37-86)	31 (28-34)	18 (15-23)	235 (217-256)	118 (101-133)
36	Algeria	Male	40 to 44	68 (50-89)	4 (4-5)	2 (1-2)	20 (17-23)	5 (4-7)
37	Algeria	Male	45 to 49	87 (61-119)	7 (6-8)	3 (2-4)	28 (24-33)	10 (7-13)
38	Algeria	Male	50 to 54	100 (74-132)	11 (10-13)	5 (4-6)	44 (38-51)	17 (14-22)
39	Algeria	Male	55 to 59	106 (75-142)	16 (15-17)	8 (6-10)	66 (57-77)	28 (22-34)
40	Algeria	Male	60 to 64	102 (71-139)	25 (22-28)	11 (8-13)	110 (96-126)	49 (39-60)
41	Algeria	Male	65 to 69	87 (61-121)	34 (30-37)	14 (10-19)	169 (147-192)	76 (62-92)
42	Algeria	Male	70 to <75	70 (46-105)	43 (39-47)	19 (15-24)	273 (239-312)	124 (102-148)
43	Andorra	Female	40 to 44	21 (16-27)	32 (25-43)	2 (2-3)	7 (5-10)	1 (0-1)
44	Andorra	Female	45 to 49	22 (15-31)	46 (37-61)	4 (3-5)	12 (8-17)	1 (1-2)
45	Andorra	Female	50 to 54	27 (20-36)	59 (47-79)	5 (4-7)	18 (13-27)	2 (1-3)
46	Andorra	Female	55 to 59	38 (27-51)	73 (58-99)	6 (4-8)	26 (19-40)	3 (2-5)
47	Andorra	Female	60 to 64	42 (32-56)	91 (73-122)	7 (5-10)	39 (29-60)	6 (4-10)
48	Andorra	Female	65 to 69	42 (32-57)	109 (87-145)	10 (7-14)	61 (46-94)	11 (8-18)
49	Andorra	Female	70 to <75	37 (26-51)	129 (103-169)	16 (13-20)	104 (77-157)	24 (17-39)
50	Andorra	Male	40 to 44	28 (22-36)	18 (15-22)	3 (2-3)	15 (10-21)	3 (2-5)
51	Andorra	Male	45 to 49	34 (23-47)	30 (26-35)	4 (3-6)	24 (16-33)	6 (4-9)
52	Andorra	Male	50 to 54	42 (33-56)	53 (46-60)	6 (5-8)	38 (25-52)	11 (7-16)
53	Andorra	Male	55 to 59	54 (39-72)	89 (78-102)	8 (6-11)	61 (42-78)	18 (12-25)
54	Andorra	Male	60 to 64	59 (43-78)	145 (126-168)	12 (9-15)	95 (69-116)	28 (20-37)
55	Andorra	Male	65 to 69	56 (42-77)	216 (184-252)	17 (12-22)	147 (110-180)	45 (33-59)
56	Andorra	Male	70 to <75	47 (33-68)	289 (244-338)	25 (20-31)	241 (182-288)	79 (59-97)
57	Angola	Female	40 to 44	36 (27-47)	15 (11-19)	2 (2-3)	60 (44-85)	7 (4-11)
58	Angola	Female	45 to 49	43 (30-59)	20 (15-24)	4 (3-6)	79 (56-112)	14 (9-21)
59	Angola	Female	50 to 54	50 (37-66)	25 (20-30)	6 (5-8)	113 (79-156)	22 (14-33)
60	Angola	Female	55 to 59	57 (40-79)	31 (26-37)	9 (6-12)	159 (113-216)	39 (26-55)
61	Angola	Female	60 to 64	59 (42-82)	38 (33-43)	13 (10-16)	234 (173-304)	70 (50-95)
62	Angola	Female	65 to 69	57 (39-78)	46 (41-52)	19 (15-25)	358 (266-459)	128 (94-167)
63	Angola	Female	70 to <75	51 (34-73)	53 (47-60)	28 (23-34)	550 (427-712)	224 (166-297)
64	Angola	Male	40 to 44	39 (29-51)	5 (5-6)	2 (2-3)	69 (53-92)	7 (4-10)
65	Angola	Male	45 to 49	54 (39-74)	9 (8-11)	4 (3-5)	90 (67-121)	12 (8-18)
66	Angola	Male	50 to 54	62 (47-81)	16 (14-18)	6 (5-7)	119 (90-162)	20 (14-30)

				Diabetes type 2	Cancers	Hemorrhagic stroke	All-cause mortality	CVD mortality
ID	Country	Sex	Age	Rate (95% CI)	Rate (95% CI)	Rate (95% CI)	Rate (95% CI)	Rate (95% CI)
67	Angola	Male	55 to 59	61 (44-83)	25 (21-29)	8 (6-11)	161 (124-218)	34 (24-48)
68	Angola	Male	60 to 64	59 (41-81)	38 (32-44)	12 (10-15)	238 (187-316)	60 (44-82)
69	Angola	Male	65 to 69	55 (39-76)	55 (47-63)	18 (13-23)	351 (280-460)	97 (71-131)
70	Angola	Male	70 to <75	49 (33-70)	76 (63-90)	25 (20-30)	536 (432-691)	157 (118-210)
71	Antigua and Barbuda	Female	40 to 44	63 (46-82)	19 (17-22)	2 (2-3)	15 (12-19)	3 (2-4)
72	Antigua and Barbuda	Female	45 to 49	92 (63-129)	29 (25-33)	4 (3-5)	25 (20-30)	6 (5-8)
73	Antigua and Barbuda	Female	50 to 54	109 (81-142)	41 (35-47)	6 (5-7)	45 (37-53)	12 (9-15)
74	Antigua and Barbuda	Female	55 to 59	114 (84-159)	44 (40-50)	8 (6-11)	54 (45-64)	15 (12-19)
75	Antigua and Barbuda	Female	60 to 64	104 (71-145)	61 (55-68)	11 (9-14)	94 (78-110)	32 (26-39)
76	Antigua and Barbuda	Female	65 to 69	77 (54-109)	66 (60-73)	15 (11-19)	122 (101-143)	44 (36-54)
77	Antigua and Barbuda	Female	70 to <75	57 (35-88)	82 (75-90)	20 (17-25)	204 (170-241)	76 (63-92)
78	Antigua and Barbuda	Male	40 to 44	78 (59-100)	8 (7-10)	2 (2-3)	23 (19-28)	4 (3-5)
79	Antigua and Barbuda	Male	45 to 49	124 (89-168)	15 (13-18)	4 (3-5)	38 (31-46)	9 (7-12)
80	Antigua and Barbuda	Male	50 to 54	133 (101-173)	29 (26-34)	6 (5-8)	56 (47-67)	16 (13-21)
81	Antigua and Barbuda	Male	55 to 59	107 (79-137)	58 (51-66)	9 (7-12)	101 (85-119)	35 (28-42)
82	Antigua and Barbuda	Male	60 to 64	83 (52-118)	102 (88-118)	13 (11-17)	155 (133-179)	50 (41-60)
83	Antigua and Barbuda	Male	65 to 69	61 (41-89)	161 (135-194)	19 (14-23)	226 (194-260)	82 (69-97)
84	Antigua and Barbuda	Male	70 to <75	48 (31-75)	221 (183-268)	25 (20-29)	328 (282-376)	125 (106-148)
85	Argentina	Female	40 to 44	30 (21-40)	23 (21-26)	2 (2-3)	16 (14-17)	3 (2-3)
86	Argentina	Female	45 to 49	43 (26-62)	31 (28-34)	4 (3-5)	23 (21-26)	5 (4-6)
87	Argentina	Female	50 to 54	58 (44-75)	40 (36-45)	6 (4-7)	37 (33-41)	8 (6-9)
88	Argentina	Female	55 to 59	78 (57-104)	52 (48-58)	7 (5-9)	56 (50-62)	13 (11-15)
89	Argentina	Female	60 to 64	74 (54-104)	66 (61-72)	9 (7-11)	86 (78-95)	22 (19-26)
90	Argentina	Female	65 to 69	48 (35-66)	78 (72-85)	11 (8-14)	130 (117-143)	38 (32-44)
91	Argentina	Female	70 to <75	29 (18-46)	88 (82-95)	15 (12-18)	202 (181-223)	66 (57-76)
92	Argentina	Male	40 to 44	41 (31-52)	10 (9-10)	3 (2-3)	26 (23-29)	5 (4-6)
93	Argentina	Male	45 to 49	60 (42-83)	17 (16-18)	5 (3-6)	40 (35-44)	10 (9-12)
94	Argentina	Male	50 to 54	79 (61-101)	32 (30-35)	7 (5-8)	67 (60-75)	20 (17-23)
95	Argentina	Male	55 to 59	97 (70-126)	57 (53-61)	9 (7-12)	109 (98-121)	34 (29-39)
96	Argentina	Male	60 to 64	90 (64-121)	92 (85-100)	13 (10-16)	171 (155-188)	54 (47-62)
97	Argentina	Male	65 to 69	58 (44-76)	133 (122-145)	17 (13-21)	260 (236-285)	86 (75-98)
98	Argentina	Male	70 to <75	34 (21-55)	175 (161-194)	21 (18-26)	398 (362-436)	137 (121-155)
99	Armenia	Female	40 to 44	44 (34-55)	21 (18-25)	3 (2-4)	13 (11-15)	2 (2-3)
100	Armenia	Female	45 to 49	56 (38-81)	31 (27-37)	4 (3-6)	19 (16-23)	4 (3-5)
101	Armenia	Female	50 to 54	67 (50-87)	40 (35-47)	6 (5-8)	31 (27-37)	7 (6-9)
102	Armenia	Female	55 to 59	76 (56-102)	51 (44-60)	8 (6-11)	50 (43-60)	14 (11-17)
103	Armenia	Female	60 to 64	69 (44-98)	65 (57-76)	12 (9-15)	88 (75-103)	30 (24-36)
104	Armenia	Female	65 to 69	47 (34-65)	77 (68-87)	16 (12-21)	146 (125-170)	59 (49-70)
105	Armenia	Female	70 to <75	31 (18-49)	90 (80-102)	22 (18-27)	276 (236-318)	135 (113-157)
106	Armenia	Male	40 to 44	52 (41-64)	10 (9-12)	4 (4-6)	34 (28-40)	12 (9-15)
107	Armenia	Male	45 to 49	58 (38-80)	20 (17-24)	7 (5-10)	53 (45-62)	21 (17-25)
108	Armenia	Male	50 to 54	60 (40-81)	36 (32-43)	11 (9-13)	83 (71-97)	35 (29-42)
109	Armenia	Male	55 to 59	57 (39-77)	65 (57-74)	15 (11-19)	132 (113-151)	56 (47-66)
110	Armenia	Male	60 to 64	51 (32-72)	108 (96-122)	18 (15-23)	209 (182-237)	90 (76-103)
111	Armenia	Male	65 to 69	42 (29-63)	154 (134-180)	22 (17-28)	303 (266-340)	133 (116-150)
112	Armenia	Male	70 to <75	32 (20-51)	204 (175-241)	28 (23-34)	491 (435-545)	234 (204-265)
113	Australia	Female	40 to 44	20 (14-27)	41 (34-52)	2 (1-2)	9 (8-10)	1 (1-1)
114	Australia	Female	45 to 49	28 (19-39)	66 (51-90)	3 (2-4)	14 (12-15)	2 (1-2)
115	Australia	Female	50 to 54	37 (28-49)	89 (71-116)	3 (3-5)	21 (19-23)	3 (2-3)
116	Australia	Female	55 to 59	47 (33-64)	116 (90-156)	4 (3-6)	30 (28-33)	4 (3-5)
117	Australia	Female	60 to 64	52 (38-69)	151 (119-195)	6 (4-7)	47 (43-52)	7 (6-8)
118	Australia	Female	65 to 69	50 (36-69)	191 (156-230)	8 (6-10)	74 (67-81)	13 (11-15)
119	Australia	Female	70 to <75	42 (28-59)	231 (188-274)	13 (11-15)	126 (115-139)	28 (24-32)
120	Australia	Male	40 to 44	26 (20-34)	31 (24-44)	2 (1-2)	15 (14-17)	3 (2-3)
121	Australia	Male	45 to 49	39 (24-53)	56 (39-88)	2 (2-3)	22 (20-24)	5 (4-6)
122	Australia	Male	50 to 54	53 (40-67)	108 (81-145)	3 (3-4)	33 (30-36)	8 (7-9)
123	Australia	Male	55 to 59	71 (54-92)	191 (144-257)	4 (3-6)	50 (46-55)	12 (10-14)
124	Australia	Male	60 to 64	71 (54-93)	304 (233-385)	6 (5-8)	77 (71-85)	18 (16-21)
125	Australia	Male	65 to 69	55 (41-74)	441 (345-537)	9 (7-12)	124 (114-136)	30 (26-34)
126	Australia	Male	70 to <75	40 (26-59)	561 (438-684)	16 (13-19)	203 (187-221)	52 (46-59)
127	Austria	Female	40 to 44	19 (14-24)	24 (20-27)	3 (2-4)	8 (7-8)	1 (1-1)
128	Austria	Female	45 to 49	21 (14-30)	36 (31-41)	5 (4-7)	13 (12-15)	2 (1-2)
129	Austria	Female	50 to 54	31 (24-40)	50 (44-55)	7 (5-8)	22 (20-24)	3 (2-3)
130	Austria	Female	55 to 59	49 (34-67)	68 (61-76)	7 (5-10)	35 (32-38)	5 (4-6)
131	Austria	Female	60 to 64	56 (43-72)	94 (85-104)	9 (7-11)	57 (52-62)	10 (8-12)
132	Austria	Female	65 to 69	52 (38-71)	113 (102-124)	11 (9-14)	86 (79-94)	19 (16-22)
133	Austria	Female	70 to <75	42 (28-63)	127 (115-140)	17 (14-20)	132 (120-144)	38 (33-43)
134	Austria	Male	40 to 44	27 (20-35)	13 (12-16)	4 (3-5)	14 (12-15)	2 (2-3)
135	Austria	Male	45 to 49	35 (22-52)	25 (22-28)	6 (4-8)	24 (21-25)	5 (4-6)
136	Austria	Male	50 to 54	49 (38-64)	48 (43-54)	8 (6-10)	41 (37-44)	8 (7-10)

				Diabetes type 2	Cancers	Hemorrhagic stroke	All-cause mortality	CVD mortality
ID	Country	Sex	Age	Rate (95% CI)	Rate (95% CI)	Rate (95% CI)	Rate (95% CI)	Rate (95% CI)
137	Austria	Male	55 to 59	67 (50-91)	86 (77-97)	9 (7-11)	67 (62-72)	15 (13-18)
138	Austria	Male	60 to 64	70 (52-93)	142 (125-159)	12 (9-15)	109 (101-117)	28 (25-32)
139	Austria	Male	65 to 69	57 (42-77)	204 (181-233)	17 (13-21)	165 (152-176)	47 (42-53)
140	Austria	Male	70 to <75	43 (27-64)	245 (212-281)	25 (21-29)	239 (221-255)	78 (70-87)
141	Azerbaijan	Female	40 to 44	43 (35-54)	21 (17-26)	3 (3-4)	17 (13-22)	4 (3-6)
142	Azerbaijan	Female	45 to 49	51 (36-68)	29 (24-37)	6 (4-8)	26 (20-34)	9 (6-12)
143	Azerbaijan	Female	50 to 54	53 (39-71)	35 (29-44)	9 (8-12)	43 (32-55)	16 (12-22)
144	Azerbaijan	Female	55 to 59	50 (36-66)	40 (33-49)	14 (10-18)	67 (50-86)	31 (22-40)
145	Azerbaijan	Female	60 to 64	43 (28-60)	48 (40-58)	20 (16-25)	116 (89-145)	61 (46-78)
146	Azerbaijan	Female	65 to 69	33 (24-47)	52 (44-62)	28 (21-35)	179 (140-226)	104 (81-133)
147	Azerbaijan	Female	70 to <75	24 (16-36)	57 (48-70)	38 (31-45)	301 (242-385)	197 (158-254)
148	Azerbaijan	Male	40 to 44	48 (38-60)	12 (11-15)	5 (4-7)	41 (31-55)	16 (11-21)
149	Azerbaijan	Male	45 to 49	52 (37-73)	21 (18-26)	9 (7-12)	60 (45-78)	27 (20-36)
150	Azerbaijan	Male	50 to 54	52 (36-73)	37 (31-43)	14 (12-17)	94 (71-119)	46 (34-60)
151	Azerbaijan	Male	55 to 59	47 (34-63)	57 (48-68)	20 (15-26)	144 (110-177)	75 (56-95)
152	Azerbaijan	Male	60 to 64	42 (27-60)	83 (73-96)	27 (21-33)	229 (178-276)	129 (99-159)
153	Azerbaijan	Male	65 to 69	36 (25-51)	119 (103-138)	34 (26-43)	344 (268-410)	200 (155-243)
154	Azerbaijan	Male	70 to <75	29 (18-42)	136 (116-158)	47 (39-56)	523 (406-630)	334 (261-403)
155	Bahrain	Female	40 to 44	122 (87-161)	14 (12-17)	2 (1-3)	11 (8-15)	2 (1-3)
156	Bahrain	Female	45 to 49	186 (114-273)	21 (18-25)	3 (2-4)	18 (13-24)	4 (2-5)
157	Bahrain	Female	50 to 54	261 (199-337)	26 (22-30)	4 (3-5)	32 (23-41)	7 (5-10)
158	Bahrain	Female	55 to 59	346 (261-445)	38 (34-43)	5 (3-7)	64 (48-81)	17 (12-22)
159	Bahrain	Female	60 to 64	316 (224-426)	45 (39-53)	7 (5-9)	108 (82-135)	30 (22-39)
160	Bahrain	Female	65 to 69	171 (130-234)	57 (50-64)	11 (8-15)	202 (154-251)	66 (49-85)
161	Bahrain	Female	70 to <75	87 (50-149)	70 (63-78)	15 (12-19)	361 (277-449)	124 (93-159)
162	Bahrain	Male	40 to 44	126 (89-170)	4 (4-4)	2 (1-2)	14 (10-19)	4 (2-5)
163	Bahrain	Male	45 to 49	203 (123-296)	7 (6-8)	3 (2-4)	23 (17-32)	7 (5-10)
164	Bahrain	Male	50 to 54	287 (221-366)	12 (11-13)	4 (3-5)	39 (28-53)	13 (9-19)
165	Bahrain	Male	55 to 59	376 (280-494)	20 (18-22)	5 (4-7)	70 (52-94)	24 (17-33)
166	Bahrain	Male	60 to 64	350 (243-477)	39 (34-43)	7 (6-10)	121 (91-157)	40 (30-53)
167	Bahrain	Male	65 to 69	208 (156-277)	63 (57-69)	10 (8-14)	226 (172-290)	73 (54-97)
168	Bahrain	Male	70 to <75	116 (65-191)	97 (88-107)	15 (12-18)	422 (324-538)	133 (99-172)
169	Bangladesh	Female	40 to 44	51 (39-65)	10 (8-11)	3 (2-3)	22 (18-26)	7 (6-9)
170	Bangladesh	Female	45 to 49	56 (41-74)	15 (13-17)	4 (3-6)	33 (28-39)	11 (9-14)
171	Bangladesh	Female	50 to 54	58 (43-77)	20 (17-23)	7 (6-8)	49 (41-58)	18 (15-21)
172	Bangladesh	Female	55 to 59	56 (41-75)	23 (19-27)	10 (7-13)	72 (61-85)	30 (25-37)
173	Bangladesh	Female	60 to 64	52 (37-72)	28 (24-34)	14 (11-17)	113 (97-133)	53 (45-64)
174	Bangladesh	Female	65 to 69	47 (33-70)	31 (27-37)	19 (14-23)	175 (152-207)	82 (70-99)
175	Bangladesh	Female	70 to <75	54 (38-75)	40 (34-46)	25 (20-29)	286 (252-336)	131 (110-157)
176	Bangladesh	Male	40 to 44	51 (39-64)	6 (5-7)	3 (2-4)	37 (30-42)	13 (10-16)
177	Bangladesh	Male	45 to 49	47 (35-62)	11 (9-12)	5 (4-6)	51 (42-61)	20 (16-25)
178	Bangladesh	Male	50 to 54	48 (37-64)	16 (14-19)	7 (6-9)	74 (62-88)	32 (27-38)
179	Bangladesh	Male	55 to 59	55 (39-74)	25 (21-28)	11 (8-14)	112 (94-131)	50 (41-60)
180	Bangladesh	Male	60 to 64	54 (38-73)	39 (34-46)	15 (12-19)	172 (147-201)	75 (64-89)
181	Bangladesh	Male	65 to 69	46 (33-68)	50 (44-58)	21 (16-27)	262 (227-305)	118 (101-138)
182	Bangladesh	Male	70 to <75	47 (33-66)	63 (55-73)	26 (21-31)	410 (361-471)	171 (148-197)
183	Barbados	Female	40 to 44	69 (51-91)	34 (30-40)	2 (2-3)	20 (17-24)	3 (2-4)
184	Barbados	Female	45 to 49	100 (70-139)	48 (43-55)	4 (3-5)	29 (25-34)	5 (4-7)
185	Barbados	Female	50 to 54	126 (98-161)	63 (55-71)	5 (4-7)	43 (37-49)	8 (6-10)
186	Barbados	Female	55 to 59	147 (104-199)	75 (67-84)	7 (5-9)	61 (53-69)	14 (11-17)
187	Barbados	Female	60 to 64	140 (98-191)	83 (75-92)	10 (8-12)	88 (77-98)	23 (19-27)
188	Barbados	Female	65 to 69	107 (74-146)	100 (90-113)	14 (11-18)	136 (119-153)	41 (34-47)
189	Barbados	Female	70 to <75	79 (45-123)	107 (98-119)	20 (16-24)	203 (177-228)	66 (56-78)
190	Barbados	Male	40 to 44	74 (56-94)	12 (10-15)	2 (2-3)	26 (23-31)	5 (3-6)
191	Barbados	Male	45 to 49	113 (79-164)	24 (20-27)	4 (3-6)	45 (39-53)	11 (9-14)
192	Barbados	Male	50 to 54	130 (98-176)	40 (35-45)	6 (5-8)	63 (54-72)	18 (15-21)
193	Barbados	Male	55 to 59	127 (93-168)	65 (57-72)	9 (7-11)	91 (79-105)	27 (22-32)
194	Barbados	Male	60 to 64	111 (72-153)	116 (100-133)	12 (10-15)	142 (126-161)	42 (35-49)
195	Barbados	Male	65 to 69	82 (56-122)	175 (148-205)	17 (13-22)	211 (187-238)	67 (57-77)
196	Barbados	Male	70 to <75	65 (38-105)	248 (209-289)	23 (19-27)	327 (291-368)	103 (88-118)
197	Belarus	Female	40 to 44	36 (29-45)	20 (18-24)	3 (2-4)	19 (14-24)	4 (3-6)
198	Belarus	Female	45 to 49	31 (23-42)	28 (25-33)	5 (4-7)	26 (20-34)	7 (5-9)
199	Belarus	Female	50 to 54	30 (21-40)	39 (35-44)	8 (6-9)	40 (31-50)	13 (10-17)
200	Belarus	Female	55 to 59	32 (22-42)	52 (47-58)	10 (8-14)	65 (50-82)	27 (20-34)
201	Belarus	Female	60 to 64	29 (21-41)	65 (59-73)	14 (11-17)	96 (75-120)	49 (39-62)
202	Belarus	Female	65 to 69	23 (17-34)	76 (69-84)	18 (13-23)	148 (115-186)	92 (72-117)
203	Belarus	Female	70 to <75	19 (13-29)	83 (76-92)	23 (19-28)	243 (188-306)	171 (132-217)
204	Belarus	Male	40 to 44	43 (34-54)	13 (11-15)	5 (4-7)	62 (48-78)	17 (12-22)
205	Belarus	Male	45 to 49	37 (28-50)	25 (22-28)	9 (7-12)	88 (68-112)	30 (22-39)
206	Belarus	Male	50 to 54	33 (24-46)	49 (43-54)	13 (11-16)	131 (103-165)	51 (39-65)

				Diabetes type 2	Cancers	Hemorrhagic stroke	All-cause mortality	CVD mortality
ID	Country	Sex	Age	Rate (95% CI)	Rate (95% CI)	Rate (95% CI)	Rate (95% CI)	Rate (95% CI)
207	Belarus	Male	55 to 59	32 (24-44)	85 (76-94)	17 (13-22)	194 (154-240)	86 (66-108)
208	Belarus	Male	60 to 64	29 (20-42)	129 (116-142)	22 (18-27)	306 (249-371)	161 (130-197)
209	Belarus	Male	65 to 69	25 (18-36)	169 (151-188)	27 (21-34)	405 (332-488)	238 (193-287)
210	Belarus	Male	70 to <75	20 (13-31)	193 (172-215)	32 (25-39)	567 (467-680)	369 (301-442)
211	Belgium	Female	40 to 44	23 (17-29)	30 (26-35)	3 (2-3)	10 (8-11)	1 (1-1)
212	Belgium	Female	45 to 49	24 (16-33)	45 (39-53)	5 (4-5)	16 (14-19)	2 (2-3)
213	Belgium	Female	50 to 54	31 (23-42)	62 (54-71)	6 (5-7)	26 (22-30)	3 (3-4)
214	Belgium	Female	55 to 59	45 (34-62)	85 (75-97)	6 (5-7)	41 (36-47)	6 (4-7)
215	Belgium	Female	60 to 64	51 (38-68)	112 (100-126)	8 (7-9)	63 (54-71)	10 (8-13)
216	Belgium	Female	65 to 69	49 (35-68)	131 (117-146)	10 (8-12)	90 (77-102)	18 (15-22)
217	Belgium	Female	70 to <75	41 (28-59)	147 (132-166)	16 (14-17)	141 (121-161)	35 (29-42)
218	Belgium	Male	40 to 44	28 (22-36)	16 (13-19)	3 (2-3)	16 (14-18)	2 (2-3)
219	Belgium	Male	45 to 49	34 (23-48)	28 (24-33)	4 (4-5)	25 (22-29)	4 (3-5)
220	Belgium	Male	50 to 54	44 (34-57)	54 (48-62)	6 (5-6)	42 (37-49)	8 (7-10)
221	Belgium	Male	55 to 59	57 (42-75)	98 (87-112)	7 (6-8)	73 (64-83)	15 (12-18)
222	Belgium	Male	60 to 64	61 (45-80)	157 (138-183)	10 (9-11)	112 (100-127)	25 (21-30)
223	Belgium	Male	65 to 69	56 (40-74)	227 (200-264)	14 (12-16)	170 (151-192)	41 (34-48)
224	Belgium	Male	70 to <75	45 (30-64)	292 (256-348)	23 (21-25)	259 (230-293)	68 (58-79)
225	Belize	Female	40 to 44	73 (53-96)	15 (13-18)	2 (1-2)	24 (18-30)	3 (2-4)
226	Belize	Female	45 to 49	117 (76-164)	25 (22-28)	3 (3-5)	41 (33-49)	8 (6-10)
227	Belize	Female	50 to 54	149 (112-195)	29 (26-32)	5 (4-6)	59 (48-71)	13 (10-16)
228	Belize	Female	55 to 59	172 (127-226)	33 (30-36)	7 (5-9)	84 (69-100)	22 (17-27)
229	Belize	Female	60 to 64	157 (112-216)	45 (41-49)	10 (8-13)	133 (113-156)	40 (32-48)
230	Belize	Female	65 to 69	104 (76-145)	51 (47-56)	15 (11-19)	198 (169-231)	63 (51-76)
231	Belize	Female	70 to <75	68 (40-115)	67 (61-72)	21 (17-25)	323 (272-377)	110 (91-132)
232	Belize	Male	40 to 44	86 (63-109)	7 (6-8)	2 (1-2)	47 (38-61)	6 (4-8)
233	Belize	Male	45 to 49	139 (97-183)	12 (11-14)	4 (3-5)	69 (55-87)	12 (9-16)
234	Belize	Male	50 to 54	150 (115-188)	22 (20-25)	6 (5-7)	102 (82-125)	24 (18-30)
235	Belize	Male	55 to 59	120 (90-160)	36 (33-41)	9 (7-12)	151 (124-180)	42 (33-53)
236	Belize	Male	60 to 64	93 (58-139)	59 (53-66)	13 (11-16)	224 (186-262)	69 (56-84)
237	Belize	Male	65 to 69	68 (47-103)	85 (75-97)	18 (14-23)	332 (277-386)	117 (95-140)
238	Belize	Male	70 to <75	53 (34-84)	120 (105-140)	25 (20-30)	499 (419-578)	182 (149-216)
239	Benin	Female	40 to 44	30 (22-38)	12 (10-14)	2 (2-3)	44 (39-49)	5 (3-6)
240	Benin	Female	45 to 49	41 (29-56)	16 (13-19)	4 (3-5)	59 (52-67)	9 (6-11)
241	Benin	Female	50 to 54	47 (35-61)	20 (17-23)	6 (5-7)	85 (72-100)	17 (13-23)
242	Benin	Female	55 to 59	47 (33-66)	25 (21-29)	8 (6-11)	125 (105-148)	31 (23-40)
243	Benin	Female	60 to 64	49 (33-70)	34 (29-39)	11 (9-14)	195 (162-225)	54 (41-68)
244	Benin	Female	65 to 69	52 (35-72)	38 (33-42)	16 (12-21)	304 (254-354)	97 (75-120)
245	Benin	Female	70 to <75	50 (35-71)	41 (37-46)	23 (19-28)	484 (412-547)	160 (128-197)
246	Benin	Male	40 to 44	29 (21-38)	6 (5-7)	3 (2-3)	65 (56-75)	10 (7-13)
247	Benin	Male	45 to 49	37 (26-50)	9 (8-11)	5 (4-7)	87 (76-99)	17 (12-21)
248	Benin	Male	50 to 54	41 (30-54)	15 (13-17)	7 (6-9)	120 (107-133)	26 (21-33)
249	Benin	Male	55 to 59	42 (29-58)	21 (19-24)	10 (7-13)	168 (151-184)	39 (31-48)
250	Benin	Male	60 to 64	43 (29-61)	32 (29-36)	13 (11-17)	250 (230-269)	67 (56-79)
251	Benin	Male	65 to 69	44 (31-61)	48 (43-54)	18 (14-23)	370 (343-393)	98 (81-115)
252	Benin	Male	70 to <75	42 (29-58)	67 (59-76)	24 (20-29)	563 (527-599)	160 (134-186)
253	Bhutan	Female	40 to 44	64 (47-85)	9 (8-11)	1 (1-2)	20 (16-25)	5 (3-6)
254	Bhutan	Female	45 to 49	75 (52-99)	13 (11-16)	3 (2-4)	31 (24-39)	9 (6-11)
255	Bhutan	Female	50 to 54	76 (55-103)	17 (15-21)	4 (3-5)	44 (35-56)	13 (9-17)
256	Bhutan	Female	55 to 59	69 (47-98)	20 (17-25)	6 (5-8)	66 (53-83)	22 (16-30)
257	Bhutan	Female	60 to 64	60 (38-90)	24 (20-29)	9 (7-12)	105 (87-130)	38 (28-49)
258	Bhutan	Female	65 to 69	50 (33-76)	26 (22-32)	14 (10-18)	162 (136-202)	62 (47-81)
259	Bhutan	Female	70 to <75	51 (35-75)	29 (24-35)	19 (15-23)	262 (225-321)	101 (77-129)
260	Bhutan	Male	40 to 44	70 (50-92)	4 (4-5)	2 (1-2)	31 (24-40)	8 (6-11)
261	Bhutan	Male	45 to 49	69 (47-95)	7 (6-9)	3 (2-4)	43 (34-56)	14 (10-18)
262	Bhutan	Male	50 to 54	70 (49-96)	12 (10-15)	5 (4-6)	63 (49-79)	23 (17-30)
263	Bhutan	Male	55 to 59	72 (49-100)	18 (15-22)	7 (5-9)	93 (73-117)	36 (27-46)
264	Bhutan	Male	60 to 64	66 (43-94)	27 (22-32)	11 (9-14)	145 (118-178)	58 (45-73)
265	Bhutan	Male	65 to 69	50 (35-73)	35 (30-42)	17 (13-21)	223 (183-269)	91 (71-114)
266	Bhutan	Male	70 to <75	46 (31-68)	42 (36-49)	21 (17-26)	354 (294-418)	142 (111-174)
267	Bolivia	Female	40 to 44	42 (31-57)	17 (14-20)	3 (2-3)	24 (17-33)	4 (3-6)
268	Bolivia	Female	45 to 49	52 (35-73)	25 (20-30)	5 (3-6)	36 (26-49)	8 (5-11)
269	Bolivia	Female	50 to 54	58 (42-78)	35 (27-42)	7 (5-8)	56 (40-76)	12 (8-17)
270	Bolivia	Female	55 to 59	60 (42-84)	46 (35-56)	9 (6-11)	84 (61-114)	18 (12-26)
271	Bolivia	Female	60 to 64	56 (37-81)	58 (45-70)	11 (9-14)	129 (98-173)	30 (20-42)
272	Bolivia	Female	65 to 69	46 (32-65)	76 (59-90)	15 (11-20)	199 (151-270)	50 (35-70)
273	Bolivia	Female	70 to <75	39 (26-58)	94 (73-114)	20 (16-25)	315 (243-425)	86 (61-122)
274	Bolivia	Male	40 to 44	47 (36-61)	6 (5-7)	2 (2-3)	31 (22-42)	5 (3-8)
275	Bolivia	Male	45 to 49	60 (42-82)	9 (8-13)	4 (3-5)	43 (31-58)	9 (6-13)
276	Bolivia	Male	50 to 54	65 (48-87)	16 (14-19)	6 (5-7)	63 (46-81)	15 (10-21)

				Diabetes type 2	Cancers	Hemorrhagic stroke	All-cause mortality	CVD mortality
ID	Country	Sex	Age	Rate (95% CI)	Rate (95% CI)	Rate (95% CI)	Rate (95% CI)	Rate (95% CI)
277	Bolivia	Male	55 to 59	64 (45-87)	27 (23-32)	8 (6-10)	92 (68-119)	24 (17-34)
278	Bolivia	Male	60 to 64	58 (39-84)	45 (40-51)	10 (8-12)	142 (108-177)	38 (28-50)
279	Bolivia	Male	65 to 69	49 (34-73)	70 (62-80)	13 (10-17)	215 (168-263)	61 (45-79)
280	Bolivia	Male	70 to <75	44 (29-66)	104 (90-120)	18 (14-21)	336 (266-403)	96 (74-121)
281	Bosnia and Herzegovina	Female	40 to 44	45 (35-58)	17 (13-20)	3 (2-4)	11 (9-14)	2 (2-3)
282	Bosnia and Herzegovina	Female	45 to 49	46 (29-70)	25 (21-30)	5 (3-6)	20 (16-25)	5 (4-7)
283	Bosnia and Herzegovina	Female	50 to 54	57 (41-76)	36 (30-41)	6 (5-8)	31 (25-39)	8 (6-10)
284	Bosnia and Herzegovina	Female	55 to 59	77 (55-104)	45 (38-52)	7 (5-11)	47 (38-58)	14 (11-18)
285	Bosnia and Herzegovina	Female	60 to 64	76 (51-105)	60 (52-68)	10 (8-13)	85 (69-103)	30 (24-37)
286	Bosnia and Herzegovina	Female	65 to 69	54 (39-71)	76 (68-87)	14 (10-18)	157 (129-189)	68 (55-84)
287	Bosnia and Herzegovina	Female	70 to <75	35 (20-55)	87 (77-100)	20 (16-25)	269 (221-326)	135 (110-165)
288	Bosnia and Herzegovina	Male	40 to 44	71 (57-87)	9 (8-11)	4 (3-5)	21 (16-27)	6 (4-8)
289	Bosnia and Herzegovina	Male	45 to 49	72 (48-97)	19 (16-22)	6 (5-8)	38 (29-47)	12 (9-16)
290	Bosnia and Herzegovina	Male	50 to 54	72 (50-98)	35 (31-39)	8 (7-11)	65 (51-80)	22 (17-29)
291	Bosnia and Herzegovina	Male	55 to 59	71 (49-94)	58 (51-67)	11 (8-14)	97 (78-119)	33 (25-41)
292	Bosnia and Herzegovina	Male	60 to 64	60 (38-87)	93 (84-105)	15 (12-19)	166 (137-198)	60 (49-73)
293	Bosnia and Herzegovina	Male	65 to 69	39 (28-55)	140 (126-157)	20 (15-25)	280 (235-328)	111 (91-135)
294	Bosnia and Herzegovina	Male	70 to <75	25 (15-40)	175 (156-199)	27 (22-33)	430 (362-502)	192 (158-228)
295	Botswana	Female	40 to 44	60 (45-78)	18 (15-21)	2 (1-2)	85 (59-114)	5 (1-9)
296	Botswana	Female	45 to 49	80 (55-110)	24 (20-28)	3 (2-4)	100 (60-143)	9 (2-17)
297	Botswana	Female	50 to 54	95 (71-127)	31 (26-36)	4 (3-6)	119 (62-172)	16 (3-29)
298	Botswana	Female	55 to 59	105 (71-146)	40 (34-47)	6 (4-9)	124 (42-197)	26 (6-45)
299	Botswana	Female	60 to 64	114 (79-156)	45 (39-51)	10 (8-13)	166 (53-264)	51 (13-85)
300	Botswana	Female	65 to 69	125 (90-167)	54 (48-61)	16 (11-21)	240 (73-384)	82 (22-137)
301	Botswana	Female	70 to <75	111 (75-155)	62 (54-71)	23 (18-28)	375 (112-596)	141 (41-230)
302	Botswana	Male	40 to 44	74 (56-99)	8 (7-10)	2 (1-2)	144 (106-182)	9 (3-15)
303	Botswana	Male	45 to 49	103 (70-147)	17 (14-20)	4 (3-5)	179 (120-239)	16 (6-27)
304	Botswana	Male	50 to 54	122 (90-162)	31 (27-36)	6 (4-7)	214 (137-287)	28 (12-46)
305	Botswana	Male	55 to 59	130 (86-186)	52 (44-60)	8 (6-11)	241 (139-330)	45 (21-70)
306	Botswana	Male	60 to 64	134 (95-186)	69 (59-79)	12 (10-15)	313 (189-423)	82 (42-122)
307	Botswana	Male	65 to 69	136 (99-180)	97 (84-112)	18 (13-23)	424 (252-552)	121 (65-165)
308	Botswana	Male	70 to <75	114 (73-160)	122 (105-143)	25 (20-30)	600 (373-756)	179 (106-235)
309	Brazil	Female	40 to 44	25 (18-33)	18 (17-19)	3 (2-3)	18 (17-18)	4 (4-4)
310	Brazil	Female	45 to 49	31 (22-42)	26 (24-28)	5 (4-6)	26 (25-27)	7 (7-7)
311	Brazil	Female	50 to 54	37 (28-48)	35 (33-38)	6 (5-8)	39 (37-40)	11 (10-12)
312	Brazil	Female	55 to 59	45 (33-60)	46 (43-50)	8 (6-10)	59 (56-61)	18 (17-19)
313	Brazil	Female	60 to 64	48 (35-63)	60 (56-64)	10 (8-12)	89 (85-93)	29 (27-30)
314	Brazil	Female	65 to 69	45 (31-64)	76 (71-81)	12 (10-15)	139 (134-146)	47 (45-50)
315	Brazil	Female	70 to <75	47 (33-66)	91 (85-98)	16 (13-19)	220 (211-230)	77 (73-81)
316	Brazil	Male	40 to 44	30 (22-39)	10 (9-11)	2 (2-3)	39 (37-40)	7 (6-7)
317	Brazil	Male	45 to 49	37 (27-50)	18 (17-21)	4 (3-6)	53 (50-55)	12 (11-13)
318	Brazil	Male	50 to 54	42 (32-55)	33 (30-35)	6 (5-7)	75 (72-79)	21 (19-22)
319	Brazil	Male	55 to 59	45 (32-62)	54 (50-59)	8 (6-11)	110 (106-115)	34 (32-36)
320	Brazil	Male	60 to 64	48 (33-65)	83 (78-91)	11 (9-13)	160 (154-167)	54 (52-57)
321	Brazil	Male	65 to 69	49 (33-70)	121 (115-132)	14 (11-17)	241 (232-250)	85 (81-89)
322	Brazil	Male	70 to <75	54 (38-75)	165 (155-182)	17 (14-20)	365 (352-379)	131 (124-137)
323	Brunei	Female	40 to 44	69 (45-94)	33 (28-39)	2 (2-3)	16 (11-23)	3 (2-4)
324	Brunei	Female	45 to 49	96 (54-155)	48 (41-56)	4 (3-6)	24 (17-34)	5 (3-7)
325	Brunei	Female	50 to 54	136 (98-189)	57 (49-65)	6 (5-8)	37 (26-50)	8 (5-11)
326	Brunei	Female	55 to 59	189 (139-255)	70 (62-79)	7 (6-10)	56 (38-73)	13 (8-17)
327	Brunei	Female	60 to 64	178 (122-248)	83 (74-93)	10 (8-13)	88 (59-109)	23 (15-30)
328	Brunei	Female	65 to 69	103 (76-141)	99 (89-110)	14 (10-18)	140 (98-166)	38 (27-48)
329	Brunei	Female	70 to <75	57 (35-92)	115 (103-131)	21 (17-25)	229 (173-260)	68 (51-82)
330	Brunei	Male	40 to 44	128 (93-169)	13 (12-15)	3 (2-4)	24 (17-36)	8 (5-13)
331	Brunei	Male	45 to 49	192 (118-273)	21 (18-24)	5 (4-6)	37 (26-52)	13 (9-19)
332	Brunei	Male	50 to 54	204 (145-277)	35 (31-40)	7 (6-9)	58 (41-78)	20 (14-28)
333	Brunei	Male	55 to 59	163 (120-226)	59 (53-67)	10 (7-14)	91 (66-117)	30 (21-40)
334	Brunei	Male	60 to 64	120 (66-197)	98 (86-111)	14 (11-17)	145 (108-178)	46 (34-59)
335	Brunei	Male	65 to 69	74 (47-112)	153 (135-175)	19 (14-24)	233 (175-273)	70 (51-86)
336	Brunei	Male	70 to <75	44 (26-72)	213 (186-242)	25 (20-31)	373 (290-428)	115 (87-138)
337	Bulgaria	Female	40 to 44	38 (30-47)	28 (24-32)	3 (3-4)	16 (12-21)	4 (3-6)
338	Bulgaria	Female	45 to 49	37 (27-50)	40 (35-46)	6 (4-7)	25 (20-32)	8 (6-11)
339	Bulgaria	Female	50 to 54	39 (28-51)	55 (48-62)	8 (6-9)	40 (31-50)	14 (11-18)
340	Bulgaria	Female	55 to 59	43 (30-57)	68 (60-77)	10 (7-13)	59 (47-74)	24 (19-31)
341	Bulgaria	Female	60 to 64	43 (31-58)	77 (69-87)	14 (11-17)	84 (68-105)	40 (32-50)
342	Bulgaria	Female	65 to 69	40 (29-54)	96 (86-107)	19 (14-24)	147 (118-184)	81 (65-101)
343	Bulgaria	Female	70 to <75	33 (22-48)	106 (96-117)	27 (22-32)	257 (205-323)	160 (128-202)
344	Bulgaria	Male	40 to 44	56 (45-68)	15 (14-17)	5 (4-6)	33 (26-41)	12 (9-16)
345	Bulgaria	Male	45 to 49	53 (37-70)	28 (25-30)	9 (6-11)	57 (45-72)	24 (19-31)
346	Bulgaria	Male	50 to 54	50 (36-68)	49 (45-53)	13 (11-16)	97 (77-120)	44 (34-55)

				Diabetes type 2	Cancers	Hemorrhagic stroke	All-cause mortality	CVD mortality
ID	Country	Sex	Age	Rate (95% CI)	Rate (95% CI)	Rate (95% CI)	Rate (95% CI)	Rate (95% CI)
347	Bulgaria	Male	55 to 59	49 (36-63)	80 (73-87)	18 (14-23)	154 (123-188)	74 (58-90)
348	Bulgaria	Male	60 to 64	44 (30-59)	112 (103-123)	24 (19-29)	216 (176-259)	110 (90-134)
349	Bulgaria	Male	65 to 69	35 (24-50)	154 (142-169)	31 (24-40)	328 (269-391)	180 (148-217)
350	Bulgaria	Male	70 to <75	27 (17-40)	188 (171-209)	42 (34-50)	476 (391-567)	287 (235-343)
351	Burkina Faso	Female	40 to 44	29 (22-38)	12 (9-15)	2 (2-3)	50 (48-53)	6 (5-7)
352	Burkina Faso	Female	45 to 49	38 (27-51)	16 (13-19)	4 (3-5)	69 (64-74)	11 (8-14)
353	Burkina Faso	Female	50 to 54	43 (32-55)	20 (16-23)	5 (4-7)	101 (94-110)	20 (16-26)
354	Burkina Faso	Female	55 to 59	43 (30-61)	25 (20-29)	7 (5-10)	149 (138-161)	36 (28-44)
355	Burkina Faso	Female	60 to 64	45 (31-64)	34 (28-39)	11 (8-13)	230 (215-245)	64 (52-76)
356	Burkina Faso	Female	65 to 69	47 (33-66)	38 (33-44)	15 (11-20)	358 (333-384)	120 (98-142)
357	Burkina Faso	Female	70 to <75	47 (33-65)	42 (37-47)	22 (18-26)	564 (532-597)	198 (164-233)
358	Burkina Faso	Male	40 to 44	30 (21-40)	6 (5-8)	2 (2-3)	66 (62-70)	9 (7-12)
359	Burkina Faso	Male	45 to 49	37 (25-53)	9 (7-11)	4 (3-6)	88 (82-95)	16 (12-20)
360	Burkina Faso	Male	50 to 54	42 (30-57)	14 (12-17)	6 (5-8)	120 (111-130)	25 (20-31)
361	Burkina Faso	Male	55 to 59	45 (31-63)	20 (17-24)	9 (6-12)	165 (153-177)	41 (33-48)
362	Burkina Faso	Male	60 to 64	47 (33-65)	30 (27-34)	12 (10-15)	248 (232-264)	70 (58-82)
363	Burkina Faso	Male	65 to 69	49 (33-69)	44 (39-51)	17 (13-22)	366 (343-388)	101 (84-119)
364	Burkina Faso	Male	70 to <75	46 (32-66)	65 (56-74)	22 (18-27)	573 (548-596)	175 (149-202)
365	Burundi	Female	40 to 44	25 (19-34)	17 (13-21)	2 (2-3)	68 (55-85)	8 (5-11)
366	Burundi	Female	45 to 49	30 (20-41)	24 (18-28)	4 (3-5)	94 (75-115)	14 (10-20)
367	Burundi	Female	50 to 54	36 (27-48)	32 (26-38)	6 (4-7)	138 (110-165)	26 (18-35)
368	Burundi	Female	55 to 59	44 (31-63)	35 (29-41)	8 (6-10)	196 (160-234)	44 (31-59)
369	Burundi	Female	60 to 64	51 (36-70)	46 (39-54)	12 (10-15)	288 (241-337)	75 (55-97)
370	Burundi	Female	65 to 69	55 (39-74)	52 (45-60)	19 (14-24)	438 (376-511)	130 (96-167)
371	Burundi	Female	70 to <75	53 (38-72)	54 (46-61)	28 (23-34)	669 (577-778)	222 (167-279)
372	Burundi	Male	40 to 44	30 (23-38)	7 (6-8)	2 (2-3)	88 (70-104)	9 (6-13)
373	Burundi	Male	45 to 49	46 (32-64)	10 (9-12)	4 (3-5)	115 (92-140)	15 (10-22)
374	Burundi	Male	50 to 54	55 (42-70)	16 (13-18)	6 (5-7)	153 (124-192)	24 (17-34)
375	Burundi	Male	55 to 59	57 (41-77)	24 (21-28)	8 (5-10)	208 (169-262)	39 (28-52)
376	Burundi	Male	60 to 64	57 (41-80)	39 (30-46)	12 (9-15)	301 (249-380)	71 (53-93)
377	Burundi	Male	65 to 69	56 (40-76)	56 (42-67)	18 (13-23)	436 (367-541)	112 (87-145)
378	Burundi	Male	70 to <75	52 (36-71)	81 (58-101)	25 (21-30)	660 (563-797)	183 (142-236)
379	Cambodia	Female	40 to 44	45 (34-58)	13 (10-17)	3 (3-4)	32 (29-38)	6 (5-8)
380	Cambodia	Female	45 to 49	55 (39-74)	19 (15-23)	6 (4-8)	49 (43-56)	11 (9-14)
381	Cambodia	Female	50 to 54	63 (47-81)	24 (19-28)	9 (8-11)	74 (65-85)	21 (16-26)
382	Cambodia	Female	55 to 59	70 (52-92)	30 (25-35)	13 (10-17)	112 (98-125)	34 (27-41)
383	Cambodia	Female	60 to 64	65 (47-90)	36 (31-41)	18 (15-22)	170 (152-188)	54 (44-64)
384	Cambodia	Female	65 to 69	50 (37-67)	43 (38-49)	25 (19-31)	266 (239-289)	98 (83-115)
385	Cambodia	Female	70 to <75	38 (26-53)	49 (44-55)	38 (31-44)	419 (385-450)	163 (141-187)
386	Cambodia	Male	40 to 44	43 (32-55)	6 (5-6)	3 (2-4)	61 (52-69)	10 (7-13)
387	Cambodia	Male	45 to 49	42 (30-59)	10 (9-11)	6 (4-7)	83 (72-94)	18 (14-23)
388	Cambodia	Male	50 to 54	44 (32-62)	16 (14-19)	9 (8-11)	117 (105-130)	31 (25-37)
389	Cambodia	Male	55 to 59	49 (35-65)	27 (23-31)	14 (11-18)	167 (151-188)	50 (41-59)
390	Cambodia	Male	60 to 64	49 (34-65)	41 (35-47)	20 (16-24)	248 (229-278)	77 (66-90)
391	Cambodia	Male	65 to 69	43 (30-59)	61 (51-72)	26 (19-33)	373 (343-410)	123 (106-142)
392	Cambodia	Male	70 to <75	37 (25-53)	85 (71-102)	36 (30-43)	578 (526-625)	199 (172-229)
393	Cameroon	Female	40 to 44	39 (29-51)	14 (10-18)	2 (2-3)	83 (71-95)	5 (3-7)
394	Cameroon	Female	45 to 49	55 (37-76)	19 (14-23)	4 (3-5)	92 (75-111)	9 (6-13)
395	Cameroon	Female	50 to 54	63 (46-84)	23 (18-28)	5 (4-6)	111 (85-145)	18 (11-27)
396	Cameroon	Female	55 to 59	62 (43-89)	29 (23-35)	7 (5-9)	143 (101-192)	30 (19-45)
397	Cameroon	Female	60 to 64	65 (43-92)	39 (32-46)	10 (8-13)	210 (145-277)	51 (32-74)
398	Cameroon	Female	65 to 69	69 (48-97)	44 (37-52)	15 (11-20)	321 (221-421)	91 (59-131)
399	Cameroon	Female	70 to <75	66 (46-94)	48 (41-56)	22 (17-27)	503 (358-637)	154 (105-212)
400	Cameroon	Male	40 to 44	35 (25-46)	7 (6-10)	3 (2-3)	109 (92-126)	10 (6-15)
401	Cameroon	Male	45 to 49	45 (31-63)	11 (9-14)	5 (3-6)	131 (107-156)	17 (10-23)
402	Cameroon	Male	50 to 54	52 (38-70)	18 (15-21)	7 (5-8)	158 (132-184)	27 (17-35)
403	Cameroon	Male	55 to 59	54 (37-75)	25 (21-30)	9 (7-12)	197 (162-232)	39 (28-51)
404	Cameroon	Male	60 to 64	56 (39-78)	37 (33-43)	13 (10-16)	277 (230-320)	65 (48-82)
405	Cameroon	Male	65 to 69	57 (40-79)	55 (49-63)	17 (13-22)	397 (334-453)	96 (73-119)
406	Cameroon	Male	70 to <75	53 (36-73)	77 (67-87)	24 (19-28)	599 (523-681)	158 (126-192)
407	Canada	Female	40 to 44	27 (20-35)	27 (23-34)	3 (2-3)	10 (9-10)	1 (1-1)
408	Canada	Female	45 to 49	37 (27-51)	40 (33-48)	4 (3-6)	15 (14-16)	2 (1-2)
409	Canada	Female	50 to 54	52 (40-65)	56 (47-67)	5 (4-7)	24 (23-25)	3 (2-4)
410	Canada	Female	55 to 59	69 (51-91)	76 (67-89)	6 (4-8)	37 (36-39)	5 (4-6)
411	Canada	Female	60 to 64	72 (54-94)	96 (83-114)	7 (5-9)	54 (52-57)	9 (7-10)
412	Canada	Female	65 to 69	59 (45-78)	129 (114-149)	9 (6-12)	93 (89-98)	18 (15-21)
413	Canada	Female	70 to <75	43 (29-63)	155 (138-175)	14 (11-17)	155 (147-162)	36 (31-40)
414	Canada	Male	40 to 44	41 (30-52)	14 (12-17)	2 (2-3)	15 (15-16)	3 (2-3)
415	Canada	Male	45 to 49	57 (38-80)	23 (20-26)	4 (2-5)	22 (21-23)	5 (4-6)
416	Canada	Male	50 to 54	79 (63-103)	43 (38-49)	5 (4-6)	36 (34-38)	9 (7-10)

				Diabetes type 2	Cancers	Hemorrhagic stroke	All-cause mortality	CVD mortality
ID	Country	Sex	Age	Rate (95% CI)	Rate (95% CI)	Rate (95% CI)	Rate (95% CI)	Rate (95% CI)
417	Canada	Male	55 to 59	108 (80-140)	75 (67-87)	6 (5-9)	59 (56-62)	15 (13-17)
418	Canada	Male	60 to 64	109 (82-144)	122 (106-144)	9 (7-11)	86 (82-90)	22 (19-25)
419	Canada	Male	65 to 69	82 (63-111)	192 (167-230)	12 (9-16)	145 (138-152)	38 (34-43)
420	Canada	Male	70 to <75	56 (36-84)	261 (227-312)	19 (15-23)	240 (228-251)	66 (59-73)
421	Cape Verde	Female	40 to 44	31 (23-41)	10 (9-13)	2 (1-2)	18 (15-22)	2 (2-3)
422	Cape Verde	Female	45 to 49	44 (31-62)	15 (13-18)	3 (2-4)	25 (20-31)	5 (3-6)
423	Cape Verde	Female	50 to 54	52 (39-69)	18 (16-22)	5 (4-6)	37 (29-46)	10 (7-13)
424	Cape Verde	Female	55 to 59	54 (38-76)	25 (22-29)	6 (5-9)	55 (43-69)	17 (12-22)
425	Cape Verde	Female	60 to 64	57 (41-79)	36 (32-40)	9 (7-11)	90 (69-108)	32 (24-40)
426	Cape Verde	Female	65 to 69	61 (42-87)	44 (40-49)	12 (9-16)	142 (110-168)	53 (40-65)
427	Cape Verde	Female	70 to <75	59 (41-85)	52 (48-57)	17 (14-21)	231 (186-263)	92 (73-109)
428	Cape Verde	Male	40 to 44	34 (25-46)	7 (6-8)	2 (2-3)	49 (39-64)	6 (4-9)
429	Cape Verde	Male	45 to 49	45 (32-63)	14 (12-15)	5 (3-6)	69 (55-88)	12 (8-17)
430	Cape Verde	Male	50 to 54	50 (36-68)	24 (22-27)	7 (6-9)	99 (80-124)	23 (17-30)
431	Cape Verde	Male	55 to 59	49 (34-67)	35 (32-39)	10 (7-13)	147 (117-178)	40 (30-52)
432	Cape Verde	Male	60 to 64	49 (34-68)	57 (51-64)	14 (11-17)	220 (178-261)	71 (55-88)
433	Cape Verde	Male	65 to 69	50 (35-71)	98 (86-110)	19 (14-23)	332 (276-384)	108 (87-129)
434	Cape Verde	Male	70 to <75	46 (30-65)	148 (124-172)	25 (20-30)	506 (431-571)	185 (151-218)
435	Central African Republic	Female	40 to 44	35 (27-47)	19 (11-27)	3 (2-3)	145 (112-187)	9 (5-15)
436	Central African Republic	Female	45 to 49	42 (29-57)	24 (16-31)	4 (3-6)	172 (122-228)	19 (11-29)
437	Central African Republic	Female	50 to 54	48 (36-64)	30 (21-38)	6 (5-8)	216 (148-282)	30 (17-45)
438	Central African Republic	Female	55 to 59	54 (38-74)	37 (27-45)	9 (6-12)	276 (187-362)	55 (33-82)
439	Central African Republic	Female	60 to 64	56 (39-77)	43 (34-52)	14 (11-17)	380 (265-487)	98 (63-139)
440	Central African Republic	Female	65 to 69	54 (39-72)	52 (43-61)	22 (16-28)	562 (405-713)	178 (118-242)
441	Central African Republic	Female	70 to <75	48 (33-66)	60 (51-69)	32 (26-39)	845 (620-1066)	307 (214-407)
442	Central African Republic	Male	40 to 44	47 (35-60)	7 (5-9)	3 (2-4)	192 (152-243)	17 (11-25)
443	Central African Republic	Male	45 to 49	66 (47-88)	13 (10-17)	5 (4-7)	252 (190-326)	31 (19-48)
444	Central African Republic	Male	50 to 54	72 (55-93)	22 (17-28)	8 (6-10)	322 (233-421)	51 (33-74)
445	Central African Republic	Male	55 to 59	67 (49-91)	34 (26-41)	11 (8-15)	402 (294-512)	78 (53-110)
446	Central African Republic	Male	60 to 64	62 (43-85)	48 (40-57)	17 (13-21)	537 (404-654)	130 (92-174)
447	Central African Republic	Male	65 to 69	57 (41-77)	66 (57-77)	25 (19-32)	727 (565-854)	192 (140-246)
448	Central African Republic	Male	70 to <75	49 (33-68)	87 (75-102)	35 (28-42)	1030 (833-1165)	286 (221-359)
449	Chad	Female	40 to 44	28 (22-37)	12 (10-15)	2 (2-3)	69 (61-78)	4 (3-6)
450	Chad	Female	45 to 49	40 (29-54)	16 (14-19)	4 (3-5)	79 (69-91)	8 (6-11)
451	Chad	Female	50 to 54	45 (34-59)	20 (17-23)	6 (4-7)	98 (84-115)	17 (13-23)
452	Chad	Female	55 to 59	45 (32-61)	25 (21-29)	8 (5-10)	132 (111-158)	31 (22-40)
453	Chad	Female	60 to 64	46 (31-64)	33 (29-38)	11 (9-14)	198 (165-237)	54 (41-70)
454	Chad	Female	65 to 69	49 (33-69)	37 (32-41)	16 (11-20)	307 (253-366)	97 (73-125)
455	Chad	Female	70 to <75	48 (34-67)	41 (36-46)	23 (18-27)	489 (416-574)	162 (127-205)
456	Chad	Male	40 to 44	27 (20-35)	6 (5-8)	3 (2-3)	85 (76-96)	9 (6-12)
457	Chad	Male	45 to 49	35 (24-49)	9 (8-11)	5 (3-6)	105 (90-121)	15 (11-20)
458	Chad	Male	50 to 54	39 (29-53)	14 (13-17)	7 (6-8)	132 (114-152)	25 (19-32)
459	Chad	Male	55 to 59	39 (28-55)	20 (18-23)	9 (7-12)	172 (146-197)	38 (29-49)
460	Chad	Male	60 to 64	40 (28-57)	31 (27-34)	13 (10-16)	252 (217-283)	66 (53-82)
461	Chad	Male	65 to 69	42 (29-58)	46 (40-52)	18 (13-23)	370 (316-410)	99 (78-120)
462	Chad	Male	70 to <75	40 (28-54)	66 (57-75)	24 (20-29)	576 (510-625)	169 (136-204)
463	Chile	Female	40 to 44	23 (17-31)	16 (15-18)	2 (2-3)	10 (6-15)	2 (1-2)
464	Chile	Female	45 to 49	33 (23-47)	24 (21-27)	4 (3-5)	16 (11-23)	3 (2-4)
465	Chile	Female	50 to 54	46 (36-60)	31 (29-34)	5 (4-6)	26 (18-36)	5 (3-7)
466	Chile	Female	55 to 59	62 (47-84)	40 (37-44)	6 (5-9)	39 (27-55)	8 (5-11)
467	Chile	Female	60 to 64	61 (45-83)	54 (51-58)	9 (7-11)	63 (43-87)	13 (9-19)
468	Chile	Female	65 to 69	43 (33-56)	72 (67-76)	12 (9-15)	103 (71-144)	24 (16-34)
469	Chile	Female	70 to <75	28 (18-41)	89 (83-95)	17 (13-20)	166 (113-234)	44 (29-61)
470	Chile	Male	40 to 44	30 (22-38)	8 (7-9)	2 (2-3)	21 (15-30)	3 (2-5)
471	Chile	Male	45 to 49	42 (27-58)	13 (12-14)	4 (3-6)	31 (22-45)	6 (4-10)
472	Chile	Male	50 to 54	58 (45-73)	23 (21-24)	6 (5-8)	48 (33-68)	11 (7-17)
473	Chile	Male	55 to 59	78 (58-105)	39 (36-42)	9 (6-12)	72 (51-99)	18 (13-26)
474	Chile	Male	60 to 64	76 (56-104)	71 (65-78)	12 (10-15)	114 (83-154)	31 (22-43)
475	Chile	Male	65 to 69	51 (39-68)	118 (107-130)	17 (13-22)	192 (140-257)	54 (39-73)
476	Chile	Male	70 to <75	32 (20-49)	170 (152-189)	24 (19-28)	302 (221-404)	89 (64-120)
477	China	Female	40 to 44	30 (23-38)	16 (14-18)	5 (4-6)	12 (11-12)	3 (2-3)
478	China	Female	45 to 49	48 (35-65)	24 (22-26)	9 (7-11)	19 (18-20)	5 (5-5)
479	China	Female	50 to 54	60 (49-76)	33 (30-36)	13 (11-15)	30 (29-31)	10 (9-10)
480	China	Female	55 to 59	67 (52-85)	41 (38-45)	18 (14-22)	45 (44-47)	15 (14-16)
481	China	Female	60 to 64	62 (46-82)	54 (50-58)	24 (20-29)	80 (77-83)	31 (29-32)
482	China	Female	65 to 69	44 (33-62)	66 (62-71)	32 (25-40)	132 (127-137)	56 (54-59)
483	China	Female	70 to <75	31 (19-48)	79 (74-84)	46 (39-54)	221 (214-229)	104 (99-108)
484	China	Male	40 to 44	36 (26-48)	15 (14-16)	6 (6-8)	27 (26-28)	7 (6-7)
485	China	Male	45 to 49	41 (29-54)	26 (25-27)	13 (11-16)	42 (40-43)	12 (11-13)
486	China	Male	50 to 54	41 (30-55)	43 (41-45)	20 (17-23)	64 (62-66)	21 (20-21)

				Diabetes type 2	Cancers	Hemorrhagic stroke	All-cause mortality	CVD mortality
ID	Country	Sex	Age	Rate (95% CI)	Rate (95% CI)	Rate (95% CI)	Rate (95% CI)	Rate (95% CI)
487	China	Male	55 to 59	38 (26-51)	66 (63-69)	26 (21-33)	97 (94-100)	32 (31-34)
488	China	Male	60 to 64	34 (22-47)	102 (98-106)	36 (30-43)	164 (160-169)	60 (57-62)
489	China	Male	65 to 69	30 (21-44)	140 (134-146)	51 (41-62)	264 (257-272)	105 (101-109)
490	China	Male	70 to <75	25 (16-39)	175 (168-183)	68 (59-78)	413 (402-424)	180 (173-185)
491	Colombia	Female	40 to 44	30 (23-39)	15 (13-18)	2 (1-3)	11 (9-13)	2 (1-2)
492	Colombia	Female	45 to 49	33 (24-43)	22 (18-27)	3 (2-4)	16 (14-18)	3 (3-4)
493	Colombia	Female	50 to 54	37 (28-48)	29 (25-35)	5 (4-6)	25 (22-29)	6 (5-7)
494	Colombia	Female	55 to 59	42 (31-56)	37 (32-44)	6 (5-8)	38 (33-43)	10 (8-12)
495	Colombia	Female	60 to 64	43 (32-57)	49 (42-57)	8 (7-11)	62 (54-69)	18 (15-21)
496	Colombia	Female	65 to 69	41 (30-57)	65 (57-74)	12 (9-15)	106 (93-119)	34 (29-39)
497	Colombia	Female	70 to <75	40 (28-55)	86 (76-100)	17 (13-20)	192 (168-216)	67 (57-78)
498	Colombia	Male	40 to 44	38 (30-48)	7 (5-9)	2 (1-2)	22 (20-26)	3 (2-4)
499	Colombia	Male	45 to 49	49 (36-66)	11 (9-15)	3 (2-4)	28 (24-32)	5 (4-6)
500	Colombia	Male	50 to 54	53 (40-70)	19 (16-24)	4 (3-5)	40 (34-45)	10 (8-12)
501	Colombia	Male	55 to 59	52 (38-71)	33 (27-41)	6 (4-8)	59 (52-67)	17 (15-20)
502	Colombia	Male	60 to 64	50 (36-68)	57 (48-67)	9 (7-11)	96 (85-107)	31 (27-36)
503	Colombia	Male	65 to 69	47 (33-64)	94 (79-111)	12 (9-15)	165 (146-185)	57 (49-66)
504	Colombia	Male	70 to <75	45 (31-64)	141 (121-168)	17 (14-20)	292 (260-327)	105 (92-120)
505	Comoros	Female	40 to 44	28 (20-36)	15 (11-20)	2 (1-2)	36 (31-44)	6 (4-7)
506	Comoros	Female	45 to 49	32 (22-44)	20 (16-28)	3 (2-4)	53 (44-65)	10 (7-14)
507	Comoros	Female	50 to 54	40 (30-53)	28 (22-37)	4 (3-5)	82 (65-100)	17 (12-23)
508	Comoros	Female	55 to 59	52 (36-72)	31 (25-40)	6 (4-8)	125 (96-148)	30 (21-40)
509	Comoros	Female	60 to 64	60 (43-81)	40 (33-50)	9 (7-12)	193 (150-221)	52 (37-66)
510	Comoros	Female	65 to 69	65 (45-92)	46 (38-55)	14 (11-19)	302 (233-345)	93 (68-116)
511	Comoros	Female	70 to <75	63 (43-88)	47 (40-55)	21 (18-26)	474 (377-529)	162 (124-198)
512	Comoros	Male	40 to 44	31 (23-40)	6 (5-8)	2 (1-2)	47 (39-58)	8 (5-11)
513	Comoros	Male	45 to 49	47 (33-63)	10 (8-11)	3 (2-5)	66 (55-80)	13 (9-18)
514	Comoros	Male	50 to 54	57 (43-71)	15 (12-18)	5 (4-6)	95 (78-114)	21 (15-29)
515	Comoros	Male	55 to 59	58 (42-80)	24 (19-28)	7 (5-9)	138 (116-160)	33 (25-45)
516	Comoros	Male	60 to 64	59 (43-81)	40 (29-49)	10 (8-13)	210 (179-240)	57 (44-76)
517	Comoros	Male	65 to 69	59 (42-82)	58 (41-73)	15 (11-19)	314 (268-358)	91 (69-120)
518	Comoros	Male	70 to <75	56 (38-78)	85 (58-109)	21 (17-26)	484 (423-554)	145 (113-185)
519	Congo	Female	40 to 44	49 (36-63)	20 (12-26)	3 (2-3)	92 (69-114)	9 (5-13)
520	Congo	Female	45 to 49	57 (39-77)	25 (16-33)	4 (3-6)	116 (87-147)	17 (11-25)
521	Congo	Female	50 to 54	65 (48-85)	31 (22-40)	6 (5-8)	151 (112-189)	27 (18-39)
522	Congo	Female	55 to 59	74 (51-103)	38 (28-48)	9 (6-11)	200 (150-252)	49 (33-68)
523	Congo	Female	60 to 64	76 (53-108)	45 (36-53)	13 (10-16)	279 (215-344)	81 (57-107)
524	Congo	Female	65 to 69	71 (49-98)	54 (46-63)	20 (14-25)	417 (328-513)	146 (109-186)
525	Congo	Female	70 to <75	61 (39-87)	62 (53-72)	29 (24-34)	640 (497-795)	258 (193-329)
526	Congo	Male	40 to 44	42 (32-56)	5 (5-7)	2 (1-2)	79 (63-100)	6 (4-9)
527	Congo	Male	45 to 49	61 (42-83)	10 (8-12)	3 (2-4)	102 (78-130)	11 (7-16)
528	Congo	Male	50 to 54	68 (51-90)	16 (14-19)	5 (4-6)	127 (96-162)	18 (12-27)
529	Congo	Male	55 to 59	65 (48-88)	26 (23-31)	7 (5-10)	163 (119-209)	30 (19-43)
530	Congo	Male	60 to 64	62 (44-86)	40 (35-46)	11 (9-14)	232 (174-297)	54 (37-72)
531	Congo	Male	65 to 69	58 (40-80)	59 (50-68)	16 (11-20)	338 (256-428)	87 (61-115)
532	Congo	Male	70 to <75	50 (35-71)	80 (67-94)	22 (18-27)	514 (397-642)	143 (101-191)
533	Costa Rica	Female	40 to 44	28 (21-37)	16 (13-18)	2 (1-3)	10 (9-12)	2 (1-2)
534	Costa Rica	Female	45 to 49	30 (22-40)	22 (19-25)	3 (2-5)	15 (14-17)	3 (2-3)
535	Costa Rica	Female	50 to 54	33 (25-43)	29 (26-33)	4 (3-6)	24 (21-27)	5 (4-6)
536	Costa Rica	Female	55 to 59	37 (26-49)	40 (36-45)	5 (4-7)	39 (35-43)	9 (7-10)
537	Costa Rica	Female	60 to 64	38 (27-50)	48 (43-53)	7 (5-9)	54 (49-60)	13 (11-16)
538	Costa Rica	Female	65 to 69	36 (26-50)	63 (57-69)	9 (7-11)	85 (76-94)	23 (19-26)
539	Costa Rica	Female	70 to <75	35 (25-49)	81 (74-88)	13 (10-15)	142 (127-158)	42 (36-49)
540	Costa Rica	Male	40 to 44	40 (30-51)	8 (7-10)	2 (1-2)	22 (19-24)	4 (3-5)
541	Costa Rica	Male	45 to 49	48 (35-64)	12 (11-14)	3 (2-4)	29 (25-32)	6 (5-7)
542	Costa Rica	Male	50 to 54	51 (38-66)	21 (19-24)	4 (4-5)	43 (38-49)	11 (9-13)
543	Costa Rica	Male	55 to 59	49 (36-65)	37 (33-40)	6 (4-8)	64 (57-72)	18 (15-21)
544	Costa Rica	Male	60 to 64	46 (33-64)	57 (52-63)	7 (6-9)	89 (79-98)	27 (23-31)
545	Costa Rica	Male	65 to 69	43 (30-61)	92 (84-102)	10 (7-12)	134 (121-148)	42 (37-48)
546	Costa Rica	Male	70 to <75	42 (29-59)	138 (123-154)	13 (11-16)	215 (193-237)	68 (60-78)
547	Cote d'Ivoire	Female	40 to 44	39 (29-51)	10 (9-12)	3 (2-3)	70 (61-82)	8 (6-11)
548	Cote d'Ivoire	Female	45 to 49	56 (38-78)	14 (12-17)	5 (3-6)	88 (73-106)	14 (10-19)
549	Cote d'Ivoire	Female	50 to 54	64 (47-84)	19 (17-22)	7 (5-8)	121 (98-145)	29 (21-39)
550	Cote d'Ivoire	Female	55 to 59	63 (43-91)	28 (22-32)	9 (7-12)	167 (137-199)	47 (35-63)
551	Cote d'Ivoire	Female	60 to 64	66 (44-93)	38 (30-45)	13 (10-16)	247 (207-286)	78 (61-99)
552	Cote d'Ivoire	Female	65 to 69	72 (49-101)	44 (33-51)	18 (13-23)	380 (320-431)	134 (105-165)
553	Cote d'Ivoire	Female	70 to <75	69 (47-97)	48 (37-54)	25 (21-31)	582 (507-653)	212 (170-257)
554	Cote d'Ivoire	Male	40 to 44	33 (23-43)	4 (3-4)	3 (3-4)	107 (92-121)	15 (10-20)
555	Cote d'Ivoire	Male	45 to 49	41 (28-57)	6 (5-7)	6 (4-8)	133 (115-154)	26 (18-34)
556	Cote d'Ivoire	Male	50 to 54	46 (34-63)	10 (9-11)	8 (7-10)	163 (143-188)	40 (30-51)

				Diabetes type 2	Cancers	Hemorrhagic stroke	All-cause mortality	CVD mortality
ID	Country	Sex	Age	Rate (95% CI)	Rate (95% CI)	Rate (95% CI)	Rate (95% CI)	Rate (95% CI)
557	Cote d'Ivoire	Male	55 to 59	47 (33-65)	17 (15-19)	11 (9-15)	210 (185-242)	58 (45-72)
558	Cote d'Ivoire	Male	60 to 64	49 (35-66)	29 (25-33)	15 (12-19)	294 (267-339)	92 (74-114)
559	Cote d'Ivoire	Male	65 to 69	50 (35-69)	48 (38-57)	20 (15-26)	421 (387-483)	128 (104-156)
560	Cote d'Ivoire	Male	70 to <75	46 (31-65)	71 (51-89)	27 (21-32)	634 (587-725)	205 (171-246)
561	Croatia	Female	40 to 44	33 (26-42)	28 (24-32)	3 (2-4)	10 (8-13)	2 (1-2)
562	Croatia	Female	45 to 49	33 (23-47)	45 (39-52)	5 (4-6)	19 (15-23)	3 (3-4)
563	Croatia	Female	50 to 54	36 (27-49)	62 (55-70)	6 (5-7)	31 (25-37)	6 (5-8)
564	Croatia	Female	55 to 59	43 (30-56)	78 (69-89)	7 (6-9)	47 (39-56)	12 (9-15)
565	Croatia	Female	60 to 64	45 (33-59)	97 (86-109)	10 (9-11)	70 (58-83)	21 (17-26)
566	Croatia	Female	65 to 69	43 (30-59)	123 (111-138)	14 (12-16)	122 (100-145)	46 (37-57)
567	Croatia	Female	70 to <75	36 (25-53)	139 (124-155)	22 (19-24)	219 (179-261)	102 (83-125)
568	Croatia	Male	40 to 44	52 (41-66)	16 (14-19)	4 (4-5)	23 (19-27)	6 (4-7)
569	Croatia	Male	45 to 49	52 (38-71)	31 (27-35)	6 (5-7)	42 (34-50)	12 (9-15)
570	Croatia	Male	50 to 54	53 (38-70)	56 (51-62)	9 (8-10)	73 (60-86)	22 (17-27)
571	Croatia	Male	55 to 59	55 (41-72)	95 (85-106)	12 (10-14)	116 (96-136)	36 (29-44)
572	Croatia	Male	60 to 64	51 (36-70)	143 (126-161)	16 (15-18)	173 (145-200)	60 (48-72)
573	Croatia	Male	65 to 69	41 (30-56)	209 (184-240)	22 (19-25)	272 (230-314)	102 (85-121)
574	Croatia	Male	70 to <75	31 (20-48)	267 (231-304)	32 (29-35)	420 (355-484)	176 (146-207)
575	Cuba	Female	40 to 44	28 (22-37)	21 (19-23)	2 (1-3)	12 (11-14)	2 (2-3)
576	Cuba	Female	45 to 49	32 (23-45)	33 (30-37)	4 (3-5)	20 (18-23)	5 (4-5)
577	Cuba	Female	50 to 54	37 (28-50)	46 (42-51)	5 (4-6)	34 (30-37)	8 (7-10)
578	Cuba	Female	55 to 59	41 (31-54)	64 (59-71)	7 (5-9)	56 (50-62)	15 (13-18)
579	Cuba	Female	60 to 64	39 (29-53)	82 (75-90)	10 (8-12)	93 (83-103)	29 (24-33)
580	Cuba	Female	65 to 69	33 (24-46)	97 (89-105)	14 (10-17)	142 (126-157)	50 (43-58)
581	Cuba	Female	70 to <75	27 (19-40)	108 (99-117)	18 (15-21)	209 (186-232)	82 (72-94)
582	Cuba	Male	40 to 44	40 (29-51)	9 (8-12)	2 (2-3)	21 (18-23)	4 (3-5)
583	Cuba	Male	45 to 49	53 (38-69)	18 (16-21)	4 (3-5)	33 (29-37)	8 (7-10)
584	Cuba	Male	50 to 54	56 (42-73)	35 (32-39)	6 (5-7)	55 (49-62)	16 (13-19)
585	Cuba	Male	55 to 59	50 (37-64)	62 (56-67)	8 (6-10)	87 (78-98)	28 (24-32)
586	Cuba	Male	60 to 64	43 (30-59)	101 (92-111)	11 (9-13)	141 (127-156)	50 (43-56)
587	Cuba	Male	65 to 69	37 (26-52)	150 (134-166)	15 (11-19)	217 (196-239)	80 (70-91)
588	Cuba	Male	70 to <75	32 (21-48)	193 (171-215)	19 (16-23)	316 (285-349)	122 (108-139)
589	Cyprus	Female	40 to 44	29 (22-38)	24 (20-29)	3 (2-3)	8 (7-8)	1 (1-1)
590	Cyprus	Female	45 to 49	33 (21-49)	36 (30-43)	4 (3-6)	12 (11-13)	2 (1-2)
591	Cyprus	Female	50 to 54	56 (41-73)	50 (42-59)	5 (4-7)	20 (19-22)	3 (3-4)
592	Cyprus	Female	55 to 59	99 (63-139)	64 (53-75)	6 (4-8)	32 (30-34)	6 (5-7)
593	Cyprus	Female	60 to 64	117 (90-151)	80 (66-94)	8 (6-10)	49 (46-53)	10 (8-12)
594	Cyprus	Female	65 to 69	110 (81-144)	101 (86-116)	11 (9-15)	90 (84-97)	24 (20-28)
595	Cyprus	Female	70 to <75	85 (48-125)	121 (104-140)	20 (17-25)	161 (149-173)	49 (43-57)
596	Cyprus	Male	40 to 44	40 (30-52)	14 (12-16)	3 (2-4)	16 (15-17)	4 (3-5)
597	Cyprus	Male	45 to 49	46 (29-68)	22 (19-26)	5 (4-7)	24 (22-26)	8 (6-9)
598	Cyprus	Male	50 to 54	77 (55-100)	38 (33-44)	7 (5-8)	36 (33-39)	13 (11-15)
599	Cyprus	Male	55 to 59	132 (86-181)	68 (60-77)	9 (7-12)	62 (57-67)	23 (20-25)
600	Cyprus	Male	60 to 64	145 (107-188)	116 (101-133)	13 (10-15)	100 (93-107)	36 (31-41)
601	Cyprus	Male	65 to 69	117 (87-155)	184 (162-209)	18 (14-23)	179 (166-191)	62 (54-70)
602	Cyprus	Male	70 to <75	82 (45-130)	251 (219-288)	28 (23-33)	285 (266-305)	100 (88-114)
603	Czech Republic	Female	40 to 44	43 (34-55)	24 (21-27)	3 (3-4)	9 (8-10)	1 (1-2)
604	Czech Republic	Female	45 to 49	42 (29-58)	38 (34-43)	5 (4-7)	16 (14-17)	2 (2-3)
605	Czech Republic	Female	50 to 54	47 (34-62)	52 (47-58)	6 (5-8)	26 (24-28)	5 (4-6)
606	Czech Republic	Female	55 to 59	56 (39-77)	73 (66-82)	7 (5-9)	42 (39-45)	9 (7-10)
607	Czech Republic	Female	60 to 64	59 (41-79)	102 (92-113)	9 (7-11)	68 (63-73)	17 (15-20)
608	Czech Republic	Female	65 to 69	56 (40-76)	133 (122-147)	12 (9-15)	111 (103-120)	36 (32-41)
609	Czech Republic	Female	70 to <75	47 (32-67)	156 (143-172)	17 (14-20)	182 (168-196)	73 (65-81)
610	Czech Republic	Male	40 to 44	68 (52-87)	15 (13-16)	4 (3-6)	19 (17-20)	4 (3-4)
611	Czech Republic	Male	45 to 49	64 (44-91)	26 (23-29)	6 (4-8)	32 (29-34)	8 (7-9)
612	Czech Republic	Male	50 to 54	65 (48-92)	48 (43-53)	8 (6-10)	54 (50-59)	15 (13-18)
613	Czech Republic	Male	55 to 59	71 (51-94)	89 (79-100)	10 (8-13)	93 (86-100)	29 (25-33)
614	Czech Republic	Male	60 to 64	67 (47-93)	156 (138-175)	14 (11-17)	153 (143-165)	53 (48-59)
615	Czech Republic	Male	65 to 69	54 (37-77)	241 (211-276)	18 (14-23)	234 (218-251)	89 (80-98)
616	Czech Republic	Male	70 to <75	42 (27-63)	311 (272-354)	24 (20-29)	339 (316-363)	139 (127-153)
617	Democratic Republic of the Congo	Female	40 to 44	34 (25-44)	13 (9-16)	2 (2-3)	63 (57-70)	5 (3-6)
618	Democratic Republic of the Congo	Female	45 to 49	41 (29-56)	16 (13-20)	4 (3-5)	83 (73-93)	11 (8-15)
619	Democratic Republic of the Congo	Female	50 to 54	48 (36-63)	21 (17-26)	6 (5-7)	120 (105-133)	18 (13-24)
620	Democratic Republic of the Congo	Female	55 to 59	55 (39-74)	27 (22-32)	9 (6-11)	172 (152-188)	37 (28-48)
621	Democratic Republic of the Congo	Female	60 to 64	57 (43-77)	32 (27-38)	13 (11-16)	256 (231-277)	69 (54-83)
622	Democratic Republic of the Congo	Female	65 to 69	55 (40-75)	40 (34-46)	20 (15-26)	393 (359-424)	128 (104-156)
623	Democratic Republic of the	Female	70 to <75	50 (34-69)	46 (40-53)	30 (25-36)	600 (555-646)	225 (188-267)

				Diabetes type 2	Cancers	Hemorrhagic stroke	All-cause mortality	CVD mortality
ID	Country	Sex	Age	Rate (95% CI)	Rate (95% CI)	Rate (95% CI)	Rate (95% CI)	Rate (95% CI)
	Congo							
624	Democratic Republic of the Congo	Male	40 to 44	43 (32-56)	5 (4-6)	2 (2-3)	80 (71-88)	8 (6-11)
625	Democratic Republic of the Congo	Male	45 to 49	62 (43-82)	8 (7-10)	4 (3-5)	105 (94-115)	14 (10-19)
626	Democratic Republic of the Congo	Male	50 to 54	69 (52-89)	14 (12-16)	6 (5-7)	138 (127-151)	23 (17-30)
627	Democratic Republic of the Congo	Male	55 to 59	66 (47-90)	22 (18-26)	8 (6-11)	188 (173-205)	37 (28-48)
628	Democratic Republic of the Congo	Male	60 to 64	62 (42-90)	34 (28-39)	12 (10-15)	271 (252-292)	65 (51-82)
629	Democratic Republic of the Congo	Male	65 to 69	58 (41-79)	49 (39-57)	18 (13-23)	395 (371-425)	101 (79-126)
630	Democratic Republic of the Congo	Male	70 to <75	50 (33-72)	67 (53-81)	25 (21-30)	601 (566-647)	162 (127-203)
631	Denmark	Female	40 to 44	27 (21-34)	26 (23-31)	2 (2-3)	8 (7-10)	1 (1-1)
632	Denmark	Female	45 to 49	30 (21-40)	43 (38-50)	4 (3-5)	15 (13-18)	1 (1-2)
633	Denmark	Female	50 to 54	37 (27-47)	63 (56-71)	5 (4-6)	27 (23-32)	3 (2-3)
634	Denmark	Female	55 to 59	49 (36-65)	86 (77-96)	5 (4-7)	43 (36-51)	5 (4-6)
635	Denmark	Female	60 to 64	55 (41-71)	118 (107-130)	7 (6-9)	68 (57-79)	9 (7-11)
636	Denmark	Female	65 to 69	55 (39-74)	149 (135-164)	10 (7-13)	104 (88-122)	16 (12-20)
637	Denmark	Female	70 to <75	48 (33-68)	174 (157-194)	17 (13-20)	170 (142-200)	33 (26-41)
638	Denmark	Male	40 to 44	40 (31-49)	16 (14-19)	2 (2-3)	14 (12-17)	2 (1-2)
639	Denmark	Male	45 to 49	49 (33-69)	26 (23-30)	4 (3-5)	24 (20-29)	4 (3-5)
640	Denmark	Male	50 to 54	60 (45-80)	49 (44-54)	6 (5-7)	43 (36-51)	8 (6-10)
641	Denmark	Male	55 to 59	74 (54-96)	86 (77-95)	8 (6-11)	69 (59-82)	13 (10-16)
642	Denmark	Male	60 to 64	78 (58-100)	144 (128-159)	11 (9-14)	107 (93-126)	23 (18-28)
643	Denmark	Male	65 to 69	72 (53-97)	219 (188-246)	15 (11-19)	162 (140-189)	37 (30-45)
644	Denmark	Male	70 to <75	60 (40-85)	288 (244-321)	23 (19-27)	259 (224-302)	66 (55-79)
645	Djibouti	Female	40 to 44	28 (21-36)	14 (10-22)	2 (1-2)	55 (44-70)	4 (2-6)
646	Djibouti	Female	45 to 49	33 (23-47)	19 (14-30)	3 (2-4)	66 (52-85)	8 (5-13)
647	Djibouti	Female	50 to 54	41 (31-56)	27 (19-39)	4 (3-5)	85 (64-110)	14 (7-22)
648	Djibouti	Female	55 to 59	51 (34-72)	29 (22-41)	6 (4-8)	114 (80-150)	25 (14-38)
649	Djibouti	Female	60 to 64	59 (42-80)	38 (29-53)	9 (7-11)	168 (117-216)	44 (26-64)
650	Djibouti	Female	65 to 69	64 (45-89)	43 (33-57)	14 (10-18)	257 (177-330)	79 (49-112)
651	Djibouti	Female	70 to <75	62 (43-87)	45 (35-58)	20 (16-24)	405 (289-502)	141 (92-194)
652	Djibouti	Male	40 to 44	37 (27-48)	7 (6-9)	2 (2-3)	72 (60-88)	9 (6-13)
653	Djibouti	Male	45 to 49	58 (41-81)	11 (9-13)	4 (3-5)	91 (75-111)	15 (11-22)
654	Djibouti	Male	50 to 54	68 (51-90)	16 (13-19)	6 (4-7)	117 (96-141)	24 (17-33)
655	Djibouti	Male	55 to 59	68 (48-96)	26 (21-30)	7 (5-10)	156 (130-183)	37 (27-49)
656	Djibouti	Male	60 to 64	67 (46-93)	41 (31-51)	11 (9-14)	227 (192-261)	64 (48-84)
657	Djibouti	Male	65 to 69	65 (45-91)	60 (43-73)	16 (12-21)	336 (281-387)	101 (78-130)
658	Djibouti	Male	70 to <75	60 (41-87)	85 (59-107)	22 (18-27)	513 (433-597)	163 (128-210)
659	Dominica	Female	40 to 44	66 (47-86)	22 (19-26)	2 (2-3)	20 (17-25)	4 (3-5)
660	Dominica	Female	45 to 49	90 (57-133)	32 (28-37)	4 (3-5)	31 (25-37)	7 (6-9)
661	Dominica	Female	50 to 54	127 (89-170)	39 (35-44)	5 (4-7)	47 (39-57)	12 (10-16)
662	Dominica	Female	55 to 59	178 (111-243)	44 (40-50)	7 (5-10)	64 (52-76)	17 (13-21)
663	Dominica	Female	60 to 64	179 (124-241)	64 (58-71)	10 (8-13)	108 (90-127)	35 (28-41)
664	Dominica	Female	65 to 69	130 (94-188)	76 (69-83)	15 (11-19)	164 (139-196)	58 (48-72)
665	Dominica	Female	70 to <75	91 (47-164)	99 (91-107)	21 (17-25)	295 (253-358)	119 (99-146)
666	Dominica	Male	40 to 44	87 (65-111)	14 (12-18)	2 (2-3)	43 (36-52)	8 (6-10)
667	Dominica	Male	45 to 49	126 (91-176)	24 (21-28)	4 (3-5)	59 (49-70)	13 (10-17)
668	Dominica	Male	50 to 54	135 (103-180)	42 (37-47)	6 (5-8)	85 (71-101)	23 (18-28)
669	Dominica	Male	55 to 59	113 (85-154)	68 (60-77)	9 (7-12)	121 (102-142)	37 (30-45)
670	Dominica	Male	60 to 64	90 (54-134)	116 (100-135)	13 (10-15)	183 (155-214)	59 (49-72)
671	Dominica	Male	65 to 69	68 (45-97)	179 (153-211)	17 (13-22)	275 (232-321)	92 (76-110)
672	Dominica	Male	70 to <75	54 (35-81)	287 (237-339)	24 (20-28)	453 (379-526)	153 (125-181)
673	Dominican Republic	Female	40 to 44	32 (24-40)	11 (9-13)	2 (2-3)	19 (16-22)	3 (2-4)
674	Dominican Republic	Female	45 to 49	39 (28-52)	15 (13-18)	4 (3-5)	25 (21-31)	6 (4-8)
675	Dominican Republic	Female	50 to 54	45 (35-59)	20 (18-23)	5 (4-7)	33 (27-42)	10 (7-13)
676	Dominican Republic	Female	55 to 59	51 (37-66)	25 (23-28)	7 (5-10)	47 (38-61)	16 (12-22)
677	Dominican Republic	Female	60 to 64	50 (36-67)	33 (30-37)	11 (8-13)	76 (58-96)	31 (23-41)
678	Dominican Republic	Female	65 to 69	42 (31-57)	43 (39-47)	15 (11-20)	122 (93-150)	55 (40-70)
679	Dominican Republic	Female	70 to <75	36 (25-50)	54 (49-60)	21 (17-26)	206 (156-248)	101 (74-125)
680	Dominican Republic	Male	40 to 44	44 (34-56)	5 (4-6)	2 (2-3)	40 (33-48)	6 (4-8)
681	Dominican Republic	Male	45 to 49	58 (42-76)	9 (7-11)	4 (3-6)	52 (42-62)	11 (8-15)
682	Dominican Republic	Male	50 to 54	61 (45-79)	16 (14-19)	6 (5-8)	65 (53-77)	20 (15-26)
683	Dominican Republic	Male	55 to 59	54 (39-72)	27 (23-31)	9 (7-12)	86 (70-104)	32 (24-40)
684	Dominican Republic	Male	60 to 64	47 (32-67)	46 (41-54)	13 (10-16)	128 (103-153)	54 (41-65)
685	Dominican Republic	Male	65 to 69	40 (28-58)	73 (64-84)	18 (13-23)	193 (152-229)	86 (65-103)
686	Dominican Republic	Male	70 to <75	35 (25-52)	109 (94-127)	24 (20-29)	298 (245-350)	136 (110-162)
687	Ecuador	Female	40 to 44	39 (30-51)	14 (12-15)	2 (2-3)	16 (14-18)	2 (2-3)
688	Ecuador	Female	45 to 49	49 (34-67)	20 (18-22)	4 (3-5)	23 (20-25)	4 (3-5)

				Diabetes type 2	Cancers	Hemorrhagic stroke	All-cause mortality	CVD mortality
**ID**	**Country**	**Sex**	**Age**	**Rate (95% CI)**	**Rate (95% CI)**	**Rate (95% CI)**	**Rate (95% CI)**	**Rate (95% CI)**
689	Ecuador	Female	50 to 54	55 (40-72)	27 (25-29)	5 (4-6)	34 (31-38)	7 (5-8)
690	Ecuador	Female	55 to 59	59 (41-78)	34 (31-37)	6 (5-8)	49 (44-54)	10 (8-12)
691	Ecuador	Female	60 to 64	57 (40-78)	44 (41-47)	8 (7-10)	75 (68-83)	16 (13-19)
692	Ecuador	Female	65 to 69	49 (34-71)	56 (52-60)	11 (8-14)	111 (101-123)	25 (21-30)
693	Ecuador	Female	70 to <75	44 (28-67)	70 (65-76)	14 (11-17)	174 (157-192)	44 (38-51)
694	Ecuador	Male	40 to 44	43 (34-53)	6 (5-6)	2 (1-2)	30 (27-34)	4 (3-5)
695	Ecuador	Male	45 to 49	56 (41-76)	9 (8-10)	3 (2-4)	39 (35-44)	7 (5-8)
696	Ecuador	Male	50 to 54	63 (47-81)	15 (14-16)	5 (4-6)	54 (48-60)	11 (9-13)
697	Ecuador	Male	55 to 59	64 (46-86)	25 (23-28)	6 (4-8)	75 (67-84)	17 (14-20)
698	Ecuador	Male	60 to 64	61 (41-83)	42 (38-46)	8 (6-10)	110 (100-122)	28 (24-32)
699	Ecuador	Male	65 to 69	51 (36-72)	66 (60-73)	11 (8-14)	163 (148-180)	43 (37-49)
700	Ecuador	Male	70 to <75	45 (30-65)	101 (92-114)	14 (12-17)	259 (236-286)	70 (61-80)
701	Egypt	Female	40 to 44	81 (60-106)	10 (8-12)	3 (2-3)	18 (14-22)	7 (5-8)
702	Egypt	Female	45 to 49	96 (68-128)	14 (12-20)	4 (3-6)	32 (26-39)	14 (11-18)
703	Egypt	Female	50 to 54	97 (68-130)	20 (16-28)	6 (5-8)	63 (52-74)	30 (24-37)
704	Egypt	Female	55 to 59	86 (61-114)	25 (19-37)	8 (6-12)	108 (90-128)	56 (44-68)
705	Egypt	Female	60 to 64	70 (47-99)	29 (24-38)	12 (9-15)	165 (138-193)	89 (74-106)
706	Egypt	Female	65 to 69	50 (36-69)	31 (26-42)	17 (12-21)	238 (198-279)	135 (111-163)
707	Egypt	Female	70 to <75	38 (25-55)	36 (29-50)	22 (18-28)	379 (315-446)	216 (177-259)
708	Egypt	Male	40 to 44	86 (63-113)	8 (7-9)	2 (2-3)	32 (26-39)	9 (7-12)
709	Egypt	Male	45 to 49	102 (72-138)	14 (12-17)	4 (3-6)	62 (50-74)	22 (16-28)
710	Egypt	Male	50 to 54	103 (69-138)	26 (21-31)	7 (5-8)	113 (92-134)	45 (35-57)
711	Egypt	Male	55 to 59	89 (64-122)	35 (28-46)	10 (8-13)	187 (155-219)	81 (64-101)
712	Egypt	Male	60 to 64	76 (50-111)	47 (39-57)	14 (11-17)	262 (221-304)	120 (97-143)
713	Egypt	Male	65 to 69	63 (45-86)	52 (44-64)	18 (14-23)	362 (307-418)	172 (141-205)
714	Egypt	Male	70 to <75	50 (34-73)	64 (53-80)	24 (20-29)	535 (455-615)	250 (207-297)
715	El Salvador	Female	40 to 44	52 (39-68)	17 (14-20)	2 (1-3)	19 (16-22)	3 (2-4)
716	El Salvador	Female	45 to 49	56 (40-75)	24 (20-29)	3 (2-4)	28 (24-33)	5 (4-6)
717	El Salvador	Female	50 to 54	59 (43-78)	30 (25-35)	5 (4-6)	41 (36-48)	8 (6-10)
718	El Salvador	Female	55 to 59	62 (45-81)	36 (30-43)	6 (4-8)	60 (52-70)	12 (9-15)
719	El Salvador	Female	60 to 64	59 (42-80)	43 (37-50)	8 (6-10)	90 (78-104)	21 (17-26)
720	El Salvador	Female	65 to 69	52 (37-75)	51 (43-58)	10 (7-13)	130 (112-151)	33 (27-41)
721	El Salvador	Female	70 to <75	47 (32-69)	61 (52-73)	13 (11-16)	200 (171-233)	58 (47-70)
722	El Salvador	Male	40 to 44	62 (46-81)	7 (6-9)	2 (1-2)	55 (46-64)	5 (4-7)
723	El Salvador	Male	45 to 49	75 (54-101)	11 (9-14)	3 (2-4)	65 (54-77)	8 (6-11)
724	El Salvador	Male	50 to 54	77 (56-104)	17 (14-21)	4 (3-5)	81 (68-95)	13 (10-17)
725	El Salvador	Male	55 to 59	71 (52-98)	26 (21-32)	6 (4-8)	107 (91-125)	21 (16-27)
726	El Salvador	Male	60 to 64	63 (43-90)	41 (35-49)	7 (6-9)	143 (122-164)	32 (26-39)
727	El Salvador	Male	65 to 69	53 (38-78)	59 (49-71)	10 (7-12)	187 (160-214)	47 (38-57)
728	El Salvador	Male	70 to <75	50 (34-75)	81 (67-100)	13 (10-16)	264 (227-304)	73 (61-87)
729	Equatorial Guinea	Female	40 to 44	50 (36-69)	18 (14-23)	2 (1-2)	76 (58-99)	3 (1-5)
730	Equatorial Guinea	Female	45 to 49	60 (41-86)	23 (19-29)	3 (2-4)	84 (57-115)	7 (3-11)
731	Equatorial Guinea	Female	50 to 54	71 (51-96)	29 (24-35)	4 (3-6)	99 (65-144)	11 (5-19)
732	Equatorial Guinea	Female	55 to 59	82 (56-112)	35 (29-42)	6 (4-8)	119 (71-183)	20 (9-34)
733	Equatorial Guinea	Female	60 to 64	84 (57-119)	43 (36-52)	9 (7-12)	167 (95-255)	36 (18-61)
734	Equatorial Guinea	Female	65 to 69	78 (55-111)	52 (45-62)	14 (10-19)	248 (139-379)	67 (34-109)
735	Equatorial Guinea	Female	70 to <75	67 (45-99)	61 (52-71)	21 (16-25)	385 (219-571)	130 (71-202)
736	Equatorial Guinea	Male	40 to 44	58 (43-77)	6 (5-7)	2 (1-2)	87 (64-111)	4 (2-6)
737	Equatorial Guinea	Male	45 to 49	87 (60-121)	10 (9-13)	3 (2-4)	99 (71-134)	7 (4-12)
738	Equatorial Guinea	Male	50 to 54	100 (74-133)	18 (16-21)	5 (4-6)	112 (79-155)	12 (7-21)
739	Equatorial Guinea	Male	55 to 59	96 (66-133)	29 (25-34)	7 (5-9)	132 (88-191)	21 (12-35)
740	Equatorial Guinea	Male	60 to 64	88 (57-129)	47 (39-55)	10 (7-12)	186 (125-263)	38 (22-59)
741	Equatorial Guinea	Male	65 to 69	78 (55-109)	69 (56-83)	14 (10-19)	268 (177-377)	62 (38-95)
742	Equatorial Guinea	Male	70 to <75	64 (42-95)	99 (80-119)	21 (16-25)	412 (274-567)	107 (65-157)
743	Eritrea	Female	40 to 44	29 (22-38)	19 (15-24)	2 (2-3)	65 (53-77)	7 (5-10)
744	Eritrea	Female	45 to 49	34 (24-47)	26 (20-31)	3 (2-5)	90 (74-105)	14 (9-19)
745	Eritrea	Female	50 to 54	42 (32-55)	34 (27-41)	5 (4-6)	129 (109-149)	23 (16-31)
746	Eritrea	Female	55 to 59	52 (35-73)	37 (30-44)	7 (5-9)	184 (155-211)	40 (29-53)
747	Eritrea	Female	60 to 64	60 (42-80)	46 (38-54)	11 (9-14)	266 (230-301)	70 (52-88)
748	Eritrea	Female	65 to 69	65 (47-90)	52 (44-60)	17 (13-22)	404 (350-456)	122 (93-153)
749	Eritrea	Female	70 to <75	62 (44-85)	53 (46-61)	26 (21-30)	627 (538-710)	215 (168-263)
750	Eritrea	Male	40 to 44	31 (23-39)	7 (6-8)	2 (2-3)	72 (59-85)	9 (6-13)
751	Eritrea	Male	45 to 49	49 (34-65)	10 (9-12)	4 (3-5)	97 (82-115)	16 (11-22)
752	Eritrea	Male	50 to 54	57 (43-73)	16 (14-18)	5 (4-7)	133 (113-162)	24 (17-34)
753	Eritrea	Male	55 to 59	57 (41-79)	24 (20-28)	7 (5-10)	184 (156-225)	37 (28-52)
754	Eritrea	Male	60 to 64	58 (41-80)	40 (31-48)	11 (9-14)	270 (233-331)	66 (49-90)
755	Eritrea	Male	65 to 69	58 (41-80)	58 (43-72)	16 (12-21)	399 (346-478)	107 (82-138)
756	Eritrea	Male	70 to <75	54 (37-74)	85 (61-108)	23 (19-28)	610 (528-715)	172 (134-223)
757	Estonia	Female	40 to 44	46 (37-58)	24 (21-28)	3 (2-4)	12 (10-15)	2 (1-2)
758	Estonia	Female	45 to 49	43 (30-59)	38 (33-46)	4 (3-6)	20 (16-24)	3 (2-4)

				Diabetes type 2	Cancers	Hemorrhagic stroke	All-cause mortality	CVD mortality
ID	Country	Sex	Age	Rate (95% CI)	Rate (95% CI)	Rate (95% CI)	Rate (95% CI)	Rate (95% CI)
759	Estonia	Female	50 to 54	42 (31-58)	54 (47-62)	6 (4-7)	31 (25-36)	6 (5-8)
760	Estonia	Female	55 to 59	45 (32-62)	71 (62-83)	7 (5-10)	48 (39-55)	13 (10-15)
761	Estonia	Female	60 to 64	43 (30-61)	92 (80-105)	10 (7-12)	71 (58-81)	24 (19-28)
762	Estonia	Female	65 to 69	35 (25-50)	114 (101-131)	13 (9-17)	109 (88-123)	45 (36-52)
763	Estonia	Female	70 to <75	28 (18-44)	132 (116-152)	16 (13-21)	177 (142-199)	88 (71-101)
764	Estonia	Male	40 to 44	61 (49-78)	11 (10-14)	4 (3-5)	37 (30-45)	7 (5-9)
765	Estonia	Male	45 to 49	54 (40-74)	24 (20-28)	6 (4-8)	59 (48-69)	16 (12-19)
766	Estonia	Male	50 to 54	49 (34-70)	47 (41-54)	9 (7-10)	88 (73-102)	27 (22-34)
767	Estonia	Male	55 to 59	47 (33-63)	88 (74-101)	11 (8-15)	136 (114-156)	49 (40-58)
768	Estonia	Male	60 to 64	42 (29-59)	148 (122-170)	15 (11-18)	205 (175-233)	85 (71-99)
769	Estonia	Male	65 to 69	35 (25-52)	221 (177-260)	18 (13-23)	287 (247-325)	130 (109-149)
770	Estonia	Male	70 to <75	29 (19-43)	286 (225-337)	21 (16-26)	398 (346-448)	196 (168-225)
771	Ethiopia	Female	40 to 44	28 (21-36)	16 (13-23)	2 (2-2)	56 (42-71)	7 (4-9)
772	Ethiopia	Female	45 to 49	33 (23-47)	22 (17-30)	3 (2-4)	78 (59-98)	13 (9-17)
773	Ethiopia	Female	50 to 54	41 (30-54)	30 (24-40)	5 (4-6)	114 (88-139)	23 (16-30)
774	Ethiopia	Female	55 to 59	50 (35-67)	32 (26-41)	7 (5-10)	161 (129-196)	40 (30-51)
775	Ethiopia	Female	60 to 64	58 (41-77)	42 (35-53)	11 (9-14)	235 (193-278)	67 (52-84)
776	Ethiopia	Female	65 to 69	63 (45-87)	46 (40-58)	17 (13-22)	358 (305-421)	113 (90-142)
777	Ethiopia	Female	70 to <75	60 (42-83)	50 (42-60)	26 (21-31)	555 (469-658)	197 (156-238)
778	Ethiopia	Male	40 to 44	31 (23-39)	7 (5-8)	2 (1-2)	64 (49-79)	8 (6-12)
779	Ethiopia	Male	45 to 49	48 (33-65)	9 (8-11)	4 (3-5)	87 (67-109)	15 (10-20)
780	Ethiopia	Male	50 to 54	57 (43-73)	15 (12-17)	5 (4-7)	120 (92-152)	24 (17-31)
781	Ethiopia	Male	55 to 59	58 (41-80)	23 (19-27)	7 (5-10)	166 (130-211)	37 (27-50)
782	Ethiopia	Male	60 to 64	58 (41-80)	37 (28-44)	11 (9-14)	244 (196-310)	66 (50-86)
783	Ethiopia	Male	65 to 69	57 (39-77)	54 (40-66)	17 (13-22)	364 (291-451)	106 (82-136)
784	Ethiopia	Male	70 to <75	53 (36-73)	80 (56-101)	24 (20-29)	559 (448-677)	178 (138-222)
785	Federated States of Micronesia	Female	40 to 44	205 (155-258)	16 (11-21)	4 (3-5)	61 (45-81)	13 (2-24)
786	Federated States of Micronesia	Female	45 to 49	330 (227-444)	25 (16-34)	7 (5-10)	86 (65-112)	29 (20-41)
787	Federated States of Micronesia	Female	50 to 54	393 (307-502)	29 (20-38)	11 (9-13)	125 (96-160)	42 (29-57)
788	Federated States of Micronesia	Female	55 to 59	394 (302-507)	35 (24-45)	15 (11-20)	180 (140-229)	66 (48-87)
789	Federated States of Micronesia	Female	60 to 64	318 (214-451)	39 (30-48)	20 (16-25)	257 (202-323)	102 (76-133)
790	Federated States of Micronesia	Female	65 to 69	165 (125-224)	50 (39-59)	26 (19-34)	381 (300-477)	159 (120-207)
791	Federated States of Micronesia	Female	70 to <75	73 (45-119)	63 (51-74)	40 (33-48)	604 (476-754)	264 (200-340)
792	Federated States of Micronesia	Male	40 to 44	230 (170-300)	8 (6-9)	4 (3-5)	89 (67-115)	31 (7-43)
793	Federated States of Micronesia	Male	45 to 49	350 (246-470)	12 (10-15)	8 (6-11)	128 (94-167)	54 (38-72)
794	Federated States of Micronesia	Male	50 to 54	372 (277-475)	17 (14-21)	12 (10-15)	180 (136-234)	82 (60-110)
795	Federated States of Micronesia	Male	55 to 59	293 (219-380)	25 (21-30)	17 (13-22)	247 (192-315)	111 (84-143)
796	Federated States of Micronesia	Male	60 to 64	211 (135-319)	36 (31-42)	23 (18-28)	345 (280-429)	151 (115-194)
797	Federated States of Micronesia	Male	65 to 69	124 (89-172)	57 (50-65)	30 (22-38)	476 (400-577)	198 (160-244)
798	Federated States of Micronesia	Male	70 to <75	68 (42-106)	81 (70-93)	41 (33-49)	673 (583-796)	275 (229-334)
799	Fiji	Female	40 to 44	393 (327-477)	39 (25-51)	3 (3-4)	47 (30-72)	13 (7-20)
800	Fiji	Female	45 to 49	745 (645-811)	44 (30-54)	6 (4-8)	69 (45-102)	19 (11-29)
801	Fiji	Female	50 to 54	928 (828-990)	52 (37-62)	9 (7-11)	109 (73-155)	28 (18-41)
802	Fiji	Female	55 to 59	942 (852-991)	63 (46-75)	12 (9-17)	167 (114-233)	46 (31-66)
803	Fiji	Female	60 to 64	741 (654-786)	66 (49-78)	16 (13-21)	257 (183-348)	79 (55-111)
804	Fiji	Female	65 to 69	327 (283-386)	75 (59-87)	22 (15-28)	355 (252-479)	112 (76-155)
805	Fiji	Female	70 to <75	96 (57-152)	85 (67-97)	32 (26-39)	554 (396-750)	186 (130-253)
806	Fiji	Male	40 to 44	379 (297-491)	7 (7-8)	3 (2-4)	66 (39-99)	27 (17-41)
807	Fiji	Male	45 to 49	717 (569-807)	13 (11-14)	6 (4-8)	101 (61-151)	44 (27-67)
808	Fiji	Male	50 to 54	877 (743-970)	18 (17-21)	9 (7-11)	158 (101-230)	70 (44-105)
809	Fiji	Male	55 to 59	858 (690-965)	28 (24-32)	12 (9-16)	234 (158-327)	98 (65-138)
810	Fiji	Male	60 to 64	662 (484-772)	44 (37-50)	17 (13-21)	343 (242-459)	138 (95-188)
811	Fiji	Male	65 to 69	288 (219-353)	70 (54-85)	22 (16-29)	470 (340-613)	175 (124-235)
812	Fiji	Male	70 to <75	84 (50-138)	87 (67-107)	30 (24-37)	722 (535-911)	271 (191-349)
813	Finland	Female	40 to 44	32 (25-40)	23 (19-27)	3 (2-4)	7 (6-8)	1 (1-1)
814	Finland	Female	45 to 49	32 (22-43)	39 (33-46)	5 (4-7)	13 (11-14)	2 (1-2)
815	Finland	Female	50 to 54	38 (28-50)	54 (47-63)	7 (5-8)	21 (19-23)	3 (2-4)
816	Finland	Female	55 to 59	50 (37-65)	70 (61-82)	7 (5-10)	31 (28-35)	5 (4-6)
817	Finland	Female	60 to 64	55 (42-72)	96 (85-108)	9 (7-12)	48 (43-53)	10 (8-12)
818	Finland	Female	65 to 69	54 (39-73)	120 (107-133)	13 (9-16)	78 (69-85)	20 (16-23)
819	Finland	Female	70 to <75	48 (34-67)	131 (114-151)	19 (15-23)	118 (104-130)	35 (30-41)
820	Finland	Male	40 to 44	43 (34-54)	13 (10-16)	3 (3-4)	17 (15-18)	3 (2-3)
821	Finland	Male	45 to 49	48 (34-63)	22 (18-28)	5 (4-7)	27 (24-30)	6 (5-7)
822	Finland	Male	50 to 54	57 (43-73)	40 (34-47)	7 (6-9)	42 (37-46)	11 (9-13)
823	Finland	Male	55 to 59	69 (51-91)	76 (65-88)	10 (7-13)	69 (63-77)	22 (19-25)
824	Finland	Male	60 to 64	74 (55-95)	131 (114-150)	13 (11-17)	108 (98-119)	38 (33-43)
825	Finland	Male	65 to 69	72 (53-99)	199 (170-229)	18 (14-23)	161 (147-176)	60 (53-68)
826	Finland	Male	70 to <75	61 (42-86)	260 (218-302)	26 (21-31)	241 (220-264)	94 (84-107)
827	France	Female	40 to 44	18 (14-23)	27 (23-31)	2 (1-3)	10 (9-10)	1 (1-1)
828	France	Female	45 to 49	19 (13-29)	41 (36-48)	3 (2-5)	16 (15-17)	2 (1-2)

				Diabetes type 2	Cancers	Hemorrhagic stroke	All-cause mortality	CVD mortality
ID	Country	Sex	Age	Rate (95% CI)	Rate (95% CI)	Rate (95% CI)	Rate (95% CI)	Rate (95% CI)
829	France	Female	50 to 54	26 (20-35)	55 (49-62)	4 (3-6)	25 (23-26)	3 (2-3)
830	France	Female	55 to 59	38 (27-52)	70 (62-78)	5 (3-7)	35 (32-37)	4 (3-5)
831	France	Female	60 to 64	42 (32-56)	86 (77-97)	6 (5-8)	48 (44-51)	6 (5-8)
832	France	Female	65 to 69	39 (29-52)	101 (90-113)	8 (6-11)	67 (62-72)	11 (9-13)
833	France	Female	70 to <75	32 (22-46)	114 (102-130)	13 (10-15)	104 (96-112)	22 (19-25)
834	France	Male	40 to 44	27 (21-35)	17 (15-20)	2 (2-3)	18 (17-20)	3 (2-3)
835	France	Male	45 to 49	34 (22-50)	32 (28-38)	4 (3-5)	31 (29-33)	5 (4-6)
836	France	Male	50 to 54	45 (33-60)	60 (54-68)	5 (4-6)	50 (47-54)	8 (7-9)
837	France	Male	55 to 59	59 (41-82)	102 (91-116)	7 (5-9)	77 (72-83)	13 (11-15)
838	France	Male	60 to 64	62 (43-84)	153 (137-173)	9 (7-11)	109 (102-116)	20 (17-23)
839	France	Male	65 to 69	52 (37-70)	209 (183-237)	12 (9-16)	150 (141-159)	30 (26-34)
840	France	Male	70 to <75	39 (25-58)	260 (226-295)	19 (15-22)	218 (205-231)	49 (43-56)
841	Gabon	Female	40 to 44	55 (40-74)	17 (13-22)	2 (2-3)	50 (38-66)	5 (3-7)
842	Gabon	Female	45 to 49	67 (45-94)	23 (18-29)	4 (3-5)	68 (51-88)	10 (7-14)
843	Gabon	Female	50 to 54	79 (55-105)	30 (23-37)	5 (4-6)	97 (71-123)	16 (11-23)
844	Gabon	Female	55 to 59	91 (62-126)	36 (30-44)	7 (5-10)	136 (99-170)	30 (21-41)
845	Gabon	Female	60 to 64	93 (65-131)	43 (37-52)	11 (9-14)	200 (151-244)	53 (38-70)
846	Gabon	Female	65 to 69	84 (59-118)	52 (45-61)	17 (12-22)	308 (228-368)	100 (72-123)
847	Gabon	Female	70 to <75	71 (45-105)	59 (52-69)	24 (20-29)	477 (364-572)	185 (141-230)
848	Gabon	Male	40 to 44	58 (41-77)	6 (5-7)	2 (1-2)	79 (61-101)	5 (3-8)
849	Gabon	Male	45 to 49	85 (58-117)	11 (9-13)	3 (2-4)	98 (74-129)	10 (6-15)
850	Gabon	Male	50 to 54	96 (72-129)	18 (16-21)	5 (4-6)	123 (93-163)	16 (10-25)
851	Gabon	Male	55 to 59	91 (65-127)	29 (26-33)	7 (5-10)	153 (111-202)	27 (17-40)
852	Gabon	Male	60 to 64	84 (59-120)	46 (39-52)	11 (9-14)	218 (160-280)	48 (33-66)
853	Gabon	Male	65 to 69	75 (54-105)	66 (57-76)	16 (12-20)	317 (233-407)	80 (56-111)
854	Gabon	Male	70 to <75	62 (40-92)	89 (75-104)	22 (18-27)	483 (366-621)	135 (97-189)
855	Georgia	Female	40 to 44	32 (25-39)	22 (19-26)	3 (2-4)	14 (10-19)	3 (2-4)
856	Georgia	Female	45 to 49	36 (26-50)	31 (27-37)	6 (4-7)	22 (16-30)	6 (4-9)
857	Georgia	Female	50 to 54	39 (29-52)	41 (35-47)	9 (8-11)	35 (24-47)	11 (8-16)
858	Georgia	Female	55 to 59	41 (30-55)	50 (42-59)	14 (11-18)	55 (38-73)	22 (15-30)
859	Georgia	Female	60 to 64	38 (26-52)	58 (50-69)	20 (16-25)	91 (64-117)	43 (30-57)
860	Georgia	Female	65 to 69	30 (22-43)	64 (56-75)	28 (21-35)	146 (104-188)	82 (58-104)
861	Georgia	Female	70 to <75	23 (14-35)	66 (57-80)	37 (31-44)	243 (179-305)	156 (114-194)
862	Georgia	Male	40 to 44	47 (37-59)	12 (11-15)	7 (5-8)	47 (34-67)	16 (11-23)
863	Georgia	Male	45 to 49	50 (35-69)	22 (19-28)	11 (9-15)	71 (51-99)	28 (20-39)
864	Georgia	Male	50 to 54	48 (35-66)	40 (35-48)	17 (14-20)	106 (78-143)	46 (33-64)
865	Georgia	Male	55 to 59	44 (31-59)	69 (59-83)	24 (18-30)	157 (118-204)	75 (56-97)
866	Georgia	Male	60 to 64	40 (26-55)	103 (87-127)	31 (25-37)	238 (184-297)	123 (95-153)
867	Georgia	Male	65 to 69	35 (25-51)	133 (110-167)	38 (29-47)	339 (268-414)	191 (149-234)
868	Georgia	Male	70 to <75	28 (18-43)	166 (133-218)	52 (43-62)	529 (431-628)	328 (268-390)
869	Germany	Female	40 to 44	35 (26-44)	26 (23-30)	3 (2-3)	8 (7-10)	1 (1-1)
870	Germany	Female	45 to 49	37 (24-54)	41 (36-46)	4 (3-6)	15 (13-16)	2 (2-2)
871	Germany	Female	50 to 54	47 (34-64)	59 (54-66)	6 (4-7)	25 (22-28)	4 (3-4)
872	Germany	Female	55 to 59	64 (44-85)	78 (70-87)	6 (4-8)	39 (34-43)	6 (5-8)
873	Germany	Female	60 to 64	68 (49-89)	105 (96-116)	7 (6-10)	60 (53-67)	12 (10-14)
874	Germany	Female	65 to 69	60 (44-80)	126 (115-139)	9 (7-12)	88 (78-98)	20 (17-24)
875	Germany	Female	70 to <75	46 (31-68)	145 (132-159)	15 (12-18)	141 (124-158)	42 (35-48)
876	Germany	Male	40 to 44	44 (33-54)	16 (14-18)	3 (2-4)	15 (13-17)	3 (2-3)
877	Germany	Male	45 to 49	63 (40-83)	28 (25-31)	5 (3-6)	26 (23-29)	6 (5-7)
878	Germany	Male	50 to 54	75 (57-91)	53 (48-58)	6 (5-8)	46 (41-52)	11 (9-13)
879	Germany	Male	55 to 59	79 (60-105)	95 (86-104)	8 (6-10)	74 (66-83)	19 (16-23)
880	Germany	Male	60 to 64	76 (56-102)	158 (142-175)	10 (8-13)	117 (105-130)	32 (28-38)
881	Germany	Male	65 to 69	63 (46-84)	225 (199-254)	15 (11-18)	168 (151-186)	51 (44-57)
882	Germany	Male	70 to <75	47 (31-69)	291 (255-328)	22 (18-26)	266 (239-295)	89 (77-101)
883	Ghana	Female	40 to 44	37 (27-48)	12 (10-15)	3 (2-3)	53 (46-62)	8 (6-11)
884	Ghana	Female	45 to 49	48 (33-67)	17 (14-20)	5 (4-7)	68 (57-82)	17 (13-21)
885	Ghana	Female	50 to 54	54 (40-72)	21 (18-25)	8 (6-9)	94 (76-112)	31 (23-39)
886	Ghana	Female	55 to 59	55 (39-78)	26 (22-31)	10 (8-13)	132 (103-155)	49 (37-61)
887	Ghana	Female	60 to 64	57 (39-80)	35 (30-40)	14 (11-17)	196 (156-226)	79 (61-96)
888	Ghana	Female	65 to 69	58 (40-83)	39 (35-44)	19 (14-24)	304 (238-344)	135 (104-160)
889	Ghana	Female	70 to <75	56 (38-77)	42 (38-47)	26 (21-31)	473 (384-524)	210 (167-247)
890	Ghana	Male	40 to 44	41 (29-53)	5 (4-6)	2 (2-3)	67 (58-79)	7 (5-10)
891	Ghana	Male	45 to 49	58 (40-79)	7 (6-8)	4 (3-6)	88 (74-103)	12 (9-16)
892	Ghana	Male	50 to 54	67 (49-89)	11 (10-13)	6 (5-8)	116 (100-135)	19 (15-25)
893	Ghana	Male	55 to 59	69 (46-99)	18 (15-21)	8 (6-11)	156 (136-179)	28 (22-35)
894	Ghana	Male	60 to 64	69 (45-96)	27 (24-32)	11 (9-14)	229 (203-258)	50 (39-60)
895	Ghana	Male	65 to 69	69 (48-96)	43 (38-50)	14 (11-18)	337 (299-378)	73 (59-89)
896	Ghana	Male	70 to <75	62 (41-88)	61 (53-72)	19 (15-22)	513 (461-576)	117 (96-141)
897	Greece	Female	40 to 44	19 (14-25)	22 (19-26)	3 (2-3)	8 (7-9)	2 (1-2)
898	Greece	Female	45 to 49	20 (13-30)	33 (29-39)	4 (3-6)	13 (12-15)	3 (2-4)

				Diabetes type 2	Cancers	Hemorrhagic stroke	All-cause mortality	CVD mortality
ID	Country	Sex	Age	Rate (95% CI)	Rate (95% CI)	Rate (95% CI)	Rate (95% CI)	Rate (95% CI)
899	Greece	Female	50 to 54	24 (18-34)	44 (38-50)	6 (4-7)	21 (19-23)	5 (4-6)
900	Greece	Female	55 to 59	34 (24-45)	56 (49-64)	7 (5-9)	31 (28-34)	8 (6-9)
901	Greece	Female	60 to 64	37 (27-49)	67 (59-77)	9 (7-11)	46 (41-50)	13 (11-15)
902	Greece	Female	65 to 69	34 (25-46)	83 (74-93)	12 (9-15)	72 (65-80)	24 (20-27)
903	Greece	Female	70 to <75	28 (19-42)	103 (91-117)	21 (17-24)	131 (118-145)	51 (45-57)
904	Greece	Male	40 to 44	23 (18-29)	13 (11-16)	3 (2-4)	17 (15-20)	6 (5-7)
905	Greece	Male	45 to 49	27 (19-37)	23 (20-28)	5 (4-7)	29 (26-32)	11 (10-13)
906	Greece	Male	50 to 54	33 (25-44)	41 (36-48)	8 (6-9)	46 (41-52)	18 (16-21)
907	Greece	Male	55 to 59	42 (31-59)	71 (62-83)	10 (8-13)	73 (66-82)	28 (24-32)
908	Greece	Male	60 to 64	44 (33-63)	112 (99-133)	14 (11-17)	107 (97-118)	39 (34-44)
909	Greece	Male	65 to 69	40 (29-53)	164 (144-194)	19 (14-23)	158 (142-174)	57 (50-64)
910	Greece	Male	70 to <75	32 (21-43)	220 (192-257)	28 (23-33)	250 (225-275)	93 (82-105)
911	Grenada	Female	40 to 44	81 (56-111)	26 (23-30)	3 (2-3)	29 (22-38)	6 (4-8)
912	Grenada	Female	45 to 49	109 (68-157)	38 (33-43)	5 (4-6)	44 (34-55)	11 (8-14)
913	Grenada	Female	50 to 54	154 (117-195)	50 (45-57)	7 (6-9)	65 (52-82)	17 (13-22)
914	Grenada	Female	55 to 59	216 (154-290)	59 (52-65)	10 (7-12)	95 (77-119)	28 (22-36)
915	Grenada	Female	60 to 64	207 (149-285)	69 (62-77)	13 (11-17)	140 (116-169)	46 (36-57)
916	Grenada	Female	65 to 69	126 (93-176)	87 (79-95)	19 (14-24)	214 (176-255)	77 (61-94)
917	Grenada	Female	70 to <75	75 (41-138)	104 (95-113)	26 (22-32)	344 (280-410)	130 (102-157)
918	Grenada	Male	40 to 44	98 (75-125)	13 (12-16)	3 (2-3)	50 (37-64)	10 (7-14)
919	Grenada	Male	45 to 49	148 (105-202)	23 (21-27)	5 (4-6)	72 (55-93)	18 (13-24)
920	Grenada	Male	50 to 54	156 (113-208)	40 (36-45)	8 (6-9)	105 (85-132)	30 (23-39)
921	Grenada	Male	55 to 59	122 (89-165)	65 (58-73)	11 (8-14)	155 (128-188)	52 (41-64)
922	Grenada	Male	60 to 64	92 (58-138)	104 (91-119)	15 (12-19)	229 (191-269)	84 (68-99)
923	Grenada	Male	65 to 69	66 (45-99)	149 (130-175)	21 (16-27)	338 (290-382)	126 (105-147)
924	Grenada	Male	70 to <75	51 (33-81)	233 (197-272)	28 (23-34)	506 (438-574)	185 (156-215)
925	Guatemala	Female	40 to 44	67 (51-89)	14 (12-17)	2 (2-3)	24 (15-36)	3 (2-5)
926	Guatemala	Female	45 to 49	71 (51-98)	21 (18-26)	4 (3-5)	37 (24-52)	5 (3-8)
927	Guatemala	Female	50 to 54	73 (54-100)	29 (25-35)	5 (4-6)	58 (38-81)	9 (6-13)
928	Guatemala	Female	55 to 59	73 (53-99)	37 (31-44)	7 (5-9)	84 (57-117)	13 (9-20)
929	Guatemala	Female	60 to 64	69 (48-97)	44 (38-51)	9 (7-11)	115 (78-158)	20 (13-29)
930	Guatemala	Female	65 to 69	60 (42-88)	53 (46-62)	11 (8-14)	165 (111-228)	32 (21-45)
931	Guatemala	Female	70 to <75	53 (36-80)	64 (55-76)	15 (12-18)	246 (164-342)	54 (36-78)
932	Guatemala	Male	40 to 44	89 (67-113)	7 (6-8)	2 (2-3)	60 (40-84)	5 (3-8)
933	Guatemala	Male	45 to 49	108 (78-150)	11 (9-14)	3 (2-5)	76 (50-106)	9 (5-13)
934	Guatemala	Male	50 to 54	108 (76-149)	17 (15-21)	5 (4-6)	100 (66-138)	14 (9-20)
935	Guatemala	Male	55 to 59	87 (62-116)	26 (22-32)	6 (5-9)	124 (85-166)	20 (13-28)
936	Guatemala	Male	60 to 64	71 (47-102)	39 (34-47)	9 (7-11)	155 (110-205)	29 (20-40)
937	Guatemala	Male	65 to 69	60 (42-87)	58 (50-69)	11 (8-15)	212 (150-278)	43 (30-58)
938	Guatemala	Male	70 to <75	56 (38-81)	84 (72-101)	15 (12-19)	320 (228-417)	72 (51-99)
939	Guinea	Female	40 to 44	30 (22-38)	21 (14-26)	2 (2-3)	63 (53-75)	6 (4-9)
940	Guinea	Female	45 to 49	42 (29-59)	25 (18-30)	4 (3-5)	80 (65-98)	12 (8-16)
941	Guinea	Female	50 to 54	49 (36-65)	28 (23-33)	6 (5-7)	112 (87-140)	24 (17-33)
942	Guinea	Female	55 to 59	50 (34-69)	28 (24-32)	8 (6-11)	162 (125-199)	44 (31-59)
943	Guinea	Female	60 to 64	53 (36-73)	34 (29-39)	12 (10-15)	244 (193-294)	77 (56-100)
944	Guinea	Female	65 to 69	58 (41-81)	33 (29-38)	17 (12-22)	378 (300-447)	133 (100-168)
945	Guinea	Female	70 to <75	57 (40-80)	37 (33-42)	25 (20-30)	585 (481-676)	206 (161-255)
946	Guinea	Male	40 to 44	24 (18-31)	13 (10-17)	3 (2-3)	76 (63-92)	9 (6-13)
947	Guinea	Male	45 to 49	31 (22-42)	17 (14-20)	5 (3-6)	98 (80-117)	15 (11-21)
948	Guinea	Male	50 to 54	35 (27-46)	23 (19-28)	7 (5-8)	128 (106-150)	25 (18-32)
949	Guinea	Male	55 to 59	37 (26-52)	25 (22-30)	9 (7-12)	174 (142-202)	40 (30-52)
950	Guinea	Male	60 to 64	40 (29-54)	34 (30-39)	13 (10-16)	257 (216-290)	69 (54-87)
951	Guinea	Male	65 to 69	42 (30-59)	43 (38-48)	18 (13-23)	377 (319-419)	104 (81-128)
952	Guinea	Male	70 to <75	41 (29-56)	54 (48-60)	24 (20-29)	581 (512-635)	173 (142-209)
953	Guinea-Bissau	Female	40 to 44	39 (29-51)	16 (10-21)	3 (2-4)	85 (71-100)	10 (7-14)
954	Guinea-Bissau	Female	45 to 49	55 (38-77)	21 (14-26)	5 (4-7)	103 (84-123)	19 (13-25)
955	Guinea-Bissau	Female	50 to 54	63 (47-84)	26 (19-32)	7 (6-9)	139 (112-162)	34 (25-45)
956	Guinea-Bissau	Female	55 to 59	63 (44-90)	32 (23-40)	10 (7-13)	189 (156-220)	55 (41-70)
957	Guinea-Bissau	Female	60 to 64	65 (44-92)	41 (32-50)	14 (11-17)	274 (233-314)	91 (70-113)
958	Guinea-Bissau	Female	65 to 69	69 (46-99)	45 (35-53)	18 (14-24)	415 (362-473)	155 (124-189)
959	Guinea-Bissau	Female	70 to <75	67 (45-93)	48 (39-56)	26 (21-32)	632 (552-725)	240 (195-295)
960	Guinea-Bissau	Male	40 to 44	34 (25-45)	7 (5-9)	3 (3-4)	126 (112-143)	18 (13-23)
961	Guinea-Bissau	Male	45 to 49	44 (29-62)	11 (9-14)	6 (5-8)	158 (137-183)	29 (22-39)
962	Guinea-Bissau	Male	50 to 54	49 (36-66)	18 (14-22)	9 (7-11)	205 (171-241)	47 (35-61)
963	Guinea-Bissau	Male	55 to 59	50 (35-70)	25 (20-30)	12 (9-15)	266 (218-314)	71 (53-90)
964	Guinea-Bissau	Male	60 to 64	51 (36-71)	37 (31-44)	16 (13-20)	378 (308-436)	117 (90-146)
965	Guinea-Bissau	Male	65 to 69	51 (37-72)	54 (47-65)	22 (17-28)	535 (445-606)	165 (128-200)
966	Guinea-Bissau	Male	70 to <75	47 (33-67)	75 (65-90)	30 (25-36)	788 (672-878)	260 (212-312)
967	Guyana	Female	40 to 44	86 (63-115)	18 (16-20)	3 (2-4)	37 (30-43)	8 (6-11)
968	Guyana	Female	45 to 49	123 (81-179)	26 (23-30)	6 (4-7)	53 (44-62)	16 (13-19)

				Diabetes type 2	Cancers	Hemorrhagic stroke	All-cause mortality	CVD mortality
**ID**	**Country**	**Sex**	**Age**	**Rate (95% CI)**	**Rate (95% CI)**	**Rate (95% CI)**	**Rate (95% CI)**	**Rate (95% CI)**
969	Guyana	Female	50 to 54	154 (113-205)	31 (28-35)	9 (7-10)	76 (65-88)	27 (22-32)
970	Guyana	Female	55 to 59	178 (131-233)	40 (36-45)	12 (9-15)	121 (104-138)	47 (39-56)
971	Guyana	Female	60 to 64	160 (109-218)	47 (43-52)	17 (14-21)	175 (152-198)	76 (65-88)
972	Guyana	Female	65 to 69	100 (70-138)	56 (51-61)	23 (18-29)	257 (223-291)	124 (107-143)
973	Guyana	Female	70 to <75	62 (37-105)	69 (63-75)	34 (29-40)	422 (364-480)	217 (185-248)
974	Guyana	Male	40 to 44	103 (78-133)	7 (6-8)	3 (2-3)	67 (58-77)	12 (9-16)
975	Guyana	Male	45 to 49	163 (114-223)	11 (10-13)	5 (4-7)	92 (79-107)	25 (20-30)
976	Guyana	Male	50 to 54	171 (127-219)	20 (18-23)	8 (7-10)	133 (115-153)	48 (39-57)
977	Guyana	Male	55 to 59	127 (95-163)	33 (30-38)	13 (10-17)	204 (177-233)	88 (74-102)
978	Guyana	Male	60 to 64	92 (52-137)	54 (48-61)	20 (16-24)	291 (256-328)	136 (118-156)
979	Guyana	Male	65 to 69	67 (46-102)	77 (68-90)	29 (23-36)	401 (356-449)	199 (175-226)
980	Guyana	Male	70 to <75	52 (32-83)	117 (99-139)	39 (32-46)	592 (529-658)	295 (260-334)
981	Haiti	Female	40 to 44	67 (50-89)	19 (12-25)	4 (3-4)	66 (50-87)	15 (10-22)
982	Haiti	Female	45 to 49	92 (62-131)	26 (18-34)	6 (5-8)	87 (63-113)	26 (18-36)
983	Haiti	Female	50 to 54	112 (83-148)	33 (23-42)	9 (7-11)	121 (88-157)	45 (30-60)
984	Haiti	Female	55 to 59	127 (92-173)	41 (31-51)	12 (9-16)	170 (125-216)	71 (50-93)
985	Haiti	Female	60 to 64	118 (79-165)	51 (40-61)	17 (14-21)	247 (190-305)	113 (83-142)
986	Haiti	Female	65 to 69	84 (61-117)	64 (52-78)	24 (18-30)	374 (292-457)	183 (140-229)
987	Haiti	Female	70 to <75	60 (37-93)	79 (64-93)	34 (28-41)	575 (455-713)	296 (230-369)
988	Haiti	Male	40 to 44	56 (43-70)	7 (6-8)	3 (2-4)	74 (58-94)	15 (10-22)
989	Haiti	Male	45 to 49	77 (57-101)	12 (10-14)	5 (4-7)	94 (72-121)	26 (17-36)
990	Haiti	Male	50 to 54	81 (61-105)	19 (16-22)	8 (6-9)	121 (92-156)	40 (27-56)
991	Haiti	Male	55 to 59	69 (51-95)	30 (26-34)	10 (8-14)	161 (124-208)	62 (44-82)
992	Haiti	Male	60 to 64	57 (38-85)	47 (41-53)	14 (12-18)	233 (184-293)	96 (71-126)
993	Haiti	Male	65 to 69	47 (33-65)	71 (61-80)	20 (15-26)	343 (271-426)	147 (109-192)
994	Haiti	Male	70 to <75	40 (27-58)	103 (87-119)	27 (22-33)	524 (415-642)	227 (171-295)
995	Honduras	Female	40 to 44	52 (38-70)	13 (11-16)	2 (1-3)	29 (17-44)	5 (3-8)
996	Honduras	Female	45 to 49	56 (39-75)	21 (18-26)	3 (3-5)	44 (27-64)	8 (5-13)
997	Honduras	Female	50 to 54	59 (43-79)	28 (24-34)	5 (4-6)	66 (43-94)	14 (8-20)
998	Honduras	Female	55 to 59	61 (44-81)	35 (29-42)	6 (4-9)	98 (69-137)	21 (14-31)
999	Honduras	Female	60 to 64	59 (40-81)	45 (38-53)	8 (6-11)	149 (111-196)	35 (25-48)
1000	Honduras	Female	65 to 69	52 (37-72)	57 (49-66)	11 (8-14)	229 (166-295)	59 (41-81)
1001	Honduras	Female	70 to <75	47 (31-67)	68 (58-81)	15 (12-18)	362 (248-469)	101 (68-135)
1002	Honduras	Male	40 to 44	51 (39-65)	5 (4-6)	2 (2-3)	36 (23-53)	8 (5-13)
1003	Honduras	Male	45 to 49	63 (46-86)	8 (7-12)	4 (3-5)	51 (32-75)	14 (9-22)
1004	Honduras	Male	50 to 54	66 (49-90)	15 (12-19)	5 (4-7)	75 (48-109)	25 (15-38)
1005	Honduras	Male	55 to 59	61 (44-83)	24 (20-31)	7 (5-10)	111 (72-160)	43 (28-63)
1006	Honduras	Male	60 to 64	56 (37-79)	40 (34-48)	10 (8-13)	168 (114-240)	71 (48-103)
1007	Honduras	Male	65 to 69	49 (35-70)	61 (51-72)	14 (10-18)	253 (175-359)	113 (78-161)
1008	Honduras	Male	70 to <75	46 (32-67)	86 (73-104)	18 (14-22)	389 (271-545)	177 (121-251)
1009	Hungary	Female	40 to 44	39 (31-49)	25 (22-29)	3 (2-4)	14 (12-17)	3 (2-4)
1010	Hungary	Female	45 to 49	40 (28-55)	44 (38-51)	5 (3-6)	29 (25-33)	6 (5-7)
1011	Hungary	Female	50 to 54	43 (32-59)	63 (56-71)	6 (5-7)	48 (42-56)	10 (8-13)
1012	Hungary	Female	55 to 59	50 (36-66)	82 (72-93)	7 (5-10)	72 (63-83)	18 (15-22)
1013	Hungary	Female	60 to 64	51 (37-69)	106 (95-118)	9 (7-12)	105 (92-121)	31 (26-37)
1014	Hungary	Female	65 to 69	48 (34-66)	121 (108-135)	12 (9-15)	151 (131-173)	55 (46-65)
1015	Hungary	Female	70 to <75	41 (28-60)	134 (121-152)	16 (13-20)	236 (205-272)	106 (91-124)
1016	Hungary	Male	40 to 44	59 (48-73)	15 (14-18)	4 (3-5)	30 (25-35)	8 (6-9)
1017	Hungary	Male	45 to 49	58 (42-80)	35 (31-39)	6 (5-8)	60 (51-71)	17 (13-21)
1018	Hungary	Male	50 to 54	58 (41-79)	70 (64-77)	9 (7-11)	110 (93-128)	33 (27-40)
1019	Hungary	Male	55 to 59	59 (44-79)	114 (103-126)	13 (10-17)	168 (144-194)	55 (45-65)
1020	Hungary	Male	60 to 64	55 (38-76)	162 (144-182)	16 (13-20)	238 (207-271)	86 (73-101)
1021	Hungary	Male	65 to 69	45 (33-64)	208 (182-243)	20 (15-25)	316 (276-359)	125 (107-144)
1022	Hungary	Male	70 to <75	36 (24-55)	252 (220-301)	25 (20-31)	451 (395-511)	201 (172-230)
1023	Iceland	Female	40 to 44	22 (17-29)	38 (33-43)	2 (2-3)	8 (7-10)	1 (1-1)
1024	Iceland	Female	45 to 49	22 (16-30)	46 (40-54)	3 (2-4)	11 (10-13)	1 (1-1)
1025	Iceland	Female	50 to 54	28 (21-36)	63 (56-72)	4 (3-5)	19 (16-22)	2 (1-2)
1026	Iceland	Female	55 to 59	39 (28-51)	88 (77-99)	4 (3-6)	32 (28-37)	3 (3-4)
1027	Iceland	Female	60 to 64	43 (32-56)	127 (113-141)	6 (5-8)	55 (48-63)	8 (6-9)
1028	Iceland	Female	65 to 69	42 (30-56)	156 (139-173)	9 (7-11)	89 (78-101)	15 (12-18)
1029	Iceland	Female	70 to <75	36 (25-50)	179 (160-200)	14 (12-17)	145 (127-165)	31 (25-37)
1030	Iceland	Male	40 to 44	28 (22-35)	21 (18-25)	2 (2-3)	12 (11-14)	2 (1-2)
1031	Iceland	Male	45 to 49	32 (23-46)	31 (27-38)	4 (3-5)	18 (16-20)	4 (3-5)
1032	Iceland	Male	50 to 54	41 (31-54)	51 (45-59)	5 (4-7)	29 (26-32)	7 (6-8)
1033	Iceland	Male	55 to 59	55 (38-75)	84 (74-97)	7 (5-9)	47 (42-53)	12 (10-15)
1034	Iceland	Male	60 to 64	60 (45-81)	134 (118-154)	9 (7-11)	73 (66-81)	20 (17-23)
1035	Iceland	Male	65 to 69	58 (42-78)	218 (190-251)	12 (9-15)	128 (116-142)	38 (33-43)
1036	Iceland	Male	70 to <75	48 (33-68)	300 (256-346)	19 (16-23)	215 (195-237)	67 (58-77)
1037	India	Female	40 to 44	52 (40-65)	11 (11-12)	2 (1-2)	29 (27-30)	7 (6-7)
1038	India	Female	45 to 49	66 (49-84)	16 (15-17)	3 (2-4)	43 (41-45)	12 (11-12)

				Diabetes type 2	Cancers	Hemorrhagic stroke	All-cause mortality	CVD mortality
ID	Country	Sex	Age	Rate (95% CI)	Rate (95% CI)	Rate (95% CI)	Rate (95% CI)	Rate (95% CI)
1039	India	Female	50 to 54	68 (51-87)	22 (21-24)	5 (4-6)	70 (67-73)	20 (19-22)
1040	India	Female	55 to 59	55 (40-73)	26 (25-28)	7 (5-9)	104 (99-108)	34 (31-36)
1041	India	Female	60 to 64	44 (28-65)	30 (29-33)	10 (8-13)	173 (166-181)	58 (53-63)
1042	India	Female	65 to 69	36 (24-52)	34 (33-36)	14 (11-18)	280 (269-293)	97 (87-104)
1043	India	Female	70 to <75	38 (26-54)	37 (36-39)	19 (15-22)	438 (420-457)	146 (129-158)
1044	India	Male	40 to 44	63 (48-78)	6 (6-6)	2 (2-3)	53 (51-55)	14 (13-14)
1045	India	Male	45 to 49	69 (50-91)	10 (9-10)	3 (3-5)	75 (72-78)	23 (21-24)
1046	India	Male	50 to 54	71 (53-93)	16 (16-17)	6 (5-7)	106 (102-111)	38 (36-40)
1047	India	Male	55 to 59	71 (52-95)	25 (24-26)	8 (6-11)	160 (154-166)	61 (57-64)
1048	India	Male	60 to 64	63 (42-87)	33 (32-35)	12 (10-15)	233 (225-242)	86 (81-91)
1049	India	Male	65 to 69	49 (34-70)	43 (42-45)	17 (13-21)	353 (341-367)	132 (124-139)
1050	India	Male	70 to <75	47 (31-68)	51 (49-53)	20 (16-24)	540 (522-559)	196 (181-207)
1051	Indonesia	Female	40 to 44	64 (47-82)	17 (15-19)	3 (2-4)	27 (26-29)	7 (7-8)
1052	Indonesia	Female	45 to 49	78 (54-105)	23 (21-25)	5 (4-7)	41 (39-44)	13 (12-14)
1053	Indonesia	Female	50 to 54	91 (69-115)	27 (24-30)	8 (7-10)	63 (60-67)	23 (22-25)
1054	Indonesia	Female	55 to 59	101 (75-131)	32 (29-35)	11 (8-14)	96 (90-101)	38 (35-40)
1055	Indonesia	Female	60 to 64	92 (66-125)	36 (33-40)	15 (12-18)	149 (142-156)	61 (57-65)
1056	Indonesia	Female	65 to 69	63 (47-88)	40 (37-44)	18 (14-23)	233 (222-245)	107 (100-114)
1057	Indonesia	Female	70 to <75	45 (29-68)	44 (41-49)	28 (23-33)	373 (354-392)	176 (164-190)
1058	Indonesia	Male	40 to 44	52 (37-69)	5 (5-6)	4 (4-5)	39 (37-41)	13 (12-14)
1059	Indonesia	Male	45 to 49	50 (35-69)	8 (8-10)	8 (6-10)	56 (53-60)	21 (20-23)
1060	Indonesia	Male	50 to 54	55 (39-74)	14 (12-15)	12 (10-14)	84 (78-90)	35 (32-38)
1061	Indonesia	Male	55 to 59	66 (46-88)	21 (20-23)	17 (13-22)	128 (118-138)	56 (51-61)
1062	Indonesia	Male	60 to 64	65 (44-89)	32 (30-35)	23 (19-28)	196 (180-212)	85 (78-93)
1063	Indonesia	Male	65 to 69	51 (36-72)	47 (43-51)	30 (23-38)	299 (271-326)	133 (120-146)
1064	Indonesia	Male	70 to <75	42 (28-62)	65 (58-69)	40 (33-48)	461 (417-503)	201 (180-222)
1065	Iran	Female	40 to 44	66 (51-84)	13 (10-15)	2 (1-2)	13 (10-17)	3 (2-5)
1066	Iran	Female	45 to 49	82 (59-108)	17 (14-20)	2 (2-3)	20 (15-26)	6 (5-8)
1067	Iran	Female	50 to 54	92 (70-118)	21 (17-24)	3 (3-4)	31 (24-41)	12 (9-15)
1068	Iran	Female	55 to 59	97 (72-127)	26 (22-29)	4 (3-6)	50 (38-65)	21 (16-28)
1069	Iran	Female	60 to 64	92 (66-123)	34 (28-38)	6 (5-8)	86 (65-109)	39 (30-51)
1070	Iran	Female	65 to 69	75 (54-104)	42 (36-47)	9 (7-12)	145 (111-183)	72 (55-92)
1071	Iran	Female	70 to <75	62 (43-90)	53 (46-59)	13 (10-16)	250 (192-304)	132 (100-161)
1072	Iran	Male	40 to 44	66 (51-85)	7 (6-7)	2 (1-2)	27 (20-34)	8 (6-11)
1073	Iran	Male	45 to 49	87 (63-115)	11 (10-12)	3 (2-4)	38 (28-49)	14 (10-19)
1074	Iran	Male	50 to 54	97 (74-124)	19 (17-21)	4 (3-5)	59 (45-78)	26 (19-33)
1075	Iran	Male	55 to 59	95 (70-129)	30 (28-33)	6 (4-8)	89 (67-116)	41 (30-53)
1076	Iran	Male	60 to 64	91 (64-126)	46 (41-51)	8 (6-10)	137 (108-174)	65 (51-83)
1077	Iran	Male	65 to 69	84 (61-116)	70 (62-76)	10 (7-13)	214 (168-271)	103 (81-129)
1078	Iran	Male	70 to <75	74 (50-106)	97 (87-107)	13 (11-17)	331 (263-417)	160 (127-202)
1079	Iraq	Female	40 to 44	124 (92-165)	11 (9-13)	3 (2-4)	38 (26-54)	11 (7-17)
1080	Iraq	Female	45 to 49	151 (102-206)	16 (13-19)	5 (4-7)	55 (38-77)	19 (12-28)
1081	Iraq	Female	50 to 54	162 (116-216)	20 (17-23)	7 (6-9)	82 (58-111)	35 (24-48)
1082	Iraq	Female	55 to 59	158 (114-211)	24 (21-28)	10 (7-13)	120 (87-161)	58 (41-77)
1083	Iraq	Female	60 to 64	134 (89-194)	29 (25-34)	14 (11-17)	178 (134-228)	94 (69-121)
1084	Iraq	Female	65 to 69	92 (66-127)	33 (29-37)	20 (15-26)	274 (211-344)	156 (117-196)
1085	Iraq	Female	70 to <75	65 (42-97)	38 (34-42)	27 (22-33)	431 (339-540)	259 (204-326)
1086	Iraq	Male	40 to 44	114 (83-154)	5 (5-6)	3 (2-4)	61 (43-86)	19 (12-28)
1087	Iraq	Male	45 to 49	136 (89-188)	9 (8-9)	5 (4-7)	82 (59-114)	34 (24-50)
1088	Iraq	Male	50 to 54	148 (102-200)	14 (13-16)	8 (7-10)	116 (81-158)	58 (40-82)
1089	Iraq	Male	55 to 59	148 (103-206)	23 (21-25)	12 (9-15)	169 (118-221)	92 (65-125)
1090	Iraq	Male	60 to 64	133 (90-200)	33 (30-36)	16 (13-20)	251 (175-317)	144 (101-184)
1091	Iraq	Male	65 to 69	104 (75-144)	48 (44-53)	22 (16-28)	374 (260-453)	222 (157-272)
1092	Iraq	Male	70 to <75	78 (51-116)	61 (56-67)	29 (23-34)	569 (393-668)	340 (237-404)
1093	Ireland	Female	40 to 44	26 (20-34)	31 (26-37)	2 (2-3)	8 (7-10)	1 (1-1)
1094	Ireland	Female	45 to 49	27 (19-39)	47 (40-55)	4 (3-5)	15 (12-18)	2 (2-3)
1095	Ireland	Female	50 to 54	35 (26-47)	60 (52-69)	5 (4-6)	22 (19-27)	3 (2-4)
1096	Ireland	Female	55 to 59	49 (35-67)	79 (69-91)	6 (4-8)	35 (29-42)	5 (4-7)
1097	Ireland	Female	60 to 64	56 (43-74)	106 (93-120)	7 (6-10)	56 (47-67)	10 (8-12)
1098	Ireland	Female	65 to 69	56 (40-79)	130 (116-148)	10 (7-13)	89 (74-106)	19 (15-24)
1099	Ireland	Female	70 to <75	49 (33-74)	161 (142-183)	16 (13-20)	154 (128-184)	39 (31-49)
1100	Ireland	Male	40 to 44	32 (25-41)	16 (14-20)	3 (2-4)	15 (12-18)	3 (2-4)
1101	Ireland	Male	45 to 49	38 (26-50)	27 (23-33)	4 (3-6)	22 (19-27)	6 (5-7)
1102	Ireland	Male	50 to 54	50 (38-64)	47 (41-55)	6 (5-7)	35 (29-42)	10 (8-13)
1103	Ireland	Male	55 to 59	68 (50-90)	83 (71-98)	7 (5-10)	56 (47-67)	17 (13-20)
1104	Ireland	Male	60 to 64	75 (57-98)	144 (124-171)	10 (8-12)	91 (78-108)	28 (23-33)
1105	Ireland	Male	65 to 69	73 (52-97)	220 (184-263)	14 (10-18)	149 (128-175)	46 (38-55)
1106	Ireland	Male	70 to <75	61 (41-87)	301 (253-361)	21 (18-26)	251 (215-294)	81 (68-96)
1107	Israel	Female	40 to 44	33 (25-42)	21 (18-24)	2 (2-3)	7 (5-9)	1 (0-1)
1108	Israel	Female	45 to 49	39 (26-55)	30 (26-36)	4 (3-5)	12 (9-16)	1 (1-2)

				Diabetes type 2	Cancers	Hemorrhagic stroke	All-cause mortality	CVD mortality
ID	Country	Sex	Age	Rate (95% CI)	Rate (95% CI)	Rate (95% CI)	Rate (95% CI)	Rate (95% CI)
1109	Israel	Female	50 to 54	56 (43-72)	40 (35-46)	5 (4-6)	20 (15-25)	2 (1-3)
1110	Israel	Female	55 to 59	83 (58-113)	51 (44-59)	5 (4-7)	31 (24-40)	4 (3-5)
1111	Israel	Female	60 to 64	94 (69-123)	68 (60-76)	7 (5-8)	49 (38-62)	7 (5-10)
1112	Israel	Female	65 to 69	87 (65-115)	85 (76-96)	9 (7-12)	79 (62-101)	14 (11-19)
1113	Israel	Female	70 to <75	71 (46-100)	108 (97-121)	15 (12-18)	147 (114-189)	33 (25-43)
1114	Israel	Male	40 to 44	42 (32-53)	11 (9-13)	3 (2-3)	14 (10-19)	2 (2-3)
1115	Israel	Male	45 to 49	57 (39-81)	17 (15-22)	4 (3-6)	21 (15-27)	4 (3-5)
1116	Israel	Male	50 to 54	78 (59-100)	29 (26-35)	6 (5-7)	32 (24-42)	7 (5-9)
1117	Israel	Male	55 to 59	103 (74-136)	49 (43-58)	8 (5-10)	51 (38-66)	12 (8-16)
1118	Israel	Male	60 to 64	110 (84-143)	82 (73-94)	11 (9-14)	85 (66-109)	20 (15-27)
1119	Israel	Male	65 to 69	97 (71-129)	129 (116-147)	16 (12-21)	149 (115-188)	37 (28-48)
1120	Israel	Male	70 to <75	76 (49-112)	174 (156-200)	23 (19-28)	239 (185-303)	64 (48-83)
1121	Italy	Female	40 to 44	27 (21-35)	33 (29-38)	2 (1-2)	7 (7-8)	1 (1-1)
1122	Italy	Female	45 to 49	29 (20-41)	51 (45-57)	3 (2-4)	12 (11-14)	2 (1-2)
1123	Italy	Female	50 to 54	39 (30-51)	65 (58-73)	4 (3-5)	20 (18-21)	3 (2-3)
1124	Italy	Female	55 to 59	58 (41-77)	80 (72-89)	5 (4-6)	30 (27-33)	4 (3-5)
1125	Italy	Female	60 to 64	66 (51-84)	99 (90-110)	7 (6-8)	46 (42-50)	8 (6-9)
1126	Italy	Female	65 to 69	63 (46-84)	124 (112-136)	10 (8-12)	73 (67-80)	15 (13-18)
1127	Italy	Female	70 to <75	52 (34-75)	140 (128-154)	17 (15-19)	123 (111-134)	32 (27-37)
1128	Italy	Male	40 to 44	31 (24-39)	17 (15-19)	2 (2-3)	12 (11-14)	2 (2-3)
1129	Italy	Male	45 to 49	40 (27-57)	28 (25-31)	4 (3-5)	20 (18-22)	4 (4-5)
1130	Italy	Male	50 to 54	54 (41-69)	51 (47-57)	6 (5-6)	32 (29-36)	7 (6-9)
1131	Italy	Male	55 to 59	73 (52-98)	91 (82-101)	8 (7-9)	53 (48-58)	12 (11-15)
1132	Italy	Male	60 to 64	79 (59-103)	152 (137-169)	11 (10-12)	87 (80-96)	21 (18-24)
1133	Italy	Male	65 to 69	70 (51-91)	229 (202-257)	15 (13-18)	141 (129-153)	35 (30-40)
1134	Italy	Male	70 to <75	56 (36-77)	301 (268-340)	25 (23-28)	230 (211-251)	63 (55-72)
1135	Jamaica	Female	40 to 44	74 (52-101)	22 (19-26)	3 (2-3)	20 (14-26)	3 (2-5)
1136	Jamaica	Female	45 to 49	106 (64-155)	31 (27-36)	4 (3-6)	30 (23-40)	6 (4-9)
1137	Jamaica	Female	50 to 54	187 (140-239)	40 (35-46)	7 (5-8)	47 (36-59)	11 (8-14)
1138	Jamaica	Female	55 to 59	318 (213-405)	53 (46-60)	9 (7-12)	72 (55-88)	18 (13-23)
1139	Jamaica	Female	60 to 64	312 (230-398)	67 (59-76)	13 (11-17)	114 (90-138)	33 (25-41)
1140	Jamaica	Female	65 to 69	170 (128-238)	77 (69-85)	19 (14-25)	174 (135-214)	55 (42-71)
1141	Jamaica	Female	70 to <75	84 (42-185)	86 (79-96)	27 (22-33)	272 (216-338)	97 (74-123)
1142	Jamaica	Male	40 to 44	93 (68-120)	8 (7-10)	3 (2-3)	31 (24-39)	5 (3-7)
1143	Jamaica	Male	45 to 49	161 (107-218)	15 (13-17)	5 (3-7)	44 (34-55)	10 (7-13)
1144	Jamaica	Male	50 to 54	185 (139-234)	27 (23-31)	7 (6-9)	65 (50-84)	17 (12-22)
1145	Jamaica	Male	55 to 59	166 (116-226)	45 (38-52)	10 (8-14)	99 (76-127)	28 (20-37)
1146	Jamaica	Male	60 to 64	137 (80-202)	77 (61-90)	15 (12-18)	152 (121-193)	47 (36-61)
1147	Jamaica	Male	65 to 69	96 (66-142)	113 (89-133)	21 (15-26)	231 (185-295)	74 (57-96)
1148	Jamaica	Male	70 to <75	71 (42-119)	157 (121-190)	28 (22-34)	358 (290-454)	119 (93-155)
1149	Japan	Female	40 to 44	23 (16-31)	25 (24-27)	2 (2-3)	8 (7-8)	1 (1-1)
1150	Japan	Female	45 to 49	27 (18-37)	36 (35-39)	4 (3-5)	12 (11-12)	2 (2-2)
1151	Japan	Female	50 to 54	31 (22-42)	45 (43-48)	5 (4-6)	18 (17-18)	3 (3-3)
1152	Japan	Female	55 to 59	36 (25-50)	55 (53-58)	6 (4-8)	25 (25-26)	4 (4-4)
1153	Japan	Female	60 to 64	37 (25-52)	66 (64-69)	8 (6-10)	37 (36-38)	6 (6-7)
1154	Japan	Female	65 to 69	35 (24-52)	78 (76-84)	10 (8-13)	55 (53-57)	11 (10-11)
1155	Japan	Female	70 to <75	31 (21-48)	96 (92-103)	14 (12-17)	90 (87-93)	20 (19-21)
1156	Japan	Male	40 to 44	32 (23-42)	12 (11-12)	3 (2-3)	13 (13-13)	3 (3-3)
1157	Japan	Male	45 to 49	36 (25-50)	19 (18-20)	5 (4-6)	20 (20-21)	5 (4-5)
1158	Japan	Male	50 to 54	40 (30-54)	35 (33-37)	7 (6-8)	33 (32-34)	8 (7-8)
1159	Japan	Male	55 to 59	43 (31-57)	64 (60-67)	9 (7-12)	52 (51-54)	11 (11-12)
1160	Japan	Male	60 to 64	41 (28-57)	109 (105-115)	12 (9-14)	84 (82-87)	18 (17-19)
1161	Japan	Male	65 to 69	34 (24-51)	168 (162-176)	15 (11-18)	130 (127-134)	27 (26-29)
1162	Japan	Male	70 to <75	28 (18-43)	229 (217-240)	19 (16-23)	205 (199-211)	45 (43-47)
1163	Jordan	Female	40 to 44	88 (65-118)	15 (12-18)	2 (1-2)	15 (10-23)	3 (2-4)
1164	Jordan	Female	45 to 49	122 (83-168)	22 (18-27)	3 (2-4)	23 (16-35)	5 (3-8)
1165	Jordan	Female	50 to 54	142 (106-188)	26 (22-32)	4 (3-5)	38 (25-56)	11 (7-17)
1166	Jordan	Female	55 to 59	146 (109-200)	32 (27-38)	6 (4-8)	60 (39-88)	21 (13-31)
1167	Jordan	Female	60 to 64	129 (88-183)	39 (31-48)	9 (7-11)	99 (66-138)	39 (25-55)
1168	Jordan	Female	65 to 69	89 (64-123)	43 (36-53)	12 (9-16)	157 (106-216)	70 (46-98)
1169	Jordan	Female	70 to <75	63 (40-93)	52 (44-62)	16 (13-20)	254 (178-347)	119 (82-164)
1170	Jordan	Male	40 to 44	93 (70-121)	7 (6-7)	2 (1-2)	24 (16-32)	8 (5-11)
1171	Jordan	Male	45 to 49	127 (86-167)	11 (10-12)	3 (2-4)	34 (23-48)	14 (9-20)
1172	Jordan	Male	50 to 54	141 (102-183)	18 (16-19)	5 (4-6)	53 (35-76)	23 (15-34)
1173	Jordan	Male	55 to 59	137 (99-192)	26 (24-28)	7 (5-9)	81 (53-118)	36 (23-52)
1174	Jordan	Male	60 to 64	122 (83-176)	42 (38-48)	10 (8-12)	133 (90-188)	60 (40-86)
1175	Jordan	Male	65 to 69	97 (69-138)	58 (53-64)	13 (9-17)	204 (139-292)	93 (64-131)
1176	Jordan	Male	70 to <75	74 (46-110)	77 (70-85)	17 (14-21)	317 (216-447)	143 (97-203)
1177	Kazakhstan	Female	40 to 44	29 (22-36)	20 (17-23)	4 (3-4)	24 (18-31)	6 (4-8)
1178	Kazakhstan	Female	45 to 49	31 (22-42)	28 (24-33)	6 (5-8)	32 (25-41)	10 (8-14)

				Diabetes type 2	Cancers	Hemorrhagic stroke	All-cause mortality	CVD mortality
ID	Country	Sex	Age	Rate (95% CI)	Rate (95% CI)	Rate (95% CI)	Rate (95% CI)	Rate (95% CI)
1179	Kazakhstan	Female	50 to 54	33 (24-43)	37 (32-44)	9 (7-11)	49 (39-62)	18 (14-23)
1180	Kazakhstan	Female	55 to 59	35 (26-45)	48 (42-57)	13 (10-17)	75 (60-95)	32 (25-40)
1181	Kazakhstan	Female	60 to 64	33 (24-45)	61 (53-71)	18 (14-22)	122 (97-152)	60 (47-75)
1182	Kazakhstan	Female	65 to 69	27 (19-38)	70 (62-80)	23 (17-29)	184 (146-229)	101 (79-127)
1183	Kazakhstan	Female	70 to <75	21 (14-31)	79 (69-93)	30 (25-36)	308 (243-386)	192 (149-240)
1184	Kazakhstan	Male	40 to 44	34 (25-42)	9 (8-12)	6 (5-7)	66 (50-85)	19 (14-26)
1185	Kazakhstan	Male	45 to 49	33 (23-44)	18 (15-23)	10 (7-13)	87 (66-113)	32 (23-42)
1186	Kazakhstan	Male	50 to 54	32 (23-44)	35 (30-41)	15 (12-18)	127 (98-164)	54 (41-69)
1187	Kazakhstan	Male	55 to 59	32 (22-42)	62 (54-73)	20 (15-25)	187 (146-236)	85 (65-110)
1188	Kazakhstan	Male	60 to 64	30 (20-42)	101 (90-115)	25 (20-31)	286 (229-354)	143 (114-176)
1189	Kazakhstan	Male	65 to 69	28 (20-41)	137 (122-153)	30 (22-37)	401 (324-493)	213 (171-264)
1190	Kazakhstan	Male	70 to <75	23 (16-35)	168 (147-191)	38 (31-46)	584 (474-712)	341 (274-418)
1191	Kenya	Female	40 to 44	20 (14-26)	10 (7-13)	2 (1-2)	52 (50-55)	3 (2-4)
1192	Kenya	Female	45 to 49	24 (16-33)	15 (11-20)	3 (2-3)	65 (61-69)	6 (5-8)
1193	Kenya	Female	50 to 54	29 (21-38)	22 (15-29)	4 (3-5)	85 (79-90)	11 (8-14)
1194	Kenya	Female	55 to 59	35 (24-49)	26 (17-34)	5 (4-7)	111 (102-119)	19 (14-25)
1195	Kenya	Female	60 to 64	39 (27-53)	40 (26-52)	8 (6-11)	165 (153-176)	31 (22-42)
1196	Kenya	Female	65 to 69	41 (28-58)	47 (31-60)	13 (9-17)	254 (235-274)	56 (40-74)
1197	Kenya	Female	70 to <75	41 (28-57)	47 (32-59)	19 (15-23)	401 (373-428)	99 (71-130)
1198	Kenya	Male	40 to 44	29 (22-39)	6 (5-7)	2 (1-2)	78 (74-82)	5 (4-6)
1199	Kenya	Male	45 to 49	43 (30-59)	9 (8-12)	3 (2-4)	98 (93-103)	9 (7-11)
1200	Kenya	Male	50 to 54	50 (38-65)	15 (13-17)	5 (4-6)	121 (114-128)	15 (12-18)
1201	Kenya	Male	55 to 59	52 (37-71)	22 (20-25)	7 (5-9)	154 (145-163)	24 (20-29)
1202	Kenya	Male	60 to 64	51 (35-71)	35 (31-39)	10 (8-13)	221 (209-233)	44 (35-53)
1203	Kenya	Male	65 to 69	48 (33-68)	48 (40-53)	15 (11-20)	324 (306-342)	73 (58-87)
1204	Kenya	Male	70 to <75	44 (30-62)	67 (55-74)	21 (17-26)	493 (466-519)	117 (93-141)
1205	Kiribati	Female	40 to 44	272 (200-350)	10 (8-14)	4 (3-5)	55 (42-70)	15 (11-20)
1206	Kiribati	Female	45 to 49	479 (303-663)	13 (10-17)	6 (5-9)	79 (61-98)	23 (17-31)
1207	Kiribati	Female	50 to 54	573 (438-720)	16 (13-22)	10 (8-12)	119 (94-144)	30 (21-39)
1208	Kiribati	Female	55 to 59	554 (418-716)	20 (17-26)	14 (10-18)	174 (140-208)	53 (40-69)
1209	Kiribati	Female	60 to 64	431 (270-629)	25 (22-29)	19 (15-23)	256 (211-302)	82 (62-105)
1210	Kiribati	Female	65 to 69	206 (145-286)	33 (28-41)	25 (18-32)	386 (324-448)	135 (105-168)
1211	Kiribati	Female	70 to <75	77 (46-122)	43 (38-50)	34 (27-40)	598 (501-701)	174 (136-218)
1212	Kiribati	Male	40 to 44	346 (263-454)	10 (8-11)	6 (5-7)	114 (91-138)	42 (32-55)
1213	Kiribati	Male	45 to 49	612 (418-775)	15 (13-17)	11 (8-14)	162 (130-196)	61 (45-77)
1214	Kiribati	Male	50 to 54	641 (475-793)	21 (18-24)	16 (13-19)	229 (186-272)	97 (76-123)
1215	Kiribati	Male	55 to 59	432 (334-582)	28 (25-32)	21 (16-26)	311 (260-363)	130 (104-161)
1216	Kiribati	Male	60 to 64	268 (156-447)	38 (34-43)	27 (22-33)	431 (372-494)	179 (147-214)
1217	Kiribati	Male	65 to 69	147 (96-215)	54 (49-60)	36 (27-45)	593 (523-671)	209 (173-251)
1218	Kiribati	Male	70 to <75	72 (44-119)	65 (59-73)	49 (41-59)	847 (750-948)	314 (264-371)
1219	Kuwait	Female	40 to 44	74 (55-97)	11 (9-12)	2 (1-2)	6 (4-10)	2 (1-3)
1220	Kuwait	Female	45 to 49	93 (66-132)	17 (14-20)	3 (2-4)	10 (6-15)	3 (2-5)
1221	Kuwait	Female	50 to 54	114 (87-152)	23 (20-26)	4 (3-5)	18 (12-27)	6 (4-9)
1222	Kuwait	Female	55 to 59	135 (101-180)	34 (30-39)	4 (3-6)	34 (22-50)	11 (7-16)
1223	Kuwait	Female	60 to 64	130 (94-176)	57 (50-64)	7 (5-9)	75 (49-108)	29 (19-42)
1224	Kuwait	Female	65 to 69	97 (69-136)	78 (70-87)	11 (7-14)	163 (106-234)	69 (45-100)
1225	Kuwait	Female	70 to <75	74 (47-109)	94 (84-103)	16 (12-19)	296 (191-427)	134 (85-194)
1226	Kuwait	Male	40 to 44	72 (54-93)	3 (3-4)	2 (1-2)	13 (8-19)	6 (4-9)
1227	Kuwait	Male	45 to 49	91 (65-127)	6 (5-7)	3 (2-4)	22 (13-32)	12 (8-18)
1228	Kuwait	Male	50 to 54	104 (77-140)	11 (10-12)	5 (4-6)	35 (22-53)	20 (13-30)
1229	Kuwait	Male	55 to 59	113 (83-157)	19 (17-21)	6 (4-9)	61 (39-89)	36 (23-52)
1230	Kuwait	Male	60 to 64	111 (78-158)	35 (32-40)	9 (7-11)	96 (63-137)	53 (35-76)
1231	Kuwait	Male	65 to 69	97 (69-141)	57 (51-65)	11 (8-15)	156 (104-221)	82 (55-116)
1232	Kuwait	Male	70 to <75	79 (53-118)	76 (68-88)	15 (12-18)	236 (157-333)	113 (76-159)
1233	Kyrgyzstan	Female	40 to 44	22 (18-28)	13 (11-16)	3 (3-4)	22 (19-25)	5 (4-6)
1234	Kyrgyzstan	Female	45 to 49	24 (17-31)	19 (16-24)	6 (5-8)	30 (27-35)	10 (8-11)
1235	Kyrgyzstan	Female	50 to 54	25 (19-32)	25 (22-30)	10 (8-12)	49 (43-56)	19 (16-22)
1236	Kyrgyzstan	Female	55 to 59	27 (20-35)	32 (27-39)	15 (11-19)	76 (67-86)	35 (31-41)
1237	Kyrgyzstan	Female	60 to 64	25 (18-35)	42 (36-51)	20 (17-25)	126 (112-142)	69 (60-79)
1238	Kyrgyzstan	Female	65 to 69	22 (16-30)	49 (42-58)	27 (22-34)	194 (172-219)	120 (105-136)
1239	Kyrgyzstan	Female	70 to <75	18 (12-25)	59 (50-71)	33 (27-39)	334 (296-378)	233 (206-263)
1240	Kyrgyzstan	Male	40 to 44	27 (21-33)	7 (6-10)	5 (4-7)	55 (48-63)	15 (12-18)
1241	Kyrgyzstan	Male	45 to 49	27 (19-38)	14 (11-19)	10 (8-13)	76 (65-86)	25 (21-30)
1242	Kyrgyzstan	Male	50 to 54	26 (19-38)	26 (21-32)	15 (13-18)	113 (98-128)	47 (39-54)
1243	Kyrgyzstan	Male	55 to 59	25 (18-34)	45 (37-60)	22 (17-27)	165 (145-186)	79 (69-90)
1244	Kyrgyzstan	Male	60 to 64	24 (17-33)	72 (60-96)	28 (23-34)	263 (233-293)	145 (127-163)
1245	Kyrgyzstan	Male	65 to 69	23 (17-33)	101 (83-141)	34 (27-42)	361 (321-400)	215 (190-241)
1246	Kyrgyzstan	Male	70 to <75	20 (14-30)	129 (103-186)	40 (32-47)	563 (503-622)	367 (325-408)
1247	Laos	Female	40 to 44	66 (47-86)	14 (11-18)	4 (3-4)	32 (30-35)	9 (7-11)
1248	Laos	Female	45 to 49	82 (57-111)	19 (16-23)	6 (5-8)	47 (44-50)	15 (12-17)

				Diabetes type 2	Cancers	Hemorrhagic stroke	All-cause mortality	CVD mortality
**ID**	**Country**	**Sex**	**Age**	**Rate (95% CI)**	**Rate (95% CI)**	**Rate (95% CI)**	**Rate (95% CI)**	**Rate (95% CI)**
1249	Laos	Female	50 to 54	96 (73-125)	24 (21-28)	9 (7-11)	70 (65-76)	25 (21-29)
1250	Laos	Female	55 to 59	108 (82-141)	31 (26-36)	12 (9-16)	106 (96-118)	41 (35-48)
1251	Laos	Female	60 to 64	100 (70-135)	37 (33-42)	16 (13-20)	167 (151-182)	69 (58-79)
1252	Laos	Female	65 to 69	70 (52-94)	44 (39-50)	21 (15-27)	261 (235-286)	119 (101-136)
1253	Laos	Female	70 to <75	48 (31-71)	52 (47-58)	31 (26-38)	421 (382-454)	202 (174-228)
1254	Laos	Male	40 to 44	53 (38-71)	6 (5-7)	4 (3-5)	53 (48-59)	16 (10-20)
1255	Laos	Male	45 to 49	50 (36-68)	10 (9-12)	7 (6-10)	75 (68-81)	26 (19-31)
1256	Laos	Male	50 to 54	52 (37-71)	17 (14-20)	12 (10-14)	107 (98-115)	41 (34-48)
1257	Laos	Male	55 to 59	58 (41-78)	27 (22-31)	17 (13-21)	153 (142-163)	64 (53-73)
1258	Laos	Male	60 to 64	56 (40-79)	41 (34-49)	23 (19-28)	231 (215-247)	99 (85-112)
1259	Laos	Male	65 to 69	48 (36-67)	61 (50-73)	30 (23-38)	346 (322-368)	152 (133-172)
1260	Laos	Male	70 to <75	41 (28-58)	81 (68-97)	40 (33-47)	529 (490-560)	230 (203-260)
1261	Latvia	Female	40 to 44	49 (38-61)	22 (19-26)	4 (3-5)	18 (15-22)	4 (3-5)
1262	Latvia	Female	45 to 49	46 (32-65)	36 (31-42)	6 (4-8)	28 (23-33)	6 (5-8)
1263	Latvia	Female	50 to 54	44 (31-61)	52 (45-62)	8 (6-10)	40 (34-47)	12 (9-15)
1264	Latvia	Female	55 to 59	45 (32-64)	72 (62-85)	11 (8-14)	61 (51-72)	22 (18-26)
1265	Latvia	Female	60 to 64	40 (26-61)	94 (83-109)	15 (12-18)	92 (77-108)	39 (33-47)
1266	Latvia	Female	65 to 69	30 (21-45)	109 (95-125)	20 (15-25)	133 (112-157)	67 (55-80)
1267	Latvia	Female	70 to <75	22 (14-36)	122 (106-141)	26 (20-31)	211 (176-249)	120 (98-143)
1268	Latvia	Male	40 to 44	61 (48-74)	14 (11-16)	6 (4-7)	52 (43-62)	14 (11-18)
1269	Latvia	Male	45 to 49	54 (40-75)	26 (23-31)	9 (7-12)	80 (66-95)	27 (21-33)
1270	Latvia	Male	50 to 54	47 (31-67)	52 (45-59)	14 (11-16)	121 (100-142)	47 (38-57)
1271	Latvia	Male	55 to 59	39 (26-54)	90 (78-101)	18 (14-23)	178 (149-207)	76 (63-90)
1272	Latvia	Male	60 to 64	32 (20-47)	143 (123-161)	23 (18-28)	263 (223-302)	125 (105-145)
1273	Latvia	Male	65 to 69	25 (17-37)	206 (177-234)	28 (21-34)	362 (309-414)	186 (157-215)
1274	Latvia	Male	70 to <75	19 (12-30)	256 (215-292)	33 (27-39)	491 (420-560)	267 (227-308)
1275	Lebanon	Female	40 to 44	51 (39-66)	27 (22-34)	2 (1-3)	11 (8-13)	2 (2-3)
1276	Lebanon	Female	45 to 49	59 (42-79)	39 (31-50)	3 (2-4)	16 (13-19)	4 (3-5)
1277	Lebanon	Female	50 to 54	65 (49-85)	47 (38-62)	4 (3-5)	25 (20-30)	7 (5-8)
1278	Lebanon	Female	55 to 59	68 (50-90)	57 (46-72)	5 (4-7)	37 (31-45)	12 (9-15)
1279	Lebanon	Female	60 to 64	65 (45-88)	69 (53-89)	8 (6-10)	61 (52-74)	22 (17-27)
1280	Lebanon	Female	65 to 69	54 (39-76)	79 (63-98)	11 (8-14)	102 (86-123)	40 (32-49)
1281	Lebanon	Female	70 to <75	46 (32-66)	103 (81-126)	16 (12-19)	179 (149-217)	75 (60-95)
1282	Lebanon	Male	40 to 44	56 (41-74)	14 (12-17)	2 (1-2)	15 (12-18)	4 (3-5)
1283	Lebanon	Male	45 to 49	73 (52-101)	24 (21-27)	3 (2-4)	22 (18-27)	7 (5-9)
1284	Lebanon	Male	50 to 54	87 (64-115)	39 (35-44)	4 (3-5)	35 (29-42)	13 (10-16)
1285	Lebanon	Male	55 to 59	96 (68-134)	58 (52-64)	5 (4-7)	54 (45-66)	20 (16-25)
1286	Lebanon	Male	60 to 64	97 (68-136)	90 (79-103)	6 (4-8)	91 (77-110)	36 (30-45)
1287	Lebanon	Male	65 to 69	88 (63-122)	131 (114-149)	7 (5-9)	145 (122-177)	58 (47-71)
1288	Lebanon	Male	70 to <75	74 (49-108)	185 (160-211)	9 (7-11)	237 (198-291)	93 (75-117)
1289	Lesotho	Female	40 to 44	68 (52-87)	20 (12-27)	2 (2-3)	201 (156-248)	7 (3-13)
1290	Lesotho	Female	45 to 49	96 (64-135)	27 (17-36)	4 (2-5)	195 (141-256)	15 (7-25)
1291	Lesotho	Female	50 to 54	116 (87-152)	35 (24-44)	6 (4-7)	208 (137-283)	27 (13-44)
1292	Lesotho	Female	55 to 59	126 (85-181)	45 (33-56)	8 (6-11)	226 (131-318)	45 (22-69)
1293	Lesotho	Female	60 to 64	137 (96-188)	50 (38-62)	14 (11-17)	300 (175-416)	89 (46-131)
1294	Lesotho	Female	65 to 69	148 (109-194)	62 (47-75)	22 (16-28)	438 (248-609)	145 (80-216)
1295	Lesotho	Female	70 to <75	129 (88-179)	72 (54-86)	32 (25-39)	666 (387-927)	246 (136-356)
1296	Lesotho	Male	40 to 44	88 (65-114)	11 (9-14)	3 (2-3)	303 (250-358)	16 (10-25)
1297	Lesotho	Male	45 to 49	127 (84-175)	24 (19-30)	5 (4-7)	344 (266-427)	30 (18-46)
1298	Lesotho	Male	50 to 54	145 (108-188)	43 (35-52)	8 (6-10)	399 (299-500)	54 (33-76)
1299	Lesotho	Male	55 to 59	144 (101-199)	65 (53-78)	12 (9-15)	451 (339-557)	80 (55-111)
1300	Lesotho	Male	60 to 64	141 (96-200)	80 (65-97)	17 (14-21)	569 (449-688)	138 (100-182)
1301	Lesotho	Male	65 to 69	137 (98-186)	106 (89-129)	25 (19-32)	742 (611-876)	194 (146-244)
1302	Lesotho	Male	70 to <75	113 (73-161)	129 (105-162)	36 (29-42)	1015 (870-1162)	274 (207-342)
1303	Liberia	Female	40 to 44	35 (26-45)	13 (10-16)	2 (2-3)	59 (52-67)	5 (4-7)
1304	Liberia	Female	45 to 49	52 (36-71)	18 (14-21)	4 (3-5)	77 (66-89)	10 (8-14)
1305	Liberia	Female	50 to 54	60 (44-78)	22 (18-27)	6 (5-7)	109 (92-125)	23 (17-30)
1306	Liberia	Female	55 to 59	57 (39-82)	28 (22-34)	9 (7-11)	154 (133-174)	40 (30-50)
1307	Liberia	Female	60 to 64	58 (40-82)	37 (31-45)	12 (10-15)	229 (204-254)	66 (51-81)
1308	Liberia	Female	65 to 69	63 (43-89)	42 (34-49)	17 (13-22)	355 (315-386)	116 (92-139)
1309	Liberia	Female	70 to <75	61 (42-86)	44 (38-51)	25 (20-29)	544 (497-586)	184 (148-217)
1310	Liberia	Male	40 to 44	28 (20-37)	6 (5-7)	2 (2-3)	63 (57-71)	7 (5-10)
1311	Liberia	Male	45 to 49	36 (25-49)	9 (7-11)	4 (3-6)	84 (73-94)	13 (10-18)
1312	Liberia	Male	50 to 54	41 (31-55)	14 (12-17)	7 (5-8)	112 (99-124)	23 (17-29)
1313	Liberia	Male	55 to 59	41 (30-56)	20 (17-23)	9 (7-12)	152 (137-167)	36 (29-45)
1314	Liberia	Male	60 to 64	43 (30-57)	30 (26-35)	13 (10-16)	228 (205-247)	63 (51-76)
1315	Liberia	Male	65 to 69	45 (31-64)	45 (39-52)	17 (13-22)	337 (307-364)	94 (77-110)
1316	Liberia	Male	70 to <75	42 (30-61)	64 (54-75)	23 (18-27)	516 (479-550)	154 (123-180)
1317	Libya	Female	40 to 44	72 (54-95)	17 (14-21)	2 (2-3)	17 (15-22)	5 (4-6)
1318	Libya	Female	45 to 49	95 (64-131)	25 (21-30)	4 (3-5)	27 (23-32)	8 (7-11)

				Diabetes type 2	Cancers	Hemorrhagic stroke	All-cause mortality	CVD mortality
ID	Country	Sex	Age	Rate (95% CI)	Rate (95% CI)	Rate (95% CI)	Rate (95% CI)	Rate (95% CI)
1319	Libya	Female	50 to 54	107 (78-143)	30 (26-35)	5 (4-6)	43 (36-51)	14 (11-18)
1320	Libya	Female	55 to 59	107 (75-146)	33 (29-40)	7 (5-9)	66 (55-78)	25 (20-31)
1321	Libya	Female	60 to 64	93 (62-136)	41 (36-47)	9 (7-12)	105 (90-121)	43 (35-52)
1322	Libya	Female	65 to 69	67 (47-93)	46 (41-51)	13 (10-18)	165 (145-186)	73 (62-85)
1323	Libya	Female	70 to <75	49 (32-73)	60 (54-66)	19 (15-23)	263 (237-297)	120 (103-140)
1324	Libya	Male	40 to 44	68 (49-90)	8 (7-9)	2 (2-3)	28 (23-34)	7 (5-9)
1325	Libya	Male	45 to 49	88 (60-123)	14 (13-16)	4 (3-5)	42 (35-49)	12 (9-16)
1326	Libya	Male	50 to 54	100 (72-133)	27 (24-30)	6 (4-7)	63 (53-75)	21 (16-26)
1327	Libya	Male	55 to 59	103 (75-139)	39 (34-46)	8 (6-11)	99 (80-117)	36 (28-45)
1328	Libya	Male	60 to 64	96 (66-136)	63 (56-70)	11 (9-14)	158 (132-184)	64 (52-76)
1329	Libya	Male	65 to 69	78 (56-112)	92 (81-106)	15 (11-20)	251 (204-288)	104 (84-124)
1330	Libya	Male	70 to <75	60 (38-93)	147 (128-170)	21 (17-26)	403 (325-453)	162 (127-191)
1331	Lithuania	Female	40 to 44	32 (26-39)	25 (22-30)	3 (3-4)	18 (16-20)	3 (2-3)
1332	Lithuania	Female	45 to 49	28 (20-39)	41 (35-47)	5 (4-7)	28 (25-31)	5 (4-6)
1333	Lithuania	Female	50 to 54	27 (19-38)	56 (49-64)	7 (6-9)	40 (36-44)	9 (8-11)
1334	Lithuania	Female	55 to 59	28 (20-39)	74 (65-85)	9 (7-12)	59 (53-66)	17 (14-20)
1335	Lithuania	Female	60 to 64	26 (18-37)	91 (81-103)	12 (10-15)	84 (76-93)	31 (27-35)
1336	Lithuania	Female	65 to 69	21 (15-30)	107 (95-120)	17 (13-21)	125 (112-138)	57 (50-63)
1337	Lithuania	Female	70 to <75	16 (11-25)	113 (100-127)	21 (17-25)	185 (166-205)	99 (88-111)
1338	Lithuania	Male	40 to 44	45 (36-54)	16 (13-19)	5 (4-6)	58 (52-64)	12 (10-15)
1339	Lithuania	Male	45 to 49	38 (28-50)	30 (26-35)	9 (6-11)	84 (76-94)	23 (19-27)
1340	Lithuania	Male	50 to 54	32 (22-45)	60 (50-68)	12 (10-14)	119 (107-132)	38 (33-44)
1341	Lithuania	Male	55 to 59	29 (20-40)	112 (94-127)	15 (12-19)	178 (161-196)	67 (59-76)
1342	Lithuania	Male	60 to 64	25 (16-37)	179 (147-211)	19 (15-23)	261 (239-285)	111 (100-126)
1343	Lithuania	Male	65 to 69	20 (14-30)	266 (209-316)	24 (18-29)	360 (330-391)	169 (152-187)
1344	Lithuania	Male	70 to <75	16 (10-25)	318 (240-384)	27 (22-33)	446 (409-485)	226 (204-252)
1345	Luxembourg	Female	40 to 44	28 (21-37)	34 (30-39)	2 (2-3)	8 (6-9)	1 (1-1)
1346	Luxembourg	Female	45 to 49	29 (20-41)	57 (49-65)	3 (2-4)	15 (12-17)	2 (1-2)
1347	Luxembourg	Female	50 to 54	39 (29-52)	73 (65-83)	4 (3-5)	24 (20-28)	3 (2-4)
1348	Luxembourg	Female	55 to 59	56 (41-77)	97 (86-110)	5 (4-6)	35 (29-40)	5 (4-6)
1349	Luxembourg	Female	60 to 64	62 (46-83)	123 (110-138)	7 (5-8)	56 (46-64)	10 (8-12)
1350	Luxembourg	Female	65 to 69	57 (40-76)	143 (128-158)	9 (6-11)	86 (72-100)	18 (15-22)
1351	Luxembourg	Female	70 to <75	46 (31-65)	159 (144-177)	16 (13-19)	137 (114-161)	39 (32-47)
1352	Luxembourg	Male	40 to 44	34 (26-44)	21 (18-24)	2 (2-3)	12 (10-15)	2 (1-2)
1353	Luxembourg	Male	45 to 49	41 (28-58)	33 (28-39)	3 (2-4)	20 (17-23)	4 (3-5)
1354	Luxembourg	Male	50 to 54	53 (40-69)	59 (52-68)	5 (4-6)	35 (30-41)	7 (6-9)
1355	Luxembourg	Male	55 to 59	69 (49-91)	94 (83-109)	7 (5-9)	56 (48-65)	13 (10-16)
1356	Luxembourg	Male	60 to 64	74 (54-96)	146 (130-176)	10 (9-12)	91 (79-103)	23 (19-28)
1357	Luxembourg	Male	65 to 69	68 (49-91)	209 (183-263)	15 (12-19)	140 (123-157)	38 (32-45)
1358	Luxembourg	Male	70 to <75	55 (35-78)	288 (251-345)	23 (19-27)	226 (199-254)	69 (59-80)
1359	Macedonia	Female	40 to 44	49 (37-61)	22 (18-27)	4 (3-4)	12 (11-14)	4 (3-4)
1360	Macedonia	Female	45 to 49	55 (36-77)	35 (29-42)	6 (5-8)	22 (20-24)	7 (6-8)
1361	Macedonia	Female	50 to 54	66 (49-86)	47 (39-55)	9 (7-11)	36 (33-40)	13 (11-15)
1362	Macedonia	Female	55 to 59	80 (58-110)	59 (51-70)	12 (9-15)	59 (54-65)	23 (20-27)
1363	Macedonia	Female	60 to 64	80 (57-112)	73 (63-85)	17 (13-20)	100 (91-108)	46 (40-51)
1364	Macedonia	Female	65 to 69	64 (47-85)	87 (77-99)	24 (18-31)	176 (161-191)	95 (85-106)
1365	Macedonia	Female	70 to <75	47 (30-70)	99 (87-114)	37 (31-44)	327 (298-353)	202 (183-219)
1366	Macedonia	Male	40 to 44	69 (55-86)	12 (11-14)	5 (4-6)	23 (21-26)	9 (7-10)
1367	Macedonia	Male	45 to 49	76 (51-116)	25 (22-29)	9 (7-11)	44 (39-49)	18 (15-20)
1368	Macedonia	Male	50 to 54	79 (54-115)	43 (38-49)	13 (11-16)	75 (67-82)	31 (27-36)
1369	Macedonia	Male	55 to 59	76 (56-101)	71 (63-80)	18 (13-23)	119 (108-130)	51 (44-58)
1370	Macedonia	Male	60 to 64	66 (44-94)	109 (96-122)	24 (20-30)	193 (177-209)	88 (77-98)
1371	Macedonia	Male	65 to 69	47 (34-67)	150 (133-171)	34 (26-42)	310 (287-334)	157 (141-173)
1372	Macedonia	Male	70 to <75	33 (21-52)	188 (165-214)	48 (40-57)	505 (467-542)	282 (257-308)
1373	Madagascar	Female	40 to 44	26 (19-33)	16 (12-20)	3 (2-4)	51 (37-70)	11 (7-17)
1374	Madagascar	Female	45 to 49	30 (21-42)	22 (17-27)	5 (4-6)	72 (50-98)	21 (14-30)
1375	Madagascar	Female	50 to 54	36 (27-47)	29 (23-35)	7 (6-9)	108 (70-146)	36 (22-50)
1376	Madagascar	Female	55 to 59	44 (30-61)	32 (27-39)	10 (7-13)	159 (102-211)	60 (38-83)
1377	Madagascar	Female	60 to 64	50 (36-67)	42 (35-50)	15 (12-18)	240 (158-307)	97 (62-128)
1378	Madagascar	Female	65 to 69	53 (38-73)	48 (41-56)	23 (17-30)	372 (248-465)	164 (109-211)
1379	Madagascar	Female	70 to <75	51 (37-70)	49 (42-55)	33 (27-40)	575 (403-713)	271 (190-342)
1380	Madagascar	Male	40 to 44	29 (22-38)	7 (6-8)	3 (3-4)	69 (49-90)	20 (13-28)
1381	Madagascar	Male	45 to 49	44 (31-60)	10 (9-12)	6 (4-8)	92 (67-122)	30 (20-41)
1382	Madagascar	Male	50 to 54	52 (39-67)	15 (13-18)	8 (7-10)	126 (92-170)	44 (31-59)
1383	Madagascar	Male	55 to 59	51 (36-68)	24 (20-28)	11 (8-15)	176 (130-234)	64 (45-85)
1384	Madagascar	Male	60 to 64	50 (36-69)	39 (30-47)	16 (13-19)	262 (201-343)	100 (75-130)
1385	Madagascar	Male	65 to 69	49 (36-68)	56 (42-70)	23 (17-29)	385 (301-491)	152 (114-190)
1386	Madagascar	Male	70 to <75	46 (33-65)	82 (58-104)	32 (26-37)	588 (484-737)	235 (187-295)
1387	Malawi	Female	40 to 44	24 (18-31)	28 (16-37)	1 (1-2)	79 (64-98)	4 (2-5)
1388	Malawi	Female	45 to 49	30 (20-41)	30 (20-38)	2 (2-3)	92 (69-120)	8 (5-11)

				Diabetes type 2	Cancers	Hemorrhagic stroke	All-cause mortality	CVD mortality
ID	Country	Sex	Age	Rate (95% CI)	Rate (95% CI)	Rate (95% CI)	Rate (95% CI)	Rate (95% CI)
1389	Malawi	Female	50 to 54	37 (28-48)	41 (30-49)	4 (3-5)	117 (83-154)	14 (9-21)
1390	Malawi	Female	55 to 59	47 (32-67)	38 (31-46)	6 (4-8)	145 (93-195)	28 (17-40)
1391	Malawi	Female	60 to 64	53 (38-74)	48 (40-56)	10 (8-12)	213 (137-280)	49 (30-66)
1392	Malawi	Female	65 to 69	58 (42-80)	45 (40-51)	15 (11-19)	324 (208-417)	93 (59-126)
1393	Malawi	Female	70 to <75	55 (39-76)	47 (42-52)	22 (18-27)	504 (340-634)	163 (108-214)
1394	Malawi	Male	40 to 44	33 (24-42)	17 (11-21)	2 (1-2)	129 (105-154)	7 (4-11)
1395	Malawi	Male	45 to 49	51 (35-69)	19 (15-23)	3 (2-4)	145 (115-179)	14 (9-20)
1396	Malawi	Male	50 to 54	60 (46-78)	27 (23-31)	5 (4-6)	169 (132-214)	22 (15-31)
1397	Malawi	Male	55 to 59	61 (44-85)	34 (30-39)	7 (5-9)	200 (157-257)	36 (26-49)
1398	Malawi	Male	60 to 64	61 (42-85)	45 (39-51)	10 (8-13)	278 (226-358)	64 (47-84)
1399	Malawi	Male	65 to 69	60 (41-82)	54 (49-61)	15 (11-20)	398 (330-507)	103 (78-134)
1400	Malawi	Male	70 to <75	55 (37-77)	75 (66-85)	21 (17-26)	601 (500-750)	166 (127-213)
1401	Malaysia	Female	40 to 44	60 (43-80)	18 (15-22)	3 (2-3)	13 (12-14)	3 (2-3)
1402	Malaysia	Female	45 to 49	79 (54-109)	27 (22-32)	5 (3-6)	22 (20-24)	6 (5-7)
1403	Malaysia	Female	50 to 54	95 (73-122)	33 (28-38)	7 (6-9)	36 (34-39)	10 (9-13)
1404	Malaysia	Female	55 to 59	109 (81-145)	39 (34-46)	9 (7-12)	59 (56-63)	19 (16-22)
1405	Malaysia	Female	60 to 64	102 (71-140)	46 (40-53)	13 (10-16)	97 (91-103)	35 (30-41)
1406	Malaysia	Female	65 to 69	74 (53-101)	57 (51-65)	17 (13-22)	174 (164-184)	72 (62-83)
1407	Malaysia	Female	70 to <75	52 (33-80)	70 (63-79)	26 (21-30)	303 (285-321)	133 (117-151)
1408	Malaysia	Male	40 to 44	57 (40-76)	8 (7-9)	3 (2-4)	31 (29-33)	9 (8-11)
1409	Malaysia	Male	45 to 49	58 (41-80)	12 (11-14)	5 (4-7)	47 (44-50)	17 (14-19)
1410	Malaysia	Male	50 to 54	61 (44-83)	20 (18-23)	8 (7-10)	74 (68-79)	30 (26-34)
1411	Malaysia	Male	55 to 59	66 (47-89)	32 (29-36)	12 (9-16)	115 (106-124)	49 (43-56)
1412	Malaysia	Male	60 to 64	63 (44-87)	50 (45-56)	17 (13-20)	178 (165-191)	76 (67-85)
1413	Malaysia	Male	65 to 69	53 (38-76)	75 (67-85)	22 (17-27)	289 (267-309)	126 (111-141)
1414	Malaysia	Male	70 to <75	43 (29-65)	103 (92-117)	29 (23-34)	431 (401-459)	181 (160-202)
1415	Maldives	Female	40 to 44	43 (33-56)	7 (6-8)	2 (1-3)	8 (5-12)	2 (1-2)
1416	Maldives	Female	45 to 49	55 (38-75)	10 (9-12)	3 (2-5)	12 (9-17)	3 (2-4)
1417	Maldives	Female	50 to 54	66 (51-86)	16 (14-19)	5 (4-6)	25 (18-34)	7 (4-10)
1418	Maldives	Female	55 to 59	78 (56-104)	20 (18-23)	6 (5-9)	37 (27-51)	10 (7-15)
1419	Maldives	Female	60 to 64	75 (53-100)	28 (24-31)	9 (7-11)	74 (54-100)	24 (17-34)
1420	Maldives	Female	65 to 69	57 (42-77)	34 (30-38)	12 (9-16)	127 (92-172)	48 (34-67)
1421	Maldives	Female	70 to <75	43 (28-63)	39 (35-44)	18 (15-22)	226 (162-309)	86 (61-121)
1422	Maldives	Male	40 to 44	41 (30-54)	3 (3-4)	2 (2-3)	13 (9-18)	5 (3-7)
1423	Maldives	Male	45 to 49	43 (30-58)	6 (5-7)	4 (3-5)	22 (16-30)	9 (6-13)
1424	Maldives	Male	50 to 54	47 (36-63)	11 (10-12)	6 (5-8)	41 (29-55)	18 (13-25)
1425	Maldives	Male	55 to 59	54 (38-71)	19 (17-21)	10 (7-13)	70 (51-93)	32 (23-43)
1426	Maldives	Male	60 to 64	54 (38-72)	32 (30-35)	13 (11-17)	122 (91-158)	56 (41-74)
1427	Maldives	Male	65 to 69	47 (34-64)	50 (45-55)	18 (13-23)	196 (147-253)	94 (70-122)
1428	Maldives	Male	70 to <75	40 (26-57)	67 (60-74)	24 (19-29)	330 (247-426)	164 (120-213)
1429	Mali	Female	40 to 44	31 (23-41)	14 (12-18)	3 (2-3)	48 (39-58)	8 (5-11)
1430	Mali	Female	45 to 49	43 (30-59)	19 (16-24)	4 (3-6)	63 (51-77)	13 (9-17)
1431	Mali	Female	50 to 54	50 (37-66)	24 (20-30)	6 (5-7)	87 (69-114)	21 (15-30)
1432	Mali	Female	55 to 59	51 (35-71)	29 (24-36)	8 (5-10)	127 (96-167)	33 (22-46)
1433	Mali	Female	60 to 64	53 (37-74)	39 (33-48)	11 (8-13)	195 (148-254)	60 (42-83)
1434	Mali	Female	65 to 69	57 (39-80)	40 (34-47)	15 (11-20)	305 (230-396)	107 (77-150)
1435	Mali	Female	70 to <75	55 (39-77)	38 (33-44)	23 (18-27)	489 (386-615)	190 (139-250)
1436	Mali	Male	40 to 44	24 (18-32)	10 (7-13)	2 (2-2)	62 (50-75)	5 (3-8)
1437	Mali	Male	45 to 49	31 (22-42)	15 (11-18)	4 (3-5)	77 (62-95)	9 (6-13)
1438	Mali	Male	50 to 54	35 (26-46)	20 (16-26)	5 (4-6)	98 (79-123)	14 (10-20)
1439	Mali	Male	55 to 59	37 (26-50)	27 (22-33)	7 (5-10)	128 (103-163)	23 (16-31)
1440	Mali	Male	60 to 64	39 (28-53)	40 (33-47)	10 (8-13)	193 (156-242)	41 (30-54)
1441	Mali	Male	65 to 69	41 (29-57)	47 (41-54)	15 (11-19)	284 (232-355)	66 (50-86)
1442	Mali	Male	70 to <75	39 (28-54)	58 (50-67)	20 (17-25)	454 (369-560)	126 (97-165)
1443	Malta	Female	40 to 44	27 (20-35)	20 (17-23)	2 (2-3)	6 (4-8)	1 (1-1)
1444	Malta	Female	45 to 49	29 (20-42)	31 (27-36)	4 (3-5)	11 (8-14)	2 (1-2)
1445	Malta	Female	50 to 54	40 (30-52)	45 (39-52)	5 (4-6)	18 (14-23)	3 (2-4)
1446	Malta	Female	55 to 59	59 (42-78)	59 (52-68)	6 (4-8)	30 (22-37)	6 (4-7)
1447	Malta	Female	60 to 64	65 (50-84)	80 (72-91)	7 (6-9)	51 (39-63)	13 (9-16)
1448	Malta	Female	65 to 69	59 (42-80)	90 (80-104)	10 (7-13)	73 (55-91)	21 (16-27)
1449	Malta	Female	70 to <75	48 (31-71)	113 (100-132)	18 (15-22)	129 (98-163)	46 (34-58)
1450	Malta	Male	40 to 44	35 (27-44)	12 (11-14)	3 (2-4)	13 (10-16)	3 (2-4)
1451	Malta	Male	45 to 49	42 (27-59)	19 (17-21)	5 (4-7)	18 (14-24)	5 (4-7)
1452	Malta	Male	50 to 54	55 (41-71)	35 (32-39)	7 (5-8)	33 (25-42)	11 (8-14)
1453	Malta	Male	55 to 59	74 (53-97)	63 (57-70)	8 (6-11)	56 (44-70)	19 (15-24)
1454	Malta	Male	60 to 64	79 (59-102)	112 (100-125)	12 (9-15)	94 (75-115)	32 (25-40)
1455	Malta	Male	65 to 69	71 (52-95)	169 (151-189)	17 (12-21)	151 (121-185)	55 (44-68)
1456	Malta	Male	70 to <75	56 (38-81)	224 (198-255)	28 (24-33)	239 (190-292)	88 (70-110)
1457	Marshall Islands	Female	40 to 44	758 (467-989)	18 (11-25)	4 (3-4)	60 (45-80)	12 (8-17)
1458	Marshall Islands	Female	45 to 49	898 (775-991)	22 (15-29)	6 (5-8)	86 (65-111)	17 (12-24)

				Diabetes type 2	Cancers	Hemorrhagic stroke	All-cause mortality	CVD mortality
ID	Country	Sex	Age	Rate (95% CI)	Rate (95% CI)	Rate (95% CI)	Rate (95% CI)	Rate (95% CI)
1459	Marshall Islands	Female	50 to 54	969 (898-997)	27 (19-34)	9 (7-11)	125 (97-159)	23 (17-32)
1460	Marshall Islands	Female	55 to 59	972 (918-997)	32 (23-41)	12 (9-16)	180 (142-228)	39 (29-53)
1461	Marshall Islands	Female	60 to 64	851 (740-982)	36 (28-44)	16 (13-20)	258 (205-321)	63 (47-82)
1462	Marshall Islands	Female	65 to 69	606 (344-967)	46 (36-54)	22 (16-28)	383 (305-474)	100 (75-128)
1463	Marshall Islands	Female	70 to <75	371 (106-734)	58 (48-68)	33 (26-39)	606 (483-751)	166 (127-213)
1464	Marshall Islands	Male	40 to 44	528 (402-682)	8 (6-10)	4 (4-5)	91 (68-114)	28 (20-38)
1465	Marshall Islands	Male	45 to 49	806 (717-876)	12 (10-14)	8 (6-10)	131 (98-164)	43 (30-57)
1466	Marshall Islands	Male	50 to 54	944 (855-993)	17 (14-21)	11 (9-14)	184 (141-231)	64 (47-83)
1467	Marshall Islands	Male	55 to 59	941 (834-993)	25 (21-29)	15 (11-20)	253 (201-310)	84 (63-107)
1468	Marshall Islands	Male	60 to 64	734 (631-786)	37 (31-43)	20 (16-25)	354 (291-424)	116 (89-147)
1469	Marshall Islands	Male	65 to 69	321 (269-391)	56 (48-65)	26 (19-33)	491 (413-573)	148 (117-185)
1470	Marshall Islands	Male	70 to <75	94 (54-159)	72 (63-83)	35 (29-42)	699 (600-799)	216 (174-262)
1471	Mauritania	Female	40 to 44	35 (27-46)	12 (10-15)	2 (2-3)	33 (25-43)	4 (2-6)
1472	Mauritania	Female	45 to 49	50 (34-70)	17 (14-20)	4 (3-5)	48 (36-63)	9 (5-13)
1473	Mauritania	Female	50 to 54	58 (42-78)	21 (18-25)	5 (4-6)	72 (53-97)	18 (12-26)
1474	Mauritania	Female	55 to 59	57 (39-81)	26 (22-31)	7 (5-10)	109 (76-143)	31 (20-44)
1475	Mauritania	Female	60 to 64	58 (40-82)	35 (30-41)	10 (8-13)	169 (120-215)	54 (35-73)
1476	Mauritania	Female	65 to 69	61 (42-86)	38 (34-44)	14 (10-18)	264 (187-333)	94 (63-129)
1477	Mauritania	Female	70 to <75	58 (40-80)	42 (37-48)	20 (15-24)	419 (308-507)	157 (109-204)
1478	Mauritania	Male	40 to 44	26 (19-35)	5 (4-7)	2 (1-2)	31 (23-41)	4 (1-7)
1479	Mauritania	Male	45 to 49	33 (23-46)	8 (6-10)	3 (2-5)	44 (32-57)	8 (3-12)
1480	Mauritania	Male	50 to 54	38 (28-52)	12 (10-15)	5 (4-6)	64 (47-82)	14 (8-20)
1481	Mauritania	Male	55 to 59	39 (27-54)	18 (14-22)	7 (5-9)	93 (69-122)	22 (14-32)
1482	Mauritania	Male	60 to 64	40 (28-56)	29 (23-34)	9 (7-12)	148 (111-188)	41 (27-57)
1483	Mauritania	Male	65 to 69	42 (29-58)	44 (35-52)	13 (9-17)	226 (171-282)	62 (43-83)
1484	Mauritania	Male	70 to <75	40 (27-54)	64 (50-77)	18 (14-22)	362 (275-448)	104 (73-139)
1485	Mauritius	Female	40 to 44	113 (78-152)	17 (15-20)	3 (2-3)	17 (14-22)	3 (3-5)
1486	Mauritius	Female	45 to 49	163 (96-278)	25 (22-30)	5 (3-6)	28 (22-34)	6 (5-8)
1487	Mauritius	Female	50 to 54	246 (190-332)	29 (26-33)	6 (5-8)	43 (35-52)	10 (8-13)
1488	Mauritius	Female	55 to 59	362 (275-445)	36 (32-41)	8 (6-11)	70 (56-84)	16 (12-20)
1489	Mauritius	Female	60 to 64	336 (219-429)	43 (38-48)	10 (8-13)	112 (91-134)	29 (22-36)
1490	Mauritius	Female	65 to 69	168 (126-218)	50 (45-55)	13 (10-17)	173 (142-208)	49 (39-61)
1491	Mauritius	Female	70 to <75	71 (41-131)	54 (50-59)	20 (16-24)	270 (224-327)	88 (71-107)
1492	Mauritius	Male	40 to 44	174 (116-236)	6 (5-6)	3 (2-4)	40 (33-48)	10 (8-13)
1493	Mauritius	Male	45 to 49	237 (112-391)	10 (9-11)	5 (4-7)	59 (48-71)	16 (13-21)
1494	Mauritius	Male	50 to 54	269 (177-396)	19 (17-21)	8 (6-9)	90 (74-107)	27 (21-33)
1495	Mauritius	Male	55 to 59	271 (178-390)	29 (27-32)	11 (8-14)	131 (109-154)	41 (33-50)
1496	Mauritius	Male	60 to 64	226 (104-367)	46 (42-51)	15 (12-19)	200 (168-232)	64 (53-77)
1497	Mauritius	Male	65 to 69	133 (75-190)	69 (63-76)	20 (15-25)	299 (253-345)	100 (84-120)
1498	Mauritius	Male	70 to <75	75 (43-124)	99 (87-110)	28 (23-33)	450 (383-517)	160 (133-188)
1499	Mexico	Female	40 to 44	81 (61-104)	20 (17-24)	2 (2-3)	15 (15-16)	2 (2-2)
1500	Mexico	Female	45 to 49	95 (69-126)	30 (26-38)	3 (3-5)	25 (24-26)	4 (3-4)
1501	Mexico	Female	50 to 54	104 (78-134)	40 (35-48)	5 (4-6)	42 (40-44)	6 (6-7)
1502	Mexico	Female	55 to 59	110 (83-142)	52 (44-63)	6 (5-8)	66 (64-68)	11 (10-11)
1503	Mexico	Female	60 to 64	100 (72-134)	65 (56-79)	9 (7-11)	104 (100-107)	19 (18-20)
1504	Mexico	Female	65 to 69	75 (54-109)	84 (73-98)	12 (9-16)	169 (163-175)	36 (35-38)
1505	Mexico	Female	70 to <75	60 (39-91)	98 (83-120)	17 (14-20)	247 (238-255)	61 (58-64)
1506	Mexico	Male	40 to 44	125 (98-158)	10 (8-13)	2 (1-2)	34 (33-36)	4 (4-5)
1507	Mexico	Male	45 to 49	160 (120-206)	16 (12-23)	3 (2-4)	48 (46-49)	7 (7-8)
1508	Mexico	Male	50 to 54	161 (119-206)	28 (23-36)	5 (4-6)	72 (69-75)	13 (12-14)
1509	Mexico	Male	55 to 59	129 (91-172)	46 (37-60)	6 (5-8)	102 (99-106)	21 (19-22)
1510	Mexico	Male	60 to 64	102 (66-146)	77 (64-96)	9 (7-11)	144 (139-149)	32 (30-33)
1511	Mexico	Male	65 to 69	80 (55-121)	121 (102-147)	12 (9-15)	220 (213-226)	53 (51-56)
1512	Mexico	Male	70 to <75	70 (45-107)	167 (140-204)	16 (13-18)	323 (314-333)	84 (80-87)
1513	Moldova	Female	40 to 44	50 (41-61)	15 (13-18)	3 (2-4)	20 (17-24)	4 (3-5)
1514	Moldova	Female	45 to 49	42 (31-56)	22 (20-27)	5 (4-6)	32 (27-38)	7 (6-9)
1515	Moldova	Female	50 to 54	39 (27-53)	30 (26-35)	8 (6-9)	50 (42-58)	14 (11-17)
1516	Moldova	Female	55 to 59	40 (29-53)	40 (35-47)	11 (8-15)	81 (68-94)	29 (24-35)
1517	Moldova	Female	60 to 64	35 (25-50)	55 (49-64)	17 (14-21)	137 (117-158)	64 (54-76)
1518	Moldova	Female	65 to 69	26 (19-37)	67 (60-75)	24 (18-30)	229 (194-264)	133 (112-155)
1519	Moldova	Female	70 to <75	19 (13-30)	71 (62-83)	31 (25-37)	361 (305-417)	244 (206-283)
1520	Moldova	Male	40 to 44	56 (45-68)	11 (10-13)	5 (4-7)	60 (50-71)	14 (10-17)
1521	Moldova	Male	45 to 49	48 (35-65)	22 (19-24)	10 (7-13)	93 (77-110)	25 (20-31)
1522	Moldova	Male	50 to 54	42 (30-58)	39 (36-44)	14 (12-17)	135 (112-159)	42 (33-52)
1523	Moldova	Male	55 to 59	38 (27-51)	65 (59-72)	19 (15-25)	195 (164-227)	71 (57-84)
1524	Moldova	Male	60 to 64	33 (22-48)	95 (88-104)	26 (21-33)	292 (249-336)	132 (112-152)
1525	Moldova	Male	65 to 69	26 (19-38)	119 (109-130)	35 (28-43)	419 (360-479)	228 (195-261)
1526	Moldova	Male	70 to <75	20 (13-32)	131 (118-149)	41 (33-49)	584 (504-666)	368 (318-420)
1527	Mongolia	Female	40 to 44	20 (16-26)	18 (15-21)	6 (5-7)	32 (25-40)	10 (7-13)
1528	Mongolia	Female	45 to 49	21 (15-29)	26 (22-31)	11 (9-15)	49 (38-60)	20 (15-24)

				Diabetes type 2	Cancers	Hemorrhagic stroke	All-cause mortality	CVD mortality
ID	Country	Sex	Age	Rate (95% CI)	Rate (95% CI)	Rate (95% CI)	Rate (95% CI)	Rate (95% CI)
1529	Mongolia	Female	50 to 54	22 (16-30)	36 (30-42)	18 (15-21)	75 (61-90)	33 (26-40)
1530	Mongolia	Female	55 to 59	23 (17-30)	50 (41-61)	25 (19-32)	110 (91-131)	47 (37-58)
1531	Mongolia	Female	60 to 64	22 (16-30)	77 (57-96)	32 (26-39)	165 (141-192)	69 (55-83)
1532	Mongolia	Female	65 to 69	20 (15-27)	104 (76-129)	39 (30-48)	251 (216-292)	114 (93-137)
1533	Mongolia	Female	70 to <75	17 (12-24)	132 (91-165)	50 (42-59)	389 (330-456)	199 (166-238)
1534	Mongolia	Male	40 to 44	29 (23-36)	15 (12-19)	9 (8-11)	85 (68-104)	23 (17-30)
1535	Mongolia	Male	45 to 49	27 (19-35)	32 (26-39)	17 (13-22)	127 (101-154)	43 (33-55)
1536	Mongolia	Male	50 to 54	25 (18-33)	61 (49-76)	27 (22-32)	181 (147-219)	72 (56-90)
1537	Mongolia	Male	55 to 59	23 (16-31)	92 (75-111)	38 (30-48)	254 (210-301)	111 (89-136)
1538	Mongolia	Male	60 to 64	22 (15-32)	132 (111-156)	48 (40-59)	347 (294-406)	163 (133-197)
1539	Mongolia	Male	65 to 69	23 (16-32)	177 (151-206)	57 (44-71)	471 (403-542)	232 (193-271)
1540	Mongolia	Male	70 to <75	21 (14-29)	229 (197-267)	74 (61-88)	654 (568-742)	341 (290-398)
1541	Montenegro	Female	40 to 44	43 (34-54)	24 (20-29)	4 (4-5)	14 (12-16)	4 (3-5)
1542	Montenegro	Female	45 to 49	42 (30-58)	36 (30-44)	8 (6-10)	24 (21-27)	7 (5-9)
1543	Montenegro	Female	50 to 54	45 (32-60)	47 (40-56)	11 (9-13)	37 (32-43)	12 (10-15)
1544	Montenegro	Female	55 to 59	50 (36-68)	56 (48-65)	14 (11-19)	56 (48-63)	21 (17-26)
1545	Montenegro	Female	60 to 64	50 (35-70)	68 (58-79)	21 (17-26)	86 (74-96)	40 (33-46)
1546	Montenegro	Female	65 to 69	44 (32-62)	74 (65-86)	31 (23-39)	141 (118-162)	78 (65-92)
1547	Montenegro	Female	70 to <75	36 (24-52)	81 (71-96)	49 (41-57)	241 (200-283)	151 (124-178)
1548	Montenegro	Male	40 to 44	62 (49-77)	11 (10-13)	6 (5-7)	28 (25-30)	9 (8-11)
1549	Montenegro	Male	45 to 49	54 (39-72)	22 (19-26)	9 (7-12)	45 (40-49)	17 (14-20)
1550	Montenegro	Male	50 to 54	51 (37-70)	39 (34-45)	13 (11-16)	68 (61-80)	27 (23-33)
1551	Montenegro	Male	55 to 59	53 (37-72)	65 (57-75)	20 (15-25)	111 (99-127)	48 (41-57)
1552	Montenegro	Male	60 to 64	49 (32-67)	99 (86-114)	28 (23-35)	170 (151-196)	79 (68-92)
1553	Montenegro	Male	65 to 69	37 (27-53)	132 (115-155)	40 (31-51)	252 (222-289)	129 (112-151)
1554	Montenegro	Male	70 to <75	28 (18-43)	163 (140-193)	60 (49-71)	379 (332-433)	212 (183-246)
1555	Morocco	Female	40 to 44	80 (60-103)	16 (11-20)	2 (2-3)	19 (16-22)	4 (3-6)
1556	Morocco	Female	45 to 49	104 (71-143)	23 (17-29)	3 (2-5)	30 (26-34)	8 (6-10)
1557	Morocco	Female	50 to 54	120 (90-156)	28 (21-33)	5 (4-6)	47 (41-53)	15 (12-19)
1558	Morocco	Female	55 to 59	128 (93-175)	33 (26-39)	6 (5-9)	73 (65-80)	27 (22-32)
1559	Morocco	Female	60 to 64	117 (81-166)	33 (28-39)	9 (7-12)	115 (106-126)	48 (38-57)
1560	Morocco	Female	65 to 69	87 (62-121)	34 (31-38)	14 (10-18)	180 (161-200)	81 (66-97)
1561	Morocco	Female	70 to <75	65 (42-98)	35 (31-42)	19 (15-24)	287 (250-329)	136 (105-167)
1562	Morocco	Male	40 to 44	64 (48-82)	6 (5-7)	2 (2-3)	28 (24-31)	7 (5-10)
1563	Morocco	Male	45 to 49	81 (55-110)	11 (10-13)	3 (3-5)	40 (35-45)	13 (9-16)
1564	Morocco	Male	50 to 54	92 (68-121)	20 (17-22)	5 (4-7)	60 (54-67)	22 (18-27)
1565	Morocco	Male	55 to 59	99 (72-133)	30 (26-33)	8 (6-11)	92 (82-101)	36 (29-42)
1566	Morocco	Male	60 to 64	95 (66-129)	38 (32-43)	11 (9-14)	145 (133-156)	61 (49-71)
1567	Morocco	Male	65 to 69	82 (60-114)	48 (41-54)	15 (11-19)	219 (202-236)	99 (82-113)
1568	Morocco	Male	70 to <75	67 (46-100)	54 (48-61)	21 (17-25)	342 (316-369)	155 (128-176)
1569	Mozambique	Female	40 to 44	25 (19-33)	12 (9-18)	2 (1-2)	78 (65-95)	4 (3-6)
1570	Mozambique	Female	45 to 49	30 (21-41)	17 (13-24)	3 (2-4)	85 (68-110)	9 (6-12)
1571	Mozambique	Female	50 to 54	36 (27-49)	24 (18-33)	5 (4-6)	102 (81-134)	15 (10-21)
1572	Mozambique	Female	55 to 59	45 (32-63)	27 (21-37)	7 (5-9)	126 (93-166)	28 (19-40)
1573	Mozambique	Female	60 to 64	51 (37-67)	38 (29-48)	11 (9-14)	186 (139-241)	49 (34-67)
1574	Mozambique	Female	65 to 69	54 (39-73)	43 (34-53)	18 (13-23)	285 (209-368)	95 (66-127)
1575	Mozambique	Female	70 to <75	52 (38-70)	49 (38-59)	26 (21-31)	451 (339-561)	175 (131-226)
1576	Mozambique	Male	40 to 44	34 (26-45)	5 (5-6)	2 (2-3)	138 (118-159)	10 (6-14)
1577	Mozambique	Male	45 to 49	52 (36-72)	8 (7-10)	5 (3-6)	147 (119-180)	17 (12-24)
1578	Mozambique	Male	50 to 54	61 (46-79)	14 (12-16)	7 (6-9)	163 (132-203)	28 (20-38)
1579	Mozambique	Male	55 to 59	63 (44-86)	23 (20-26)	10 (7-13)	195 (157-240)	45 (33-59)
1580	Mozambique	Male	60 to 64	62 (44-86)	33 (29-37)	15 (12-18)	273 (225-334)	84 (65-108)
1581	Mozambique	Male	65 to 69	61 (43-83)	46 (41-52)	22 (17-28)	393 (326-475)	132 (105-165)
1582	Mozambique	Male	70 to <75	56 (39-78)	65 (59-74)	29 (24-34)	592 (502-707)	198 (160-245)
1583	Myanmar	Female	40 to 44	48 (35-60)	12 (10-14)	2 (2-3)	27 (25-30)	3 (2-4)
1584	Myanmar	Female	45 to 49	56 (40-76)	17 (14-20)	4 (3-6)	41 (37-46)	6 (5-8)
1585	Myanmar	Female	50 to 54	67 (51-85)	21 (19-24)	7 (5-8)	63 (58-70)	12 (10-16)
1586	Myanmar	Female	55 to 59	79 (59-104)	27 (24-31)	10 (7-13)	95 (87-105)	21 (16-26)
1587	Myanmar	Female	60 to 64	75 (55-101)	33 (29-36)	14 (11-17)	148 (136-161)	36 (29-44)
1588	Myanmar	Female	65 to 69	56 (42-74)	39 (35-43)	18 (14-23)	232 (214-252)	68 (56-80)
1589	Myanmar	Female	70 to <75	41 (27-59)	45 (41-49)	28 (23-33)	372 (344-399)	119 (100-138)
1590	Myanmar	Male	40 to 44	48 (35-63)	7 (6-8)	3 (3-4)	56 (49-64)	8 (6-11)
1591	Myanmar	Male	45 to 49	49 (33-68)	12 (10-14)	6 (4-8)	76 (68-87)	14 (10-18)
1592	Myanmar	Male	50 to 54	55 (39-73)	20 (17-23)	10 (8-12)	109 (99-123)	26 (20-33)
1593	Myanmar	Male	55 to 59	64 (45-85)	32 (28-37)	16 (12-20)	157 (146-179)	43 (35-52)
1594	Myanmar	Male	60 to 64	65 (46-88)	49 (43-55)	22 (18-27)	238 (222-266)	67 (56-79)
1595	Myanmar	Male	65 to 69	56 (41-77)	73 (65-83)	29 (22-37)	359 (335-395)	109 (93-128)
1596	Myanmar	Male	70 to <75	47 (32-68)	97 (87-110)	41 (33-48)	555 (513-606)	176 (152-205)
1597	Namibia	Female	40 to 44	38 (30-49)	11 (7-13)	2 (1-2)	75 (54-102)	3 (1-5)
1598	Namibia	Female	45 to 49	52 (37-70)	15 (11-18)	3 (2-4)	77 (52-108)	6 (2-10)

				Diabetes type 2	Cancers	Hemorrhagic stroke	All-cause mortality	CVD mortality
ID	Country	Sex	Age	Rate (95% CI)	Rate (95% CI)	Rate (95% CI)	Rate (95% CI)	Rate (95% CI)
1599	Namibia	Female	50 to 54	62 (48-80)	19 (14-23)	4 (3-5)	88 (51-126)	11 (4-19)
1600	Namibia	Female	55 to 59	70 (49-97)	24 (18-31)	6 (4-9)	97 (45-147)	20 (7-34)
1601	Namibia	Female	60 to 64	80 (59-108)	27 (21-33)	10 (8-13)	137 (64-202)	42 (17-65)
1602	Namibia	Female	65 to 69	93 (69-122)	31 (26-37)	16 (11-22)	205 (93-303)	71 (30-111)
1603	Namibia	Female	70 to <75	86 (61-116)	34 (30-40)	23 (19-29)	323 (148-465)	123 (53-185)
1604	Namibia	Male	40 to 44	47 (36-59)	6 (4-8)	2 (1-2)	135 (108-165)	7 (4-10)
1605	Namibia	Male	45 to 49	65 (46-89)	10 (8-12)	3 (2-5)	151 (119-188)	14 (9-19)
1606	Namibia	Male	50 to 54	79 (60-104)	18 (14-21)	6 (4-7)	176 (136-222)	26 (17-36)
1607	Namibia	Male	55 to 59	89 (59-130)	28 (22-32)	8 (6-11)	206 (160-259)	43 (31-59)
1608	Namibia	Male	60 to 64	105 (78-140)	38 (31-44)	13 (10-16)	284 (228-348)	85 (63-110)
1609	Namibia	Male	65 to 69	125 (90-167)	52 (44-61)	19 (14-25)	407 (330-484)	135 (102-169)
1610	Namibia	Male	70 to <75	115 (79-157)	66 (57-75)	27 (22-33)	612 (494-710)	211 (163-257)
1611	Nepal	Female	40 to 44	59 (45-77)	9 (8-11)	2 (1-2)	30 (27-33)	6 (4-7)
1612	Nepal	Female	45 to 49	71 (51-97)	14 (12-16)	3 (2-4)	45 (40-49)	10 (8-13)
1613	Nepal	Female	50 to 54	73 (53-100)	19 (17-22)	5 (4-6)	70 (61-78)	18 (14-22)
1614	Nepal	Female	55 to 59	66 (45-92)	23 (20-27)	8 (6-10)	107 (94-117)	32 (25-39)
1615	Nepal	Female	60 to 64	57 (37-86)	27 (23-31)	11 (9-14)	166 (152-179)	53 (42-64)
1616	Nepal	Female	65 to 69	48 (32-74)	30 (26-35)	16 (12-20)	262 (242-282)	89 (71-107)
1617	Nepal	Female	70 to <75	49 (33-72)	34 (29-39)	21 (17-25)	412 (386-436)	133 (106-157)
1618	Nepal	Male	40 to 44	53 (40-68)	4 (4-5)	2 (2-3)	40 (37-45)	10 (8-13)
1619	Nepal	Male	45 to 49	53 (37-71)	7 (6-9)	4 (3-5)	58 (52-64)	19 (15-23)
1620	Nepal	Male	50 to 54	53 (39-73)	13 (10-15)	6 (5-7)	84 (74-94)	33 (27-39)
1621	Nepal	Male	55 to 59	56 (40-76)	20 (16-23)	9 (7-12)	126 (112-139)	52 (43-61)
1622	Nepal	Male	60 to 64	52 (35-74)	28 (23-33)	14 (11-17)	194 (170-215)	84 (71-97)
1623	Nepal	Male	65 to 69	41 (28-59)	37 (31-43)	20 (15-25)	295 (258-329)	130 (110-152)
1624	Nepal	Male	70 to <75	38 (26-56)	43 (36-50)	24 (19-28)	452 (397-503)	196 (166-227)
1625	Netherlands	Female	40 to 44	29 (23-37)	35 (30-41)	2 (2-3)	9 (7-10)	1 (1-1)
1626	Netherlands	Female	45 to 49	32 (22-43)	53 (47-61)	4 (3-5)	15 (14-18)	2 (1-2)
1627	Netherlands	Female	50 to 54	41 (31-53)	75 (66-85)	5 (4-6)	26 (23-29)	3 (2-4)
1628	Netherlands	Female	55 to 59	56 (42-74)	95 (85-105)	5 (4-7)	39 (34-44)	4 (3-5)
1629	Netherlands	Female	60 to 64	65 (49-84)	120 (108-134)	7 (5-8)	58 (51-65)	8 (6-10)
1630	Netherlands	Female	65 to 69	67 (49-92)	148 (133-165)	9 (7-12)	87 (77-99)	14 (12-17)
1631	Netherlands	Female	70 to <75	60 (42-85)	173 (157-191)	15 (12-19)	141 (125-160)	30 (25-36)
1632	Netherlands	Male	40 to 44	38 (30-48)	19 (16-22)	2 (2-3)	11 (10-13)	2 (1-2)
1633	Netherlands	Male	45 to 49	47 (33-64)	30 (27-35)	4 (3-5)	18 (16-21)	4 (3-4)
1634	Netherlands	Male	50 to 54	59 (46-76)	53 (48-59)	5 (4-7)	32 (28-37)	7 (6-8)
1635	Netherlands	Male	55 to 59	75 (54-102)	93 (83-102)	6 (5-9)	54 (48-61)	11 (9-13)
1636	Netherlands	Male	60 to 64	82 (62-109)	156 (139-174)	9 (7-11)	88 (79-99)	19 (16-23)
1637	Netherlands	Male	65 to 69	80 (58-111)	246 (216-278)	13 (9-17)	146 (132-165)	34 (29-40)
1638	Netherlands	Male	70 to <75	68 (46-98)	332 (288-372)	21 (17-25)	247 (222-279)	63 (54-74)
1639	New Zealand	Female	40 to 44	15 (11-20)	37 (32-43)	2 (2-3)	10 (8-12)	1 (1-2)
1640	New Zealand	Female	45 to 49	21 (15-31)	57 (48-70)	3 (3-4)	16 (13-18)	2 (2-3)
1641	New Zealand	Female	50 to 54	27 (21-36)	76 (65-90)	4 (4-5)	24 (21-28)	4 (3-5)
1642	New Zealand	Female	55 to 59	32 (24-44)	94 (80-112)	5 (4-6)	35 (30-40)	5 (4-7)
1643	New Zealand	Female	60 to 64	35 (26-46)	128 (111-149)	7 (6-8)	56 (48-64)	10 (8-12)
1644	New Zealand	Female	65 to 69	35 (26-49)	168 (146-194)	10 (8-12)	89 (76-102)	17 (14-21)
1645	New Zealand	Female	70 to <75	30 (21-44)	202 (174-239)	17 (14-19)	146 (124-168)	36 (30-43)
1646	New Zealand	Male	40 to 44	21 (16-27)	22 (18-27)	2 (2-3)	15 (13-18)	4 (3-5)
1647	New Zealand	Male	45 to 49	31 (22-41)	37 (31-47)	3 (3-4)	23 (19-26)	6 (5-8)
1648	New Zealand	Male	50 to 54	41 (32-51)	66 (55-81)	5 (4-5)	34 (29-39)	10 (8-12)
1649	New Zealand	Male	55 to 59	50 (37-65)	115 (95-142)	6 (5-7)	53 (45-61)	16 (13-19)
1650	New Zealand	Male	60 to 64	50 (37-65)	188 (157-231)	9 (7-10)	81 (70-93)	23 (19-27)
1651	New Zealand	Male	65 to 69	41 (29-54)	291 (244-355)	12 (10-15)	135 (117-154)	39 (33-46)
1652	New Zealand	Male	70 to <75	31 (21-46)	379 (323-457)	21 (19-24)	227 (197-259)	70 (59-82)
1653	Nicaragua	Female	40 to 44	45 (34-59)	12 (10-15)	2 (2-3)	14 (10-18)	2 (2-3)
1654	Nicaragua	Female	45 to 49	49 (34-65)	18 (14-23)	4 (3-5)	21 (16-27)	4 (3-6)
1655	Nicaragua	Female	50 to 54	53 (39-70)	22 (18-28)	5 (4-7)	32 (25-40)	7 (5-9)
1656	Nicaragua	Female	55 to 59	58 (42-76)	28 (24-36)	7 (5-9)	51 (40-64)	12 (9-15)
1657	Nicaragua	Female	60 to 64	56 (41-75)	36 (31-44)	10 (8-12)	83 (65-102)	22 (17-28)
1658	Nicaragua	Female	65 to 69	49 (35-68)	42 (36-50)	13 (10-17)	110 (86-137)	33 (25-42)
1659	Nicaragua	Female	70 to <75	44 (31-62)	50 (41-62)	17 (13-21)	166 (129-208)	56 (42-71)
1660	Nicaragua	Male	40 to 44	56 (44-69)	5 (4-7)	2 (2-3)	35 (27-44)	4 (3-6)
1661	Nicaragua	Male	45 to 49	68 (49-90)	8 (7-12)	4 (3-5)	45 (35-57)	7 (4-9)
1662	Nicaragua	Male	50 to 54	71 (53-94)	14 (11-18)	5 (4-7)	61 (47-77)	11 (8-15)
1663	Nicaragua	Male	55 to 59	67 (50-91)	23 (18-29)	8 (6-10)	92 (72-114)	20 (15-26)
1664	Nicaragua	Male	60 to 64	60 (42-84)	36 (31-43)	10 (8-13)	135 (108-165)	36 (27-45)
1665	Nicaragua	Male	65 to 69	51 (36-75)	52 (44-61)	14 (10-18)	170 (136-207)	50 (39-63)
1666	Nicaragua	Male	70 to <75	47 (32-70)	71 (60-86)	18 (15-22)	254 (203-309)	83 (65-104)
1667	Niger	Female	40 to 44	27 (20-36)	11 (9-14)	2 (2-3)	51 (43-60)	5 (4-8)
1668	Niger	Female	45 to 49	37 (26-49)	15 (12-18)	4 (3-5)	66 (55-81)	10 (7-13)

				Diabetes type 2	Cancers	Hemorrhagic stroke	All-cause mortality	CVD mortality
ID	Country	Sex	Age	Rate (95% CI)	Rate (95% CI)	Rate (95% CI)	Rate (95% CI)	Rate (95% CI)
1669	Niger	Female	50 to 54	43 (33-55)	18 (15-21)	6 (5-7)	91 (73-118)	20 (15-29)
1670	Niger	Female	55 to 59	43 (30-60)	22 (18-26)	8 (6-11)	131 (102-172)	35 (24-49)
1671	Niger	Female	60 to 64	46 (31-63)	30 (25-36)	11 (9-14)	201 (155-260)	60 (42-81)
1672	Niger	Female	65 to 69	49 (34-67)	33 (28-38)	16 (11-20)	313 (241-407)	108 (77-143)
1673	Niger	Female	70 to <75	48 (33-66)	37 (31-42)	23 (18-28)	500 (400-623)	177 (130-233)
1674	Niger	Male	40 to 44	25 (19-32)	5 (4-6)	3 (2-3)	62 (51-76)	9 (6-13)
1675	Niger	Male	45 to 49	32 (23-44)	8 (6-9)	4 (3-6)	82 (67-101)	15 (10-21)
1676	Niger	Male	50 to 54	36 (27-49)	12 (10-15)	7 (5-8)	112 (91-136)	24 (18-33)
1677	Niger	Male	55 to 59	38 (26-52)	17 (15-20)	9 (6-12)	155 (125-189)	37 (27-49)
1678	Niger	Male	60 to 64	39 (27-53)	27 (23-31)	12 (9-15)	235 (193-278)	63 (47-81)
1679	Niger	Male	65 to 69	41 (30-58)	40 (34-46)	16 (12-21)	349 (290-408)	93 (72-117)
1680	Niger	Male	70 to <75	39 (28-54)	58 (49-68)	22 (18-27)	549 (467-621)	156 (123-193)
1681	Nigeria	Female	40 to 44	22 (17-29)	15 (11-18)	2 (1-2)	45 (37-56)	2 (1-3)
1682	Nigeria	Female	45 to 49	30 (21-41)	20 (14-24)	3 (2-4)	52 (40-66)	4 (3-6)
1683	Nigeria	Female	50 to 54	35 (26-45)	23 (17-27)	4 (3-5)	63 (47-79)	8 (5-11)
1684	Nigeria	Female	55 to 59	36 (25-50)	29 (22-34)	5 (4-7)	81 (61-102)	13 (9-18)
1685	Nigeria	Female	60 to 64	39 (28-53)	34 (28-40)	8 (6-10)	123 (93-153)	27 (18-36)
1686	Nigeria	Female	65 to 69	43 (30-58)	38 (31-43)	12 (8-15)	193 (145-236)	52 (36-69)
1687	Nigeria	Female	70 to <75	43 (31-58)	34 (31-39)	18 (14-21)	322 (243-391)	100 (69-131)
1688	Nigeria	Male	40 to 44	18 (14-24)	6 (4-9)	2 (1-2)	58 (49-69)	3 (2-5)
1689	Nigeria	Male	45 to 49	23 (17-31)	9 (7-12)	3 (2-4)	69 (56-84)	6 (3-9)
1690	Nigeria	Male	50 to 54	27 (20-35)	14 (11-19)	5 (4-6)	83 (67-102)	10 (6-14)
1691	Nigeria	Male	55 to 59	28 (20-39)	23 (16-28)	6 (4-8)	102 (81-127)	15 (10-21)
1692	Nigeria	Male	60 to 64	30 (22-41)	41 (25-54)	9 (7-11)	152 (121-187)	30 (20-41)
1693	Nigeria	Male	65 to 69	32 (23-44)	65 (39-85)	13 (9-16)	223 (179-273)	47 (33-64)
1694	Nigeria	Male	70 to <75	32 (23-43)	83 (56-106)	17 (13-21)	354 (281-434)	82 (58-110)
1695	North Korea	Female	40 to 44	34 (26-45)	13 (10-16)	4 (4-5)	26 (23-30)	7 (5-9)
1696	North Korea	Female	45 to 49	50 (36-66)	19 (15-23)	8 (6-11)	40 (35-45)	12 (9-15)
1697	North Korea	Female	50 to 54	58 (45-74)	26 (21-31)	13 (10-15)	61 (54-69)	20 (17-24)
1698	North Korea	Female	55 to 59	59 (44-79)	35 (29-41)	18 (14-23)	92 (83-105)	35 (29-41)
1699	North Korea	Female	60 to 64	52 (35-71)	47 (39-56)	24 (20-29)	144 (130-160)	58 (49-68)
1700	North Korea	Female	65 to 69	35 (26-46)	59 (50-68)	30 (23-39)	227 (206-251)	97 (81-113)
1701	North Korea	Female	70 to <75	22 (14-33)	71 (60-82)	47 (40-56)	365 (334-400)	163 (135-191)
1702	North Korea	Male	40 to 44	37 (28-49)	11 (10-14)	6 (5-7)	46 (39-54)	13 (10-17)
1703	North Korea	Male	45 to 49	39 (28-52)	22 (19-26)	11 (8-14)	66 (58-76)	21 (16-26)
1704	North Korea	Male	50 to 54	37 (25-50)	36 (30-42)	17 (14-20)	99 (89-110)	34 (28-40)
1705	North Korea	Male	55 to 59	32 (22-42)	56 (48-65)	23 (18-30)	149 (138-161)	55 (47-63)
1706	North Korea	Male	60 to 64	28 (18-39)	85 (72-99)	31 (26-38)	229 (214-242)	89 (77-101)
1707	North Korea	Male	65 to 69	25 (18-34)	104 (90-120)	40 (31-51)	351 (329-370)	145 (126-164)
1708	North Korea	Male	70 to <75	20 (14-29)	128 (110-148)	59 (49-69)	543 (507-577)	234 (205-263)
1709	Norway	Female	40 to 44	29 (23-38)	27 (23-32)	3 (2-4)	7 (6-8)	1 (0-1)
1710	Norway	Female	45 to 49	30 (21-42)	44 (38-51)	5 (4-6)	13 (11-15)	1 (1-2)
1711	Norway	Female	50 to 54	38 (29-50)	64 (57-73)	6 (5-8)	22 (18-26)	2 (2-3)
1712	Norway	Female	55 to 59	53 (38-71)	83 (73-94)	7 (5-9)	33 (28-39)	4 (3-5)
1713	Norway	Female	60 to 64	60 (44-78)	111 (100-123)	8 (6-11)	53 (45-62)	7 (6-9)
1714	Norway	Female	65 to 69	59 (43-80)	146 (132-161)	11 (8-14)	86 (73-100)	15 (12-18)
1715	Norway	Female	70 to <75	52 (35-73)	166 (149-187)	18 (15-21)	136 (115-160)	30 (24-36)
1716	Norway	Male	40 to 44	41 (32-53)	17 (14-20)	3 (2-4)	12 (10-13)	2 (1-2)
1717	Norway	Male	45 to 49	47 (32-64)	27 (23-33)	5 (4-7)	18 (15-21)	4 (3-4)
1718	Norway	Male	50 to 54	58 (44-76)	50 (43-57)	7 (5-9)	31 (26-35)	7 (6-9)
1719	Norway	Male	55 to 59	76 (55-102)	92 (81-106)	8 (6-11)	50 (43-57)	12 (10-15)
1720	Norway	Male	60 to 64	82 (61-107)	161 (141-182)	11 (9-14)	82 (71-93)	21 (18-25)
1721	Norway	Male	65 to 69	77 (55-102)	250 (210-286)	16 (12-21)	136 (119-154)	37 (31-43)
1722	Norway	Male	70 to <75	64 (43-92)	323 (276-370)	26 (21-31)	223 (194-252)	65 (55-76)
1723	Oman	Female	40 to 44	112 (83-147)	9 (8-12)	2 (2-3)	13 (11-16)	3 (2-4)
1724	Oman	Female	45 to 49	150 (107-208)	13 (11-18)	3 (3-5)	20 (17-24)	6 (4-7)
1725	Oman	Female	50 to 54	165 (122-219)	17 (14-21)	5 (4-6)	33 (29-38)	11 (9-13)
1726	Oman	Female	55 to 59	157 (114-210)	21 (18-26)	7 (5-9)	53 (45-60)	20 (16-24)
1727	Oman	Female	60 to 64	132 (90-191)	26 (22-31)	11 (8-14)	88 (75-99)	37 (31-44)
1728	Oman	Female	65 to 69	90 (64-127)	30 (26-35)	17 (12-21)	147 (127-161)	68 (57-78)
1729	Oman	Female	70 to <75	63 (40-96)	36 (32-43)	23 (19-28)	237 (213-255)	113 (99-127)
1730	Oman	Male	40 to 44	120 (90-156)	3 (3-4)	2 (2-3)	20 (17-24)	6 (4-7)
1731	Oman	Male	45 to 49	189 (123-268)	6 (5-8)	4 (3-5)	33 (28-37)	12 (9-14)
1732	Oman	Male	50 to 54	217 (157-291)	11 (10-13)	6 (5-8)	52 (45-59)	21 (18-25)
1733	Oman	Male	55 to 59	205 (148-284)	18 (16-21)	10 (7-13)	83 (73-92)	37 (31-42)
1734	Oman	Male	60 to 64	174 (113-251)	30 (27-34)	14 (11-17)	140 (128-151)	63 (56-71)
1735	Oman	Male	65 to 69	125 (85-175)	46 (41-53)	19 (14-25)	222 (204-238)	102 (90-114)
1736	Oman	Male	70 to <75	87 (52-137)	62 (55-73)	29 (23-34)	365 (337-387)	175 (157-193)
1737	Pakistan	Female	40 to 44	116 (82-153)	18 (15-23)	2 (2-3)	33 (27-41)	8 (6-11)
1738	Pakistan	Female	45 to 49	138 (96-185)	26 (22-32)	4 (3-5)	48 (39-60)	14 (11-18)

ID	Country	Sex	Age	Diabetes type 2<br>Rate (95% CI)	Cancers<br>Rate (95% CI)	Hemorrhagic stroke<br>Rate (95% CI)	All-cause mortality<br>Rate (95% CI)	CVD mortality<br>Rate (95% CI)
1739	Pakistan	Female	50 to 54	134 (94-185)	37 (32-45)	6 (5-7)	73 (58-96)	22 (16-30)
1740	Pakistan	Female	55 to 59	104 (73-143)	43 (37-53)	9 (6-12)	113 (88-144)	43 (32-56)
1741	Pakistan	Female	60 to 64	80 (46-126)	51 (44-61)	13 (10-16)	179 (142-220)	77 (59-98)
1742	Pakistan	Female	65 to 69	61 (40-93)	54 (48-66)	19 (14-24)	287 (226-346)	135 (102-166)
1743	Pakistan	Female	70 to <75	57 (37-85)	49 (43-62)	26 (21-31)	454 (364-528)	222 (171-268)
1744	Pakistan	Male	40 to 44	94 (65-122)	12 (11-14)	3 (2-3)	48 (38-62)	17 (13-23)
1745	Pakistan	Male	45 to 49	88 (62-121)	18 (16-20)	4 (3-6)	67 (53-86)	28 (21-36)
1746	Pakistan	Male	50 to 54	82 (57-114)	27 (24-30)	7 (5-8)	98 (76-121)	45 (35-56)
1747	Pakistan	Male	55 to 59	74 (52-102)	39 (35-43)	10 (7-13)	142 (116-172)	69 (55-84)
1748	Pakistan	Male	60 to 64	62 (39-93)	59 (53-65)	15 (12-19)	219 (181-256)	109 (89-129)
1749	Pakistan	Male	65 to 69	46 (32-66)	78 (70-85)	22 (17-28)	331 (279-380)	171 (143-198)
1750	Pakistan	Male	70 to <75	40 (27-59)	74 (68-82)	28 (22-33)	505 (443-580)	270 (230-315)
1751	Panama	Female	40 to 44	43 (33-55)	18 (16-21)	2 (1-3)	13 (10-16)	2 (2-3)
1752	Panama	Female	45 to 49	49 (35-68)	26 (23-30)	3 (2-5)	19 (15-23)	3 (2-4)
1753	Panama	Female	50 to 54	54 (40-74)	34 (30-39)	5 (4-6)	28 (23-34)	5 (4-7)
1754	Panama	Female	55 to 59	60 (43-79)	41 (37-46)	6 (5-8)	40 (33-48)	8 (7-11)
1755	Panama	Female	60 to 64	60 (43-79)	52 (47-57)	9 (7-11)	63 (53-75)	15 (12-19)
1756	Panama	Female	65 to 69	54 (38-76)	63 (58-71)	12 (9-15)	97 (81-116)	26 (21-32)
1757	Panama	Female	70 to <75	49 (33-71)	78 (72-85)	16 (13-20)	157 (130-188)	46 (37-56)
1758	Panama	Male	40 to 44	58 (43-72)	8 (6-10)	2 (2-3)	26 (21-32)	4 (3-5)
1759	Panama	Male	45 to 49	74 (53-99)	12 (11-14)	4 (3-5)	34 (27-42)	7 (5-9)
1760	Panama	Male	50 to 54	79 (59-106)	22 (20-24)	6 (5-7)	47 (38-58)	12 (9-15)
1761	Panama	Male	55 to 59	72 (55-95)	39 (34-44)	8 (6-10)	70 (57-85)	20 (16-25)
1762	Panama	Male	60 to 64	65 (45-89)	76 (65-86)	11 (9-13)	113 (94-136)	34 (28-42)
1763	Panama	Male	65 to 69	58 (39-81)	115 (99-132)	15 (11-19)	167 (139-199)	53 (43-65)
1764	Panama	Male	70 to <75	54 (37-79)	170 (142-196)	20 (16-24)	260 (216-310)	86 (70-104)
1765	Papua New Guinea	Female	40 to 44	158 (120-199)	19 (12-25)	5 (4-6)	80 (63-102)	25 (19-34)
1766	Papua New Guinea	Female	45 to 49	228 (167-306)	22 (15-27)	9 (7-12)	110 (89-138)	38 (29-50)
1767	Papua New Guinea	Female	50 to 54	265 (204-340)	27 (19-33)	13 (10-16)	161 (132-196)	53 (41-70)
1768	Papua New Guinea	Female	55 to 59	267 (210-338)	32 (24-39)	17 (12-22)	231 (193-279)	81 (63-103)
1769	Papua New Guinea	Female	60 to 64	222 (160-301)	36 (30-43)	22 (18-28)	330 (280-390)	119 (94-150)
1770	Papua New Guinea	Female	65 to 69	129 (97-168)	48 (40-56)	30 (22-38)	492 (418-577)	188 (150-234)
1771	Papua New Guinea	Female	70 to <75	68 (43-102)	60 (51-70)	45 (37-54)	765 (655-897)	286 (229-362)
1772	Papua New Guinea	Male	40 to 44	110 (82-141)	7 (6-9)	5 (4-6)	97 (79-123)	34 (26-45)
1773	Papua New Guinea	Male	45 to 49	142 (104-187)	11 (9-13)	9 (6-12)	137 (108-174)	52 (38-67)
1774	Papua New Guinea	Male	50 to 54	151 (112-194)	15 (14-18)	13 (10-15)	194 (152-242)	78 (59-102)
1775	Papua New Guinea	Male	55 to 59	136 (101-177)	22 (20-27)	17 (13-22)	268 (212-324)	105 (80-134)
1776	Papua New Guinea	Male	60 to 64	112 (76-158)	32 (29-38)	22 (18-27)	383 (312-446)	148 (117-179)
1777	Papua New Guinea	Male	65 to 69	79 (58-106)	49 (44-57)	29 (22-37)	537 (455-614)	190 (152-228)
1778	Papua New Guinea	Male	70 to <75	54 (35-78)	62 (55-72)	40 (33-47)	786 (676-882)	269 (220-317)
1779	Paraguay	Female	40 to 44	53 (38-68)	19 (14-23)	3 (3-4)	18 (15-22)	4 (3-5)
1780	Paraguay	Female	45 to 49	77 (52-109)	26 (21-30)	6 (4-8)	29 (25-35)	7 (6-9)
1781	Paraguay	Female	50 to 54	95 (72-124)	33 (27-38)	8 (7-10)	45 (39-53)	12 (10-15)
1782	Paraguay	Female	55 to 59	108 (78-142)	41 (36-47)	11 (8-15)	70 (62-80)	20 (17-24)
1783	Paraguay	Female	60 to 64	104 (74-140)	51 (44-58)	15 (12-19)	112 (101-124)	35 (29-41)
1784	Paraguay	Female	65 to 69	86 (59-122)	62 (55-70)	21 (16-28)	174 (151-194)	59 (49-69)
1785	Paraguay	Female	70 to <75	72 (45-107)	71 (64-80)	29 (24-35)	272 (233-311)	100 (82-118)
1786	Paraguay	Male	40 to 44	56 (41-71)	7 (6-8)	3 (3-4)	34 (30-39)	7 (5-8)
1787	Paraguay	Male	45 to 49	76 (52-105)	12 (11-13)	6 (4-8)	50 (43-57)	13 (10-15)
1788	Paraguay	Male	50 to 54	84 (60-111)	20 (18-23)	9 (7-11)	74 (63-85)	22 (18-27)
1789	Paraguay	Male	55 to 59	80 (57-109)	34 (30-38)	12 (9-16)	109 (96-128)	37 (31-44)
1790	Paraguay	Male	60 to 64	75 (52-108)	53 (48-60)	17 (14-20)	165 (147-193)	58 (49-69)
1791	Paraguay	Male	65 to 69	70 (49-104)	79 (70-88)	22 (17-28)	250 (222-294)	92 (78-110)
1792	Paraguay	Male	70 to <75	66 (45-96)	111 (98-125)	30 (24-36)	386 (341-457)	146 (122-174)
1793	Peru	Female	40 to 44	25 (19-33)	14 (12-17)	2 (1-3)	12 (9-16)	1 (1-2)
1794	Peru	Female	45 to 49	30 (21-39)	20 (17-24)	3 (2-4)	18 (13-24)	2 (2-3)
1795	Peru	Female	50 to 54	33 (25-42)	27 (24-32)	5 (4-6)	28 (20-38)	4 (3-6)
1796	Peru	Female	55 to 59	35 (25-46)	36 (31-41)	6 (4-8)	41 (30-58)	6 (4-9)
1797	Peru	Female	60 to 64	34 (24-46)	46 (41-52)	8 (6-10)	67 (49-94)	11 (8-16)
1798	Peru	Female	65 to 69	30 (22-42)	59 (53-66)	11 (8-14)	104 (76-144)	19 (13-28)
1799	Peru	Female	70 to <75	27 (19-39)	72 (65-82)	15 (12-18)	169 (123-224)	35 (25-48)
1800	Peru	Male	40 to 44	32 (24-41)	5 (5-7)	2 (1-2)	19 (14-25)	3 (2-4)
1801	Peru	Male	45 to 49	40 (29-54)	9 (7-11)	3 (2-4)	26 (19-35)	4 (3-6)
1802	Peru	Male	50 to 54	44 (33-58)	15 (13-18)	4 (3-5)	39 (29-52)	7 (5-10)
1803	Peru	Male	55 to 59	46 (34-60)	24 (21-29)	6 (4-8)	58 (42-78)	11 (8-15)
1804	Peru	Male	60 to 64	44 (31-58)	41 (37-47)	8 (6-10)	94 (71-122)	19 (14-26)
1805	Peru	Male	65 to 69	38 (27-56)	66 (59-75)	10 (8-13)	147 (110-190)	31 (23-41)
1806	Peru	Male	70 to <75	36 (24-54)	98 (86-112)	13 (11-16)	231 (174-299)	50 (36-68)
1807	Philippines	Female	40 to 44	53 (39-67)	19 (16-21)	3 (3-4)	26 (20-32)	7 (5-9)
1808	Philippines	Female	45 to 49	68 (46-93)	25 (22-29)	6 (5-8)	38 (30-46)	12 (9-15)

				Diabetes type 2	Cancers	Hemorrhagic stroke	All-cause mortality	CVD mortality
ID	Country	Sex	Age	Rate (95% CI)	Rate (95% CI)	Rate (95% CI)	Rate (95% CI)	Rate (95% CI)
1809	Philippines	Female	50 to 54	81 (61-108)	30 (27-34)	9 (7-11)	56 (45-67)	19 (14-23)
1810	Philippines	Female	55 to 59	93 (71-120)	37 (33-41)	12 (9-16)	85 (69-102)	30 (24-37)
1811	Philippines	Female	60 to 64	88 (64-117)	41 (38-45)	16 (13-20)	128 (105-154)	49 (39-59)
1812	Philippines	Female	65 to 69	64 (48-87)	48 (44-52)	21 (16-26)	201 (164-241)	82 (65-99)
1813	Philippines	Female	70 to <75	46 (30-70)	54 (50-58)	30 (24-36)	321 (261-387)	136 (109-166)
1814	Philippines	Male	40 to 44	57 (43-78)	7 (7-8)	4 (3-5)	51 (41-63)	16 (12-21)
1815	Philippines	Male	45 to 49	59 (42-79)	12 (11-13)	7 (5-9)	75 (60-93)	27 (21-34)
1816	Philippines	Male	50 to 54	62 (45-85)	19 (18-21)	11 (9-14)	112 (91-137)	43 (34-54)
1817	Philippines	Male	55 to 59	67 (47-93)	32 (29-35)	17 (13-22)	176 (144-213)	70 (56-86)
1818	Philippines	Male	60 to 64	64 (46-88)	49 (45-54)	23 (18-28)	264 (220-314)	105 (86-127)
1819	Philippines	Male	65 to 69	55 (41-79)	70 (64-76)	29 (22-36)	399 (336-471)	160 (132-190)
1820	Philippines	Male	70 to <75	46 (30-71)	89 (81-101)	38 (31-45)	578 (488-679)	230 (189-273)
1821	Poland	Female	40 to 44	33 (26-40)	20 (17-23)	2 (2-2)	11 (9-12)	2 (1-2)
1822	Poland	Female	45 to 49	32 (22-45)	33 (29-38)	3 (3-4)	19 (16-22)	3 (3-4)
1823	Poland	Female	50 to 54	34 (25-48)	50 (45-57)	4 (4-5)	32 (28-37)	6 (5-8)
1824	Poland	Female	55 to 59	41 (30-53)	69 (62-77)	6 (5-6)	51 (45-58)	11 (9-13)
1825	Poland	Female	60 to 64	42 (30-55)	90 (81-100)	7 (7-8)	79 (70-89)	21 (18-25)
1826	Poland	Female	65 to 69	37 (27-52)	103 (93-114)	9 (8-10)	116 (102-132)	38 (32-44)
1827	Poland	Female	70 to <75	30 (20-44)	103 (93-116)	14 (13-15)	179 (157-204)	73 (63-84)
1828	Poland	Male	40 to 44	61 (48-76)	12 (10-13)	4 (3-4)	31 (27-36)	6 (5-8)
1829	Poland	Male	45 to 49	52 (37-69)	22 (19-25)	5 (5-6)	50 (44-57)	13 (11-16)
1830	Poland	Male	50 to 54	46 (30-66)	43 (38-48)	7 (6-8)	82 (72-93)	25 (21-30)
1831	Poland	Male	55 to 59	44 (31-59)	76 (68-86)	10 (9-11)	127 (112-142)	42 (36-49)
1832	Poland	Male	60 to 64	39 (26-55)	124 (111-140)	13 (12-14)	187 (167-208)	67 (58-76)
1833	Poland	Male	65 to 69	29 (21-42)	175 (154-199)	16 (14-18)	266 (238-295)	100 (88-113)
1834	Poland	Male	70 to <75	22 (14-33)	211 (185-245)	22 (20-24)	377 (338-417)	155 (136-177)
1835	Portugal	Female	40 to 44	27 (21-35)	22 (19-26)	2 (1-3)	9 (8-10)	1 (1-1)
1836	Portugal	Female	45 to 49	30 (20-41)	32 (27-37)	3 (2-4)	15 (13-16)	2 (2-2)
1837	Portugal	Female	50 to 54	43 (32-55)	38 (34-44)	4 (3-5)	21 (19-23)	3 (2-4)
1838	Portugal	Female	55 to 59	67 (48-92)	48 (42-54)	5 (4-7)	30 (27-33)	5 (4-6)
1839	Portugal	Female	60 to 64	77 (57-99)	61 (54-70)	7 (5-9)	44 (40-48)	9 (7-10)
1840	Portugal	Female	65 to 69	72 (52-96)	74 (66-83)	10 (8-13)	71 (64-78)	17 (15-20)
1841	Portugal	Female	70 to <75	59 (38-83)	88 (78-100)	17 (14-20)	128 (115-140)	38 (33-44)
1842	Portugal	Male	40 to 44	34 (26-43)	16 (14-18)	3 (2-3)	24 (21-26)	4 (3-4)
1843	Portugal	Male	45 to 49	43 (29-61)	29 (26-32)	5 (3-6)	36 (32-40)	6 (5-8)
1844	Portugal	Male	50 to 54	60 (45-77)	48 (44-54)	7 (5-8)	54 (48-59)	10 (8-12)
1845	Portugal	Male	55 to 59	84 (59-116)	74 (67-84)	9 (7-11)	74 (67-81)	15 (13-18)
1846	Portugal	Male	60 to 64	91 (67-124)	109 (98-125)	12 (10-15)	106 (96-115)	24 (21-28)
1847	Portugal	Male	65 to 69	82 (61-112)	158 (140-184)	18 (14-22)	162 (148-176)	40 (35-46)
1848	Portugal	Male	70 to <75	65 (42-100)	207 (182-242)	26 (22-31)	261 (239-283)	75 (66-84)
1849	Qatar	Female	40 to 44	135 (95-182)	16 (13-19)	2 (2-3)	10 (6-16)	2 (1-3)
1850	Qatar	Female	45 to 49	202 (123-308)	22 (19-26)	3 (2-5)	16 (10-25)	3 (2-6)
1851	Qatar	Female	50 to 54	266 (204-352)	27 (23-32)	4 (3-6)	25 (15-39)	5 (3-9)
1852	Qatar	Female	55 to 59	328 (231-443)	33 (29-39)	5 (4-8)	38 (24-61)	10 (6-17)
1853	Qatar	Female	60 to 64	294 (178-419)	37 (31-46)	8 (6-10)	61 (40-97)	18 (11-30)
1854	Qatar	Female	65 to 69	165 (115-228)	45 (38-53)	11 (8-15)	101 (65-153)	33 (20-50)
1855	Qatar	Female	70 to <75	88 (52-148)	52 (45-60)	15 (12-19)	175 (110-248)	61 (38-88)
1856	Qatar	Male	40 to 44	115 (81-155)	3 (3-4)	2 (2-3)	14 (9-21)	3 (2-5)
1857	Qatar	Male	45 to 49	188 (111-301)	6 (5-7)	4 (3-5)	21 (14-32)	6 (4-10)
1858	Qatar	Male	50 to 54	251 (187-341)	11 (10-12)	5 (4-6)	35 (22-51)	12 (8-18)
1859	Qatar	Male	55 to 59	306 (217-425)	19 (17-22)	7 (5-9)	54 (34-81)	19 (12-28)
1860	Qatar	Male	60 to 64	283 (176-411)	34 (30-39)	9 (7-12)	92 (60-133)	32 (20-46)
1861	Qatar	Male	65 to 69	183 (129-253)	53 (45-61)	11 (8-15)	146 (94-217)	52 (33-77)
1862	Qatar	Male	70 to <75	112 (63-182)	79 (66-92)	14 (11-18)	237 (151-356)	78 (48-117)
1863	Romania	Female	40 to 44	27 (21-34)	25 (21-29)	3 (3-4)	14 (12-16)	3 (2-4)
1864	Romania	Female	45 to 49	25 (17-34)	36 (31-42)	5 (4-7)	24 (20-27)	6 (5-7)
1865	Romania	Female	50 to 54	25 (17-35)	50 (45-57)	8 (6-9)	41 (36-47)	11 (9-13)
1866	Romania	Female	55 to 59	27 (20-36)	60 (54-68)	10 (8-13)	59 (52-67)	19 (16-23)
1867	Romania	Female	60 to 64	27 (19-36)	72 (65-81)	14 (12-17)	90 (79-102)	36 (30-42)
1868	Romania	Female	65 to 69	23 (17-32)	83 (75-93)	20 (16-25)	144 (126-163)	71 (61-81)
1869	Romania	Female	70 to <75	19 (12-28)	93 (84-105)	31 (26-36)	248 (217-282)	146 (126-166)
1870	Romania	Male	40 to 44	42 (33-52)	15 (14-17)	5 (4-6)	35 (30-41)	9 (7-11)
1871	Romania	Male	45 to 49	36 (26-46)	30 (27-33)	8 (6-11)	60 (52-70)	18 (15-22)
1872	Romania	Male	50 to 54	32 (23-43)	58 (53-64)	12 (10-15)	107 (93-125)	36 (30-42)
1873	Romania	Male	55 to 59	31 (22-43)	91 (81-102)	17 (13-21)	154 (134-177)	56 (47-66)
1874	Romania	Male	60 to 64	28 (19-40)	128 (113-146)	24 (20-29)	216 (190-245)	88 (76-102)
1875	Romania	Male	65 to 69	22 (16-31)	171 (149-202)	32 (25-40)	307 (272-348)	142 (124-163)
1876	Romania	Male	70 to <75	17 (11-26)	209 (180-251)	44 (37-53)	471 (417-531)	253 (222-286)
1877	Russia	Female	40 to 44	43 (34-53)	26 (22-30)	3 (3-4)	29 (17-48)	7 (4-12)
1878	Russia	Female	45 to 49	39 (28-52)	36 (32-41)	6 (4-8)	38 (23-61)	11 (7-18)

				Diabetes type 2	Cancers	Hemorrhagic stroke	All-cause mortality	CVD mortality
ID	Country	Sex	Age	Rate (95% CI)	Rate (95% CI)	Rate (95% CI)	Rate (95% CI)	Rate (95% CI)
1879	Russia	Female	50 to 54	38 (28-51)	49 (44-56)	8 (6-10)	53 (33-84)	18 (11-29)
1880	Russia	Female	55 to 59	39 (28-53)	66 (59-75)	11 (8-15)	80 (51-125)	33 (20-50)
1881	Russia	Female	60 to 64	35 (24-51)	82 (74-91)	15 (12-19)	117 (75-179)	57 (36-87)
1882	Russia	Female	65 to 69	27 (20-40)	94 (86-104)	21 (15-27)	174 (111-269)	99 (63-152)
1883	Russia	Female	70 to <75	21 (14-33)	101 (93-110)	28 (22-34)	284 (178-441)	185 (114-286)
1884	Russia	Male	40 to 44	48 (38-60)	13 (11-15)	6 (5-7)	85 (53-122)	24 (14-35)
1885	Russia	Male	45 to 49	42 (30-56)	24 (22-27)	10 (7-13)	112 (70-163)	39 (24-58)
1886	Russia	Male	50 to 54	38 (26-52)	46 (42-50)	14 (11-17)	156 (99-223)	64 (40-94)
1887	Russia	Male	55 to 59	36 (27-48)	81 (75-89)	18 (13-25)	221 (145-309)	99 (66-140)
1888	Russia	Male	60 to 64	32 (22-44)	125 (115-137)	24 (18-30)	318 (217-429)	160 (110-217)
1889	Russia	Male	65 to 69	26 (18-38)	167 (153-184)	30 (23-38)	413 (285-552)	226 (157-302)
1890	Russia	Male	70 to <75	21 (13-31)	197 (178-219)	36 (29-44)	583 (406-772)	353 (249-472)
1891	Rwanda	Female	40 to 44	26 (19-33)	14 (10-19)	1 (1-2)	40 (35-48)	3 (2-5)
1892	Rwanda	Female	45 to 49	31 (21-43)	19 (14-27)	3 (2-3)	56 (48-68)	7 (5-10)
1893	Rwanda	Female	50 to 54	38 (29-50)	26 (19-36)	4 (3-5)	81 (67-100)	12 (8-18)
1894	Rwanda	Female	55 to 59	48 (33-67)	29 (22-39)	5 (4-7)	113 (90-139)	23 (16-33)
1895	Rwanda	Female	60 to 64	56 (40-76)	39 (30-50)	9 (7-11)	174 (138-208)	42 (30-57)
1896	Rwanda	Female	65 to 69	62 (44-85)	44 (35-56)	13 (9-17)	269 (212-324)	77 (53-103)
1897	Rwanda	Female	70 to <75	60 (43-82)	46 (37-56)	20 (16-24)	427 (342-499)	138 (100-180)
1898	Rwanda	Male	40 to 44	31 (23-41)	6 (5-8)	2 (1-2)	55 (47-68)	5 (3-8)
1899	Rwanda	Male	45 to 49	49 (34-67)	9 (8-11)	3 (2-4)	76 (62-93)	9 (6-13)
1900	Rwanda	Male	50 to 54	60 (47-78)	15 (12-17)	4 (3-5)	106 (86-128)	15 (11-21)
1901	Rwanda	Male	55 to 59	65 (45-90)	23 (18-28)	6 (4-8)	144 (120-170)	25 (18-33)
1902	Rwanda	Male	60 to 64	67 (46-94)	38 (28-47)	9 (7-11)	214 (182-249)	46 (35-58)
1903	Rwanda	Male	65 to 69	67 (46-92)	55 (39-70)	13 (10-17)	317 (271-368)	78 (58-97)
1904	Rwanda	Male	70 to <75	62 (42-88)	80 (55-103)	19 (15-23)	485 (420-569)	125 (93-157)
1905	Saint Lucia	Female	40 to 44	64 (46-87)	22 (19-25)	2 (2-3)	22 (19-24)	4 (3-4)
1906	Saint Lucia	Female	45 to 49	75 (51-102)	28 (25-32)	4 (3-5)	29 (26-32)	6 (5-8)
1907	Saint Lucia	Female	50 to 54	91 (68-119)	32 (28-36)	6 (5-7)	42 (38-45)	10 (8-12)
1908	Saint Lucia	Female	55 to 59	111 (77-148)	39 (35-44)	8 (6-11)	61 (56-66)	17 (14-20)
1909	Saint Lucia	Female	60 to 64	107 (75-145)	46 (41-51)	12 (10-14)	91 (84-98)	30 (26-34)
1910	Saint Lucia	Female	65 to 69	78 (56-105)	60 (55-65)	17 (13-21)	150 (138-162)	53 (46-60)
1911	Saint Lucia	Female	70 to <75	57 (36-86)	63 (58-69)	23 (19-28)	209 (191-227)	82 (73-92)
1912	Saint Lucia	Male	40 to 44	77 (58-99)	10 (9-12)	2 (2-3)	36 (33-40)	6 (5-8)
1913	Saint Lucia	Male	45 to 49	109 (75-144)	18 (16-21)	4 (3-6)	54 (50-60)	12 (10-15)
1914	Saint Lucia	Male	50 to 54	116 (85-147)	29 (26-32)	7 (5-8)	72 (66-79)	20 (17-23)
1915	Saint Lucia	Male	55 to 59	98 (72-132)	46 (42-52)	10 (7-13)	107 (99-117)	34 (29-38)
1916	Saint Lucia	Male	60 to 64	79 (50-115)	76 (68-87)	14 (12-17)	162 (150-175)	55 (48-62)
1917	Saint Lucia	Male	65 to 69	59 (42-85)	110 (95-127)	20 (15-25)	227 (211-246)	81 (72-91)
1918	Saint Lucia	Male	70 to <75	47 (30-75)	156 (134-183)	26 (21-30)	345 (321-373)	122 (109-136)
1919	Saint Vincent and the Grenadines	Female	40 to 44	91 (65-126)	23 (20-26)	2 (2-3)	27 (23-31)	5 (4-6)
1920	Saint Vincent and the Grenadines	Female	45 to 49	126 (75-191)	35 (31-40)	4 (3-6)	41 (36-46)	9 (8-12)
1921	Saint Vincent and the Grenadines	Female	50 to 54	167 (124-217)	44 (39-49)	7 (5-8)	61 (55-67)	16 (13-19)
1922	Saint Vincent and the Grenadines	Female	55 to 59	214 (154-281)	55 (50-61)	9 (7-12)	90 (81-100)	26 (21-30)
1923	Saint Vincent and the Grenadines	Female	60 to 64	199 (133-270)	64 (58-70)	13 (10-16)	136 (119-152)	46 (39-54)
1924	Saint Vincent and the Grenadines	Female	65 to 69	122 (85-168)	80 (73-87)	17 (13-22)	210 (182-235)	78 (65-91)
1925	Saint Vincent and the Grenadines	Female	70 to <75	73 (38-127)	93 (86-101)	26 (21-31)	338 (293-378)	147 (126-168)
1926	Saint Vincent and the Grenadines	Male	40 to 44	118 (88-149)	10 (9-11)	3 (2-3)	47 (41-54)	8 (6-10)
1927	Saint Vincent and the Grenadines	Male	45 to 49	199 (143-263)	17 (15-20)	5 (4-7)	68 (61-77)	16 (13-20)
1928	Saint Vincent and the Grenadines	Male	50 to 54	212 (163-267)	31 (28-35)	8 (6-9)	100 (91-114)	27 (22-32)
1929	Saint Vincent and the Grenadines	Male	55 to 59	155 (118-202)	52 (46-59)	11 (8-14)	148 (133-170)	48 (41-57)
1930	Saint Vincent and the Grenadines	Male	60 to 64	110 (63-168)	88 (77-100)	16 (13-20)	222 (202-254)	78 (68-91)
1931	Saint Vincent and the Grenadines	Male	65 to 69	76 (50-111)	135 (117-158)	22 (17-28)	332 (301-376)	124 (108-144)
1932	Saint Vincent and the Grenadines	Male	70 to <75	56 (34-87)	187 (159-222)	30 (25-35)	502 (458-575)	204 (180-236)
1933	Samoa	Female	40 to 44	164 (127-208)	16 (12-19)	4 (3-5)	34 (26-41)	9 (7-12)
1934	Samoa	Female	45 to 49	248 (176-338)	19 (16-23)	6 (5-8)	49 (39-60)	15 (11-19)
1935	Samoa	Female	50 to 54	297 (231-379)	23 (20-27)	9 (8-11)	72 (57-88)	21 (15-27)
1936	Samoa	Female	55 to 59	311 (241-399)	27 (23-31)	13 (9-17)	105 (84-128)	35 (26-45)
1937	Samoa	Female	60 to 64	259 (185-359)	31 (27-35)	17 (14-21)	153 (124-185)	56 (42-71)
1938	Samoa	Female	65 to 69	144 (109-193)	38 (34-43)	22 (17-29)	229 (189-274)	89 (69-112)
1939	Samoa	Female	70 to <75	71 (44-108)	46 (40-52)	33 (27-40)	367 (300-438)	149 (116-186)
1940	Samoa	Male	40 to 44	178 (130-232)	6 (5-6)	4 (3-5)	46 (35-60)	16 (11-22)
1941	Samoa	Male	45 to 49	265 (186-362)	9 (8-10)	7 (5-9)	68 (51-89)	27 (19-36)
1942	Samoa	Male	50 to 54	289 (217-374)	14 (12-15)	11 (8-13)	103 (80-130)	45 (33-59)
1943	Samoa	Male	55 to 59	247 (187-317)	20 (18-22)	15 (11-20)	153 (121-184)	69 (52-85)
1944	Samoa	Male	60 to 64	190 (125-268)	29 (26-32)	21 (16-25)	226 (179-265)	102 (80-125)
1945	Samoa	Male	65 to 69	117 (85-158)	44 (40-49)	27 (21-35)	332 (269-376)	142 (112-168)
1946	Samoa	Male	70 to <75	67 (40-103)	53 (46-59)	37 (30-44)	493 (404-552)	210 (169-246)
1947	Sao Tome and Principe	Female	40 to 44	22 (17-29)	13 (10-16)	3 (2-3)	31 (25-39)	6 (4-8)
1948	Sao Tome and Principe	Female	45 to 49	29 (22-39)	18 (14-22)	4 (3-6)	47 (39-58)	11 (8-15)

				Diabetes type 2	Cancers	Hemorrhagic stroke	All-cause mortality	CVD mortality
**ID**	**Country**	**Sex**	**Age**	**Rate (95% CI)**	**Rate (95% CI)**	**Rate (95% CI)**	**Rate (95% CI)**	**Rate (95% CI)**
1949	Sao Tome and Principe	Female	50 to 54	33 (25-44)	21 (17-26)	6 (5-8)	72 (59-88)	19 (14-26)
1950	Sao Tome and Principe	Female	55 to 59	33 (24-45)	27 (21-32)	8 (6-11)	109 (88-129)	32 (24-41)
1951	Sao Tome and Principe	Female	60 to 64	34 (24-47)	36 (29-42)	12 (9-15)	165 (138-190)	53 (41-67)
1952	Sao Tome and Principe	Female	65 to 69	36 (26-49)	39 (33-45)	17 (12-21)	258 (216-291)	91 (71-112)
1953	Sao Tome and Principe	Female	70 to <75	36 (26-49)	47 (40-54)	24 (20-29)	408 (349-456)	148 (120-180)
1954	Sao Tome and Principe	Male	40 to 44	23 (18-31)	4 (4-5)	3 (2-3)	41 (34-51)	6 (3-8)
1955	Sao Tome and Principe	Male	45 to 49	31 (22-42)	7 (6-8)	4 (3-6)	60 (48-72)	10 (6-14)
1956	Sao Tome and Principe	Male	50 to 54	34 (26-46)	13 (11-15)	7 (5-8)	87 (69-106)	17 (11-23)
1957	Sao Tome and Principe	Male	55 to 59	35 (24-48)	19 (17-23)	9 (6-12)	130 (103-156)	27 (19-36)
1958	Sao Tome and Principe	Male	60 to 64	36 (25-49)	36 (32-41)	12 (10-15)	199 (157-238)	44 (32-57)
1959	Sao Tome and Principe	Male	65 to 69	37 (26-53)	58 (52-67)	17 (12-22)	300 (238-364)	68 (50-88)
1960	Sao Tome and Principe	Male	70 to <75	35 (24-51)	87 (78-98)	24 (19-29)	461 (371-568)	113 (83-149)
1961	Saudi Arabia	Female	40 to 44	53 (40-70)	12 (9-14)	2 (1-3)	9 (8-10)	2 (2-3)
1962	Saudi Arabia	Female	45 to 49	71 (48-96)	16 (13-20)	3 (2-4)	13 (11-14)	4 (3-4)
1963	Saudi Arabia	Female	50 to 54	83 (63-108)	20 (17-23)	4 (3-5)	22 (20-25)	7 (6-8)
1964	Saudi Arabia	Female	55 to 59	92 (68-123)	22 (18-27)	5 (4-8)	31 (27-35)	11 (10-13)
1965	Saudi Arabia	Female	60 to 64	84 (58-117)	37 (32-41)	9 (7-11)	105 (93-117)	46 (40-52)
1966	Saudi Arabia	Female	65 to 69	61 (44-85)	45 (39-50)	15 (11-19)	180 (160-198)	83 (72-94)
1967	Saudi Arabia	Female	70 to <75	47 (31-69)	64 (57-71)	21 (17-25)	322 (286-357)	148 (128-167)
1968	Saudi Arabia	Male	40 to 44	57 (42-76)	4 (4-5)	2 (1-2)	16 (14-18)	4 (3-5)
1969	Saudi Arabia	Male	45 to 49	77 (52-109)	7 (6-8)	3 (2-4)	24 (21-28)	8 (7-9)
1970	Saudi Arabia	Male	50 to 54	96 (72-126)	12 (11-13)	5 (4-6)	39 (34-44)	15 (13-18)
1971	Saudi Arabia	Male	55 to 59	113 (82-151)	18 (16-19)	7 (5-9)	56 (50-64)	24 (21-28)
1972	Saudi Arabia	Male	60 to 64	107 (75-149)	33 (30-37)	10 (8-13)	153 (138-170)	71 (63-80)
1973	Saudi Arabia	Male	65 to 69	78 (57-111)	50 (47-54)	15 (11-19)	231 (209-254)	109 (98-122)
1974	Saudi Arabia	Male	70 to <75	58 (37-89)	79 (74-85)	20 (16-24)	352 (319-386)	152 (136-171)
1975	Senegal	Female	40 to 44	37 (27-47)	13 (10-16)	3 (2-3)	44 (39-51)	6 (5-8)
1976	Senegal	Female	45 to 49	53 (37-74)	17 (13-21)	5 (3-6)	64 (56-73)	13 (10-17)
1977	Senegal	Female	50 to 54	61 (46-81)	22 (17-26)	7 (5-8)	97 (86-110)	25 (19-32)
1978	Senegal	Female	55 to 59	60 (42-84)	27 (22-32)	9 (7-12)	143 (129-159)	42 (33-52)
1979	Senegal	Female	60 to 64	62 (43-88)	35 (29-41)	12 (10-15)	213 (194-235)	70 (57-84)
1980	Senegal	Female	65 to 69	67 (46-93)	38 (32-44)	17 (12-21)	330 (307-359)	121 (100-143)
1981	Senegal	Female	70 to <75	64 (45-89)	42 (37-48)	24 (19-28)	505 (474-548)	186 (158-220)
1982	Senegal	Male	40 to 44	31 (23-40)	6 (5-7)	3 (2-3)	64 (56-72)	9 (6-12)
1983	Senegal	Male	45 to 49	41 (29-57)	9 (8-11)	5 (3-6)	86 (75-96)	15 (11-20)
1984	Senegal	Male	50 to 54	46 (35-61)	14 (13-17)	7 (6-8)	117 (103-130)	25 (18-31)
1985	Senegal	Male	55 to 59	46 (32-65)	20 (18-23)	9 (7-12)	160 (144-177)	37 (29-45)
1986	Senegal	Male	60 to 64	47 (33-66)	31 (28-35)	13 (10-15)	236 (214-258)	63 (51-76)
1987	Senegal	Male	65 to 69	48 (34-68)	47 (41-53)	17 (13-22)	351 (318-382)	92 (75-110)
1988	Senegal	Male	70 to <75	45 (31-64)	67 (58-76)	23 (18-27)	539 (483-589)	157 (125-185)
1989	Serbia	Female	40 to 44	44 (34-55)	28 (24-33)	3 (3-3)	15 (13-16)	3 (2-3)
1990	Serbia	Female	45 to 49	45 (31-61)	41 (35-48)	5 (5-6)	24 (21-26)	5 (4-6)
1991	Serbia	Female	50 to 54	49 (36-65)	55 (49-63)	7 (7-8)	39 (36-43)	9 (7-11)
1992	Serbia	Female	55 to 59	58 (41-78)	69 (60-79)	9 (8-10)	60 (55-65)	17 (15-20)
1993	Serbia	Female	60 to 64	59 (43-82)	81 (72-92)	12 (12-13)	92 (86-99)	32 (28-37)
1994	Serbia	Female	65 to 69	55 (38-80)	95 (84-108)	18 (16-19)	150 (141-161)	65 (58-72)
1995	Serbia	Female	70 to <75	45 (29-72)	106 (93-122)	24 (22-26)	269 (254-287)	142 (131-155)
1996	Serbia	Male	40 to 44	70 (56-86)	16 (14-18)	5 (4-6)	28 (25-32)	8 (6-10)
1997	Serbia	Male	45 to 49	68 (47-91)	29 (26-34)	8 (7-9)	47 (43-52)	15 (12-17)
1998	Serbia	Male	50 to 54	67 (46-90)	53 (48-60)	11 (10-12)	78 (71-86)	26 (22-30)
1999	Serbia	Male	55 to 59	66 (48-87)	83 (74-93)	14 (12-16)	122 (111-133)	43 (37-49)
2000	Serbia	Male	60 to 64	60 (42-82)	121 (107-137)	17 (15-19)	179 (164-193)	68 (59-76)
2001	Serbia	Male	65 to 69	48 (34-68)	163 (142-189)	19 (17-22)	270 (248-289)	114 (102-127)
2002	Serbia	Male	70 to <75	37 (24-55)	207 (179-243)	26 (24-30)	445 (412-470)	213 (193-232)
2003	Seychelles	Female	40 to 44	53 (39-70)	23 (19-28)	2 (2-3)	19 (15-26)	4 (3-6)
2004	Seychelles	Female	45 to 49	67 (45-99)	34 (29-39)	4 (3-6)	30 (23-39)	7 (5-10)
2005	Seychelles	Female	50 to 54	80 (59-109)	43 (37-50)	6 (5-8)	47 (37-58)	13 (9-16)
2006	Seychelles	Female	55 to 59	90 (67-120)	53 (46-60)	9 (6-11)	73 (58-84)	21 (16-25)
2007	Seychelles	Female	60 to 64	84 (59-114)	63 (56-71)	11 (9-14)	113 (94-127)	36 (28-43)
2008	Seychelles	Female	65 to 69	62 (45-83)	71 (64-79)	14 (10-18)	175 (150-195)	63 (51-74)
2009	Seychelles	Female	70 to <75	45 (29-66)	78 (70-86)	20 (16-24)	278 (245-310)	109 (93-126)
2010	Seychelles	Male	40 to 44	54 (40-71)	13 (11-14)	3 (2-4)	44 (33-57)	10 (7-14)
2011	Seychelles	Male	45 to 49	53 (38-72)	22 (20-24)	5 (4-7)	64 (50-84)	17 (13-24)
2012	Seychelles	Male	50 to 54	55 (40-75)	39 (35-42)	8 (6-10)	97 (77-122)	29 (22-38)
2013	Seychelles	Male	55 to 59	60 (42-83)	62 (57-70)	11 (9-15)	145 (120-177)	47 (37-58)
2014	Seychelles	Male	60 to 64	58 (39-80)	99 (88-113)	15 (12-18)	214 (181-257)	70 (57-86)
2015	Seychelles	Male	65 to 69	49 (34-71)	152 (132-176)	18 (14-23)	316 (271-368)	101 (83-120)
2016	Seychelles	Male	70 to <75	41 (27-61)	208 (179-252)	24 (20-29)	473 (407-536)	150 (124-176)
2017	Sierra Leone	Female	40 to 44	31 (23-41)	15 (11-19)	3 (2-4)	62 (55-71)	8 (6-11)
2018	Sierra Leone	Female	45 to 49	43 (30-61)	20 (15-24)	5 (4-7)	79 (67-94)	14 (11-19)

				Diabetes type 2	Cancers	Hemorrhagic stroke	All-cause mortality	CVD mortality
ID	Country	Sex	Age	Rate (95% CI)	Rate (95% CI)	Rate (95% CI)	Rate (95% CI)	Rate (95% CI)
2019	Sierra Leone	Female	50 to 54	49 (37-66)	24 (19-29)	7 (6-8)	112 (91-136)	27 (20-36)
2020	Sierra Leone	Female	55 to 59	51 (35-73)	31 (24-37)	9 (7-12)	161 (131-195)	45 (33-59)
2021	Sierra Leone	Female	60 to 64	53 (36-74)	41 (32-48)	13 (10-16)	245 (199-290)	75 (58-95)
2022	Sierra Leone	Female	65 to 69	56 (39-80)	45 (37-52)	18 (13-24)	382 (313-447)	133 (102-166)
2023	Sierra Leone	Female	70 to <75	54 (38-76)	49 (40-56)	26 (21-31)	595 (501-680)	211 (168-255)
2024	Sierra Leone	Male	40 to 44	24 (18-32)	6 (5-8)	3 (2-4)	78 (68-90)	12 (8-16)
2025	Sierra Leone	Male	45 to 49	30 (22-41)	10 (8-12)	5 (4-7)	100 (87-116)	19 (14-26)
2026	Sierra Leone	Male	50 to 54	34 (26-45)	16 (13-18)	8 (6-9)	132 (114-151)	31 (23-40)
2027	Sierra Leone	Male	55 to 59	36 (25-50)	22 (19-25)	10 (8-13)	179 (154-205)	46 (35-58)
2028	Sierra Leone	Male	60 to 64	37 (26-53)	33 (30-37)	14 (11-17)	265 (233-295)	78 (60-97)
2029	Sierra Leone	Male	65 to 69	39 (27-54)	49 (43-55)	18 (13-23)	389 (344-427)	112 (91-136)
2030	Sierra Leone	Male	70 to <75	37 (27-51)	69 (59-77)	25 (20-29)	601 (549-647)	183 (149-217)
2031	Singapore	Female	40 to 44	29 (21-39)	22 (19-26)	2 (1-3)	7 (5-9)	1 (1-1)
2032	Singapore	Female	45 to 49	36 (25-52)	33 (29-39)	3 (2-5)	11 (8-14)	2 (1-3)
2033	Singapore	Female	50 to 54	44 (33-60)	46 (39-52)	4 (3-6)	19 (14-24)	4 (3-5)
2034	Singapore	Female	55 to 59	51 (37-69)	62 (54-71)	5 (3-7)	31 (23-40)	7 (5-9)
2035	Singapore	Female	60 to 64	51 (35-68)	71 (63-80)	7 (5-9)	46 (34-59)	12 (8-15)
2036	Singapore	Female	65 to 69	43 (31-59)	103 (92-115)	11 (8-14)	95 (69-122)	28 (20-37)
2037	Singapore	Female	70 to <75	36 (24-51)	111 (100-122)	15 (12-18)	138 (100-179)	43 (31-57)
2038	Singapore	Male	40 to 44	40 (29-51)	11 (9-13)	2 (2-3)	11 (8-15)	4 (3-5)
2039	Singapore	Male	45 to 49	46 (33-63)	16 (14-18)	4 (3-5)	17 (13-23)	6 (4-8)
2040	Singapore	Male	50 to 54	50 (37-67)	28 (25-32)	5 (4-6)	31 (23-41)	11 (8-15)
2041	Singapore	Male	55 to 59	51 (37-68)	52 (47-58)	7 (5-10)	56 (42-74)	20 (15-26)
2042	Singapore	Male	60 to 64	48 (32-66)	81 (72-90)	10 (8-13)	85 (65-109)	29 (22-38)
2043	Singapore	Male	65 to 69	39 (28-55)	152 (137-168)	14 (11-18)	172 (132-220)	57 (44-74)
2044	Singapore	Male	70 to <75	31 (20-46)	191 (173-213)	19 (15-23)	246 (189-315)	76 (59-99)
2045	Slovakia	Female	40 to 44	33 (25-41)	26 (22-31)	3 (3-4)	11 (9-13)	2 (1-2)
2046	Slovakia	Female	45 to 49	32 (22-46)	41 (35-49)	5 (4-6)	19 (16-23)	3 (3-4)
2047	Slovakia	Female	50 to 54	34 (25-47)	56 (49-65)	7 (6-8)	31 (26-37)	6 (5-8)
2048	Slovakia	Female	55 to 59	38 (28-52)	76 (66-88)	9 (7-10)	50 (42-59)	13 (10-16)
2049	Slovakia	Female	60 to 64	39 (27-53)	99 (86-113)	11 (9-12)	79 (67-92)	25 (20-30)
2050	Slovakia	Female	65 to 69	35 (25-48)	122 (104-140)	14 (12-16)	123 (104-143)	49 (41-58)
2051	Slovakia	Female	70 to <75	29 (19-42)	140 (118-163)	20 (17-22)	208 (175-245)	102 (84-121)
2052	Slovakia	Male	40 to 44	50 (39-61)	15 (13-17)	5 (4-6)	27 (23-32)	6 (5-8)
2053	Slovakia	Male	45 to 49	45 (33-61)	29 (25-33)	7 (6-10)	47 (40-56)	13 (10-16)
2054	Slovakia	Male	50 to 54	43 (31-58)	54 (48-61)	11 (9-13)	80 (68-95)	24 (19-31)
2055	Slovakia	Male	55 to 59	43 (31-58)	95 (83-108)	14 (11-19)	128 (109-149)	43 (35-52)
2056	Slovakia	Male	60 to 64	38 (26-53)	149 (130-171)	19 (15-23)	199 (172-228)	75 (63-89)
2057	Slovakia	Male	65 to 69	30 (21-41)	214 (182-250)	24 (19-31)	284 (247-326)	119 (100-139)
2058	Slovakia	Male	70 to <75	22 (15-33)	279 (234-331)	31 (25-37)	424 (369-485)	198 (170-230)
2059	Slovenia	Female	40 to 44	32 (24-41)	25 (21-29)	3 (2-3)	8 (6-9)	1 (1-1)
2060	Slovenia	Female	45 to 49	30 (22-40)	37 (32-43)	4 (3-5)	14 (11-17)	2 (1-2)
2061	Slovenia	Female	50 to 54	32 (24-42)	50 (44-58)	5 (4-6)	23 (18-27)	3 (2-4)
2062	Slovenia	Female	55 to 59	38 (27-51)	68 (61-76)	6 (5-7)	36 (30-43)	5 (4-6)
2063	Slovenia	Female	60 to 64	41 (30-55)	86 (77-95)	7 (6-9)	53 (43-62)	9 (7-11)
2064	Slovenia	Female	65 to 69	41 (29-56)	102 (91-115)	9 (7-11)	80 (66-95)	19 (15-24)
2065	Slovenia	Female	70 to <75	37 (25-53)	119 (107-134)	14 (12-16)	135 (110-160)	42 (33-52)
2066	Slovenia	Male	40 to 44	46 (36-59)	14 (12-16)	4 (4-4)	17 (14-20)	2 (2-3)
2067	Slovenia	Male	45 to 49	43 (31-58)	25 (22-29)	6 (5-6)	28 (23-34)	5 (4-7)
2068	Slovenia	Male	50 to 54	44 (33-59)	49 (43-55)	8 (7-8)	49 (40-59)	10 (8-13)
2069	Slovenia	Male	55 to 59	50 (36-67)	85 (74-98)	10 (9-11)	81 (67-97)	18 (14-22)
2070	Slovenia	Male	60 to 64	48 (32-66)	136 (118-155)	13 (12-14)	126 (105-149)	31 (26-39)
2071	Slovenia	Male	65 to 69	38 (27-52)	191 (162-220)	18 (15-20)	185 (155-217)	51 (42-62)
2072	Slovenia	Male	70 to <75	29 (19-44)	240 (205-271)	25 (23-27)	288 (242-338)	90 (74-109)
2073	Solomon Islands	Female	40 to 44	228 (169-291)	19 (11-26)	5 (4-6)	79 (61-103)	27 (20-37)
2074	Solomon Islands	Female	45 to 49	371 (242-515)	22 (15-30)	9 (7-12)	109 (86-139)	40 (31-53)
2075	Solomon Islands	Female	50 to 54	442 (332-561)	27 (19-36)	13 (11-16)	157 (126-196)	56 (42-73)
2076	Solomon Islands	Female	55 to 59	442 (347-555)	33 (24-42)	18 (13-23)	226 (182-278)	88 (69-111)
2077	Solomon Islands	Female	60 to 64	354 (239-491)	37 (30-45)	23 (19-29)	319 (262-389)	132 (106-165)
2078	Solomon Islands	Female	65 to 69	179 (133-241)	49 (39-59)	30 (22-39)	473 (390-575)	205 (164-254)
2079	Solomon Islands	Female	70 to <75	75 (46-119)	62 (52-73)	47 (39-57)	744 (614-899)	331 (267-412)
2080	Solomon Islands	Male	40 to 44	178 (138-232)	9 (7-11)	5 (4-6)	95 (73-123)	37 (27-49)
2081	Solomon Islands	Male	45 to 49	253 (181-339)	13 (10-16)	9 (7-12)	137 (104-177)	58 (43-76)
2082	Solomon Islands	Male	50 to 54	271 (206-350)	18 (14-22)	13 (11-16)	193 (151-248)	88 (68-114)
2083	Solomon Islands	Male	55 to 59	233 (178-304)	25 (21-31)	18 (14-24)	264 (215-331)	119 (95-150)
2084	Solomon Islands	Male	60 to 64	180 (117-256)	37 (31-44)	25 (20-30)	367 (309-449)	164 (135-203)
2085	Solomon Islands	Male	65 to 69	112 (80-150)	54 (47-64)	32 (24-40)	509 (440-602)	213 (176-257)
2086	Solomon Islands	Male	70 to <75	66 (42-100)	66 (57-75)	43 (35-52)	726 (641-834)	304 (259-361)
2087	Somalia	Female	40 to 44	33 (25-42)	22 (15-29)	3 (2-3)	90 (76-109)	12 (8-16)
2088	Somalia	Female	45 to 49	38 (25-52)	30 (21-40)	4 (3-6)	123 (105-147)	21 (14-29)

				Diabetes type 2	Cancers	Hemorrhagic stroke	All-cause mortality	CVD mortality
ID	Country	Sex	Age	Rate (95% CI)	Rate (95% CI)	Rate (95% CI)	Rate (95% CI)	Rate (95% CI)
2089	Somalia	Female	50 to 54	46 (34-60)	40 (29-50)	6 (5-7)	175 (152-205)	37 (27-49)
2090	Somalia	Female	55 to 59	57 (39-80)	43 (33-55)	8 (6-11)	248 (217-288)	63 (47-81)
2091	Somalia	Female	60 to 64	65 (47-87)	54 (43-69)	13 (11-16)	355 (316-405)	106 (81-133)
2092	Somalia	Female	65 to 69	70 (51-98)	59 (48-74)	21 (16-27)	535 (478-608)	180 (141-221)
2093	Somalia	Female	70 to <75	66 (47-93)	61 (50-74)	31 (26-37)	818 (726-929)	307 (243-372)
2094	Somalia	Male	40 to 44	32 (23-41)	7 (6-9)	2 (2-3)	95 (82-110)	11 (8-16)
2095	Somalia	Male	45 to 49	48 (33-64)	11 (9-12)	4 (3-5)	125 (107-148)	20 (14-28)
2096	Somalia	Male	50 to 54	57 (43-73)	16 (14-18)	6 (5-7)	165 (141-201)	31 (21-44)
2097	Somalia	Male	55 to 59	58 (41-81)	25 (21-29)	8 (6-11)	225 (195-273)	48 (35-65)
2098	Somalia	Male	60 to 64	59 (42-80)	40 (31-47)	12 (10-15)	323 (282-394)	83 (64-110)
2099	Somalia	Male	65 to 69	57 (41-79)	57 (42-69)	18 (14-24)	464 (408-558)	129 (99-169)
2100	Somalia	Male	70 to <75	53 (37-74)	83 (59-103)	26 (21-32)	700 (619-823)	206 (162-268)
2101	South Africa	Female	40 to 44	52 (38-66)	22 (17-27)	2 (1-2)	100 (91-110)	4 (3-6)
2102	South Africa	Female	45 to 49	74 (52-102)	27 (23-32)	3 (2-4)	101 (92-112)	7 (5-8)
2103	South Africa	Female	50 to 54	95 (74-121)	37 (33-43)	4 (3-5)	109 (101-117)	12 (10-13)
2104	South Africa	Female	55 to 59	113 (82-149)	49 (43-58)	6 (5-8)	122 (114-130)	20 (18-22)
2105	South Africa	Female	60 to 64	127 (97-164)	59 (52-68)	10 (8-13)	163 (153-173)	41 (37-44)
2106	South Africa	Female	65 to 69	132 (97-181)	73 (63-84)	16 (12-20)	217 (204-229)	61 (55-67)
2107	South Africa	Female	70 to <75	119 (84-170)	79 (67-97)	22 (18-26)	294 (276-311)	91 (82-100)
2108	South Africa	Male	40 to 44	70 (51-90)	10 (9-13)	2 (1-2)	153 (142-164)	7 (5-8)
2109	South Africa	Male	45 to 49	101 (69-134)	20 (17-27)	3 (2-4)	167 (155-179)	11 (9-13)
2110	South Africa	Male	50 to 54	123 (94-154)	40 (34-48)	5 (4-6)	189 (178-200)	22 (19-24)
2111	South Africa	Male	55 to 59	136 (100-179)	68 (56-82)	8 (6-11)	220 (208-233)	36 (32-40)
2112	South Africa	Male	60 to 64	138 (103-183)	97 (84-115)	12 (10-15)	292 (278-307)	70 (65-76)
2113	South Africa	Male	65 to 69	126 (92-173)	139 (118-164)	18 (14-22)	381 (363-400)	100 (92-108)
2114	South Africa	Male	70 to <75	106 (72-153)	166 (139-203)	23 (19-27)	482 (458-506)	129 (120-139)
2115	South Korea	Female	40 to 44	33 (24-43)	27 (21-32)	2 (1-2)	9 (6-14)	1 (0-1)
2116	South Korea	Female	45 to 49	44 (29-63)	35 (28-41)	3 (2-4)	13 (8-19)	1 (1-2)
2117	South Korea	Female	50 to 54	60 (46-79)	38 (32-45)	4 (3-5)	18 (12-27)	2 (1-3)
2118	South Korea	Female	55 to 59	84 (58-113)	44 (38-51)	5 (3-7)	24 (16-35)	3 (2-5)
2119	South Korea	Female	60 to 64	89 (63-117)	56 (48-65)	7 (5-9)	38 (26-55)	6 (4-9)
2120	South Korea	Female	65 to 69	74 (55-102)	77 (69-86)	11 (8-14)	71 (47-102)	14 (9-22)
2121	South Korea	Female	70 to <75	58 (36-91)	101 (91-111)	17 (14-20)	133 (86-195)	34 (21-50)
2122	South Korea	Male	40 to 44	65 (51-80)	16 (14-18)	2 (2-3)	22 (13-31)	3 (2-4)
2123	South Korea	Male	45 to 49	91 (65-121)	27 (23-31)	4 (3-5)	35 (22-52)	5 (3-8)
2124	South Korea	Male	50 to 54	101 (78-128)	49 (42-58)	6 (5-7)	54 (34-78)	8 (5-12)
2125	South Korea	Male	55 to 59	96 (73-127)	77 (67-88)	8 (6-10)	74 (48-105)	11 (7-16)
2126	South Korea	Male	60 to 64	82 (55-117)	115 (100-130)	11 (8-14)	107 (71-148)	17 (11-24)
2127	South Korea	Male	65 to 69	59 (42-82)	177 (156-198)	15 (11-19)	177 (119-243)	31 (21-45)
2128	South Korea	Male	70 to <75	41 (25-62)	256 (229-284)	21 (17-26)	307 (207-419)	64 (43-88)
2129	South Sudan	Female	40 to 44	29 (22-38)	16 (11-28)	2 (2-2)	78 (60-103)	5 (3-8)
2130	South Sudan	Female	45 to 49	35 (24-49)	22 (15-39)	3 (2-4)	95 (71-126)	10 (6-15)
2131	South Sudan	Female	50 to 54	43 (32-57)	30 (21-47)	5 (4-6)	128 (94-168)	20 (13-29)
2132	South Sudan	Female	55 to 59	53 (36-76)	34 (24-54)	7 (5-10)	173 (126-224)	35 (23-51)
2133	South Sudan	Female	60 to 64	60 (42-83)	44 (33-67)	11 (9-14)	255 (190-319)	61 (41-82)
2134	South Sudan	Female	65 to 69	64 (45-87)	49 (38-72)	17 (13-22)	389 (290-479)	106 (76-142)
2135	South Sudan	Female	70 to <75	60 (43-83)	51 (39-75)	25 (20-30)	597 (463-723)	191 (139-245)
2136	South Sudan	Male	40 to 44	33 (24-43)	7 (5-8)	2 (2-3)	93 (74-118)	8 (5-12)
2137	South Sudan	Male	45 to 49	51 (36-72)	10 (8-12)	4 (3-5)	115 (90-146)	14 (9-20)
2138	South Sudan	Male	50 to 54	60 (46-79)	15 (11-17)	6 (5-7)	147 (116-181)	22 (15-32)
2139	South Sudan	Male	55 to 59	60 (44-81)	23 (18-27)	8 (6-10)	188 (147-230)	35 (24-47)
2140	South Sudan	Male	60 to 64	60 (42-82)	36 (27-43)	11 (9-14)	271 (220-324)	60 (44-80)
2141	South Sudan	Male	65 to 69	59 (41-81)	52 (38-63)	17 (12-22)	392 (322-459)	94 (71-123)
2142	South Sudan	Male	70 to <75	54 (38-78)	75 (52-94)	24 (19-29)	597 (512-691)	157 (120-201)
2143	Spain	Female	40 to 44	25 (19-32)	21 (18-24)	2 (2-3)	7 (7-8)	1 (1-1)
2144	Spain	Female	45 to 49	25 (17-37)	32 (28-37)	4 (3-5)	12 (11-13)	2 (1-2)
2145	Spain	Female	50 to 54	34 (26-45)	43 (39-49)	5 (4-6)	19 (18-20)	3 (2-3)
2146	Spain	Female	55 to 59	50 (34-69)	54 (48-61)	5 (4-7)	27 (26-29)	4 (3-5)
2147	Spain	Female	60 to 64	58 (44-76)	67 (60-75)	7 (5-9)	39 (36-41)	7 (6-8)
2148	Spain	Female	65 to 69	58 (42-79)	80 (71-90)	9 (7-12)	58 (55-62)	12 (10-14)
2149	Spain	Female	70 to <75	48 (31-73)	98 (88-113)	14 (11-17)	101 (95-108)	26 (22-29)
2150	Spain	Male	40 to 44	30 (23-39)	12 (10-14)	3 (2-3)	13 (12-14)	3 (2-3)
2151	Spain	Male	45 to 49	37 (25-52)	24 (21-28)	4 (3-5)	23 (22-25)	5 (4-6)
2152	Spain	Male	50 to 54	50 (37-64)	45 (41-51)	6 (4-7)	39 (36-42)	9 (7-10)
2153	Spain	Male	55 to 59	70 (49-101)	78 (70-87)	7 (5-10)	61 (57-65)	14 (12-15)
2154	Spain	Male	60 to 64	74 (54-103)	120 (108-133)	10 (8-13)	93 (88-100)	21 (18-24)
2155	Spain	Male	65 to 69	64 (46-89)	175 (157-196)	14 (10-18)	140 (131-149)	33 (29-37)
2156	Spain	Male	70 to <75	48 (28-75)	232 (208-264)	21 (17-25)	219 (206-234)	54 (48-61)
2157	Sri Lanka	Female	40 to 44	59 (43-78)	8 (7-10)	2 (1-3)	11 (7-14)	2 (1-3)
2158	Sri Lanka	Female	45 to 49	81 (53-116)	13 (11-15)	3 (2-5)	17 (12-22)	4 (2-5)

				Diabetes type 2	Cancers	Hemorrhagic stroke	All-cause mortality	CVD mortality
ID	Country	Sex	Age	Rate (95% CI)	Rate (95% CI)	Rate (95% CI)	Rate (95% CI)	Rate (95% CI)
2159	Sri Lanka	Female	50 to 54	112 (86-142)	17 (15-20)	5 (4-6)	27 (19-35)	7 (4-9)
2160	Sri Lanka	Female	55 to 59	150 (109-194)	21 (18-24)	6 (4-8)	40 (29-52)	11 (7-14)
2161	Sri Lanka	Female	60 to 64	148 (109-196)	26 (22-30)	8 (7-11)	64 (47-82)	19 (14-26)
2162	Sri Lanka	Female	65 to 69	106 (79-142)	33 (29-36)	12 (9-15)	121 (88-156)	43 (31-57)
2163	Sri Lanka	Female	70 to <75	72 (44-113)	41 (37-45)	18 (14-22)	240 (174-311)	95 (66-127)
2164	Sri Lanka	Male	40 to 44	72 (52-96)	5 (5-5)	2 (2-3)	36 (26-48)	7 (5-10)
2165	Sri Lanka	Male	45 to 49	79 (52-121)	9 (8-10)	4 (3-5)	53 (38-71)	13 (9-19)
2166	Sri Lanka	Male	50 to 54	93 (63-130)	15 (14-17)	6 (5-7)	78 (57-105)	24 (16-34)
2167	Sri Lanka	Male	55 to 59	113 (76-160)	23 (21-25)	9 (6-11)	107 (80-142)	37 (27-51)
2168	Sri Lanka	Male	60 to 64	115 (80-157)	32 (30-35)	12 (10-15)	153 (117-198)	56 (42-74)
2169	Sri Lanka	Male	65 to 69	97 (68-136)	48 (44-52)	17 (13-22)	253 (195-325)	100 (76-134)
2170	Sri Lanka	Male	70 to <75	77 (44-118)	64 (58-71)	23 (19-28)	428 (331-546)	175 (133-224)
2171	Sudan	Female	40 to 44	57 (44-74)	11 (7-15)	3 (2-4)	32 (30-34)	10 (8-12)
2172	Sudan	Female	45 to 49	71 (51-97)	15 (10-21)	5 (3-6)	45 (43-48)	17 (14-20)
2173	Sudan	Female	50 to 54	79 (59-105)	19 (13-24)	6 (5-8)	65 (60-71)	29 (25-34)
2174	Sudan	Female	55 to 59	81 (60-106)	23 (17-29)	8 (6-11)	96 (86-107)	47 (40-54)
2175	Sudan	Female	60 to 64	75 (54-101)	28 (25-32)	11 (9-15)	149 (134-168)	77 (65-90)
2176	Sudan	Female	65 to 69	61 (44-85)	35 (30-40)	16 (12-21)	232 (207-262)	124 (105-145)
2177	Sudan	Female	70 to <75	50 (33-72)	45 (38-52)	22 (18-27)	375 (340-419)	203 (170-238)
2178	Sudan	Male	40 to 44	54 (42-71)	5 (4-6)	3 (2-3)	45 (42-49)	14 (10-17)
2179	Sudan	Male	45 to 49	67 (47-88)	8 (6-10)	4 (3-6)	61 (56-66)	24 (18-28)
2180	Sudan	Male	50 to 54	75 (56-98)	13 (10-15)	7 (5-8)	85 (78-92)	38 (31-44)
2181	Sudan	Male	55 to 59	79 (58-106)	19 (15-23)	9 (7-12)	122 (112-133)	58 (48-66)
2182	Sudan	Male	60 to 64	77 (55-108)	29 (25-35)	13 (10-16)	188 (173-204)	92 (78-106)
2183	Sudan	Male	65 to 69	70 (50-99)	41 (35-48)	17 (13-22)	285 (264-308)	144 (122-163)
2184	Sudan	Male	70 to <75	59 (39-87)	61 (52-70)	24 (19-29)	454 (421-488)	224 (192-253)
2185	Suriname	Female	40 to 44	71 (51-94)	18 (16-21)	3 (2-3)	24 (22-26)	4 (3-5)
2186	Suriname	Female	45 to 49	93 (65-128)	24 (21-28)	5 (4-7)	36 (33-39)	9 (7-11)
2187	Suriname	Female	50 to 54	105 (79-138)	31 (28-35)	8 (6-10)	55 (51-60)	17 (14-19)
2188	Suriname	Female	55 to 59	108 (81-142)	38 (34-43)	11 (8-14)	82 (75-90)	27 (22-31)
2189	Suriname	Female	60 to 64	95 (68-133)	48 (44-53)	16 (12-19)	125 (115-138)	46 (40-54)
2190	Suriname	Female	65 to 69	67 (49-93)	60 (55-66)	22 (17-28)	193 (176-214)	78 (67-91)
2191	Suriname	Female	70 to <75	48 (30-71)	74 (67-81)	31 (26-36)	304 (277-341)	129 (113-148)
2192	Suriname	Male	40 to 44	97 (74-122)	9 (8-10)	3 (3-4)	44 (41-47)	9 (7-11)
2193	Suriname	Male	45 to 49	152 (108-200)	15 (13-17)	6 (4-8)	63 (58-67)	17 (14-21)
2194	Suriname	Male	50 to 54	158 (118-201)	24 (22-27)	9 (7-11)	91 (83-98)	31 (26-35)
2195	Suriname	Male	55 to 59	116 (85-158)	39 (35-43)	13 (10-17)	134 (121-144)	50 (43-56)
2196	Suriname	Male	60 to 64	84 (53-129)	62 (55-69)	19 (16-23)	204 (181-220)	82 (71-93)
2197	Suriname	Male	65 to 69	61 (41-90)	91 (81-103)	27 (21-33)	304 (267-334)	128 (110-146)
2198	Suriname	Male	70 to <75	47 (28-74)	133 (116-152)	36 (29-42)	466 (410-515)	195 (168-222)
2199	Swaziland	Female	40 to 44	86 (63-113)	15 (12-19)	2 (1-2)	118 (82-161)	4 (2-9)
2200	Swaziland	Female	45 to 49	120 (81-174)	21 (17-25)	3 (2-4)	125 (74-183)	9 (3-19)
2201	Swaziland	Female	50 to 54	146 (108-195)	28 (23-33)	4 (3-6)	144 (81-214)	16 (6-32)
2202	Swaziland	Female	55 to 59	164 (109-237)	37 (31-45)	6 (4-9)	146 (67-241)	27 (10-50)
2203	Swaziland	Female	60 to 64	177 (120-251)	42 (36-49)	10 (8-13)	199 (94-318)	56 (24-97)
2204	Swaziland	Female	65 to 69	187 (135-249)	53 (46-60)	16 (11-22)	292 (139-464)	94 (43-155)
2205	Swaziland	Female	70 to <75	158 (100-225)	62 (54-71)	24 (19-29)	449 (223-709)	162 (77-263)
2206	Swaziland	Male	40 to 44	109 (79-144)	8 (7-10)	2 (2-3)	203 (160-257)	10 (5-15)
2207	Swaziland	Male	45 to 49	161 (100-238)	18 (15-21)	4 (3-5)	228 (163-307)	19 (10-30)
2208	Swaziland	Male	50 to 54	194 (138-266)	33 (29-38)	6 (5-7)	269 (183-370)	33 (19-50)
2209	Swaziland	Male	55 to 59	207 (132-298)	53 (46-60)	9 (6-12)	303 (193-415)	51 (29-75)
2210	Swaziland	Male	60 to 64	206 (139-296)	68 (59-78)	13 (11-17)	399 (260-519)	97 (61-132)
2211	Swaziland	Male	65 to 69	192 (139-256)	93 (82-107)	20 (15-26)	545 (368-679)	147 (97-195)
2212	Swaziland	Male	70 to <75	151 (89-228)	117 (99-141)	29 (24-34)	783 (548-936)	219 (149-277)
2213	Sweden	Female	40 to 44	36 (28-46)	25 (22-29)	3 (2-4)	7 (6-8)	1 (1-1)
2214	Sweden	Female	45 to 49	41 (27-59)	38 (34-43)	5 (3-6)	12 (10-14)	1 (1-2)
2215	Sweden	Female	50 to 54	52 (38-70)	54 (49-60)	6 (4-8)	21 (17-25)	3 (2-3)
2216	Sweden	Female	55 to 59	70 (47-95)	73 (66-81)	6 (4-9)	33 (28-39)	5 (4-6)
2217	Sweden	Female	60 to 64	76 (55-103)	101 (92-113)	7 (5-10)	53 (45-63)	9 (7-11)
2218	Sweden	Female	65 to 69	71 (49-102)	135 (124-148)	10 (7-13)	88 (74-104)	18 (15-22)
2219	Sweden	Female	70 to <75	59 (36-91)	161 (148-175)	15 (12-18)	142 (120-169)	36 (29-44)
2220	Sweden	Male	40 to 44	46 (35-59)	13 (11-15)	3 (2-4)	11 (9-13)	2 (1-2)
2221	Sweden	Male	45 to 49	59 (41-82)	21 (18-23)	5 (3-6)	18 (15-21)	4 (3-5)
2222	Sweden	Male	50 to 54	79 (61-101)	40 (36-44)	6 (5-8)	30 (26-36)	8 (7-10)
2223	Sweden	Male	55 to 59	105 (75-141)	74 (65-83)	7 (5-10)	49 (42-58)	14 (12-17)
2224	Sweden	Male	60 to 64	109 (80-145)	134 (117-149)	10 (8-13)	82 (71-96)	26 (22-31)
2225	Sweden	Male	65 to 69	91 (65-128)	211 (182-240)	15 (11-19)	135 (116-156)	44 (37-52)
2226	Sweden	Male	70 to <75	71 (43-109)	274 (238-306)	23 (18-27)	223 (191-257)	77 (65-90)
2227	Switzerland	Female	40 to 44	21 (16-27)	24 (21-28)	3 (2-3)	6 (4-9)	1 (0-1)
2228	Switzerland	Female	45 to 49	22 (15-31)	39 (34-45)	5 (4-6)	11 (7-16)	1 (1-2)

				Diabetes type 2	Cancers	Hemorrhagic stroke	All-cause mortality	CVD mortality
ID	Country	Sex	Age	Rate (95% CI)	Rate (95% CI)	Rate (95% CI)	Rate (95% CI)	Rate (95% CI)
2229	Switzerland	Female	50 to 54	30 (23-38)	54 (48-60)	6 (5-7)	18 (12-26)	2 (1-3)
2230	Switzerland	Female	55 to 59	43 (31-58)	73 (66-81)	7 (5-8)	29 (20-41)	3 (2-5)
2231	Switzerland	Female	60 to 64	50 (39-65)	97 (87-107)	8 (7-10)	44 (31-62)	6 (4-9)
2232	Switzerland	Female	65 to 69	50 (37-67)	127 (116-139)	12 (9-14)	69 (48-98)	11 (7-16)
2233	Switzerland	Female	70 to <75	44 (31-61)	147 (134-160)	19 (15-21)	112 (77-161)	25 (17-36)
2234	Switzerland	Male	40 to 44	29 (22-37)	16 (14-18)	3 (2-4)	11 (8-16)	2 (1-3)
2235	Switzerland	Male	45 to 49	35 (24-48)	26 (23-29)	5 (4-6)	17 (12-25)	3 (2-5)
2236	Switzerland	Male	50 to 54	46 (35-59)	50 (45-55)	6 (5-8)	29 (20-43)	6 (4-9)
2237	Switzerland	Male	55 to 59	62 (44-83)	89 (81-99)	8 (5-10)	49 (34-70)	10 (7-16)
2238	Switzerland	Male	60 to 64	69 (51-91)	153 (135-173)	10 (8-13)	78 (56-109)	18 (12-25)
2239	Switzerland	Male	65 to 69	66 (48-93)	234 (205-264)	15 (11-19)	125 (91-174)	31 (23-43)
2240	Switzerland	Male	70 to <75	55 (36-81)	295 (250-332)	24 (19-30)	195 (142-271)	55 (38-76)
2241	Syria	Female	40 to 44	46 (35-60)	7 (6-8)	2 (2-3)	25 (17-34)	6 (5-7)
2242	Syria	Female	45 to 49	54 (39-71)	10 (8-11)	3 (2-4)	34 (24-44)	11 (9-12)
2243	Syria	Female	50 to 54	60 (44-77)	12 (10-14)	5 (4-6)	47 (36-60)	18 (15-21)
2244	Syria	Female	55 to 59	64 (49-85)	15 (13-17)	6 (4-8)	67 (53-81)	29 (24-34)
2245	Syria	Female	60 to 64	61 (44-82)	20 (17-24)	9 (7-11)	103 (84-122)	50 (41-59)
2246	Syria	Female	65 to 69	49 (36-70)	24 (20-28)	12 (9-16)	159 (134-182)	83 (70-96)
2247	Syria	Female	70 to <75	41 (28-61)	30 (26-35)	17 (14-21)	256 (221-281)	143 (123-158)
2248	Syria	Male	40 to 44	47 (36-60)	4 (4-5)	3 (2-3)	62 (34-92)	12 (10-15)
2249	Syria	Male	45 to 49	56 (41-75)	7 (6-8)	4 (3-6)	67 (44-92)	22 (18-26)
2250	Syria	Male	50 to 54	62 (47-83)	12 (10-13)	6 (5-8)	86 (64-109)	36 (31-42)
2251	Syria	Male	55 to 59	65 (48-85)	18 (16-20)	9 (7-12)	123 (97-149)	60 (50-70)
2252	Syria	Male	60 to 64	64 (45-85)	26 (24-30)	12 (10-15)	184 (154-213)	101 (87-113)
2253	Syria	Male	65 to 69	58 (42-81)	38 (33-45)	16 (12-21)	276 (235-311)	160 (136-178)
2254	Syria	Male	70 to <75	50 (34-71)	53 (45-66)	22 (17-26)	428 (369-473)	253 (216-280)
2255	Tajikistan	Female	40 to 44	31 (25-38)	10 (8-12)	3 (3-4)	22 (17-27)	6 (4-7)
2256	Tajikistan	Female	45 to 49	35 (25-47)	14 (11-19)	6 (5-8)	32 (26-39)	11 (9-15)
2257	Tajikistan	Female	50 to 54	36 (26-48)	19 (16-25)	10 (8-12)	51 (40-60)	22 (17-28)
2258	Tajikistan	Female	55 to 59	34 (25-45)	25 (20-33)	14 (11-19)	79 (64-94)	39 (31-47)
2259	Tajikistan	Female	60 to 64	31 (21-43)	32 (26-41)	21 (17-25)	126 (106-150)	71 (59-86)
2260	Tajikistan	Female	65 to 69	26 (19-35)	39 (32-47)	28 (21-35)	199 (170-239)	124 (105-151)
2261	Tajikistan	Female	70 to <75	21 (14-30)	45 (37-57)	37 (30-44)	323 (278-389)	219 (187-264)
2262	Tajikistan	Male	40 to 44	37 (29-46)	6 (5-9)	5 (4-6)	37 (30-44)	10 (7-13)
2263	Tajikistan	Male	45 to 49	38 (27-51)	11 (9-16)	8 (6-11)	52 (42-63)	19 (15-24)
2264	Tajikistan	Male	50 to 54	37 (26-51)	19 (15-25)	13 (11-16)	78 (64-96)	34 (27-43)
2265	Tajikistan	Male	55 to 59	34 (25-45)	31 (24-40)	19 (14-24)	116 (96-144)	57 (47-71)
2266	Tajikistan	Male	60 to 64	31 (21-43)	49 (39-62)	25 (20-31)	185 (156-228)	101 (84-126)
2267	Tajikistan	Male	65 to 69	28 (20-39)	73 (55-94)	31 (24-40)	280 (238-345)	165 (139-207)
2268	Tajikistan	Male	70 to <75	23 (16-34)	95 (69-125)	42 (34-51)	448 (386-544)	289 (250-355)
2269	Tanzania	Female	40 to 44	25 (19-32)	14 (11-18)	2 (1-2)	56 (48-67)	5 (3-7)
2270	Tanzania	Female	45 to 49	30 (21-42)	19 (15-23)	3 (2-4)	71 (59-88)	9 (6-13)
2271	Tanzania	Female	50 to 54	37 (28-49)	26 (22-32)	5 (4-6)	99 (78-122)	18 (12-24)
2272	Tanzania	Female	55 to 59	46 (32-66)	29 (24-35)	7 (5-9)	135 (101-163)	32 (22-42)
2273	Tanzania	Female	60 to 64	53 (39-73)	39 (33-46)	10 (8-13)	203 (151-238)	54 (38-70)
2274	Tanzania	Female	65 to 69	59 (43-83)	44 (38-52)	16 (11-20)	315 (233-362)	100 (71-126)
2275	Tanzania	Female	70 to <75	57 (41-79)	46 (40-52)	22 (17-26)	490 (372-549)	172 (126-208)
2276	Tanzania	Male	40 to 44	30 (22-38)	7 (6-8)	2 (1-2)	90 (77-105)	8 (5-11)
2277	Tanzania	Male	45 to 49	45 (32-59)	10 (8-11)	3 (3-5)	109 (91-132)	14 (10-20)
2278	Tanzania	Male	50 to 54	54 (41-68)	15 (13-17)	5 (4-6)	134 (111-164)	24 (17-31)
2279	Tanzania	Male	55 to 59	55 (39-75)	23 (19-27)	7 (5-9)	168 (142-202)	38 (30-49)
2280	Tanzania	Male	60 to 64	56 (40-75)	39 (29-47)	10 (8-13)	241 (208-290)	68 (54-85)
2281	Tanzania	Male	65 to 69	57 (40-79)	57 (42-69)	15 (11-19)	353 (308-422)	108 (87-133)
2282	Tanzania	Male	70 to <75	54 (37-75)	82 (58-105)	21 (17-25)	537 (469-636)	170 (137-210)
2283	Thailand	Female	40 to 44	41 (30-52)	15 (13-18)	2 (2-3)	18 (16-20)	2 (1-3)
2284	Thailand	Female	45 to 49	50 (36-69)	21 (19-24)	4 (3-5)	25 (22-28)	4 (3-5)
2285	Thailand	Female	50 to 54	60 (46-78)	26 (23-30)	5 (4-7)	34 (30-39)	6 (4-7)
2286	Thailand	Female	55 to 59	71 (54-93)	32 (28-36)	7 (5-9)	48 (43-55)	8 (6-11)
2287	Thailand	Female	60 to 64	69 (52-93)	41 (37-46)	9 (7-11)	77 (69-88)	14 (12-18)
2288	Thailand	Female	65 to 69	53 (39-73)	50 (45-55)	11 (8-15)	121 (107-138)	26 (21-32)
2289	Thailand	Female	70 to <75	41 (27-60)	59 (53-65)	17 (14-21)	195 (173-223)	47 (38-58)
2290	Thailand	Male	40 to 44	38 (28-49)	10 (9-12)	3 (3-4)	46 (40-53)	7 (6-9)
2291	Thailand	Male	45 to 49	37 (27-50)	17 (15-19)	6 (4-7)	58 (50-67)	11 (8-14)
2292	Thailand	Male	50 to 54	39 (29-52)	27 (24-31)	8 (7-10)	75 (65-86)	15 (12-19)
2293	Thailand	Male	55 to 59	44 (32-57)	39 (34-44)	10 (8-13)	94 (81-108)	20 (16-26)
2294	Thailand	Male	60 to 64	44 (32-59)	56 (50-63)	13 (10-16)	132 (116-150)	29 (23-35)
2295	Thailand	Male	65 to 69	40 (29-55)	77 (67-86)	16 (12-21)	192 (169-217)	44 (36-53)
2296	Thailand	Male	70 to <75	35 (24-49)	100 (89-110)	21 (17-26)	296 (261-335)	69 (57-84)
2297	The Bahamas	Female	40 to 44	59 (42-77)	34 (29-40)	2 (2-3)	29 (25-34)	5 (4-6)
2298	The Bahamas	Female	45 to 49	74 (52-104)	41 (35-47)	4 (3-6)	36 (31-41)	8 (6-10)

				Diabetes type 2	Cancers	Hemorrhagic stroke	All-cause mortality	CVD mortality
ID	Country	Sex	Age	Rate (95% CI)	Rate (95% CI)	Rate (95% CI)	Rate (95% CI)	Rate (95% CI)
2299	The Bahamas	Female	50 to 54	85 (63-110)	56 (49-63)	6 (5-7)	55 (48-62)	15 (12-18)
2300	The Bahamas	Female	55 to 59	91 (65-123)	72 (64-82)	8 (6-10)	80 (69-91)	24 (20-30)
2301	The Bahamas	Female	60 to 64	85 (60-119)	80 (71-89)	11 (9-14)	105 (90-122)	36 (29-43)
2302	The Bahamas	Female	65 to 69	68 (48-95)	101 (91-113)	15 (12-20)	173 (151-201)	66 (55-79)
2303	The Bahamas	Female	70 to <75	53 (33-80)	126 (114-139)	22 (18-27)	291 (245-341)	122 (101-146)
2304	The Bahamas	Male	40 to 44	76 (57-101)	16 (13-19)	3 (2-3)	53 (46-61)	11 (9-14)
2305	The Bahamas	Male	45 to 49	105 (74-140)	25 (21-28)	5 (3-6)	67 (58-77)	19 (15-23)
2306	The Bahamas	Male	50 to 54	109 (81-146)	39 (35-44)	7 (5-8)	83 (72-96)	28 (23-33)
2307	The Bahamas	Male	55 to 59	89 (64-120)	62 (55-70)	9 (7-12)	115 (100-130)	43 (35-50)
2308	The Bahamas	Male	60 to 64	71 (46-104)	99 (87-116)	12 (10-15)	159 (139-179)	62 (53-72)
2309	The Bahamas	Male	65 to 69	56 (39-80)	161 (137-188)	17 (13-21)	253 (224-283)	102 (88-118)
2310	The Bahamas	Male	70 to <75	46 (30-70)	227 (191-273)	23 (19-28)	367 (324-408)	144 (125-165)
2311	The Gambia	Female	40 to 44	26 (20-34)	13 (9-16)	2 (2-3)	40 (35-47)	4 (3-6)
2312	The Gambia	Female	45 to 49	37 (26-49)	16 (13-19)	4 (3-5)	53 (46-63)	8 (6-11)
2313	The Gambia	Female	50 to 54	42 (33-55)	20 (17-24)	6 (4-7)	78 (64-94)	17 (13-23)
2314	The Gambia	Female	55 to 59	43 (30-59)	22 (19-26)	8 (6-10)	113 (90-137)	30 (22-40)
2315	The Gambia	Female	60 to 64	44 (31-62)	27 (23-31)	11 (9-14)	175 (139-205)	55 (40-69)
2316	The Gambia	Female	65 to 69	47 (32-65)	28 (25-34)	15 (11-20)	273 (215-321)	97 (71-126)
2317	The Gambia	Female	70 to <75	46 (33-63)	33 (29-37)	22 (18-27)	433 (348-492)	158 (120-192)
2318	The Gambia	Male	40 to 44	23 (17-30)	11 (8-16)	3 (2-3)	72 (63-83)	7 (5-10)
2319	The Gambia	Male	45 to 49	29 (21-39)	15 (11-20)	5 (3-6)	88 (74-102)	13 (10-17)
2320	The Gambia	Male	50 to 54	33 (25-43)	19 (15-25)	7 (5-8)	111 (96-128)	22 (16-28)
2321	The Gambia	Male	55 to 59	34 (24-47)	25 (19-31)	9 (7-12)	144 (127-165)	34 (27-43)
2322	The Gambia	Male	60 to 64	36 (25-50)	31 (26-37)	12 (10-15)	209 (185-237)	61 (49-74)
2323	The Gambia	Male	65 to 69	37 (26-52)	37 (32-43)	17 (13-21)	307 (274-348)	90 (74-108)
2324	The Gambia	Male	70 to <75	36 (25-50)	48 (42-56)	23 (18-27)	468 (429-527)	149 (124-178)
2325	Timor-Leste	Female	40 to 44	44 (34-59)	11 (9-14)	3 (2-3)	25 (17-33)	4 (1-6)
2326	Timor-Leste	Female	45 to 49	56 (41-74)	16 (13-20)	5 (3-7)	37 (27-49)	8 (5-12)
2327	Timor-Leste	Female	50 to 54	65 (50-84)	21 (17-25)	8 (6-9)	57 (41-75)	19 (12-26)
2328	Timor-Leste	Female	55 to 59	73 (54-96)	26 (22-31)	11 (8-15)	86 (61-116)	31 (21-44)
2329	Timor-Leste	Female	60 to 64	69 (49-92)	32 (27-38)	15 (11-19)	134 (100-177)	53 (37-72)
2330	Timor-Leste	Female	65 to 69	53 (38-71)	38 (32-45)	19 (13-24)	209 (158-280)	93 (68-127)
2331	Timor-Leste	Female	70 to <75	40 (26-56)	43 (38-50)	29 (23-35)	337 (262-443)	158 (118-210)
2332	Timor-Leste	Male	40 to 44	36 (27-49)	4 (3-5)	3 (2-4)	31 (22-41)	8 (1-13)
2333	Timor-Leste	Male	45 to 49	36 (26-49)	6 (5-8)	6 (4-7)	43 (31-58)	14 (5-21)
2334	Timor-Leste	Male	50 to 54	39 (28-52)	11 (8-14)	9 (7-11)	64 (46-84)	24 (14-34)
2335	Timor-Leste	Male	55 to 59	44 (31-60)	18 (13-22)	13 (10-17)	96 (69-127)	39 (25-52)
2336	Timor-Leste	Male	60 to 64	44 (31-60)	29 (23-36)	18 (14-23)	150 (113-197)	61 (41-82)
2337	Timor-Leste	Male	65 to 69	40 (28-55)	40 (32-49)	24 (17-31)	230 (175-297)	97 (70-127)
2338	Timor-Leste	Male	70 to <75	35 (24-51)	54 (44-65)	32 (26-39)	363 (281-459)	151 (109-193)
2339	Togo	Female	40 to 44	25 (20-33)	13 (10-16)	2 (2-3)	58 (51-67)	6 (4-8)
2340	Togo	Female	45 to 49	35 (24-46)	17 (13-21)	4 (3-5)	72 (62-86)	11 (8-15)
2341	Togo	Female	50 to 54	40 (30-51)	21 (17-25)	6 (5-7)	98 (80-118)	22 (16-28)
2342	Togo	Female	55 to 59	41 (28-56)	27 (22-32)	8 (6-11)	139 (111-165)	36 (27-48)
2343	Togo	Female	60 to 64	43 (30-58)	36 (30-42)	11 (9-14)	211 (170-242)	64 (49-80)
2344	Togo	Female	65 to 69	46 (33-65)	40 (34-45)	16 (12-21)	329 (266-372)	116 (88-142)
2345	Togo	Female	70 to <75	46 (33-62)	43 (37-49)	23 (19-28)	514 (430-568)	186 (147-223)
2346	Togo	Male	40 to 44	29 (21-38)	6 (5-8)	3 (2-4)	98 (86-110)	13 (9-18)
2347	Togo	Male	45 to 49	35 (25-48)	10 (8-12)	5 (4-7)	122 (106-140)	22 (16-29)
2348	Togo	Male	50 to 54	39 (29-53)	15 (13-18)	8 (6-9)	153 (135-175)	34 (26-44)
2349	Togo	Male	55 to 59	40 (28-57)	22 (19-25)	10 (8-13)	197 (175-227)	51 (39-62)
2350	Togo	Male	60 to 64	41 (28-56)	34 (30-38)	14 (11-17)	279 (251-322)	85 (68-103)
2351	Togo	Male	65 to 69	41 (29-57)	50 (45-57)	18 (14-23)	403 (366-463)	125 (102-149)
2352	Togo	Male	70 to <75	39 (26-54)	70 (61-79)	24 (20-29)	611 (556-698)	201 (163-243)
2353	Tonga	Female	40 to 44	228 (165-296)	23 (19-28)	3 (2-4)	33 (23-44)	5 (3-7)
2354	Tonga	Female	45 to 49	399 (257-550)	27 (23-32)	5 (4-7)	49 (36-63)	8 (6-12)
2355	Tonga	Female	50 to 54	498 (385-626)	33 (28-38)	7 (6-9)	72 (54-93)	11 (8-16)
2356	Tonga	Female	55 to 59	525 (407-681)	42 (36-49)	10 (7-13)	106 (83-135)	18 (13-24)
2357	Tonga	Female	60 to 64	427 (285-600)	50 (44-58)	13 (10-17)	156 (126-196)	29 (21-40)
2358	Tonga	Female	65 to 69	205 (150-279)	66 (57-75)	18 (13-23)	237 (192-293)	55 (41-71)
2359	Tonga	Female	70 to <75	78 (48-125)	86 (74-98)	28 (23-34)	379 (309-465)	97 (74-127)
2360	Tonga	Male	40 to 44	210 (151-280)	12 (10-14)	3 (2-4)	53 (39-69)	14 (9-19)
2361	Tonga	Male	45 to 49	348 (229-464)	18 (15-21)	5 (4-7)	77 (58-99)	20 (14-28)
2362	Tonga	Male	50 to 54	385 (288-488)	25 (21-30)	8 (6-9)	113 (89-142)	34 (25-44)
2363	Tonga	Male	55 to 59	320 (243-418)	36 (31-42)	11 (8-14)	166 (136-201)	52 (40-66)
2364	Tonga	Male	60 to 64	237 (148-354)	55 (48-64)	16 (13-19)	248 (207-291)	80 (64-99)
2365	Tonga	Male	65 to 69	136 (97-192)	94 (81-108)	21 (16-28)	368 (314-420)	108 (88-131)
2366	Tonga	Male	70 to <75	72 (44-114)	118 (104-135)	29 (24-35)	555 (481-627)	169 (138-198)
2367	Trinidad and Tobago	Female	40 to 44	105 (71-147)	23 (20-26)	2 (2-3)	23 (21-26)	4 (3-5)
2368	Trinidad and Tobago	Female	45 to 49	142 (85-220)	32 (28-37)	4 (3-6)	34 (30-38)	8 (7-10)

				Diabetes type 2	Cancers	Hemorrhagic stroke	All-cause mortality	CVD mortality
ID	Country	Sex	Age	Rate (95% CI)	Rate (95% CI)	Rate (95% CI)	Rate (95% CI)	Rate (95% CI)
2369	Trinidad and Tobago	Female	50 to 54	186 (139-240)	40 (35-46)	6 (5-8)	51 (46-56)	14 (12-17)
2370	Trinidad and Tobago	Female	55 to 59	235 (165-301)	51 (45-58)	8 (6-11)	74 (67-82)	22 (19-27)
2371	Trinidad and Tobago	Female	60 to 64	212 (128-288)	62 (55-70)	12 (9-14)	116 (105-128)	40 (35-47)
2372	Trinidad and Tobago	Female	65 to 69	117 (84-158)	72 (64-80)	16 (12-20)	169 (152-187)	63 (54-72)
2373	Trinidad and Tobago	Female	70 to <75	62 (37-109)	79 (72-88)	22 (18-27)	248 (224-274)	99 (86-112)
2374	Trinidad and Tobago	Male	40 to 44	219 (162-275)	10 (8-13)	3 (2-3)	43 (38-48)	9 (7-11)
2375	Trinidad and Tobago	Male	45 to 49	482 (343-621)	15 (13-18)	5 (4-6)	59 (53-67)	17 (13-20)
2376	Trinidad and Tobago	Male	50 to 54	503 (388-619)	25 (22-28)	7 (6-9)	86 (76-96)	27 (23-33)
2377	Trinidad and Tobago	Male	55 to 59	282 (221-360)	44 (39-49)	10 (7-13)	128 (114-143)	44 (38-51)
2378	Trinidad and Tobago	Male	60 to 64	144 (67-257)	74 (64-85)	14 (11-17)	195 (176-214)	72 (62-82)
2379	Trinidad and Tobago	Male	65 to 69	88 (52-134)	116 (95-137)	20 (15-25)	289 (262-317)	111 (97-127)
2380	Trinidad and Tobago	Male	70 to <75	57 (35-91)	173 (136-204)	28 (23-33)	454 (412-499)	182 (159-207)
2381	Tunisia	Female	40 to 44	61 (45-81)	14 (12-18)	2 (1-2)	11 (8-14)	2 (2-3)
2382	Tunisia	Female	45 to 49	76 (53-103)	20 (16-24)	3 (2-4)	18 (13-23)	4 (3-6)
2383	Tunisia	Female	50 to 54	86 (63-114)	23 (20-28)	4 (3-5)	27 (21-36)	8 (5-10)
2384	Tunisia	Female	55 to 59	93 (67-124)	27 (23-32)	5 (4-8)	42 (32-56)	14 (10-19)
2385	Tunisia	Female	60 to 64	86 (60-119)	32 (28-39)	8 (6-10)	71 (54-92)	27 (19-36)
2386	Tunisia	Female	65 to 69	68 (49-92)	37 (32-42)	12 (8-16)	118 (89-149)	50 (36-65)
2387	Tunisia	Female	70 to <75	54 (36-79)	44 (39-49)	17 (14-21)	203 (153-246)	92 (66-116)
2388	Tunisia	Male	40 to 44	62 (47-81)	8 (7-9)	2 (2-3)	23 (18-30)	6 (4-9)
2389	Tunisia	Male	45 to 49	80 (58-107)	14 (13-16)	3 (3-5)	35 (27-46)	12 (8-16)
2390	Tunisia	Male	50 to 54	93 (71-121)	24 (22-27)	6 (4-7)	55 (42-72)	21 (15-28)
2391	Tunisia	Male	55 to 59	100 (73-133)	37 (33-42)	8 (6-11)	86 (64-115)	33 (24-47)
2392	Tunisia	Male	60 to 64	96 (67-130)	56 (50-62)	11 (9-14)	141 (108-180)	60 (45-79)
2393	Tunisia	Male	65 to 69	82 (59-113)	79 (71-89)	15 (11-20)	224 (168-287)	99 (71-128)
2394	Tunisia	Male	70 to <75	67 (45-96)	101 (91-113)	21 (17-26)	360 (265-454)	162 (115-208)
2395	Turkey	Female	40 to 44	53 (42-66)	13 (11-16)	2 (1-3)	9 (7-11)	2 (1-2)
2396	Turkey	Female	45 to 49	65 (49-84)	19 (16-22)	3 (2-4)	14 (10-17)	3 (2-4)
2397	Turkey	Female	50 to 54	75 (60-94)	24 (21-27)	4 (3-5)	21 (17-27)	5 (4-7)
2398	Turkey	Female	55 to 59	83 (63-106)	29 (26-33)	5 (4-7)	33 (26-42)	10 (7-13)
2399	Turkey	Female	60 to 64	80 (59-105)	38 (34-42)	8 (6-10)	57 (45-71)	18 (14-24)
2400	Turkey	Female	65 to 69	66 (48-90)	46 (42-51)	12 (8-15)	94 (73-117)	33 (25-43)
2401	Turkey	Female	70 to <75	56 (39-78)	56 (51-62)	17 (13-20)	162 (125-206)	61 (45-80)
2402	Turkey	Male	40 to 44	50 (40-61)	10 (9-12)	2 (2-3)	20 (16-25)	6 (4-8)
2403	Turkey	Male	45 to 49	67 (49-85)	18 (16-20)	3 (2-4)	31 (24-39)	10 (7-13)
2404	Turkey	Male	50 to 54	79 (61-97)	32 (29-35)	5 (4-6)	50 (39-62)	16 (12-21)
2405	Turkey	Male	55 to 59	86 (65-111)	50 (45-55)	7 (5-9)	78 (61-98)	25 (19-33)
2406	Turkey	Male	60 to 64	82 (61-106)	76 (69-83)	10 (8-12)	129 (103-157)	43 (34-54)
2407	Turkey	Male	65 to 69	68 (51-90)	103 (94-112)	13 (10-17)	195 (157-241)	67 (52-85)
2408	Turkey	Male	70 to <75	55 (38-76)	131 (120-145)	19 (15-23)	306 (245-383)	107 (83-138)
2409	Turkmenistan	Female	40 to 44	38 (30-47)	21 (18-24)	4 (4-5)	23 (21-25)	7 (6-8)
2410	Turkmenistan	Female	45 to 49	43 (32-55)	27 (23-33)	8 (6-10)	35 (32-38)	14 (12-16)
2411	Turkmenistan	Female	50 to 54	43 (32-55)	31 (26-36)	13 (10-15)	55 (51-59)	26 (23-29)
2412	Turkmenistan	Female	55 to 59	39 (28-50)	35 (30-42)	19 (14-24)	88 (81-95)	47 (42-51)
2413	Turkmenistan	Female	60 to 64	33 (24-45)	41 (35-49)	28 (23-34)	144 (133-155)	88 (81-96)
2414	Turkmenistan	Female	65 to 69	27 (19-38)	47 (41-55)	42 (33-51)	224 (207-242)	152 (140-165)
2415	Turkmenistan	Female	70 to <75	20 (14-31)	52 (44-64)	54 (45-63)	357 (329-387)	265 (245-287)
2416	Turkmenistan	Male	40 to 44	44 (34-55)	9 (8-11)	8 (6-9)	53 (48-57)	19 (16-22)
2417	Turkmenistan	Male	45 to 49	45 (32-60)	15 (13-20)	14 (11-18)	76 (70-82)	35 (31-39)
2418	Turkmenistan	Male	50 to 54	42 (30-57)	26 (22-31)	22 (18-26)	113 (105-122)	61 (55-67)
2419	Turkmenistan	Male	55 to 59	36 (26-48)	41 (35-49)	32 (25-40)	166 (155-177)	98 (90-106)
2420	Turkmenistan	Male	60 to 64	32 (20-45)	60 (52-71)	44 (36-53)	252 (238-268)	162 (151-174)
2421	Turkmenistan	Male	65 to 69	29 (20-40)	81 (68-94)	57 (46-69)	370 (350-391)	255 (239-273)
2422	Turkmenistan	Male	70 to <75	24 (16-35)	103 (83-127)	73 (60-85)	555 (523-588)	407 (379-433)
2423	Uganda	Female	40 to 44	29 (22-37)	19 (16-23)	2 (1-2)	59 (50-71)	4 (3-6)
2424	Uganda	Female	45 to 49	35 (24-49)	27 (23-32)	3 (2-4)	78 (63-97)	8 (5-11)
2425	Uganda	Female	50 to 54	43 (32-59)	38 (33-44)	4 (3-5)	112 (87-138)	15 (10-21)
2426	Uganda	Female	55 to 59	55 (37-78)	42 (37-49)	6 (5-8)	150 (115-182)	27 (19-37)
2427	Uganda	Female	60 to 64	63 (46-86)	53 (47-61)	10 (8-12)	223 (175-263)	49 (36-64)
2428	Uganda	Female	65 to 69	70 (48-98)	61 (54-68)	15 (11-20)	343 (268-396)	91 (67-113)
2429	Uganda	Female	70 to <75	66 (45-94)	60 (54-66)	23 (19-27)	527 (429-604)	161 (125-199)
2430	Uganda	Male	40 to 44	39 (29-51)	8 (7-9)	2 (1-2)	98 (83-113)	8 (6-11)
2431	Uganda	Male	45 to 49	61 (40-85)	12 (11-14)	4 (3-5)	121 (103-141)	14 (10-19)
2432	Uganda	Male	50 to 54	73 (53-94)	20 (18-22)	5 (4-6)	156 (134-186)	21 (16-28)
2433	Uganda	Male	55 to 59	74 (50-104)	32 (29-35)	7 (5-10)	197 (171-233)	33 (25-43)
2434	Uganda	Male	60 to 64	74 (50-104)	55 (49-61)	11 (9-14)	280 (247-332)	56 (45-72)
2435	Uganda	Male	65 to 69	72 (49-100)	80 (70-91)	16 (12-21)	405 (362-477)	93 (74-117)
2436	Uganda	Male	70 to <75	66 (44-91)	114 (97-133)	23 (19-28)	612 (550-711)	154 (125-192)
2437	Ukraine	Female	40 to 44	36 (29-44)	21 (18-24)	3 (2-4)	24 (15-35)	5 (3-7)
2438	Ukraine	Female	45 to 49	31 (23-43)	31 (27-36)	5 (4-7)	32 (21-46)	8 (5-12)

ID	Country	Sex	Age	Diabetes type 2<br>Rate (95% CI)	Cancers<br>Rate (95% CI)	Hemorrhagic stroke<br>Rate (95% CI)	All-cause mortality<br>Rate (95% CI)	CVD mortality<br>Rate (95% CI)
2439	Ukraine	Female	50 to 54	29 (20-41)	42 (37-48)	7 (6-9)	46 (31-65)	15 (10-21)
2440	Ukraine	Female	55 to 59	30 (22-40)	55 (48-62)	10 (7-13)	71 (49-99)	30 (21-43)
2441	Ukraine	Female	60 to 64	28 (20-38)	69 (61-78)	14 (11-17)	109 (76-150)	59 (42-84)
2442	Ukraine	Female	65 to 69	22 (16-30)	76 (68-86)	18 (13-23)	169 (117-234)	110 (76-154)
2443	Ukraine	Female	70 to <75	18 (12-26)	81 (72-93)	24 (19-29)	286 (196-399)	213 (146-299)
2444	Ukraine	Male	40 to 44	43 (35-54)	14 (13-16)	5 (4-6)	73 (48-100)	17 (11-25)
2445	Ukraine	Male	45 to 49	37 (27-50)	26 (23-29)	9 (7-12)	100 (66-138)	30 (20-43)
2446	Ukraine	Male	50 to 54	32 (23-46)	49 (44-54)	13 (10-16)	142 (95-194)	53 (35-74)
2447	Ukraine	Male	55 to 59	30 (22-40)	81 (74-89)	17 (13-22)	201 (138-268)	89 (61-120)
2448	Ukraine	Male	60 to 64	27 (19-38)	118 (107-130)	21 (17-26)	296 (211-385)	160 (113-209)
2449	Ukraine	Male	65 to 69	23 (16-33)	152 (138-168)	26 (20-34)	402 (289-517)	246 (177-318)
2450	Ukraine	Male	70 to <75	19 (13-28)	168 (151-189)	31 (25-38)	570 (412-728)	390 (283-501)
2451	United Arab Emirates	Female	40 to 44	94 (70-120)	18 (14-23)	4 (3-4)	20 (14-27)	6 (4-9)
2452	United Arab Emirates	Female	45 to 49	124 (84-169)	29 (23-36)	5 (4-7)	30 (22-40)	10 (7-14)
2453	United Arab Emirates	Female	50 to 54	142 (104-188)	35 (29-43)	7 (5-8)	46 (34-59)	17 (12-23)
2454	United Arab Emirates	Female	55 to 59	147 (108-194)	42 (33-52)	8 (6-10)	67 (52-86)	27 (20-36)
2455	United Arab Emirates	Female	60 to 64	130 (89-183)	43 (37-49)	10 (8-13)	100 (80-124)	43 (34-54)
2456	United Arab Emirates	Female	65 to 69	90 (67-125)	44 (39-49)	14 (10-18)	155 (129-187)	71 (57-89)
2457	United Arab Emirates	Female	70 to <75	63 (40-94)	62 (53-72)	19 (15-23)	248 (208-301)	114 (94-143)
2458	United Arab Emirates	Male	40 to 44	97 (70-126)	5 (4-6)	3 (2-3)	28 (19-38)	10 (6-14)
2459	United Arab Emirates	Male	45 to 49	143 (97-206)	8 (6-10)	5 (3-6)	44 (31-58)	18 (13-26)
2460	United Arab Emirates	Male	50 to 54	162 (115-222)	13 (11-17)	7 (6-8)	67 (49-89)	31 (23-43)
2461	United Arab Emirates	Male	55 to 59	153 (109-205)	23 (19-29)	10 (7-13)	101 (77-135)	46 (34-63)
2462	United Arab Emirates	Male	60 to 64	134 (91-190)	38 (34-44)	12 (10-15)	153 (121-201)	70 (54-93)
2463	United Arab Emirates	Male	65 to 69	105 (75-149)	56 (49-64)	15 (11-20)	235 (189-296)	107 (84-137)
2464	United Arab Emirates	Male	70 to <75	79 (49-123)	82 (73-93)	21 (16-26)	366 (304-445)	157 (111-201)
2465	United Kingdom	Female	40 to 44	22 (17-28)	29 (26-33)	2 (2-3)	10 (10-11)	1 (1-1)
2466	United Kingdom	Female	45 to 49	25 (17-34)	43 (38-51)	4 (3-5)	16 (16-16)	2 (2-2)
2467	United Kingdom	Female	50 to 54	30 (23-40)	60 (55-69)	5 (4-6)	26 (25-26)	4 (4-4)
2468	United Kingdom	Female	55 to 59	39 (28-51)	81 (73-93)	6 (4-7)	40 (39-41)	6 (6-7)
2469	United Kingdom	Female	60 to 64	42 (31-56)	105 (96-118)	7 (6-9)	62 (61-64)	11 (10-11)
2470	United Kingdom	Female	65 to 69	42 (30-58)	135 (124-149)	10 (8-13)	98 (96-101)	20 (19-21)
2471	United Kingdom	Female	70 to <75	37 (26-53)	167 (153-188)	16 (14-19)	165 (161-169)	40 (38-41)
2472	United Kingdom	Male	40 to 44	26 (20-33)	17 (15-22)	2 (2-3)	17 (17-18)	4 (3-4)
2473	United Kingdom	Male	45 to 49	32 (23-44)	28 (24-38)	4 (3-5)	25 (24-25)	6 (6-7)
2474	United Kingdom	Male	50 to 54	39 (30-50)	49 (42-59)	6 (4-7)	38 (37-39)	11 (10-11)
2475	United Kingdom	Male	55 to 59	47 (35-62)	84 (73-101)	7 (5-9)	61 (59-62)	18 (17-18)
2476	United Kingdom	Male	60 to 64	50 (37-66)	138 (123-160)	10 (8-12)	97 (95-99)	28 (27-29)
2477	United Kingdom	Male	65 to 69	48 (35-67)	215 (194-245)	13 (10-17)	153 (150-157)	44 (43-46)
2478	United Kingdom	Male	70 to <75	42 (28-60)	300 (269-346)	21 (17-24)	254 (249-260)	77 (75-80)
2479	United States	Female	40 to 44	43 (38-49)	35 (32-40)	3 (2-3)	16 (15-16)	3 (3-3)
2480	United States	Female	45 to 49	59 (48-72)	52 (47-61)	4 (3-6)	25 (24-25)	5 (4-5)
2481	United States	Female	50 to 54	82 (72-94)	74 (67-84)	6 (5-7)	38 (37-39)	8 (8-8)
2482	United States	Female	55 to 59	113 (97-131)	100 (91-112)	6 (5-8)	55 (54-56)	12 (12-13)
2483	United States	Female	60 to 64	109 (92-129)	133 (121-147)	8 (6-10)	79 (77-81)	19 (18-19)
2484	United States	Female	65 to 69	72 (60-87)	172 (156-192)	10 (8-13)	118 (115-121)	29 (28-30)
2485	United States	Female	70 to <75	42 (28-58)	211 (189-240)	16 (13-18)	188 (184-193)	50 (49-53)
2486	United States	Male	40 to 44	61 (54-69)	21 (18-26)	3 (2-3)	25 (24-26)	6 (5-6)
2487	United States	Male	45 to 49	77 (61-96)	38 (33-48)	4 (3-5)	38 (37-40)	10 (10-11)
2488	United States	Male	50 to 54	109 (95-124)	72 (66-84)	6 (5-7)	61 (59-63)	18 (17-18)
2489	United States	Male	55 to 59	157 (136-183)	124 (113-143)	8 (7-11)	91 (89-94)	28 (27-29)
2490	United States	Male	60 to 64	153 (129-181)	197 (181-224)	11 (9-14)	131 (128-134)	41 (40-42)
2491	United States	Male	65 to 69	97 (80-115)	283 (259-322)	14 (11-18)	183 (178-187)	56 (54-58)
2492	United States	Male	70 to <75	54 (35-76)	355 (320-405)	21 (18-25)	277 (270-283)	86 (83-89)
2493	Uruguay	Female	40 to 44	22 (17-29)	24 (21-27)	2 (2-3)	15 (14-17)	2 (2-3)
2494	Uruguay	Female	45 to 49	31 (22-44)	33 (29-37)	4 (3-6)	22 (20-25)	4 (3-4)
2495	Uruguay	Female	50 to 54	43 (34-55)	44 (40-49)	6 (5-7)	36 (33-40)	6 (5-8)
2496	Uruguay	Female	55 to 59	57 (44-73)	55 (50-60)	7 (5-9)	54 (49-59)	11 (9-13)
2497	Uruguay	Female	60 to 64	56 (42-72)	68 (62-75)	9 (7-11)	80 (73-87)	18 (16-21)
2498	Uruguay	Female	65 to 69	40 (31-52)	85 (79-92)	12 (9-15)	122 (112-133)	33 (27-38)
2499	Uruguay	Female	70 to <75	27 (18-40)	101 (94-109)	17 (14-20)	192 (175-211)	58 (51-66)
2500	Uruguay	Male	40 to 44	28 (22-37)	11 (10-12)	2 (2-3)	26 (23-28)	4 (3-5)
2501	Uruguay	Male	45 to 49	38 (26-54)	21 (19-22)	4 (3-5)	40 (36-44)	8 (7-10)
2502	Uruguay	Male	50 to 54	53 (42-66)	40 (37-43)	6 (5-7)	66 (60-72)	16 (13-18)
2503	Uruguay	Male	55 to 59	72 (54-93)	71 (66-77)	9 (7-12)	110 (100-119)	27 (23-31)
2504	Uruguay	Male	60 to 64	70 (51-93)	116 (107-126)	13 (10-15)	172 (159-186)	46 (40-52)
2505	Uruguay	Male	65 to 69	48 (37-62)	174 (160-189)	17 (13-21)	270 (249-291)	76 (67-86)
2506	Uruguay	Male	70 to <75	30 (20-44)	229 (209-252)	23 (19-27)	405 (375-437)	120 (105-135)
2507	Uzbekistan	Female	40 to 44	43 (34-52)	19 (17-22)	3 (3-4)	27 (22-34)	8 (6-10)
2508	Uzbekistan	Female	45 to 49	51 (34-67)	27 (23-32)	6 (4-8)	41 (33-51)	15 (12-20)

				Diabetes type 2	Cancers	Hemorrhagic stroke	All-cause mortality	CVD mortality
**ID**	**Country**	**Sex**	**Age**	**Rate (95% CI)**	**Rate (95% CI)**	**Rate (95% CI)**	**Rate (95% CI)**	**Rate (95% CI)**
2509	Uzbekistan	Female	50 to 54	52 (37-67)	33 (28-38)	9 (8-11)	64 (53-77)	28 (23-35)
2510	Uzbekistan	Female	55 to 59	47 (36-59)	36 (31-43)	13 (10-18)	98 (82-116)	50 (42-60)
2511	Uzbekistan	Female	60 to 64	39 (27-54)	40 (34-48)	19 (15-23)	154 (132-177)	90 (78-105)
2512	Uzbekistan	Female	65 to 69	29 (21-41)	44 (38-51)	25 (19-31)	237 (205-272)	156 (135-180)
2513	Uzbekistan	Female	70 to <75	21 (14-32)	46 (38-58)	32 (26-38)	380 (332-429)	282 (246-320)
2514	Uzbekistan	Male	40 to 44	52 (41-64)	8 (7-10)	5 (4-6)	52 (42-64)	18 (14-23)
2515	Uzbekistan	Male	45 to 49	54 (39-70)	14 (12-19)	10 (7-12)	76 (63-93)	34 (27-42)
2516	Uzbekistan	Male	50 to 54	51 (36-68)	23 (19-28)	14 (12-18)	114 (97-138)	59 (50-71)
2517	Uzbekistan	Male	55 to 59	43 (31-56)	35 (29-43)	20 (15-26)	169 (147-199)	96 (83-114)
2518	Uzbekistan	Male	60 to 64	36 (24-51)	53 (44-65)	26 (21-32)	259 (231-301)	160 (142-186)
2519	Uzbekistan	Male	65 to 69	31 (22-42)	73 (59-92)	32 (24-40)	379 (342-428)	254 (229-288)
2520	Uzbekistan	Male	70 to <75	24 (16-35)	92 (71-120)	41 (34-49)	578 (523-643)	425 (384-473)
2521	Vanuatu	Female	40 to 44	154 (119-193)	16 (11-22)	5 (4-6)	65 (51-87)	25 (18-35)
2522	Vanuatu	Female	45 to 49	225 (161-294)	20 (14-25)	9 (7-12)	92 (74-119)	39 (29-52)
2523	Vanuatu	Female	50 to 54	270 (212-339)	24 (18-30)	13 (10-16)	134 (109-170)	56 (43-74)
2524	Vanuatu	Female	55 to 59	292 (228-373)	30 (23-36)	17 (13-22)	194 (160-244)	88 (70-113)
2525	Vanuatu	Female	60 to 64	249 (181-341)	34 (29-39)	23 (18-28)	278 (232-344)	132 (107-166)
2526	Vanuatu	Female	65 to 69	142 (108-187)	46 (39-53)	30 (22-38)	413 (347-510)	203 (163-254)
2527	Vanuatu	Female	70 to <75	73 (45-113)	58 (51-66)	45 (36-53)	650 (546-798)	321 (259-398)
2528	Vanuatu	Male	40 to 44	129 (97-167)	7 (6-8)	5 (4-6)	86 (68-113)	37 (28-49)
2529	Vanuatu	Male	45 to 49	174 (128-228)	10 (9-12)	9 (7-12)	123 (97-163)	58 (43-77)
2530	Vanuatu	Male	50 to 54	189 (143-241)	15 (12-17)	13 (11-16)	175 (140-227)	89 (67-115)
2531	Vanuatu	Male	55 to 59	176 (133-229)	22 (19-26)	18 (14-24)	244 (200-307)	125 (98-156)
2532	Vanuatu	Male	60 to 64	145 (100-200)	32 (28-37)	24 (20-30)	348 (295-419)	175 (143-212)
2533	Vanuatu	Male	65 to 69	97 (71-130)	50 (45-57)	32 (25-40)	490 (425-570)	226 (191-268)
2534	Vanuatu	Male	70 to <75	62 (40-92)	61 (54-68)	45 (37-52)	715 (631-803)	327 (277-377)
2535	Venezuela	Female	40 to 44	47 (35-61)	23 (20-27)	2 (2-3)	16 (12-20)	3 (2-4)
2536	Venezuela	Female	45 to 49	52 (36-73)	33 (28-39)	4 (3-6)	23 (18-30)	5 (4-7)
2537	Venezuela	Female	50 to 54	57 (41-78)	44 (38-51)	6 (5-8)	38 (30-47)	9 (7-12)
2538	Venezuela	Female	55 to 59	62 (46-86)	56 (49-66)	8 (6-10)	59 (47-73)	16 (12-20)
2539	Venezuela	Female	60 to 64	61 (44-85)	66 (58-76)	10 (8-13)	84 (67-104)	25 (19-32)
2540	Venezuela	Female	65 to 69	55 (38-79)	75 (66-85)	13 (10-17)	122 (98-152)	40 (31-51)
2541	Venezuela	Female	70 to <75	49 (32-72)	93 (82-107)	18 (15-22)	201 (160-252)	73 (56-93)
2542	Venezuela	Male	40 to 44	69 (53-88)	10 (8-13)	2 (2-3)	34 (27-44)	6 (4-9)
2543	Venezuela	Male	45 to 49	91 (65-126)	17 (14-22)	4 (3-6)	45 (36-59)	11 (8-15)
2544	Venezuela	Male	50 to 54	97 (72-133)	32 (27-37)	6 (5-8)	70 (56-91)	22 (16-29)
2545	Venezuela	Male	55 to 59	88 (64-118)	55 (47-65)	9 (7-12)	107 (86-137)	37 (29-47)
2546	Venezuela	Male	60 to 64	77 (50-109)	87 (76-101)	12 (9-15)	151 (124-189)	56 (44-71)
2547	Venezuela	Male	65 to 69	64 (46-93)	126 (108-148)	15 (11-20)	211 (174-264)	81 (64-102)
2548	Venezuela	Male	70 to <75	58 (39-85)	177 (150-211)	21 (17-25)	328 (271-408)	129 (103-163)
2549	Vietnam	Female	40 to 44	43 (32-57)	12 (10-14)	2 (2-3)	17 (14-22)	3 (2-4)
2550	Vietnam	Female	45 to 49	53 (36-72)	18 (15-21)	5 (4-6)	26 (22-34)	6 (5-8)
2551	Vietnam	Female	50 to 54	71 (55-90)	24 (20-27)	7 (6-9)	42 (33-52)	11 (9-15)
2552	Vietnam	Female	55 to 59	97 (71-128)	31 (27-35)	10 (8-13)	65 (52-79)	18 (14-24)
2553	Vietnam	Female	60 to 64	98 (72-130)	40 (35-45)	13 (11-17)	104 (84-122)	30 (24-38)
2554	Vietnam	Female	65 to 69	74 (55-99)	47 (41-52)	18 (14-23)	163 (138-187)	56 (44-68)
2555	Vietnam	Female	70 to <75	55 (36-80)	55 (48-62)	27 (23-32)	262 (241-297)	99 (84-118)
2556	Vietnam	Male	40 to 44	35 (27-46)	12 (10-14)	3 (2-4)	36 (27-47)	7 (4-10)
2557	Vietnam	Male	45 to 49	37 (26-50)	18 (16-22)	5 (4-7)	54 (43-69)	13 (9-17)
2558	Vietnam	Male	50 to 54	41 (31-54)	31 (27-35)	10 (8-12)	85 (69-105)	24 (18-31)
2559	Vietnam	Male	55 to 59	48 (35-64)	46 (40-52)	16 (12-20)	131 (106-156)	43 (34-54)
2560	Vietnam	Male	60 to 64	50 (36-66)	66 (58-75)	22 (18-26)	201 (167-230)	72 (58-85)
2561	Vietnam	Male	65 to 69	45 (32-62)	98 (87-109)	27 (21-34)	307 (266-343)	114 (97-132)
2562	Vietnam	Male	70 to <75	39 (27-55)	127 (113-147)	38 (32-45)	472 (422-511)	185 (160-210)
2563	Yemen	Female	40 to 44	58 (44-73)	11 (7-15)	4 (3-4)	41 (36-48)	15 (12-19)
2564	Yemen	Female	45 to 49	72 (50-95)	16 (10-21)	5 (4-7)	60 (52-70)	26 (21-33)
2565	Yemen	Female	50 to 54	80 (60-105)	19 (14-25)	7 (6-9)	92 (79-105)	45 (36-54)
2566	Yemen	Female	55 to 59	82 (60-109)	24 (17-30)	10 (7-13)	136 (122-152)	72 (61-84)
2567	Yemen	Female	60 to 64	76 (53-107)	29 (25-33)	13 (11-17)	203 (184-225)	112 (98-126)
2568	Yemen	Female	65 to 69	61 (45-83)	36 (32-40)	19 (14-25)	315 (290-342)	181 (162-203)
2569	Yemen	Female	70 to <75	50 (34-72)	45 (39-51)	26 (21-32)	484 (455-525)	280 (250-313)
2570	Yemen	Male	40 to 44	47 (35-60)	5 (4-6)	3 (2-3)	54 (49-62)	16 (13-20)
2571	Yemen	Male	45 to 49	58 (42-77)	8 (6-10)	5 (3-6)	70 (62-81)	28 (23-34)
2572	Yemen	Male	50 to 54	65 (49-86)	13 (11-15)	7 (6-9)	96 (84-112)	45 (38-54)
2573	Yemen	Male	55 to 59	69 (50-90)	19 (16-22)	10 (8-14)	138 (122-157)	68 (58-81)
2574	Yemen	Male	60 to 64	68 (49-92)	28 (24-32)	14 (11-17)	205 (181-234)	106 (88-125)
2575	Yemen	Male	65 to 69	62 (45-85)	39 (35-45)	18 (13-23)	304 (267-349)	158 (132-187)
2576	Yemen	Male	70 to <75	53 (36-77)	64 (55-73)	25 (20-30)	463 (409-533)	230 (193-274)
2577	Zambia	Female	40 to 44	33 (25-44)	18 (14-22)	2 (1-2)	90 (64-120)	7 (4-12)
2578	Zambia	Female	45 to 49	37 (26-53)	23 (19-28)	3 (2-4)	110 (72-151)	13 (7-21)

				Diabetes type 2	Cancers	Hemorrhagic stroke	All-cause mortality	CVD mortality
ID	Country	Sex	Age	Rate (95% CI)	Rate (95% CI)	Rate (95% CI)	Rate (95% CI)	Rate (95% CI)
2579	Zambia	Female	50 to 54	45 (33-60)	31 (26-37)	5 (4-6)	145 (90-198)	22 (12-34)
2580	Zambia	Female	55 to 59	55 (38-75)	34 (29-40)	7 (5-9)	177 (105-249)	38 (21-58)
2581	Zambia	Female	60 to 64	62 (44-82)	45 (39-53)	10 (8-13)	251 (154-343)	64 (37-93)
2582	Zambia	Female	65 to 69	65 (46-87)	50 (44-58)	16 (11-21)	377 (230-508)	113 (66-159)
2583	Zambia	Female	70 to <75	60 (42-83)	52 (45-58)	23 (19-28)	579 (368-784)	198 (123-282)
2584	Zambia	Male	40 to 44	42 (31-54)	9 (7-11)	2 (2-3)	141 (108-178)	12 (7-18)
2585	Zambia	Male	45 to 49	65 (45-90)	13 (11-15)	4 (3-5)	175 (130-231)	22 (13-32)
2586	Zambia	Male	50 to 54	75 (57-98)	20 (17-24)	6 (5-8)	229 (163-303)	34 (21-49)
2587	Zambia	Male	55 to 59	72 (51-99)	31 (27-36)	9 (6-12)	284 (197-373)	56 (35-79)
2588	Zambia	Male	60 to 64	69 (47-96)	49 (40-61)	13 (10-16)	393 (275-496)	94 (61-127)
2589	Zambia	Male	65 to 69	64 (44-88)	69 (54-86)	18 (13-24)	546 (394-669)	137 (95-176)
2590	Zambia	Male	70 to <75	57 (38-82)	99 (75-124)	26 (21-30)	794 (598-938)	204 (146-257)
2591	Zimbabwe	Female	40 to 44	44 (34-56)	19 (15-23)	2 (2-3)	98 (78-123)	6 (3-9)
2592	Zimbabwe	Female	45 to 49	60 (43-81)	26 (20-31)	4 (3-5)	118 (86-157)	11 (7-17)
2593	Zimbabwe	Female	50 to 54	71 (54-92)	35 (28-41)	5 (4-6)	147 (104-190)	21 (12-30)
2594	Zimbabwe	Female	55 to 59	78 (56-108)	48 (39-56)	7 (5-10)	174 (115-226)	31 (19-44)
2595	Zimbabwe	Female	60 to 64	86 (64-115)	57 (45-65)	11 (8-13)	244 (167-309)	56 (37-75)
2596	Zimbabwe	Female	65 to 69	95 (70-125)	72 (58-83)	16 (11-21)	366 (248-460)	87 (57-115)
2597	Zimbabwe	Female	70 to <75	86 (61-118)	85 (71-96)	22 (18-27)	558 (390-708)	145 (98-193)
2598	Zimbabwe	Male	40 to 44	52 (39-67)	7 (6-9)	2 (2-3)	142 (121-167)	8 (6-12)
2599	Zimbabwe	Male	45 to 49	69 (48-98)	13 (12-15)	4 (3-5)	173 (136-223)	14 (9-20)
2600	Zimbabwe	Male	50 to 54	80 (60-107)	22 (20-25)	5 (4-7)	214 (164-275)	24 (16-34)
2601	Zimbabwe	Male	55 to 59	83 (59-112)	35 (31-40)	8 (6-10)	256 (193-327)	36 (25-50)
2602	Zimbabwe	Male	60 to 64	86 (61-116)	53 (45-59)	11 (8-13)	351 (267-437)	61 (43-82)
2603	Zimbabwe	Male	65 to 69	88 (65-116)	82 (67-96)	15 (11-19)	493 (387-594)	87 (62-113)
2604	Zimbabwe	Male	70 to <75	77 (52-106)	119 (94-143)	21 (17-25)	725 (579-846)	132 (99-168)

Appendix Table S3: Summary of the 10-y CVD risk thresholds by age and sex across countries: median (5th–95th percentiles) (base-case analysis)

	40–44 y	45–49 y	50–54 y	55–59 y	60–64 y	65–69 y	≥70 and <75 y	Overall
Men	14 (14–16)	15 (14–18)	15 (14–20)	16 (15–20)	17 (15–17)	18 (16–20)	18.5 (17–21)	16 (14–20)
Women	19 (18–21)	19 (19–22)	20 (19–24)	20 (19–25)	21 (20–25)	21 (20–24)	22 (21–24)	20 (19–24)

Appendix Table S4: Summary of the 10-y CVD risk thresholds (of appendix Figure 5) by age and sex across countries from the sensitivity analysis taking treatment effects from randomized controlled trials only

	**40–44 y**	**45–49 y**	**50–54 y**	**55–59 y**	**60–64 y**	**65–69 y**	**≥70 and <75 y**	**Overall**
Men	11 (11–12)	11 (11–13)	12 (11–14)	12 (12–15)	13 (12–15)	14 (13–15)	15 (13–17)	12 (11–16)
Women	15 (15–16)	15 (15–17)	16 (15–18)	16 (16–19.5)	17 (16–19·5)	17 (16–19)	18 (17–20)	16 (15–19·5)

Estimates: median (5th–95th percentiles)

References

1 Yebyo HG, Aschmann HE, Puhan MA. Finding the Balance Between Benefits and Harms When Using Statins for Primary Prevention of Cardiovascular Disease: Modeling study. *Ann Intern Med* 2019; 170. DOI:10.7326/M18-1279.

2 Yebyo HG, Aschmann HE, Yu T, Puhan MA. Should statin guidelines consider patient preferences? Eliciting preferences of benefit and harm outcomes of statins for primary prevention of cardiovascular disease in the sub-Saharan African and European contexts. *BMC Cardiovasc Disord* 2018; 18: 97.

3 Yusuf S, Bosch J, Dagenais G, *et al.* Cholesterol Lowering in intermediate-risk persons without cardiovascular disease. *N Engl J Med* 2016; 374: 2021–31.

4 Nakamura H, Arakawa K, Itakura H, *et al.* Primary prevention of cardiovascular disease with pravastatin in Japan (MEGA Study): a prospective randomised controlled trial. *Lancet* 2006; 368: 1155–63.

5 Yebyo HG, Aschmann HE, Kaufmann M, Puhan MA. Comparative effectiveness and safety of statins as a class and of specific statins for primary prevention of cardiovascular disease : A systematic review , meta-analysis , and network meta-analysis of randomized trials with 94 , 283 participants. *Am Heart J* 2019; 210: 10–3.

6 Hippisley-Cox J, Coupland C. Unintended effects of statins in men and women in England and Wales: population based cohort study using the QResearch database. *BMJ* 2010; 340: c2197–c2197.

7 Greenland S. Bayesian perspectives for epidemiological research: I. foundations and basic methods. *Int J Epidemiol* 2006; 35: 765–75.
